# *Amelogenesis imperfecta*: Next-generation sequencing sheds light on Witkop’s classification

**DOI:** 10.3389/fphys.2023.1130175

**Published:** 2023-05-09

**Authors:** Agnes Bloch-Zupan, Tristan Rey, Alexandra Jimenez-Armijo, Marzena Kawczynski, Naji Kharouf, O-Rare consortium, Muriel de La Dure-Molla, Emmanuelle Noirrit, Magali Hernandez, Clara Joseph-Beaudin, Serena Lopez, Corinne Tardieu, Béatrice Thivichon-Prince, ERN Cranio Consortium, Tatjana Dostalova, Milan Macek, International Consortium, Mustapha El Alloussi, Leila Qebibo, Supawich Morkmued, Patimaporn Pungchanchaikul, Blanca Urzúa Orellana, Marie-Cécile Manière, Bénédicte Gérard, Isaac Maximiliano Bugueno, Virginie Laugel-Haushalter

**Affiliations:** ^1^ Université de Strasbourg, Faculté de Chirurgie Dentaire, Strasbourg, France; ^2^ Université de Strasbourg, Institut d’études avancées (USIAS), Strasbourg, France; ^3^ Hôpitaux Universitaires de Strasbourg (HUS), Pôle de Médecine et Chirurgie Bucco-dentaires, Hôpital Civil, Centre de référence des maladies rares orales et dentaires, O-Rares, Filiére Santé Maladies rares TETE COU, European Reference Network ERN CRANIO, Strasbourg, France; ^4^ Université de Strasbourg, Institut de Génétique et de Biologie Moléculaire et Cellulaire (IGBMC), IN-SERM U1258, CNRS- UMR7104, Illkirch, France; ^5^ Eastman Dental Institute, University College London, London, United Kingdom; ^6^ Hôpitaux Universitaires de Strasbourg, Laboratoires de diagnostic génétique, Institut de Génétique Médicale d’Alsace, Strasbourg, France; ^7^ Université de Strasbourg, Laboratoire de Biomatériaux et Bioingénierie, Inserm UMR_S 1121, Strasbourg, France; ^8^ Rothschild Hospital, Public Assistance-Paris Hospitals (AP-HP), Reference Center for Rare Oral and Den-tal Diseases (O-Rares), Paris, France; ^9^ Centre Hospitalier Universitaire (CHU) Rangueil, Toulouse, Competence Center for Rare Oral and Den-tal Diseases, Toulouse, France; ^10^ Centre Hospitalier Régional Universitaire de Nancy, Université de Lorraine, Competence Center for Rare Oral and Dental Diseases, Nancy, France; ^11^ Centre Hospitalier Universitaire de Nice, Competence Center for Rare Oral and Dental Diseases, Nice, France; ^12^ Centre Hospitalier Universitaire de Nantes, Competence Center for Rare Oral and Dental Diseases, Nantes, France; ^13^ APHM, Hôpitaux Universitaires de Marseille, Hôpital Timone, Competence Center for Rare Oral and Dental Diseases, Marseille, France; ^14^ Centre Hospitalier Universitaire de Lyon, Competence Center for Rare Oral and Dental Diseases, Lyon, France; ^15^ Department of Stomatology (TD) and Department of Biology and Medical Genetics (MM) Charles University 2nd Faculty of Medicine and Motol University Hospital, Prague, Czechia; ^16^ Faculty of Dentistry, International University of Rabat, CReSS Centre de recherche en Sciences de la Santé, Rabat, Morocco; ^17^ Unité de génétique médicale et d’oncogénétique, CHU Hassan II, Fes, Morocco; ^18^ Faculty of Dentistry, Khon Kaen University, Khon Kaen, Thailand; ^19^ Instituto de Investigación en Ciencias Odontológicas, Facultad de Odontología, Universidad de Chile, Santiago, Chile

**Keywords:** enamel, amelogenesis imperfecta, genetics, rare diseases, NGS, next-generation sequencing

## Abstract

Amelogenesis imperfecta (AI) is a heterogeneous group of genetic rare diseases disrupting enamel development (Smith et al., Front Physiol, 2017a, 8, 333). The clinical enamel phenotypes can be described as hypoplastic, hypomineralized or hypomature and serve as a basis, together with the mode of inheritance, to Witkop’s classification (Witkop, J Oral Pathol, 1988, 17, 547–553). AI can be described in isolation or associated with others symptoms in syndromes. Its occurrence was estimated to range from 1/700 to 1/14,000. More than 70 genes have currently been identified as causative.

**Objectives:** We analyzed using next-generation sequencing (NGS) a heterogeneous cohort of AI patients in order to determine the molecular etiology of AI and to improve diagnosis and disease management.

**Methods:** Individuals presenting with so called “isolated” or syndromic AI were enrolled and examined at the Reference Centre for Rare Oral and Dental Diseases (O-Rares) using D4/phenodent protocol (www.phenodent.org). Families gave written informed consents for both phenotyping and molecular analysis and diagnosis using a dedicated NGS panel named GenoDENT. This panel explores currently simultaneously 567 genes. The study is registered under NCT01746121 and NCT02397824 (https://clinicaltrials.gov/).

**Results:** GenoDENT obtained a 60% diagnostic rate. We reported genetics results for 221 persons divided between 115 AI index cases and their 106 associated relatives from a total of 111 families. From this index cohort, 73% were diagnosed with non-syndromic amelogenesis imperfecta and 27% with syndromic amelogenesis imperfecta. Each individual was classified according to the AI phenotype. Type I hypoplastic AI represented 61 individuals (53%), Type II hypomature AI affected 31 individuals (27%), Type III hypomineralized AI was diagnosed in 18 individuals (16%) and Type IV hypoplastic-hypomature AI with taurodontism concerned 5 individuals (4%). We validated the genetic diagnosis, with class 4 (likely pathogenic) or class 5 (pathogenic) variants, for 81% of the cohort, and identified candidate variants (variant of uncertain significance or VUS) for 19% of index cases. Among the 151 sequenced variants, 47 are newly reported and classified as class 4 or 5. The most frequently discovered genotypes were associated with *MMP20* and *FAM83H* for isolated AI. *FAM20A* and *LTBP3* genes were the most frequent genes identified for syndromic AI. Patients negative to the panel were resolved with exome sequencing elucidating for example the gene involved ie *ACP4* or digenic inheritance.

**Conclusion:** NGS GenoDENT panel is a validated and cost-efficient technique offering new perspectives to understand underlying molecular mechanisms of AI. Discovering variants in genes involved in syndromic AI (*CNNM4, WDR72, FAM20A …* ) transformed patient overall care. Unravelling the genetic basis of AI sheds light on Witkop’s AI classification.

## Introduction

Enamel is the only mineralized structure of the body with an ectodermal origin. It has extraordinary mechanical and chemical properties. It is strongest and hardest material in the body and acts as an efficient barrier against environmental assaults whether mechanical, chemical, or physical. Enamel is incapable of regeneration or repair as ameloblasts, the specialized post-mitotic ectoderm-derived cells that produce the enamel matrix, disappear when the teeth erupt within the oral cavity. Normally, these ameloblasts produce proteins (enamelin, amelogenin, ameloblastin … ) in the secretory phase, mineralize this matrix and then mature it, in the maturation phase, by removing almost all the scaffold proteins *via* enzymes (KLK4, MMP20) to allow hydroxyapatite-crystal growth towards 96%–98% mineral content.

Amelogenesis imperfecta (AI) is a heterogeneous group of rare inherited diseases affecting amelogenesis, i.e. the enamel developmental process, in both primary and permanent dentitions and may be evident as an isolated trait or associated to other symptoms in syndromes. Amelogenesis imperfecta may manifest in different forms based on the phenotypic nature of the observed enamel defect, divided in three categories: hypoplastic (quantitative defect i.e. thinner enamel, pitted or striae enamel, enamel agenesis), hypomineralized (softer rough colored undermineralized enamel) or -hypomature (relatively hard but colored not translucent enamel). In 1988, Witkop (Witkop, 1988) proposed a revised classification of amelogenesis imperfecta considering the nature of the enamel defects as well the mode of inheritance (Table 1) and dividing AI into 4 classes (Type I hypoplastic, Type II hypomaturation, Type III hypocalcified, Type IV hypomaturation/hypoplastic with taurodontism). This classification was challenged by other authors cited in (Crawford et al., 2007) who proposed to add molecular data. More than 70 genes have been associated to “isolated” or “syndromic” AI. These genes encode a wide array of potential activities in amelogenesis, from enamel matrix proteins, to intracellular vesicle trafficking, to ameloblast attachment to the matrix or neighbor cells, to ion transport, to mineralization, to matrix-protein degradation. Critically, the syndromic manifestations of AI and other defects have proven to be an efficient strategy for elucidating the processes of odontogenesis providing better identification into new genes/proteins and their role in the physiopathology of enamel defects as well as the recognition of new clinical entities. Furthermore, some of these identified genes are involved in both syndromic and non-syndromic rare diseases.

**TABLE 1:** Witkop’s classification of amelogenesis imperfecta phenotypes and associated mode of inheritance (Witkop and Sauk, 1976; Witkop 1988) and current knowledge about corresponding associated genes.

TYPE	Class	Phenotype	Mode of inheritance	Phenotype OMIM number #	Genes
**I -HYPOPLASTIC**	**IA**	**HYPOPLASTIC, PITTED**	**AD**	**104530**	***LAMA3*, *LAMB3*, *LAMC2*, *COL7A1, COL17A1*, *ITGB6/4***
				**616221**	
	**IB**	**HYPOPLASTIC, LOCAL**	**AD**	**104500**	***ENAM***
	**IC**	**HYPOPLASTIC, LOCAL**	**AR**	**204650**	***ENAM***
				617297	*ACP4?*
	**ID**	**HYPOPLASTIC, SMOOTH**	**AD**	620104	*SP6?*
	**IE**	**HYPOPLASTIC, SMOOTH**	**XLD**	**301200**	***AMELX*, *ARHGAP6,*** *HCCS?*
	**IF**	**HYPOPLASTIC, ROUGH**	**AD**	**616270**	***AMBN***
	**IG**	**ENAMEL AGENESIS**	**AR**	**204690**	***FAM20A***
	IH		AR	**616221**	***ITGB6***
	IJ		AR	**617297**	***ACP4 = ACPT***
	IK		AD	**620104**	***SP6***
**II -HYPOMATURATION**	**IIA**	**HYPOMATURATION, PIGMENTED**	**AR**	**204700**	***KLK4, MMP20, WDR72, ODAPH = C4orf26, SLC24A4, GPR68***
				**612529**	
				**613211**	
				**614832**	
				**615887**	
				**617217**	
	**IIB**	**HYPOMATURATION**	**XLR**	**301200**	*AMELX?*
	**IIC**	**SNOW CAPPED TEETH**	**XL**	**301200**	***AMELX—ARHGAP6***
	**IID**	**SNOW CAPPED TEETH**	**AD**	**?**	**?**
**III -HYPOCALCIFIED**	**IIIA**		**AD**	**130900**	***FAM83H*** (***AD***) ***, AMTN*** (***AD***)
				**617607**	
	**IIIB**		**AR**	**618386**	***RELT*** (***AR***) (*IIIC?*)
**IV -HYPOMATURATION-HYPOPLASTIC WITH TAURODONTISM**	**IVA**	**HYPOMATURATION-HYPOPLASTIC WITH TAURODONTISM**	**AD**	**104510**	***DLX3***
	**IVB**	**HYPOPLASTIC-HYPOMATURATION**	**AD**	**104510**	***DLX3***
		**WITH TAURODONTISM**			
V -SYNDROMIC AI					*TSC1, FAM20A, DLX3, LTBP3, CNNM4, ROGDI, SLC13A5, SLC10A7, GALNS, AIRE, ORAI1, STIM1, PORCN, PEX1, PEX6, PEX26, CLDN16, CLDN19, FAM20C, SLC4A4, ATP6V1A …*

Bold values correspond to the original Witkop’s classification.

In this paper, we propose to revise Witkop’s classification in the light of recent progress in genetics and genomics. The next-generation sequencing panel GenoDENT (Prasad et al., 2016a; Rey et al., 2019) and exome sequencing (WES) (Laugel-Haushalter et al., 2019) have improved understanding and recognition of AI and associated syndromes. We report in this paper individuals with pathogenic variants in known genes involved in AI but also individuals with variants in new candidate genes and individuals presenting variants of uncertain significance (VUS) in known genes. It is our hope that the clinical pictures provided with the proposed classification will assist clinicians in AI recognition. By combining clinical and genetic diagnosis we expect to reveal previously undiscovered rare diseases with a broader clinical spectrum thus improving our diagnoses and management. This strategy would emphasize the role of dentists in the new era of personalized medicine.

## Material and methods

### Individual’s phenotypes

Individuals were enrolled and examined in the Reference Center (CRMR) for rare oral and dental diseases or in one of the 16 affiliated Competence Centers (CCMR) of the French O-Rares network, Filière TETECOU) or by their treating practitioners from France and other countries (ex. the ERN CRANIO). They were recruited between 2009 and 2021. When possible, parents and relatives were also included in the study.

Oral phenotype was documented using the D[4]/phenodent registry protocol, a Diagnosing Dental Defects Database [see www.phenodent.org, for assessment form], which is approved by CNIL (French National commission for informatics and liberty, number 908416). This clinical study is registered at https://clinicaltrials.gov: NCT01746121 and NCT02397824, and with the MESR (French Ministry of Higher Education and Research) Bioethics Commission as a biological collection “Orodental Manifestations of Rare Diseases” DC-2012-1,677 within DC-2012-1,002 and was acknowledged by the CPP (person protection committee) Est IV 11 December 2012.

The individuals presenting AI and the non-affected family members gave written informed consents in accordance with the Declaration of Helsinki, both for the D[4]/phenodent registry and for genetic analyses performed on salivary samples (Oragene^®^ DNA OG-250, OG_650 commercial kits (DNA Genotek Inc., Ottawa, Ont, Canada). Genomic DNA was extracted according to the manufacturer’s protocol included in the biological collection.

The terminology used to describe dental and enamel abnormalities has been detailed in (de La Dure-Molla et al., 2019).

Individuals’ biological samples were sent to the reference center of Strasbourg, France for genetical analysis.

### Individual’s genotypes

#### Next-generation sequencing panel genodent

The GenoDENT panel has been evolving through times from its first published version (Prasad et al., 2016a). The last updated version 6.0 explores 567 genes (Supplementary Table S1).

The GenoDENT panel interrogates two categories of genes: a diagnostic panel (248 genes known as responsible for rare diseases with orodental expression in human) and a discovery panel (319 candidate genes reported as being involved in tooth development or orodental anomalies in animal models for example).

Probe design was performed on the Agilent SureDesign portal (https://erray.chem.agilent.com/suredesign, Agilent, United States) in order to capture, by complementarity, the exonic sequence as well as 25 bases of their flanking intronic sequences. Libraries were prepared with the Agilent SureSelect QXT protocol and sequenced on a NextSeq 550 (Illumina, San Diego, United States). GenoDENT is implemented in a diagnostic setting and its results are directly available for the individual medical file and genetic counselling. Variants are classified according to the American College of Medical Genetics (ACMG) classification (Richards et al., 2015; Harrison et al., 2019). Upon identification of variants of class 4 (probably pathogenic) or 5 (pathogenic), extended familial segregation is performed *via* Sanger sequencing; a detailed report is written and sent to the geneticist. A variant of uncertain significance (VUS) or class 3 should not be used in clinical decision-making. Class 2 are likely benign polymorphisms.

#### Whole exome sequencing

Trio whole Exome Sequencing (WES) was performed on trio for individuals 7.10, 7.11, 9.1, 9.2, 9.3, 17.1, 17.2, 17.3, 17.4, 18.3, 18.8, 18.10, and 24.2 by Integragen (Evry, France, 2014). Exons of DNA samples were captured using in-solution enrichment methodology (SureSelect Human All Exon Kits, Agilent, Massy, France) with the company’s biotinylated oligonucleotide probe library (Agilent Human All Exon v5+UTR 75 Mb Kit) and sequenced with an Illumina HISEQ 2000 (Illumina, San Diego, United States) as paired-end 75 bp reads, resulting in an average coverage of 80X.

#### Bioinformatics analysis

STARK (Stellar Tools from raw sequencing data Analysis to variant RanKing) is a bioinformatics pipeline based on the GATK recommendations used to process the NGS data (DePristo et al., 2011). Annotation and ranking of SNV/indel were performed by VaRank (Geoffroy et al., 2015) in combination with the Alamut Batch software (Interactive Biosoftware, Rouen, France). Variant effect on the nearest splice site was predicted using MaxEntScan (Yeo and Burge, 2004), NNSplice (Reese et al., 1997) and Splice Site Finder (Shapiro and Senapathy, 1987).Very stringent criteria were applied to filter out non-pathogenic variants: 1) variants represented with an allele frequency of more than 1% in public variation databases including the 1,000 Genomes (The 1000 Genomes Project Consortium et al., 2015), the GnomAD database (Exome Aggregation Consortium et al., 2016) or our internal exome database, 2) variants in 5′ or 3′ UTR, 3) variants with intronic locations and no prediction of local splice effect, and 4) synonymous variants without pathogenic prediction of local splice effect. Annotations of structural variations (SV) were performed by AnnotSV (Geoffroy et al., 2018).

#### Sanger sequencing and segregation

Primers’ design was done using the Amplifix v1.5.4 software. Primers were then ordered from Eurofin MWG (Supplementary Table S2). The amplicons’ sizes were checked by electrophoresis on the Caliper LabChip GX (Life science). After enzymatic purification with the Illustra™ Exoprostar™ kit (Sigma Aldrich) to remove dNTPs and salts, the PCR product was used to perform a sequence reaction with the BigDye™ Terminator v1.1 Cycle Sequencing Kit (Applied Biosystems, Thermofisher Scientific). The BigDye Xterminator™ Purification Kit (Applied Biosystems by Thermofisher Scientific) was used to purified product. This purified product was then loaded on the 3,500 Series Genetic Analyzers (Applied Biosystems, Thermofisher Scientific) sequencer. Final sequence data were analyzed with SEQUENCE Pilot (JSI medical systems).

## Results

We report genetic results for 221 individuals divided between 115 amelogenesis imperfecta (AI) index cases (71 females and 44 males) and 106 relatives from 111 families. Among index cases, 73% were diagnosed with non-syndromic AI and 27% with syndromic AI. Clinical phenotype was assessed according to Witkop’s classification and repartition for index’s phenotype is: Type I hypoplastic AI (61 individuals, 53%), Type II hypomature AI (31 individuals, 27%), Type III hypomineralized AI (18 individuals 16%) and Type IV hypoplastic-hypomature with taurodontism AI (5 individuals, 4%).

Genetic variants were analyzed using NGS GenoDENT panel and following the ACMG recommendations (Richards et al., 2015; Harrison et al., 2019). Pathogenic variant (class 5) or likely pathogenic variant (class 4) were identified for 81% of the reported index individuals. Non-conclusive variants of uncertain significance (VUS) (class 3) represented the remaining 19%.

Among the 151 sequenced variants identified for indexes, 47 are newly reported and classified as class 4 or 5 (Table 2; Supplementary Table S3).

**TABLE 2:** Variations found in individuals presenting with syndromic amelogenesis imperfecta.

**Patient number**	**Diagnosis/AI**	**Gene**	**Variant and location**	**Zygosity**	**Mode of inheritance**	**Rank**	**Effect of the mutation**	**Consistent with the known disease phenotype**	**Family segregation**	**Status**
**17.1 (female)**	Hypoplastic, short stature	***LTBP3 Chr11*** (***GRCh37***) ***: NM_001130144.3***	c.421C>T; p.(Gln141*) Exon 2 Huckert M et al. (2015)	compound heterozygous	AR	4	non-sense	Yes	S(A,C)	exome
			c.1531 + 1G>T; p.? Intron 8 Huckert M et al. (2015)		AR	4	splice		S(A,C)	
**17.2 (female)**	Hypoplastic, short stature	***LTBP3 Chr11*** (***GRCh37***) ***: NM_001130144.3***	c.2071_2084del; p.(Tyr691Leufs*95) Exon 14 Huckert M et al. (2015)	homozygous	AR	4	frameshift	Yes	S(A,C) S(U,R) MoFa(U,C)	exome
**17.3 (male)**	Hypoplastic, short stature	***LTBP3 Chr11*** (***GRCh37***) ***: NM_001130144.3***	c.2216del; p.(Gly739Alafs*7) Exon 15 Huckert M et al. (2015)	homozygous	AR	4	frameshift	Yes	MoFa(U,C)	exome
**17.4 (male)**	Hypoplastic, short stature	***LTBP3 Chr11*** (***GRCh37***) ***: NM_001130144.3***	c.2356del; p.(Val786Trpfs*82) Exon 17 Huckert M et al. (2015)	homozygous	AR	4	frameshift	Yes	MoFa(U,C) 3S(A,C)	exome
**17.5 (female)**	Hypoplastic, short stature	***LTBP3 Chr11*** (***GRCh37***) ***: NM_001130144.3***	c.3087del; p.(Asn1030Thrfs*47) Exon 22	homozygous	AR	4	frameshift	Yes	MoFa(U,C)	panel
**17.6 (female)**	Hypoplastic, short stature	***LTBP3 Chr11*** (***GRCh37***) ***: NM_001130144.3***	c.3629-2A>G; p.? Intron 26	homozygous	AR	4	splice	Yes	MoFa(U,C)	panel
**Patient number**	**Diagnosis/AI**	**Gene**	**Variant and location**	**Zygosity**		**Rank**	**Effect of the mutation**	**Consistent with the known disease phenotype**	**Family segregation**	**Status**
**18.1 (female)**	Hypoplastic AI, nephrocalcinosis	***FAM20A** Chr17(GRCh37): NM_017565.4*	c.34_35del; p.(Leu12Alafs*67) Exon 1 Cho et al. (2012)	compound heterozygous	AR	5	frameshift	Yes	S(A,C)	panel
			c.610del; p.(Ala204Profs*12) Exon 3		AR	4	frameshift		S(A,C)	
**18.2 (male)**	Hypoplastic AI, nephrocalcinosis	***FAM20A** Chr17(GRCh37): NM_017565.4*	c.53_54delinsAG; p.(Leu18Arg) Exon 1	putative compound heterozygous	AR	3	missense	Yes	NA	panel
			c.976_978del; p.(Glu326del) Exon 7		AR	3	deletion		NA	
**18.3 (male)**	Hypoplastic AI, nephrocalcinosis	***FAM20A** Chr17(GRCh37): NM_017565.4*	c.217C>T; p.(Arg73*) Exon 1 Jaureguiberry et al. (2012)	compound heterozygous	AR	4	non-sense	Yes	Fa(U,C) S(A,C)	exome
			c.727C>T; p.(Arg243*) Exon 5 Jaureguiberry et al. (2012)		AR	4	non-sense		Mo(U,C) S(A,C)	
**18.4 (female)**	Hypoplastic AI, nephrocalcinosis	***FAM20A** Chr17(GRCh37): NM_017565.4*	c.406C>T; p.(Arg136*) Exon 2 O'Sullivan et al. (2011)	homozygous	AR	5	non-sense	Yes	NA	panel
**18.5 (male)**	Hypoplastic AI, nephrocalcinosis	***FAM20A** Chr17(GRCh37): NM_017565.4*	c.915_918del; p.(Phe305Leufs*76) Exon 6 Jaureguiberry et al. (2012)	compound heterozygous	AR	4	frameshift	Yes	Fa(U,C)	panel
			c.928 + 2T>C; p.? Intron 6		AR	4	splice		Mo(U,C)	
**18.6 (male)**	Hypoplastic AI, nephrocalcinosis	***FAM20A** Chr17(GRCh37): NM_017565.4*	c.915_918del; p.(Phe305Leufs*76) Exon 6 Jaureguiberry et al. (2012)	compound heterozygous	AR	4	frameshift	Yes	S(A,C) Mo(U,C)	panel
		***FAM20A** Chr17(GRCh37): NM_017565.4*	c.1301 + 5G>A; p.? Intron 9		AR	3	splice	Yes	S(A,C) Fa(U,C)	panel
**18.7 (female)**	Hypoplastic AI, nephrocalcinosis	***FAM20A** Chr17(GRCh37): NM_017565.4*	c.1106_1107delAG; p.(Glu369Glyfs*10) Exon 7 Prasad et al. (2016a)	homozygous	AR	4	frameshift	Yes	NA	panel
**18.8 (female)**	Hypoplastic AI, nephrocalcinosis	***FAM20A** Chr17(GRCh37): NM_017565.4*	c.1361 + 1G>A; p.? Intron 10	homozygous	AR	4	splice	Yes	NA	exome
**18.9 (female)**	Hypoplastic AI, nephrocalcinosis	***FAM20A** Chr17(GRCh37): NM_017565.4*	c.1369A>T; p.(Lys457*) Exon 11 Jaureguiberry et al. (2012)	homozygous	AR	4	non-sense	Yes	S(A,C)	panel
**18.10 (female)**	Hypoplastic AI, nephrocalcinosis	***FAM20A** Chr17(GRCh37): NM_017565.4*	c.1369A>T; p.(Lys457*) Exon 11 Jaureguiberry et al. (2012)	homozygous	AR	4	non-sense	Yes	MoFa(U,C)	exome
**Patient number**	**Diagnosis/AI**	**Gene**	**Variant and location**	**Zygosity**		**Rank**	**Effect of the mutation**	**Consistent with the known disease phenotype**	**Family segregation**	**Status**
**19.1 (male)**	Hypoplastic, pits, mucopolysaccharidosis IV	**GALNS Chr16(GRCh37):NM_000512.5**	c.121-31T>C; p.? Intron 1 Prasad et al. (2016b)	compound heterozygous	AR	3	splice	Yes	Mo(U,C)	panel
			c.953C>G; p.(Thr312Ser) Exon 9 Yamada et al. (1998)		AR	5	missense		Fa(U,C)	
**19.2 (female)**	Hypoplastic, pits, mucopolysaccharidosis IV	***GALNS Chr16*** (***GRCh37***) ***: NM_000512.5***	c.1156C>T; p.(Arg386Cys) Exon 11 Ogawa et al. (1995)	heterozygous	AR	5	missense	Yes	Mo(U,R) Fa(U,R)	panel
			c.1558T>C; p.(Trp520Arg) Exon 14 Zanetti et al. (2021)	heterozygous	AR	2	missense		M(U,Chom)	
**Patient number**	**Diagnosis/AI**	**Gene**	**Variant and location**	**Zygosity**		**Rank**	**Effect of the mutation**	**Consistent with the known disease phenotype**	**Family segregation**	**Status**
**20.1 (female)**	Hypoplastic, banding pattern, Lyonisation, Microphthalmia with linear skin defects (MLS) syndrome	***AMELX-ARHGAP6 ChrX*** (***GRCh37***) ***:g.125958-12725766del***	ChrX(GRCh37):g.125958-12725766del Many genes including AMELX	heterozygous	XL	4	deletion	Yes	NA	panel
**Patient number**	**Diagnosis/AI**	**Gene**	**Variant and location**	**Zygosity**		**Rank**	**Effect of the mutation**	**Consistent with the known disease phenotype**	**Family segregation**	**Status**
**21.1 (male)**	Hypoplastic, Smith Magenis syndrome	***RAI1***	arr[GRCh37] 17p11.2(17280004_20239827)x1 Many genes including RAI1	heterozygous	AD	4	deletion	-	NA	panel
**Patient number**	**Diagnosis/AI**	**Gene**	**Variant and location**	**Zygosity**		**Rank**	**Effect of the mutation**	**Consistent with the known disease phenotype**	**Family segregation**	**Status**
**22.1 (male)**	Hypoplastic, Loeys-Dietz syndrome	***TGFBR2* Chr4(GRCh37): NM_003242.6**	c.1561T>C; p.(Trp521Arg) Exon 7 Mátyás et al. (2006)	heterozygous	AD	5	missense	Yes	Fa(U,R) Mo(A,NA)	panel
**Patient number**	**Diagnosis/AI**	**Gene**	**Variant and location**	**Zygosity**		**Rank**	**Effect of the mutation**	**Consistent with the known disease phenotype**	**Family segregation**	**Status**
**23.1 (female)**	Hypoplastic, Kohlschutter-Tonz like syndrome	***SLC13A5* Chr17(GRCh37): NM_177550.5**	c.203C>A; p.(Pro68Gln) Exon 2 Schossig et al. (2017)	compound heterozygous	AR	4	missense	Yes	S(A,C)	panel
			c.434C>A; p.(Thr145Lys) Exon 4 Schossig et al. (2017)		AR	4	missense		S(A,C)	
**Patient number**	**Diagnosis/AI**	**Gene**	**Variant and location**	**Zygosity**		**Rank**	**Effect of the mutation**	**Consistent with the known disease phenotype**	**Family segregation**	**Status**
**24.1 (female)**	Hypomature, Kohlschutter Tonz syndrome	***ROGDI Chr16*** (***GRCh37***) ***: NM_024589.3***	c.46 + 37_46-30del; p.? Intron 1 Tucci et al. (2013)	compound heterozygous	AR	4	deletion	Yes	Mo(U,C)	Insbruck
			c.507del; p.(Glu170Argfs*72) Exon 7 Tucci et al. (2013)		AR	4	deletion		Fa(U,C)	
**24.2 (female)**	Hypomature, Kohlschutter Tonz syndrome	***ROGDI Chr16*** (***GRCh37***) ***: NM_024589.3***	c.117 + 1G>T; p.? Intron 2 Huckert et al. (2014)	homozygous	AR	4	splice	Yes	NA	panel
**24.3 (female)**	Hypomature, Kohlschutter Tonz syndrome	***ROGDI Chr16*** (***GRCh37***) ***: NM_024589.2***	c.366dup; p.(Ala123Serfs*19) Exon 6 Tucci et al. (2013)	compound heterozygous	AR	4	frameshift	Yes	Mo(U,C)	panel
			c.402C>G; p.(Tyr134*) Exon 6 Aswath al. (2018)		AR	4	non-sense		Fa(U,C)	
**Patient number**	**Diagnosis/AI**	**Gene**	**Variant and location**	**Zygosity**		**Rank**	**Effect of the mutation**	**Consistent with the known disease phenotype**	**Family segregation**	**Status**
**25.1 (male)**	Hypomature/Hypomineralized, short stature, intra-uterine growth retardation, skeletal dysplasia, submucosal cleft palate	***SLC10A7* Chr4(GRCh37): NM_001300842.3**	c.269T>G; p.(Leu90Arg) Exon 3	homozygous	AR	3	missense	Yes	MoFa(U,C)	panel
**25.2 (female)**	Hypomature/Hypomineralized, short stature, intra-uterine growth retardation, skeletal dysplasia	***SLC10A7* Chr4(GRCh37): NM_001300842.3**	c.908C>T; p.(Pro303Leu) Exon 11 Laugel-Haushalter et al. (2019)	homozygous	AR	4	missense	Yes	MoFa3S(U,C)	exome
**Patient number**	**Diagnosis/AI**	**Gene**	**Variant and location**	**Zygosity**		**Rank**	**Effect of the mutation**	**Consistent with the known disease phenotype**	**Family segregation**	**Status**
**26.1 (female)**	Hypomineralized, Jalili syndrome	***CNNM4 Chr2*** (***GRCh37***) ***: NM_020184.4***	c.586T>C; p.(Ser196Pro) Exon 1 Parry et al. (2009)	homozygous	AR	4	missense	Yes	S(A,C) Mo(U,C)	panel
**26.2 (male)**	Hypomineralized, Jalili syndrome	***CNNM4 Chr2*** (***GRCh37***) ***: NM_020184.4***	c.1495G>A; p.(Val499Met) Exon 2 Prasad MK et al. (2016b)	homozygous	AR	4	missense	Yes	NA	panel
**Patient number**	**Diagnosis/AI**	**Gene**	**Variant and location**	**Zygosity**		**Rank**	**Effect of the mutation**	**Consistent with the known disease phenotype**	**Family segregation**	**Status**
**27.1 (female)**	Hypoplastic, Trichodentoosseus syndrome	***DLX3 Chr17*** (***GRCh37***) ***: NM_005220.3***	c.561_562del; p.(Tyr188Glnfs*13) Exon 3 Dong et al. (2005)	heterozygous	AD	4	frameshift	Yes	MoS(A,C)	panel
**27.2 (male)**	Hypoplastic, Trichodentoosseus syndrome	***DLX3 Chr17*** (***GRCh37***) ***: NM_005220.3***	c.561_562del; p.(Tyr188Glnfs*13) Exon 3 Dong et al. (2005)	heterozygous	AD	4	frameshift	Yes	Fa(A,C)	panel

Variations found in 11 different genes in 31 individuals presenting with syndromic amelogenesis imperfecta. Forty-two variants were found, 7 variants are of uncertain significance.

Variants known before the panel implementation are reported in grey, variants previously reported by the team are represented in salmon, variants published thanks to the panel are represented in blue or green, variants reported for the first time are highlighted in green. Familial segregation is also reported when available and reported in this format: Family member code (Phenotype code, Genotype code). Fa: father; Mo: mother; S: sibling; D: daughter; So: son; Co: cousin; A: affected; U: unaffected; NA: not available; C: carrier; R: reference genotype.

**FIGURE 1:**
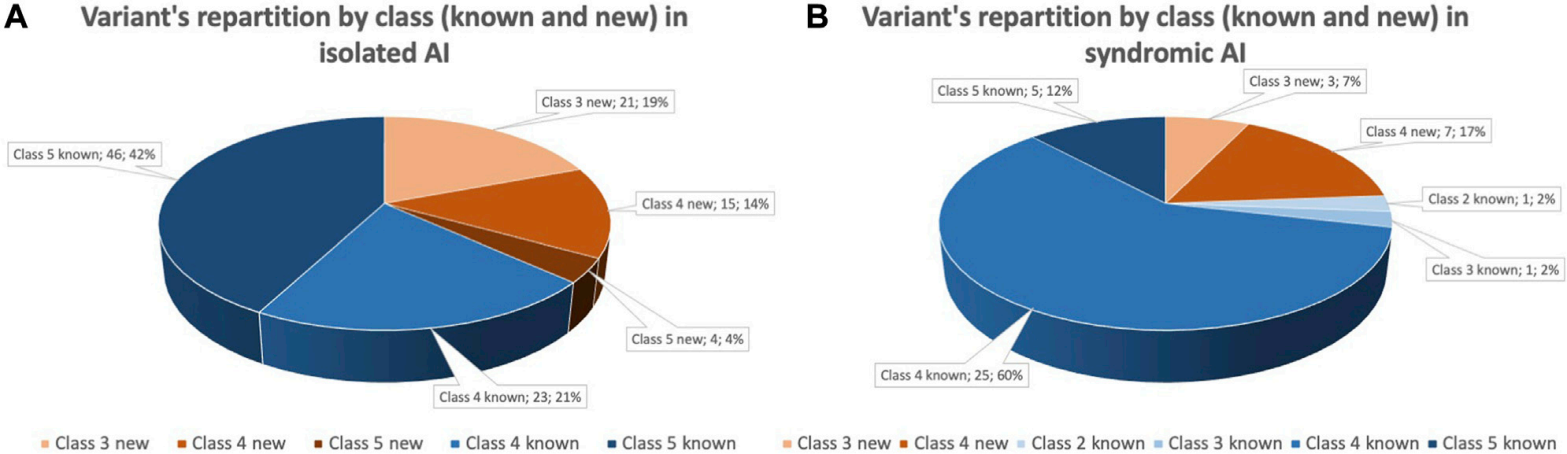
Identified variants within the cohort grouped by pathogenic class and novelty. Variant were classified following ACMG recommendations. **(A)** Variant’s repartition by pathogenic class and novelty for individual affected by isolated AI. Forty variants (37%) are newly identified variants (orange gradient), 69 (63%) variants are already reported in literature (blue gradient). Among variants reported, 50 (46%) are pathogenic (class 5), 38 (35%) are likely pathogenic (class 4) and 21 (19%) are of uncertain significance. **(B)**. Variant’s repartition by pathogenic class and novelty for individual affected by syndromic AI. Seven variants (24%) are newly identified variants (orange gradient), 32 variants (76%) are already reported in literature (blue gradient). Among variants reported, 5 (12%) are pathogenic (class 5), 32 (77%) are likely pathogenic (class 4), 4 (9%) are of uncertain significance (class 3) and 1 (2%) is probably begin (class 2).

More specifically for isolated AI individuals, 109 variants are described with 40 newly reported (four class 5, 15 class 4 and 21 VUS) and 69 already reported (46 class 5 and 23 class 4). For syndromic AI, 42 variants are listed with ten newly reported (7 class 4, 3 VUS) and 32 already reported (5 class 5, 25 class 4, 1 class 2 and 1 VUS) (Figure 1). For some individuals two variants have been reported in the case of an autosomal recessive (AR) disorder and heterozygous compound variants (23 isolated AI individuals and 10 syndromic). VUS have been identified in different situations: nine times as solo heterozygous variant, two times as homozygous variant, four times as two heterozygous VUS and seven times as heterozygous associated with a pathogenic variant.

**FIGURE 2:**
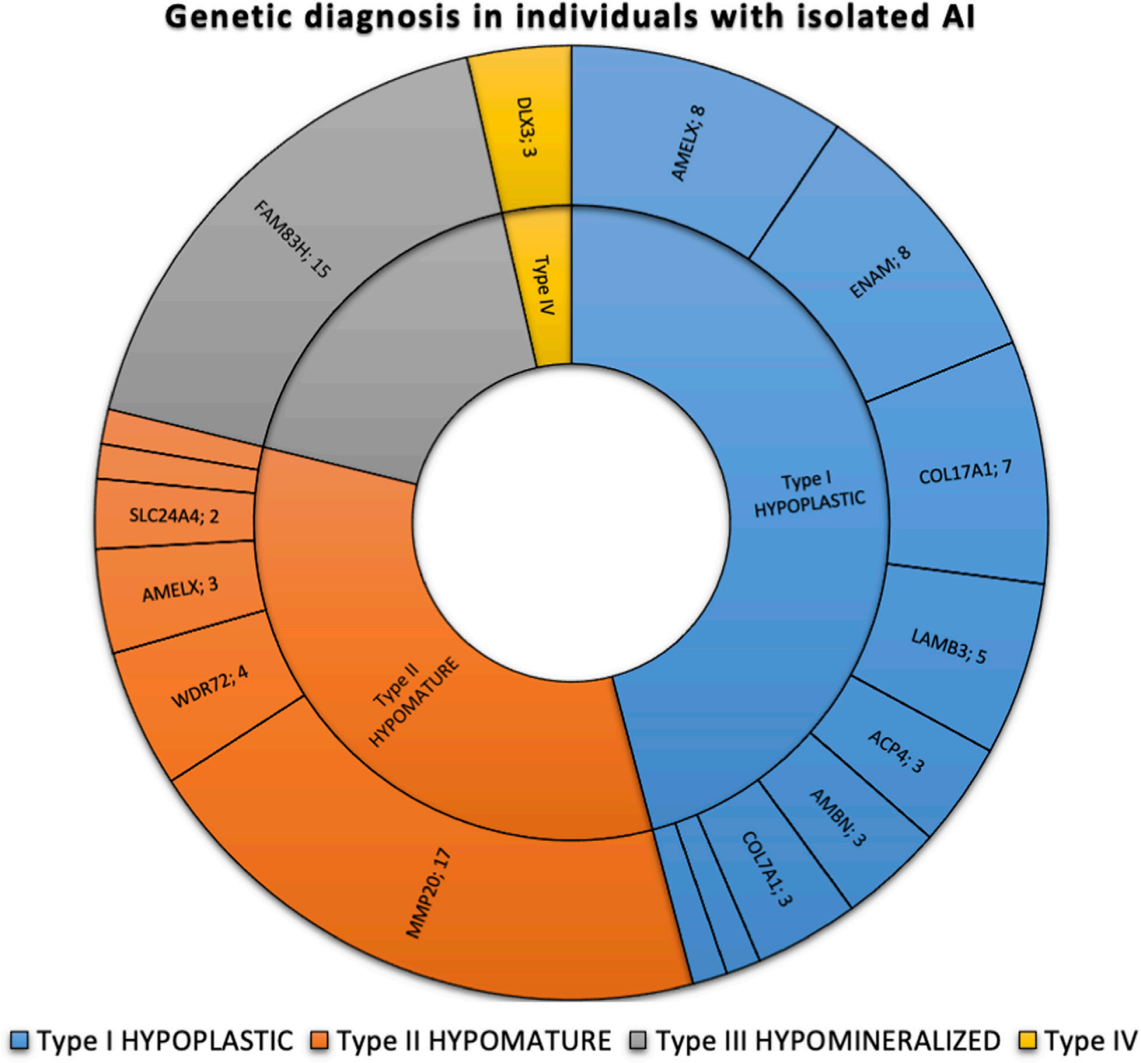
Phenotypic and genetic diagnosis in 86 individuals with isolated AI. Number of patients per main type of AI and per gene. Type I hypoplastic AI represents 39 individuals (45.3%) in blue associated with 9 different genes (*AMELX, ENAM, COL17A1, LAMB3, ACP4, AMBN, COL7A1, LAMA3, LAMC2*). Type II hypomature AI represents 28 individuals (32.6%) in orange associated with 6 different genes (*MMP20, WDR72, AMELX, SLC24A4, KLK4*). Type III hypomineralized AI represents 16 individuals (18.6%) in grey associated with 2 different genes (*FAM83H, WDR72*). Type IV hypoplastic-hypomature with taurodontism AI represents 3 individuals (3.5%) in yellow in 1 gene (*DLX3*).

**FIGURE 3:**
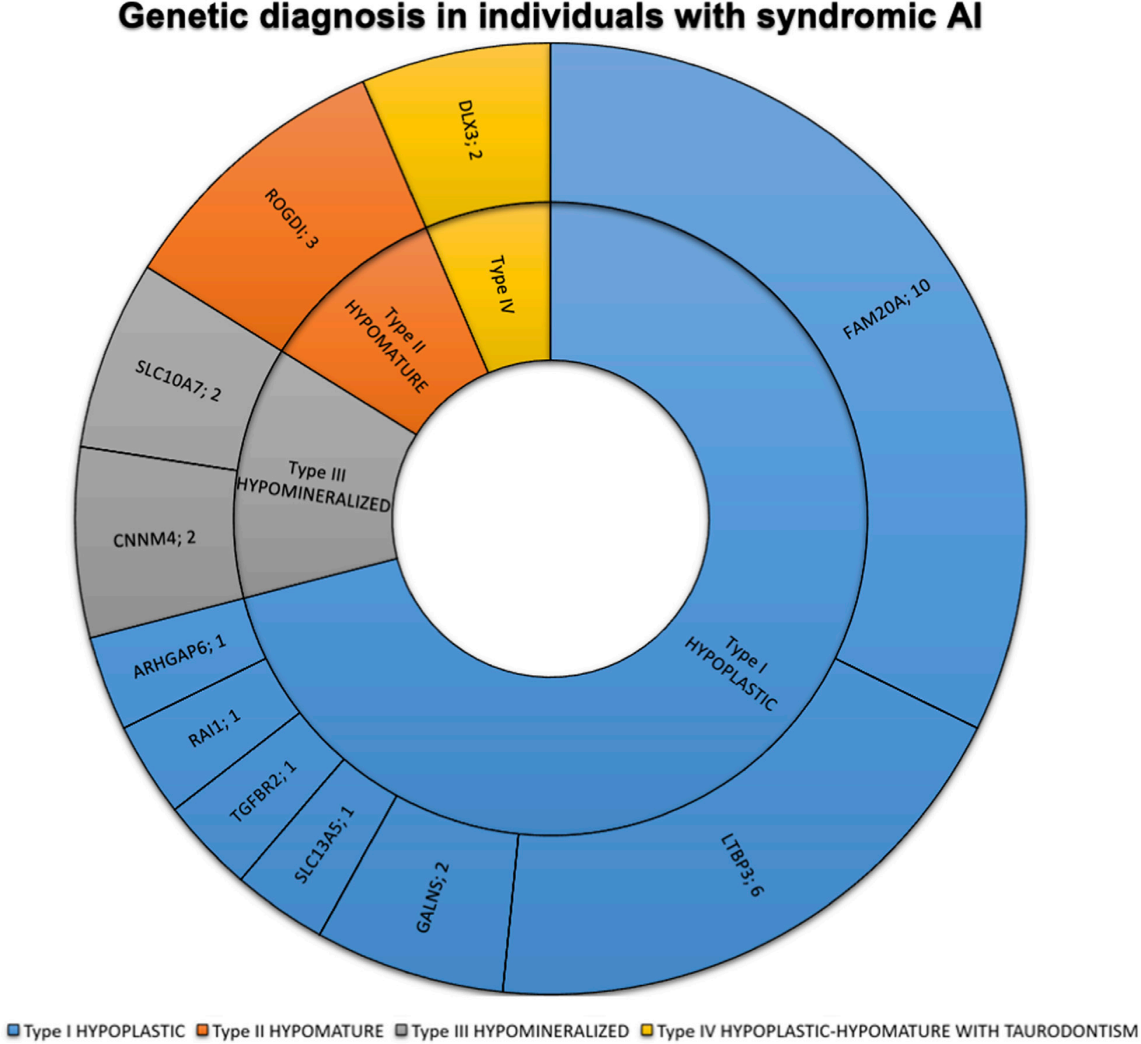
Phenotypic and genetic diagnosis in 31 individuals with syndromic AI. Number of patients per main type of AI and per gene. Type I hypoplastic AI represents 22 individuals (71%) in blue associated with 7 different genes (*FAM20A, LTBP3, GALNS, ARHGAP6, RAI1, SLC13A5, TGFBR2*). Type II hypomature AI represents 3 individuals (9.7%) in orange associated with 1 gene (*ROGDI*). Type III hypomineralized AI represents 4 individuals (12.9%) in grey associated with 2 different genes (*CNNM4, SLC10A7*). Type IV hypoplastic-hypomature with taurodontism AI represents 2 individuals (6.5%) in yellow in 1 gene (*DLX3*).

The most frequently discovered genotypes were associated with *MMP20*, *FAM83H* and then *AMELX* and *ENAM* variants for isolated AI (Figure 2). We didn’t find any variant in *GPR68*, *STIM1*, *RELT*, *ITGB6/4*, *AMTN* and *SP6 genes*. In individuals presenting syndromic AI we reported variants in *LTBP3*, *FAM20A* and *GALNS*, *SLC13A5*, *DLX3*, *RAI1*, *TGFBR2*, *CNNM4*, *SLC10A7*, *ROGDI* (Figure 3) but didn’t find any variant in *TP63*, *TSC1-2*, *AIRE*, *CLDN16*, *CLDN19*, *ORAI1*, *STIM1*, *RELT*, *PEX26*, *PEX1*, *PEX6*, *PORCN* and *MSX2*.

Familial segregation, with Sanger sequencing, of variants previously identified in index cases was performed on 106 individuals. Among them, 33 affected individuals were carrier of the familial variant, 35 non-affected individuals were carrier of one of the two familial variants in the context of a recessive pathology, 31 non-affected individuals did not carry the familial variant. Phenotype/genotype correlation was not conclusive for 7 individuals mostly because sufficient phenotypic information was not available.

Through this deliberate yet targeted strategy, we were able to identify pathogenic variants in known genes involved in AI as expected. In addition, we identified variants in candidate genes previously unreported in AI as well as individuals presenting novel VUS in known genes. As it is nearly impossible in the field of rare disease to create an exhaustive repertoire of pathological variants, we present the findings of this study following (Witkop, 1988) classification, gene by gene linking phenotypic clinical description with the associated genotype.

Additionally, working with this panel of variants, with annotated gene functions, and with the genotype/phenotype associations described in the cited literature, we aim to refine the clinical classification on the basis of Witkop’s classification to integrate the current understanding of AI in the context of genetic data, with an initial segregation of phenotypes as “isolated” or “syndromic”. Listed below are the proposed categories and sub-categories under this novel “GenoDENT” classification.

“ISOLATED” AMELOGENESIS IMPERFECTA

AI can occur with or without associated syndromic conditions. These following proposed classes of AI expand on Witkop’s classification to describe non-syndromic AI diseases at a genetic level.

### Type I—Hypoplastic

Hypoplastic AI describes quantitative enamel defects such as localized hypoplasia, generalized hypoplasia, enamel pits, enamel striae, groove defects, thin but mineralized enamel, or in extreme cases, the complete absence of enamel. Anomalies observed in hypoplastic AI, result from failure during the enamel matrix secretory stage (Wang et al., 2015). Four forms of hypoplastic AI—the pitted, local, smooth and rough forms - are autosomal dominant (type IA, IB, ID and IF), three are autosomal recessive (type IC, IG and IJ), and one is X-linked (type IE) (Witkop and Sauk, 1976).

### Type IA—Hypoplastic, pitted, autosomal dominant *COL17A1, COL7A1, LAMA3, LAMB3* (#104530)*, LAMC2, ITGB6* (#616221)

Enamel may display pits on the labial or buccal surfaces often arranged in rows and columns. Often these pits are obvious as they are colored by extrinsic stains that can be removed by professional cleaning.

The genes implicated in this subtype of AI encode proteins of the extracellular matrix, important for the attachment of the ameloblast cells to their matrix, structural component of hemidesmosomes *COL17A1*, anchoring fibril to the basement membrane *COL7A1*, laminin constituting chains *LAMA3, LAMB3, LAMC2*, integrins adhesion receptors that function in signaling from the extracellular matrix to the cell *ITGB6* (a receptor for the laminins). *COL7A1* gene encodes the alpha-1 chain of type VII collagen (Burgeson et al., 1985). *COL17A1* encodes the homotrimer type XVII collagen (COL17). *LAMA3, LAMB3, LAMC2* genes encode laminin α3, laminin β3, and laminin γ2, respectively, forming together the heterotrimer laminin-332 (LM-332).

Both LM-332 and COL17 are crucial in ameloblast differentiation and enamel formation, mutations of which result in enamel defects consisting of hypoplasia, pitting, roughness, thinning or furrowing of enamel (Yuen et al., 2012).

These same genes, under an autosomal recessive inheritance transmission are responsible for various forms of epidermolysis bullosa (EB: Non-Herlitz junctional epidermolysis bullosa (nH-JEB) *COL17A1*; recessive dystrophic epidermolysis bullosa (RDEB) *COL7A1;* junctional EB (JEB) LAMA3, *LAMB3, LAMC2*) (Masunaga, 2006). In EB, the phenotype synopsis includes nails dystrophy, skin hyperhidrosis and hyperkeratosis, blistering of skin and mucosa, eye defects, hair anomalies with alopecia or loss of eyelashes and an abnormal dentition with extensive enamel hypoplasia, focal pitting, and discoloration.

#### COL17A1

**FIGURE 4:**
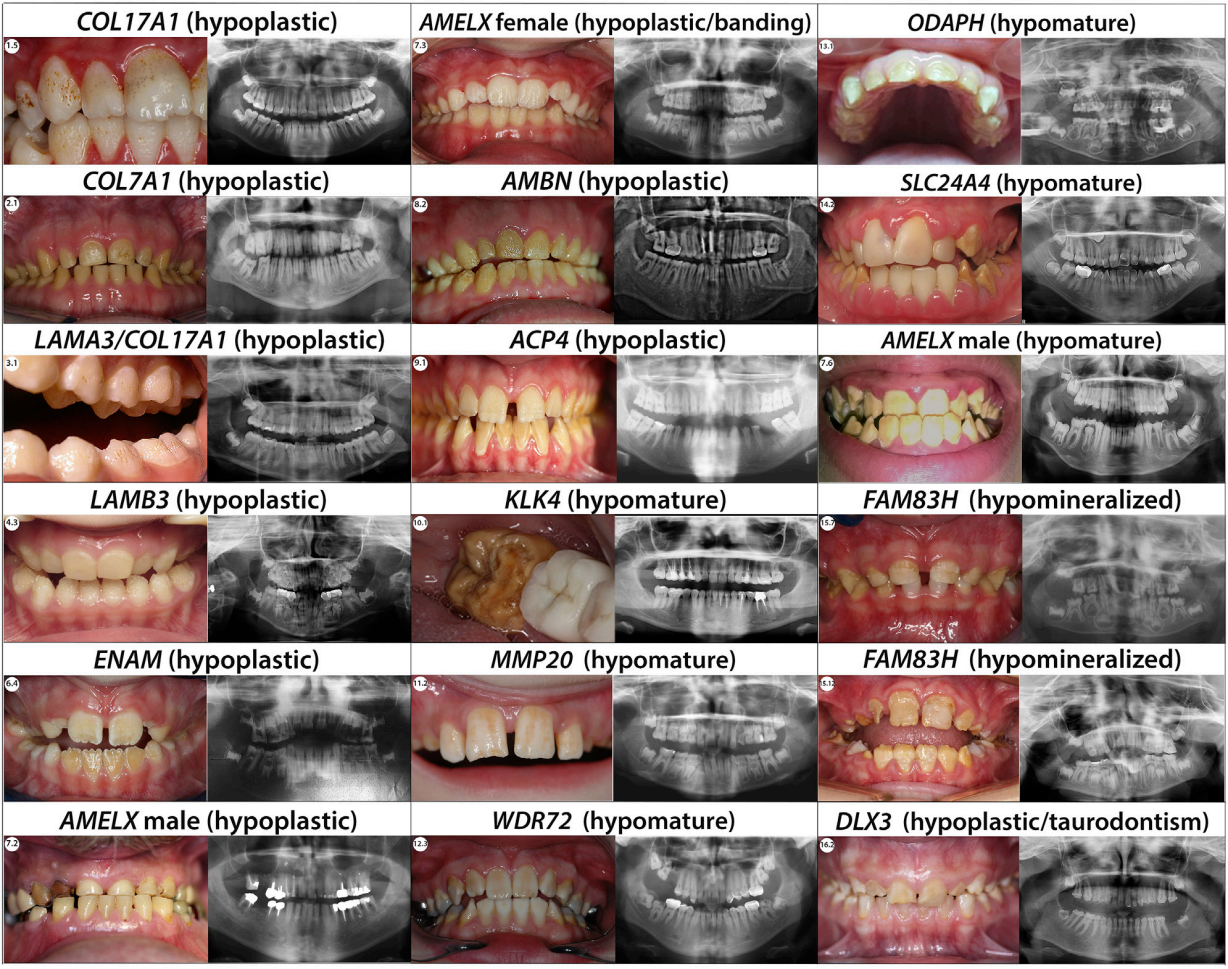
Phenotype/Genotype correlation for isolated AI. Typical phenotype/genotype correlation observed in patients presenting with isolated Amelogenesis imperfecta (intraoral pictures and radiographs). Patients who are carrier of *AMELX* mutations can present with different types of AI. Indeed, the phenotype can be either hypoplastic (severe with almost no enamel in male and with a lyonization banding pattern in female) or hypomature depending on the mutation’s localisation. When the mutations occur in a MMP20 cleavage site the *AMELX* related AI observed is a X-linked hypomature AI.

In our current study, we found six individuals (1.1–1.6) presenting with, hypoplastic pitted AI. The enamel appears pitted, rough, of normal hardness and presents yellow-brown extrinsic stains. Radiographs show normal enamel opacity (Figure 4; Supplementary Figure S1A).

We identified heterozygous loss of function variants in *COL17A1* gene in each of them (Supplementary Table S3; Supplementary Figure S2A). In individuals 1.2, 1.5, and 1.6 the heterozygous variants we identified were originally described as pathogenic in EB individuals. The heterozygous variant found in individual 1.2 was also found in her affected sister and daughters (Supplementary Figure S3.1). The heterozygous variant found in individual 1.6 was also detected in his mother but the parents’ phenotype was not known (Supplementary Figure S3.2). Individuals 1.1, 1.3 and 1.4 and their variants were already described in an autosomal dominant mode by our team in (Prasad et al., 2016b). Heterozygous variants in *COL17A1* gene, historically discovered in EB families, were reported in the literature as responsible for enamel defects and an AI phenotype in heterozygous carriers, including parents non-affected with EB (McGrath et al., 1996; Prasad et al., 2016a; b).

#### COL7A1

We report three individuals (2.1, 2.2 and 2.3) presenting with an hypoplastic pitted AI phenotype with thin enamel and yellow discoloration (Figure 4; Supplementary Figure S1B). Panoramic X-rays show thinner enamel. We found in these 3 individuals *COL7A1* heterozygous variants not previously described (Supplementary Table S3; Supplementary Figure S1B, S2B).

Individual 2.1 has an intronic heterozygous variant (NM_000094.4:c.2440 + 3A>C) in intron 19. This variant is predicted to have an impact on the splicing site possibly leading to an, in phase, exon 18 skipping. Exon 18 codes for a Fibronectin type III domain involved in interactions with integrins. A variant implicating a similar splice site defect has been described in an individual presenting epidermolysis bullosa with enamel defects (c.2440 + 1G>T) (Vahidnezhad et al., 2017).

Individual 2.2 and 2.3 both carry a heterozygous missense variant NM_000094.4:c.3605G>A; p.(Arg1202His) and NM_000094.4:c.3785T>C; p.(Met1262Thr) located respectively in the Von Willebrand factor type A and Collagen triple helix repeat protein domains. No other missense variant was described in the Von Willebrand domain, but other missense variants have been described as pathogenic in the Collagen triple helix domain (Yenamandra et al., 2018). Individual 2.2 has an affected sibling carrying the same variant. Individual 2.3 inherited his variant from his affected mother (Supplementary Figures S3.3, S3.4).

All these variants were, so far, classified as VUS and further investigations would be needed to determine their impacts on the phenotype.

Variants in this gene were only previously reported in individuals with epidermolysis bullosa in autosomal dominant or recessive conditions.

#### LAMA3

We report one individual (3.1) presenting severe hypoplastic AI carrying a mutation in *LAMA3*. A digenic inheritance with variants in both in *COL17A1*, and *LAMA3* has been previously described (Prasad et al., 2016a) (Supplementary Table S3; Supplementary Figure S2C). The heterozygous variant, transmitted by her affected mother, in *COL17A1* NM_000494.4:c.1141 + 1G>A is a pathogenic variant altering the splicing site in exon 14. The individual also carries an heterozygous variant in *LAMA3* not inherited from her mother NM_000227.6:c.1650_1659del; p.(Ile550Metfs*46). We hypothesize this additional mutation could explain the phenotype severity gradient between 3.1 and her mother. Indeed, both are presenting hypoplastic AI but in 3.1 the phenotype is more severe, and the pits are numerous and clearly visible (Figure 4; Supplementary Figure S1C).

#### LAMB3

Each of the individuals reported in this publication (4.1, 4.2, 4.3, 4.4 and 4.5) carrying mutations in *LAMB3* present with hypoplastic AI with an irregular pitted and thinner enamel and no sign of epidermolysis bullosa (Figure 4; Supplementary Figure S1C).

Among the pathogenic variants reported in this gene, bi-allelic loss of function variants was described in patients with severe EB and AI. Dominant heterozygous frameshift variations were reported in patients with isolated AI: all those variations were located in the last two exons of *LAMB3* gene. For these published patients, a dominant negative effect was proposed by Smith (Smith et al., 2019). In our cohort, two patients have such 3′end variations: patients 4.4 and 4.5, NM_000228.3: c.2926del; p.(Val976Trpfs*54) and c.3305del; p.(Gly1102Valfs*7) (Supplementary Figure S3.5; Supplementary Figure S2D).

We also report in our cohort, three patients with isolated AI and a non-sense mutation located before the 3′end of the gene (4.1, 4.2 and 4.3) 4.1 and 4.3 were reported in (Prasad et al., 2016a) and reanalyzed in the context of this publication. Revisiting these data showed that 4.1 also presented a non-sense mutation in position 42 (c.124C>T; p.(Arg42*)); we also detected a new missense variant c.3490C>T; p.(Arg1164Cys) which is predicted to be deleterious by SIFT (v6.2.0) and Polyphen-2. Familial segregation showed that those two variations affected both alleles in this patient (Supplementary Figure S3.6).

Patient 4.2 has one premature stop codon in position 635 and on the second allele, a splice variation was detected, c.1288 + 1G>T (Supplementary Figure S3.7). This variation was previously reported by Kiritsi et al. (2015) and the authors confirmed the impact of the splice variant by mRNA study: the variation induced an in-frame skipping of exon 11 and was predicted to produce an incomplete protein p.(Ser378_Arg430delinsCys). According to (Kiritsi et al., 2015), the patient presented with AI and a mild form of EB with favorable evolution.

The clinical synopsis of *LAMB3*-related AR epidermolysis bullosa, junctional 1A intermediate includes enamel hypoplasia, enamel pitting and corneal erosion, corneal scarring besides skin, hair and nails defects. Notably, individual 4.2 has a history of recurrent corneal ulcers and might therefore present a mild form of EB.

Patient 4.3 has a stop mutation in position 635. A splice variation was also detected in this patient c.944-14C>G but mRNA analysis could not be performed and familial segregation was not possible.

The first two cases (4.1 and 4.2) could thus be compatible with a recessive form of AI: those patients combined one null allele and a possible hypomorphic second allele. An AR clinical continuum may go from severe EB/AI in patients with biallelic null variations to mild EB/AI or isolated AI in patients with one null mutation occurring with a hypomorphic allele.

#### LAMC2

A 4-year-old individual (5.1) displayed a hypoplastic/hypomature AI phenotype. The primary dentition showed thin white opaque enamel (Supplementary Figure S1C).

We found a heterozygous *LAMC2* variant NM_005562.3: c.493C>T; p.(Arg165Cys) with an allele frequency of 0.2% in GnomAD, predicted deleterious by SIFT (v4.0.3) and PolyPhen-2 and located in the Laminin EGF domain (Supplementary Table S3; Supplementary Figure S2E). Heterozygous variants in this gene have not previously been associated with AI in human but this gene is known to be involved in enamel formation defects in mice (Wazen et al., 2016) and the patient’s phenotype is similar to the one observed in mice*.* The allele is inherited from her mother but her phenotype was not available (Supplementary Figure S3.8).

#### ITGB6

Individuals with AR variants in integrin-β6 (*ITGB6*), have been described as affected with hypoplastic pitted and hypomineralized AI. Enamel is less dense, presents disorganized prisms, and severe pitting in the coronal side of tooth with pigmentations (Poulter et al., 2014a; Wang S.-K. et al., 2014; Seymen et al., 2015a). Though this established phenotype infers a Type 1A classification, no *ITGB6* pathogenic variants were found in our cohort.

### Type IB—hypoplastic local, autosomal dominant, #104500

Whereas other genes with this mode of inheritance may yet to be discovered, our survey of the literature and our cohort currently suggest that this classification is composed only for mutations of *ENAM*.

#### ENAM

*ENAM* encodes enamelin, the largest enamel matrix protein, composed of 1,142 amino acid. It is a secretory protein with a 39 amino acid signal peptide. During amelogenesis, the protein is found among the developing crystallites in the enamel rods and interrods (Daubert et al., 2016). Its presence is necessary for correct prism morphogenesis, contributing to thicker enamel (Shore et al., 2010). Variants in ENAM cause hypoplastic AI (Mårdh et al., 2002; Kim et al., 2005a), in which defective enamel is thin or absent, with horizontal row of pits, linear depressions, or one large hypoplastic area. These defects appear most prominent on the buccal surfaces of the teeth involving the middle third of the enamel.

Non-sense variants disrupting the enamelin domain, splice variants, small deletions and insertions and one big insertion have been described to be causative of Amelogenesis imperfecta (Hu and Yamakoshi, 2003).

We identified in our cohort eight individuals with hypoplastic AI manifest as thin, rough enamel in permanent teeth, columns of pits, and linear depressions in enamel. Hypoplastic enamel is clearly identified in panoramic X-Rays especially visible on unerupted teeth (Figure 4; Supplementary Figure S1D).

We detected *ENAM* heterozygous variants in each of these individuals (Supplementary Table S3; Supplementary Figure S2F). Three new, never reported, variants are described in this paper. Individual 6.1 presented a missense heterozygous variant in exon 3 NM_031889.3:c.101T>C; p.(Leu34Pro) (Supplementary Table S3). Missense variants in the N-terminal domain have previously been described, reportedly negatively impacting ameloblast secretory pathway leading to endothelial reticulum (ER) stress and an activated unfolded protein response (Brookes et al., 2017). With this proposed mechanism, a functional analysis could improve identification of the VUS towards pathogenic.

For individual 6.3, we detected a heterozygous 1bp duplication on a splice site NM_031889.3:c.588 + 1 dup; p.(Asn197Glufs*25). This variant was also detected in her affected sister and father (Supplementary Figure S3.9). The variant occurred at the same position than the already described deletion c.588+1delG; p.(Asn197Ilefs*81) found in individuals 6.4, 6.5, 6.6 (Supplementary Figure S3.10) and 6.7 (Supplementary Figure S3.11). Interestingly in 6.3 the phenotype seemed slightly different to the one observed in the four individuals carrying the deletion with an hypoplastic but also hypomature whitish enamel. Individual 6.3 also presented agenesis of 16, 26, 36, 46 but no further variant explaining the missing teeth was identified.

Individual 6.8, his affected sister, and their mother were all heterozygous for a non-sense mutation in exon 9 NM_031889.3:c.664C>T; p.(Gln222*) (Supplementary Figure S3.12).

Truncating variants earlier and later in the protein were already described in this gene and found to be causative of hypoplastic amelogenesis imperfecta (Ozdemir et al., 2005a; Seymen et al., 2014a).

### Type IC -hypoplastic local, autosomal recessive, #204650

*ENAM* mutations also currently comprise the entire classification of type 1C, though further genes may await discovery.

#### ENAM

The phenotype is more severe than in local hypoplastic autosomal dominant AI and has also been linked to *ENAM* variants but in a recessive mode of inheritance (2 alleles affected). This leads to the conclusion that *ENAM* variants associated phenotype is dosage dependent. Indeed, Ozdemir et al., 2005a, Hart et al., 2003 described families for which carriers of only one *ENAM* variant were less affected presenting only localized enamel pitting, whereas family members with compound heterozygous or homozygous *ENAM* variants presented severe hypoplastic AI. Lindemeyer et al., 2010 also described the case of a nine-year-old boy with homozygous variants in *ENAM* and severe hypoplastic AI and smooth teeth.

We did not detect any cases AR mode of inheritance associated with *ENAM* in our cohort.

### Type ID - Hypoplastic, smooth, autosomal dominant

This type of AI shows crowns with a yellow to brown color. The enamel tends to be thin in some teeth and absent in others. The tooth surfaces are smooth to uneven and sensitive to temperature changes and touch. Radiographs reveal a lack of enamel.

As the texture of the crowns is smooth, the attrition is apparent on the occlusal surfaces (Burzynski et al., 1973). No gene has previously been associated to this category, but here we propose *SP6* as the first possible gene classified in this type of AI based on our literature survey.

#### SP6

*SP6* encodes a transcription factor expressed during amelogenesis and involved in regulating proliferation and differentiation of ameloblasts (Nakamura et al., 2004; 2008; Utami et al., 2011; Muto et al., 2012; Ruspita et al., 2020). Autosomal dominant pathogenic variants have been published in two unrelated families in association with severe hypoplastic AI (Smith et al., 2020; Kim et al., 2021b). Interestingly in the two families, the same amino acid (Ala273) was modified. No variant in this gene was evident in our cohort.

### Type IE—hypoplastic, smooth, x-linked dominant, #301200

The surface of the enamel can vary, showing smooth, rough, pitted, or local defects (Witkop, 1988). Due to random X chromosome inactivation in female (Lyonisation effect), differences exist in phenotypic expression between affected males and heterozygous females. Affected males (XY) have only a very thin, smooth enamel, which appears nearly homogeneous. Females (XX) present a milder phenotype with a banding pattern featuring hypoplastic enamel rough/pitted, vertically ridged and normal enamel. Radiographically, thinner enamel contrasts normally from dentin.

#### AMELX

*AMELX*, Amelogenin X-linked, has been identified as the gene involved in this AI. The gene resides in intron 1 of *ARHGAP6* in the opposite orientation (Iwase et al., 2007). Amelogenins are highly conserved proteins secreted by ameloblasts that constitute 90% of the enamel organic matrix. As the proteins are digested and removed in maturation phase, mineral crystals grow in well-organized prism patterns (Gibson et al., 2001).

Genotype-phenotype correlation revealed that variants in the N-terminal (Lagerström et al., 1991; Kim et al., 2004) and C-terminal parts (Greene et al., 2002) of AMELX cause hypoplastic AI.

In our cohort, we identified five females (7.1, 7.3, 7.4, 7.8 and 7.11) presenting the typical hypoplastic AI banding pattern (Figure 4; Supplementary Figure S1E).

We identified heterozygous variation in *AMELX* in each of these individuals (Supplementary Table S3; Supplementary Figure S2G). Female individuals 7.1 to 7.3 were carrying a non-sense *AMELX* variant NM_182680.1:c.11G>A; p.(Trp4*) firstly reported by (Hart et al., 2002b). Individual 7.4 carried a newly reported variant NM_182680.1(*AMELX*):c.47C>A; p.(Ala16Asp) predicted damaging by SIFT (v6.2.0) and Polyphen-2 and inherited by her affected father (Supplementary Figure S3.13). Individuals 7.5 to 7.7 were presenting already reported missense variant. Individuals 7.8 to 7.11 were carrying frameshift variant originally reported by (Lench and Winter, 1995).

Male individual 7.9 is the cousin of 7.8 female and carried the same variant (Supplementary Figure S3.14). He presented the characteristic female lyonisation banding pattern thus requiring further understanding and clarification. His karyotype revealed XXY aneuploidy, the most common disorder of sex chromosomes in humans, with a prevalence of one in 500 males. This finding explained the lyonisation pattern in a male and was consistent with his associated behavioral disorder.

Individual 7.11 is the mother of 7.10 male. The mother presented the typical female hypoplastic banding pattern. The boy showed a severe hypoplastic AI with almost no enamel associated with delayed dental eruption and behavioral issues. As the boy’s phenotype presented as syndromic, this family was directly sequenced by whole exome sequencing. We identified a pathogenic 1bp deletion leading to a premature stop codon c.541del; p.(Leu181Cysfs*8), a mutation previously reported (Kindelan et al., 2000) in both the mother and the boy (Supplementary Figure S3.15). This variation clearly explained the AI phenotype but no other variation was discovered as possibly explaining behavioral issues presented by this boy.

Male individuals 7.5, 7.6 and 7.7 presented a different hypomature amelogenesis imperfecta phenotype with a smooth yellow opaque enamel (Supplementary Figure S1E). Individuals 7.6 and 7.7 carry a c.208C>A; p.(Pro70Thr) variant first described by Collier et al., 1997 (Supplementary Figures S3.16, S3.17). Functional analyses showed that this variant is reducing the interactions between amelogenin and the MMP20*,* metalloproteinase degrading amelogenin in the maturation phase (Tanimoto et al., 2008). Therefore, depending on the patho-physiological mechanism, *AMELX* might also be the gene involved in the hypomature form of AI type IIC (snow-capped teeth X-linked) or eventually IIB (hypomaturation X-linked). This would be discussed further in these mentioned categories.

Individual 7.7 also had a frequently reported heterozygous variant in *WNT10* gene (c.682T>A; p.(Phe228Ile), explaining the 12, 15, 22, 28 agenesis.

### Type IF—amelogenesis imperfecta, hypoplastic, autosomal recessive #616270

Hypoplastic enamel in the primary and permanent dentitions, showing a rough and discolored appearance is the hallmark of this AI. The enamel may be absent, pitted, or thinner. We currently list only *AMBN* as the causative gene defect in this class.

#### AMBN

*AMBN* gene (4q13.3), containing 13 exons was discovered as the causative gene for non-syndromic autosomal recessive amelogenesis imperfecta (Toyosawa et al., 2000). It encodes ameloblastin (AMBN, also named “amelin” or “sheathlin”), the second most abundant enamel matrix protein, critical to amelogenesis (Lu et al., 2018).

Four cases have been reported so far in the literature with AMBN variants associated with non-syndromic AI (Poulter et al., 2014b; Lu et al., 2018; Liang et al., 2019).

In our cohort we identified three individuals with a hypoplastic amelogenesis imperfecta combined with recessive variants in *AMBN* (Supplementary Table S3; Figure 4; Supplementary Figures S1F, S2H). Individuals 8.1, and 8.2 presented compounds heterozygous variants never described before. 8.1 displayed a splice variant in intron 1 NM_016519.6:c.15 + 1G>A and a non-sense variant in exon 8 c.577G>T; p.(Gly193*) (Supplementary Figure S3.18). 8.2 was carrying a non-sense variant in exon 5 NM_016519.6:c.209C>G; p.(Ser70*) and a splice variant in intron 7 c.571-1G>C. The non-sense mutation hadn’t previously been described, however Poulter et al. (2014b) described a large deletion resulting in the loss of exon 6. Individual 8.3 was already described in (Prasad et al., 2016a). We detected a homozygous splice variant in intron 6 never described before NM_016519.6:c.532-1G>C. This is the first splicing variant described in this gene.

### Type IG—enamel agenesis, autosomal recessive enamel-renal syndrome (ERS), #204690

Commonly described as an isolated AI, enamel agenesis may be observed concomitantly with other orodental and/or systemic features such as nephrocalcinosis in Enamel Renal Syndrome (ERS, MIM#204690), or gingival hyperplasia in Amelogenesis Imperfecta and Gingival Fibromatosis Syndrome (AIGFS, MIM#614253 moved to MIM#204690) (O’Sullivan et al., 2011).

FAM20A

These conditions are allelic, and are now considered as a spectrum of ERS and due to recessive variants in *FAM20A* gene (17q24.2; 11 exons (Nalbant et al., 2005; Jaureguiberry et al., 2012). FAM20A is a secreted glycoprotein, Golgi Associated Secretory Pathway Pseudokinase and its intact signal sequence is required for secretion. Various human tissues reveal high transcript levels in lung and liver and intermediate levels in thymus and ovary (Nalbant et al., 2005).

The clinical phenotype is highly distinctive and when recognized leads to the clinical diagnosis and the subsequent exploration *via* ultrasound of the presence of renal calcifications.

The diagnostic criteria of ERS are based on oral findings, including clinical (severe enamel hypoplasia, delayed or absent tooth eruption, gingival hyperplasia) and radiographic signs (non-erupting teeth, pulp calcifications and hyperplastic dental follicle) (de la Dure-Molla et al., 2014). Associated focal ectopic calcification features, along with the presence of renal abnormalities are also pathognomonic of ERS (Torres et al., 2018). Dental defects are present since the primary dentition, with no declared tooth pain or sensitivity (Dourado et al., 2019). Nephrocalcinosis (NC) and other kidney disorders have been included as frequent findings, especially in the early adulthood (Dellow et al., 1998; Martelli-Júnior et al., 2011). In that sense, it is speculated that even those individuals with oral characteristics showing no renal defects, but with biallelic *FAM20A* variants, will eventually develop NC (Dourado et al., 2019) and should be tested for and followed for NC and other renal defects.

As the phenotype was initially described as isolated AI, it appears in “isolated” AI in the original classification. However, as nephrocalcinosis is observed in these patients, it should be reclassified as syndromic AI. Therefore, data concerning patients with *FAM20A* variations will be discussed in the syndromic section (Table 2, Supplementary Figures S1M, S2R, S3.39, S3.40, S3.41, S3.42).

### Type IJ—amelogenesis imperfecta, autosomal recessive, #617297

In this type of AI, the enamel is thin with irregular surface and teeth appears yellow.

#### ACP4

This phenotype is associated with recessive homozygous or compound heterozygous variants in *ACP4* gene encoding testicular acid phosphatase (Seymen et al., 2016; Smith C. E. et al., 2017). All variants reported so far were localized in the histidine phosphatase domain. This domain contains conserved Histidine residues that are phosphorylated during the catalytic activity of the protein and other residues that are forming a phosphate pocket and interact with the phospho group of substrates before, during and after its transfer to the Histidine residue. Variants were reported mostly in exon 4 and 7 and only one was reported in exon 3 (Seymen et al., 2016; Smith C. E. et al., 2017).

We found three unrelated families (individuals 9.1, 9.2 and 9.3) with hypoplastic amelogenesis imperfecta, with thin enamel with irregular surface, and a yellowish color. X-rays showed a thinner enamel of normal density, long teeth, and slim roots (Figure 4; Supplementary Figure S1G).

These families were previously negative on the GenoDENT panel first version (Prasad et al., 2016a), and were further investigated by whole exome sequencing analysis. We found compound heterozygous variants in the *ACP4* gene (Supplementary Table S3; Supplementary Figure S2I). Indeed *ACP4,* discovered in 2016 (Seymen et al., 2016), was not on the first version of GenoDENT but was added at version 0.4. Individual 9.1 had a previously reported missense variant in exon 4 NM_033068.3:c.331C>T; p.(Arg111Cys) and a splice variant in intron 6 c.645 + 1G>A; p.? which was predicted to lead to an in frame exon 6 skipping. This is the first case reported incidence of a splice variant of *ACP4*. Individual 9.2 had an already described missense variant in exon 4 NM_033068.3:c.428C>T; p.(Thr143Met) and a second variant not yet reported in exon 7, in the histidine phosphatase domain c.736G>A, p.(Val246Met). This variant is predicted damaging by SIFT (v6.2.0) and Polyphen-2. Individual 9.3 had 2 new missense variants, the first one is in exon 6, NM_033068.3:c.626T>C; p.(Leu209Pro) is in the domain but the second one is in the exon 11, in cytoplasmic domain c.1199C>A; p.(Ala400Asp). No variants were reported in the cytoplasmic part of the protein. Our results are generally consistent with previously described variants, but also suggest that the cytoplasmic region of the protein, as well as the extracellular region could have a role in amelogenesis.

Individual 9.3 also had agenesis of teeth 18, and 28, consistent with one of the most frequent variant found in *WNT10A* gene NM_025216.3:c.682T>A; p.(Phe228Ile).

### Type II—Hypomaturation

Hypomature amelogenesis imperfecta relates to a qualitative rather than a quantitative (hypoplastic) enamel defect. Enamel maturation begins when final enamel thickness has been reached and concentrates on proteolytic degradation and removal of secreted matrix proteins as well as on hydroxyapatite crystal growth (Robinson, 2014). Enamel might be less translucid, opaque (snow-capped), pigmented but rather hard and protective. Two forms of hypomature amelogenesis imperfecta - the pigmented and the snow-capped are autosomal dominant (type IIA and IIC) and two are X-linked (type IIB and IID) (Witkop and Sauk, 1976).

### Type IIA–hypomaturation, pigmented, autosomal recessive, A1 #204700 *KLK4*; A2 # 612529 *MMP20*; A3 # 613211 *WDR72*; A4 # 614832 *ODAPH*; A5 # 615887 *SLC24A4*; A6 # 617217 *GPR68*

This type of AI is characterized by pigmented hypomature enamel. The overall volume of the teeth is preserved, but enamel is hypomineralized therefore not differentially contrasting from dentin on X-rays. Enamel is colored from white opaque to brown. It is prone to post-eruptive breakdown. These AI have been associated with homozygous or compound heterozygous variants in six genes: *KLK4, MMP20, WDR72, C4ORF26, SLC24A4,* and *GPR68*.

All individuals (Figure 4; Supplementary Figure S1H,I) we are reporting in this section present a hypomature AI phenotype. Radiographs showed a lack of contrast between enamel and dentin.

#### KLK4

*KLK4*encodes an enamel matrix protease requiring cleavage for its activation after secretion by ameloblasts. *KLK4* encodes a serine protease that, once activated during maturation stage, will degrade enamel proteins to allow the growth in width and thickness of enamel crystallites (Simmer et al., 2009).

Truncating variants in this gene have been previously described (Wang et al., 2013; Seymen et al., 2015b; Smith C. E. L. et al., 2017). In our cohort, individual 10.1 displayed pigmented hypomature, hypomineralized enamel. As a compound heterozygous, he is carrying a previously reported non-sense variant (Hart et al., 2004) associated with a new missense mutation in *KLK4* NM_004917.4: c.443G>T; p.(Cys148Phe) and c.458G>A; p.(Trp153*) (Supplementary Table S3; Supplementary Figure S2J and Supplementary Figure S3.19). The missense variant is affecting a highly conserved amino-acid and is predicted to be deleterious by SIFT and Polyphen-2. This is the first report of the putative pathogenic missense variants c.443G>T; p.(Cys148Phe) in this gene.

#### MMP20

*MMP20* encodes a zinc-dependent endopeptidase activated during the secretion stage and continuing to be expressed by ameloblasts during the maturation stage. It supports enamel maturation by degrading the enamel protein matrix. As KLK4 and MMP20 seem to work in a collaborative manner, it is not surprising to note a similar dental phenotype in individuals with *KLK4* (Hart et al., 2004) or *MMP20* (Kim et al., 2005b) variants. We found 16 individuals (Supplementary Table S3; Supplementary Figure S2K) presenting with hypomature AI both in their primary and permanent dentitions; their phenotypes are similar to the ones described in the literature and associated with *MMP20* recessive inheritance (Kim et al., 2005b; Ozdemir et al., 2005b; Papagerakis et al., 2008; Lee et al., 2010; Gasse et al., 2013; 2017; Kim et al., 2017; 2020; Wang et al., 2020; Nikolopoulos et al., 2021).

Individuals 11.1, 11.2, 11.3 and 11.4 have compound heterozygous variants in *MMP20* gene (Supplementary Figures S3.20, 3.21, 3.22). All have in common the first variant NM_004771.4: c.103A>C; p.(Arg35 = ). This pathogenic variant was firstly reported in Prasad et al., 2016a in individual V2.13. Its splicing impact was then functionally demonstrated by Kim et al., 2020. For individual 11.4 the second variant c.1362C>G; p.(Tyr454*) is novel and has been discovered thanks to GenoDENT panel. Other variants truncating the protein were already described in this gene (Papagerakis et al., 2008; Kim et al., 2017) but never so late in the protein. Indeed c.1362C>G; p.(Tyr454*) occurred in the last exon, inducing the truncation of the hemopoxin domain involved in binding inhibitors of metallopeptidases. It is also plausible that the domain may restrict cleavage site selection (Lee et al., 2010). We have tentatively classified this variant as probably damaging. We also report another new heterozygous pathogenic mutation found in individual 11.8 c.359dup; p.(Asn120Lysfs*9), along with a second variant on the other allele (Supplementary Figure S3.23) already reported c.954-2A>T (Kim et al., 2005b). This new variant was classified as probably damaging in light of later truncations being so described (Kim et al., 2017). The GenoDENT panel highlighted a compound heterozygous VUS in individual 11.11 c.530G>A; p.(Gly177Glu) associated to a known pathogenic variant (c.954-2A>T) (Kim et al., 2005b) (Supplementary Figure S3.24). This missense VUS creates an amino-acid substitution of the conserved Gly177 in the Matrixin protein domain, involved in the peptidase activity, and is likely predicted deleterious by SIFT (v4.0.3), VariantTaster (v2021) and PolyPhen-2. This VUS was not previously reported in the GnomAD database.

Individuals 11.12 and 11.13 both have a heterozygous variant NM_004771.4:c.566T>C; p.(Leu189Pro) already reported by the team (Gasse et al., 2017) along with respectively c.910G>A; p.(Ala304Thr) described in (Lee et al., 2010) and a novel variant in individual 11.3 reported c.1126C>T; p.(Gln376*). Due to the lack of parental DNA, we could not confirm the relative position of the variant in individual 11.13. It would however lead to the loss of almost all the hemopoxin domain, so, we classified this variant as probably damaging. Individuals 11.14 to 11.17 are all homozygous for the pathogenic variant c.954-2A>T (Supplementary Figure S3.25).

#### WDR72

*WDR72* is a transporter, it encodes a protein predicted to be an intracellular vesicle coat protein that is mostly expressed during maturation stage but also at secretory stage. The stronger expression has nevertheless been detected during maturation initiation (El-Sayed et al., 2009; Katsura et al., 2014). WDR72 has recently been confirmed as regulating vesicle trafficking in ameloblasts (Katsura et al., 2022) and being involved in distal renal tubular acidosis (Khandelwal et al., 2021).

We identified four individuals with hypomature AI (Figure 4).

New variants in *WDR72* were identified by GenoDENT (Supplementary Table S3; Supplementary Figure S2L) in individuals 12.1, 12.2 and 12.4. Individual 12.1 presented a homozygous deletion including a part of intron 1 and the beginning of exon 2 NM_182758.4:c.-13 + 989_7del. In individual 12.2, we detected, a non-sense mutation in exon 2 and a 10 bp deletion at the intron 9 splice site. Truncating variation are known to be pathogenic but no splice variants were previously described in this gene. Intriguingly, the 12.2 phenotype is more complex than in other reported individuals as this patient also presented with small teeth, tooth agenesis, mild deafness and nephrocalcinosis. The recent description of the role of WDR72 in kidney-associated diseases and the syndromic nature of the diseases presented by 12.2 would demand possible future consideration of WDR72 in a class of syndromic AI. Individual 12.3 is compound heterozygous for two known pathogenic variants reported in (Prasad et al., 2016a). In individual 12.4 we detected two novel compound heterozygous 1 bp deletion in exon 15 c.2388del; p.(Lys796Asnfs*16) and c.2146del; p.(Ala716Profs*10), resulting in a frameshift and a premature stop codon a known mechanism in this disease (Supplementary Figure S3.26).

#### ODAPH

C4ORF26, named also ODAPH, odontogenesis associated phosphoprotein, function is not yet well defined. *ODAPH* encodes a proline rich protein expressed during the secretory and maturation stage, suggesting a possible role in hydroxyapatite crystallization (Parry et al., 2012). ODAPH is important to maintain the integrity of the atypical basal lamina at maturation stage (Ji et al., 2021). Our cohort included individual 13.1 with hypomature AI (Figure 4). This individual was previously described in (Prasad et al., 2016b). He carries a small homozygous deletion NM_178497.5:c.39_46del; p.(Cys14Glyfs*18) identified using the GenoDENT panel (Supplementary Table S3; Supplementary Figure S2M). The phenotype was consistent with previous individuals described with truncating variants in this gene (Parry et al., 2012).

#### SLC24A4

SLC24A4 is a potassium-dependent sodium/calcium exchanger expressed by maturation stage ameloblasts (Hu P. et al., 2012). It likely performs a role in calcium provision to the enamel during maturation (Wang S. et al., 2014). One large deletion encompassing intron 14 to exon 17 has been described (Seymen et al., 2014b) whereas Prasad et al., 2016a described a homozygous deletion including the last three exons (15,16 and 17) and Parry et al., 2013 a non-sense homozygous variant.

Two individuals (14.1 and 14.2) with hypomature AI (Figure 4) showed novel homozygous *SLC24A4* variants using our GenoDENT panel (Supplementary Table S3; Supplementary Figures S1I, S2N). Individual 14.1 was already described in (Prasad et al., 2016a) with a homozygous deletion including the 3 last exon (15,16 and 17) of the *SLC24A4* gene NM_153646.4:c.(1,537 + 1_1538-1)_*67. Individual 14.2 and her affected sister carried a homozygous variant affecting a splice site with strong exon skipping predictions c.1716 + 5G>A (Supplementary Figure S3.27). No splice variant has previously been reported so we have classified this variant as a VUS pending functional analysis.

#### GPR68

GPR68 is a proton-sensing protein present during all stages of amelogenesis. It has been suggested that the protein acts as a pH sensor directing ameloblasts to switch between the ruffle ended and smooth ended conformations during the maturation stage (Parry et al., 2016b).

pH-Sensing G-protein-Coupled Receptor (GPR68) has been showed to be implied in ameloblast (Ludwig et al., 2003; 2003; Tomura et al., 2008; Frick et al., 2009) and odontoblast (Yang et al., 2006; Pereverzev et al., 2008) function. First human pathogenic variants were reported in (Parry et al., 2016b) associated with hypomaturation AI in three families (#617217). The clinical phenotype showed an enamel of apparent normal thickness but poorly mineralized, with brown discoloration. Weaker enamel tended to be prematurely lost secondary to attrition or masticatory stress, especially in the posterior teeth. Only few other cases, were additionally published, associated with the same type of AI (Seymen et al., 2021; Spedicati et al., 2021). No variants in this gene were found in our cohort.

### Type IIB–hypomaturation, x-linked recessive

No gene has been clearly implicated in this category but we hypothesize that such phenotypes may be due to *AMELX* defects. Indeed, when *AMELX* mutations occur at specific MMP20 cleavage sites, the resulting phenotype is impaired matrix degradation and hypomature enamel. Thus, this category reasonably includes male individuals 7.5, 7.6 and 7.7 presenting a hypomature phenotype (Suplementary Figure 1E).

### Type IIC–snow capped teeth, X-linked

In this form, both primary and permanent dentitions were affected. In males, primary teeth were opaque ground-glass white, and secondary teeth were mottled yellow-brown and white. Enamel had normal thickness, moderately soft, and did not contrast from dentin on x-ray. The teeth chipped and abraded more easily than normal teeth, but the loss of enamel was not as rapid as in the hypocalcified form (Rathi et al., 2014). Because of the appearance of the teeth in this form, referred to as snow-capped in its most marked form, confusion with fluorosis sometimes occurs (Rao and Witkop, 1971).

#### AMELX

Complete deletion of *AMELX* has been associated to this phenotype (Hu J. C.-C. et al., 2012).

We did not find this phenotype among our cohort.

### Type IID—snow capped teeth, autosomal dominant

As with Type IIC, the phenotype is that of snow-capped appearance, except the X-linkage can be ruled out based on male:female incident rates. No gene has yet been implicated in this category. Although we identified individuals in our cohort with snow-capped teeth, we could not define a gene potentially responsible for this clinical entity.

### Type III—hypomineralization

Hypocalcified amelogenesis imperfecta is characterized by an enamel of normal thickness but soft, porous and shedding easily from the dentin. The color of enamel can range from white to creamy yellow (Mendoza et al., 2007).

Enamel can be easily lost after eruption (post eruptive breakdown). These teeth are very sensitive even to physical contact with a toothbrush. Oral microbiome evolving in calculus is largely depositing on teeth resulting in severe gingivitis. Both autosomal dominant and autosomal recessive classifications are described related to three known genes in total.

Two categories are described: autosomal dominant (type IIIA associated to *FAM83H* variants and IIIB associated to *AMTN* variants) and - autosomal recessive (type IIIC associated to *RELT* variants).

We identified individuals with variants in *FAM83H* gene, we didn’t detect any individual with *AMTN* or *RELT* variants in our cohort.

### Type IIIA—amelogenesis imperfecta, hypomineralization type autosomal dominant #130900

To date, two genes are associated to the hypomineralized autosomal dominant inheritance subtype of AI.

#### FAM83H

Family with sequence similarity 83, member H (FAM83H), is an intracellular protein with ubiquitous expression (Lee et al., 2011). It reaches maximum expression in ameloblasts during the secretory stages. FAM83H regulates the organization of the keratin cytoskeleton and is involved in desmosome formation (Kuga et al., 2016). Variants identified in *FAM83H* cause autosomal dominant hypocalcified AI (Mendoza et al., 2007; Kim et al., 2008).

We report 15 individuals (15.1-15.15) presenting with hypocalcified amelogenesis imperfecta (Figure 4), affecting both primary and permanent dentitions with secondary loss of enamel, and colored teeth. Occlusal and incisal wear gave a conical form to the canines. There was no difference in radio-opacity between enamel and dentin.

The 15 individuals carried autosomal dominant variants in exon 5 (Supplementary Table S3; Supplementary Figures S1J, S2O, S3.28–3.37). This is consistent with previously reported variants as each of the variants identified to date have mapped to this largest and final exon of *FAM83H*. All of them except the one encountered in 15.13 are frameshift or non-sense variants, a mechanism already described as disease causing. Only three of those individuals had a novel variant: 15.1 NM_198488.5:c.930_939dup; p.(Val314Argfs*14), 15.6 c.1309_1311delinsTAG; p.(His437*) and 15.9 c.1375C>T; p.(Gln459*). Individual 15.4 c.1282C>T; p.(Gln428*) carries a variant previously described in (Prasad et al., 2016a). Individual 15.13 is the only one presenting a missense variant NM_198488.5:c.1498C>G; p.(Leu500Val). He also displays a variant in *WDR72* NM_182758.4:c.1283T>G; p.(Ile428Ser) (Supplementary Figure S3.36). The phenotype in this individual appears more severe possibly due to the compound effect of the two variants or to his more advanced age and accentuated tooth wear.

#### AMTN

Amelotin, encoded by *AMTN*, is a proline, leucine, threonine and glutamine rich protein binding to ODAM (odontogenic, ameloblast associated) and SCPPPQ1 (secretory calcium-binding phosphoproteins proline-glutamine rich 1) to form aggregates able to maintain the attachment between ameloblasts and the produced enamel during maturation stage (Holcroft and Ganss, 2011; Fouillen et al., 2017).

A large deletion in *AMTN* spanning exon 3–6 has been reported yet to cause hypomineralized AI (Smith et al., 2016). No variant in this gene was found among our cohort.

### Type IIIB or IIIC-amelogenesis imperfecta, hypomineralization type autosomal recessive #618386

This type of AI is characterized by hypocalcified enamel in both primary and permanent dentitions. A normal or near-normal enamel volume can be found prior to tooth eruption. Post-eruptive changes are rapid and lead to enamel loss, enamel disintegrates from occlusal surfaces of the molars, leaving a ring of intact enamel remaining on the sides. Some people also have anterior open bite (Kim et al., 2019; Nikolopoulos et al., 2020). Homozygous frameshift, missense, and splice junction variants in *RELT* have been described in affected individuals (Kim et al., 2019).

#### RELT

The protein encoded by this gene is a member of the TNF-receptor superfamily able to activate the NF-kappaB pathway and selectively bind TNF receptor-associated factor 1 (TRAF1).

In mice, *Relt* is expressed in the odontoblast and ameloblast layers, specifically in secretory stage ameloblasts where there is ∼20-fold higher expression than in maturation stage ameloblasts. It has been published that *Relt* ^−/−^ incisor enamel was of normal thickness but rough-surfaced and generally hypomineralized correlating with the phenotype found in humans. *RELT* was originally classified as causative of a new type of AI called type IIIC (#618386) but following Witkop’s classification, as recessive hypocalcified hypoplastic AI, it could be classified in type IIIB.

We did not detect any individuals with *RELT* variants in our cohort.

### Type IV—hypomaturation-hypoplastic with taurodontism #104510

Amelogenesis imperfecta, hypomaturation-hypoplastic type or hypoplastic-hypomature type, with taurodontism (AIHHT) is an autosomal dominant trait associated with enamel defects and enlarged pulp chambers (Dong et al., 2005). The difference between the two subtypes is slight and is based on the dominance of the hypomaturation *versus* the hypoplastic phenotype. So far, only one gene (*DLX3*) was associated to the hypomature-hypoplastic type IVA and no gene was described for the hypoplastic-hypomature type IVB.

### Type IVA—hypomaturation-hypoplastic with taurodontism autosomal dominant #104510

For this phenotype, enamel thickness is supposed to be normal and hypomaturation is predominant over hypoplasia. Enamel appears as mottled yellow white to yellow brown with pits on the buccal surfaces. Taurodontism with large pulp chambers is observed on radiographs. Only one gene has thus far been proposed to cause this defect: *DLX3* (Wimalarathna et al., 2020).

#### DLX3

*DLX3* is an important transcription factor involved in osteogenic differentiation (Sun et al., 2019). It is located on chromosome 17q21.3-q22, and contains 3 coding exons (Scherer et al., 1995; Price et al., 1998). *DLX3* plays a role in craniofacial development, and in the development of the ventral forebrain. DLX3 has three main domains: The N- and C-terminus transactivation domains, and a central sequence-specific DNA-binding distal-less-like homeodomain, encoded by exons 2 and 3. The homeodomain can interact directly with DNA in a sequence-specific way and regulates the expression of target genes throughout numerous developmental processes (Whitehouse et al., 2019). Only one missense variant had been described in *DLX3* gene for AI with taurodontism and attenuated tricho-dento-osseous syndrome in exon 2 by (Whitehouse et al., 2019).

Individuals 16.1 and 16.2 showed hypoplastic amelogenesis imperfecta with taurodontism (Figure 4), and enamel surface with striae (16.1), or thinner enamel (16.2). Taurodontism was really apparent on 16.2. 16.3 presented an hypomature/hypoplastic enamel and smaller 12, 22, as well as probable third molar agenesis. Dental agenesis could be linked to the additional variant discovered in *WNT10A* gene NM_025216.3:c.637G>A; p.(Gly213Ser). Dental radiographs confirmed the decreased thickness of enamel, and various degrees of molar taurodontism (Supplementary Figure S1K). In 16.1 we detected a heterozygous missense variant in exon 1 NM_005220.3:c.92C>G; p.(Thr31Ser) predicted possibly damaging by Polyphen-2 and localized in the distal-less-like homeobox protein domain. In individual 16.2 the heterozygous variant was located in exon 3 c.537C>A; p.(Asn179Lys) concerning a well conserved amino acid and predicted damaging by SIFT (v6.2.0) and Polyphen-2. Individual 16.3 carries the variant c.710A>G; p.(Tyr237Cys) (Supplementary Table S3; Supplementary Figure S2P). 16.1-16.3 did not present any bone nor hair additional phenotypes.

As this gene has also been linked to tricho-dento-osseus syndrome, we will describe further syndromic individuals linked to *DLX3*, in the coming syndromic section of this publication, illustrating therefore the tight and thin border between isolated and syndromic AI.

### Type IVB—hypoplastic-hypomaturation with taurodontism, autosomal dominant

Enamel is thin with big hypoplastic areas. Hypoplasia is more pronounced than hypomaturation. Taurodontism with large pulp chambers is observed on X-Rays. No gene has ever been implicated with this phenotype (Wimalarathna et al., 2020). We suggest that types IVA and IVB might be a single subtype.

### Syndromic amelogenesis imperfecta

Amelogenesis imperfecta can be found in isolation as previously described but also in association with extra-oral clinical signs (Supplementary Table S4). Recognition of AI subtype as well as associated symptoms could orientate clinical diagnosis, refine genetic diagnosis and contribute to improving patient care. After identification of the implicated gene, retro-phenotyping will also help assess and confirm overall clinical diagnosis.

**FIGURE 5:**
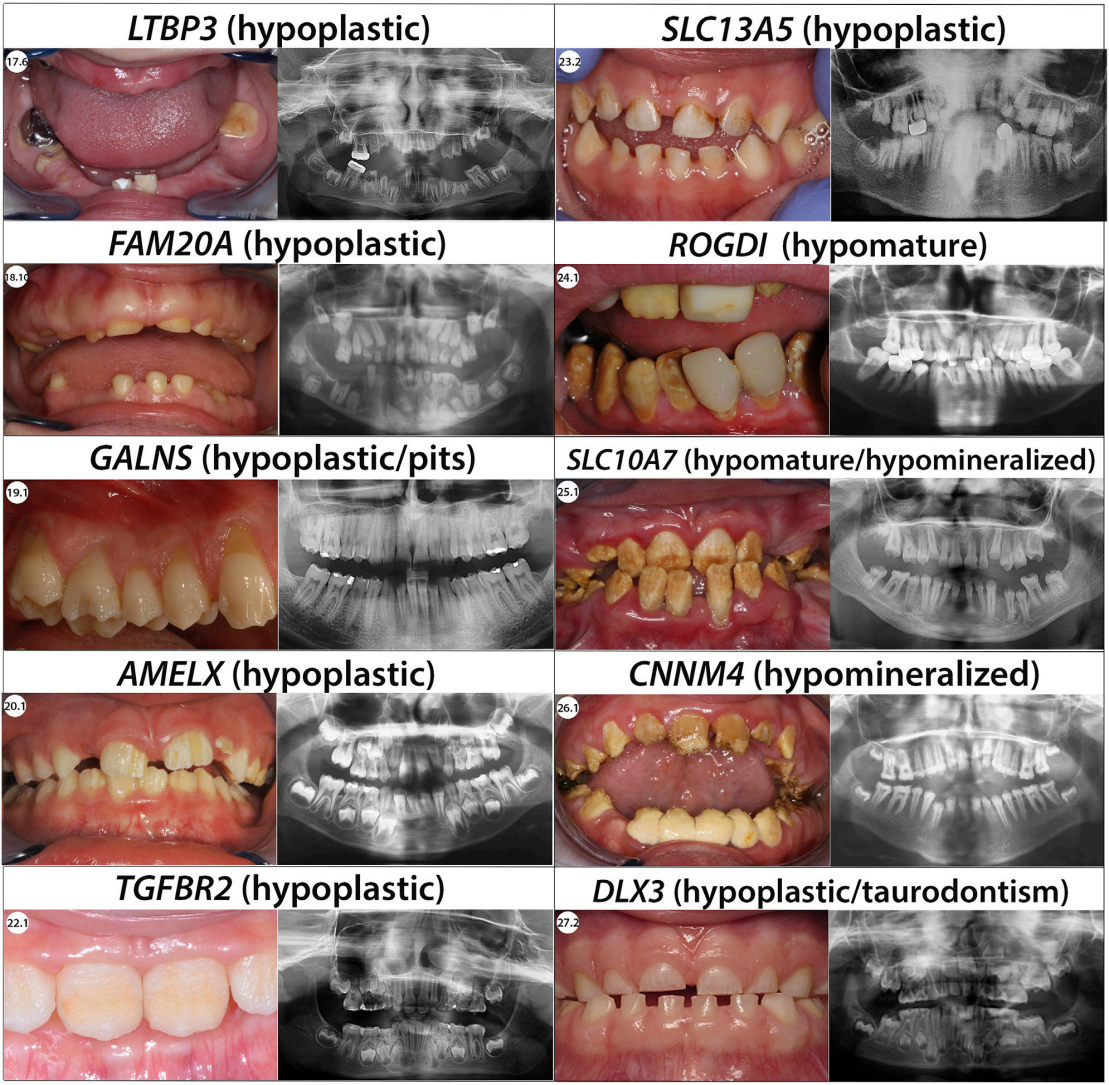
Phenotype/Genotype correlation for syndromic AI.

In this paper we are discussing extra-oral key phenotypes for syndromes in which enamel defects are well characterized. Other genes associated to syndromes with insufficient characterization or minor enamel defects are classified by major extra oral clinical signs. To facilitate recognition of AI subtypes and further diagnosis we will describe and classify syndromic AI according to the 3 main defects categories, hypoplastic, hypomature and hypomineralized AI and their mode of inheritance (Figure 5; Table 2; Supplementary Table S4).

#### Syndromic hypoplastic AI

Hypoplastic AI is the hallmark of numerous syndromes. To date 22 genes have been associated to syndromes including hypoplastic AI in their clinical synopsis. Thirteen genes (*FAM20A*, *GALNS*, *TSC1*, *TSC2*, *TP63*, *MSX2*, *FAM20C*, *ARHGAP6*, *RAI1*, *PEX1*, *PEX2*, *PEX26, TGFBR2* and *ATP6V1A*) have been associated with clinical signs within the head and neck area, eleven with skin, nail and hair defects (*TSC1*, *TSC2*, *TP63*, *ARHGAP6*, *PORCN*, *TGFBR2*, *PEX1*, *PEX2*, *PEX26*, *ORAI1* and *STIM1*), three with immune deficit (*AIRE*, *ORAI1* and *STIM1*), seven with skeletal defects (*LTBP3*, *GALNS*, *TP63*, *MSX2*, *PORCN*, and *FAM20C*), five with neurological issues (*ATP6V1A*, *SLC13A5*, *PEX1*, *PEX2* and *PEX26*), two with cardiovascular defects (*LTBP3, TGFBR2*), and eight with genitourinary defects (*FAM20A* and *FAM20C*, *CLDN19*, *CLDN16*, *WDR72, TSC1, TSC2, RAI1*). It is therefore of importance to explore these potential associated phenotypes while taking medical history and examination.

##### Dental anomalies and short stature DASS #601216 Verloes Bourguignon syndrome, Platyspondyly with hypoplastic AI absent enamel—AR—*LTBP3*

DASS is characterized by short stature with brachyolmia as well as hypoplastic amelogenesis imperfecta with almost absent enamel (Huckert et al., 2015). Some individuals exhibit valvular and/or vascular defects, including mitral valve prolapse, aortic root dilation, and aortic as well as other arterial aneurysms and dissections (Dugan et al., 2015; Guo et al., 2018).

Associated tooth agenesis was described by (Noor et al., 2009; Dugan et al., 2015). Clinical oral examination showed also microstomia, tooth crowding, high arched palate. Teeth were small and had a yellowish color. Radiographic findings included thin enamel with reduced radiopacity, irregular alveolar bone level, and alveolar bone infectious lesions (Intarak et al., 2019).

#### LTBP3

DASS is an autosomal recessive disorder caused by homozygous or compound heterozygous variants in *LTBP3* gene (11q12) (Li et al., 1995; Huckert et al., 2015). *LTBP3* encodes latent transforming growth factor-beta-binding protein 3, modulating TGFbeta bioavailability in the extracellular matrix. Deleterious variants in *LTBP3* have also been associated with autosomal dominant Geleophysic dysplasia 3 (#617809). Ultrastructural enamel defects showed an absence of initial aprismatic enamel layer and an abnormal secretion of non-prismatic bulk enamel, suggesting LTBP3 plays a role in the life cycle of ameloblasts especially at the secretory stages with Tomes process formation (Huckert et al., 2015).

We identified *LTBP3* as the gene behind short stature and absent enamel (individuals 17.1–17.4) using exome sequencing (Huckert et al., 2015). GenoDENT panel was subsequently exanded with addition of this gene and we identified in 2 unrelated consanguineous families, additional individuals 17.5 and 17.6 with a similar phenotype and two new homozygous loss of function variants (Table 2; Supplementary Figures S1L, S2Q, S3.38). Teeth were small, spaced, and had a yellowish color. No enamel, impacted teeth, and irregular alveolar bone level were visible on panoramic radiographs. The first pathogenic variant (Individual 17.5) NM_001130144.3:c.3087del; p.(Asn1030Thrfs*47) was a one nucleotide deletion leading to a frameshift and a premature stop codon. The second variant (17.6) was an intronic mutation c.3629-2A>G which led to an aberrant exon 27 splice site. This would probably induce exon 27 skipping resulting in a 132 bp in phase deletion equivalent to a 44 amino acid deletion (position 1,211–1,254).

##### Mucopolysaccharidosis type IVA #25300—AR—*GALNS*

Mucopolysaccharidosis type IVA is characterized by intracellular accumulation of excessive glycosaminoglycans (GAGs): chondroitin-6-sulfate (C6S) and keratan sulfate (KS) mainly in bone, cartilage, and its extracellular matrix. GAG accumulation leads to unique skeletal dysplasia in MPS IVA individuals.

Most MPS IVA individuals usually look healthy at the neonatal period; however, bone abnormalities in the spine can be seen through X-rays even at birth in a severe form of individuals with MPS IVA. Skeletal symptoms are found later in childhood or adolescence. The most common symptoms include short stature, skeletal dysplasia, dental anomalies, and corneal clouding (Peracha et al., 2018; Akyol et al., 2019; Sawamoto et al., 2020). There is variable severity, but individuals with the severe phenotype usually do not survive past the second or third decade of life (Montaño et al., 2008).

Primary and permanent posterior teeth are described with concave buccal surfaces with pitting, pointed cusps, and concave occlusal surfaces. The enamel is hypoplastic with rough surface. The color varies from whitish-opaque to more yellowish-grey. Enamel surface is too weak to resist minor stress explaining the strong abrasion. The characteristic teeth color may be explained by the high porosity of enamel changing its optical properties (Rølling et al., 1999).

It is important to know that dental findings are found in MPS IVA, but not in MPS IVB. On radiographs, the enamel is thin but with normal radiodensity (Supplementary Figures S1N, S2S).

#### GALNS

Mucopolysaccharidosis type IVA (MPS IVA; Morquio syndrome A) is an autosomal recessive lysosomal storage disease caused by variants in the galactosamine-6-sulfate sulfatase gene (*GALNS*), located on chromosome 16q24.3. *GALNS* contains 14 exons and 13 introns (Sawamoto et al., 2020).

We report two individuals with compound heterozygous variations in the *GALNS* gene. Individual 19.1 was previously described by our team (Prasad et al., 2016a).

In individual 19.2, we found a missense variant NM_000512.5:c.1156C>T; p.(Arg386Cys) firstly described by (Ogawa et al., 1995), the second variant c.1558T>C; p.(Trp520Arg) was a class 2 variant so we cannot conclude that this variant is indeed involved in the individual phenotype as it is inherited by her homozygous unaffected mother (Table 2). Nevertheless, individual 19.2 presents the typical enzymatic deficiency and was given a confirmed MPS clinical diagnosis. So, either this class 2 variant contributes to the phenotype, or the individual is carrying another variant in this gene not detected by the panel such as a deep intronic variation. Such deep intronic variants have already been described in *GALNS* (Caciotti et al., 2018).

##### Autoimmune polyglandular syndrome type I #240300 autoimmune polyendocrine syndrome, type I, with or without Reversible Metaphyseal dysplasia—AD, AR—*AIRE*

Autoimmune polyglandular syndrome type I (APS-1) is a rare, autosomal recessive autoimmune disease.

The main symptom triad in APS-1 comprises chronic mucocutaneous candidiasis, adrenal insufficiency, and hypoparathyroidism. Various autoimmune diseases and ectodermal abnormalities are also commonly associated with the syndrome including enamel hypoplasia in permanent teeth (Suh et al., 2019). In addition to enamel defects in permanent teeth, hypoplastic pits and hypomature patches in deciduous teeth with underlying changes in the prismatic enamel ultrastructure are observed. The enamel looks severely hypoplastic throughout, except for the most cervical region. Deciduous teeth display opacities and yellowish cervical patches, suggestive of enamel hypomaturation. They are chalky with yellowish patches. The microstructure of the enamel prisms suggests an impaired mineralization, and prisms are clearly different. It was found that, in APS-1, auto-antibodies attack ameloblasts amongst other cells types, making it the first known disease of dental hard tissues with an auto-immune aetiology (Pavlic and Waltimo-Sirén, 2009).

#### AIRE

The disease has been associated to variations in the autoimmune regulator gene (*AIRE*) which consists of 14 exons (Nagamine et al., 1997) and is located on chromosome 21q22 (Aaltonen et al., 1997). AIRE protein is localized in the cell nucleus, where it forms distinct speckles (Björses et al., 1999). Analysis of its multidomain structure reveals that human AIRE belongs to the group of proteins able to bind to chromatin and regulate the process of gene transcription (Perniola, 2018). No variant in this gene were found in our cohort.

##### Tuberous sclerosis *# 605284*—AD—*TSC1-2*

Tuberous sclerosis complex (TSC) is characterized by frequent neuropsychiatric disorders including, in a variable way, intellectual disability, attention-deficit/hyperactivity disorders, autism spectrum disorders (ASD), psychiatric disorders and learning difficulties, seizures, the development of benign tumors, and oral manifestations. The most common oral manifestations are fibromas, gingival hyperplasia and enamel hypoplasia. Other less frequent oral findings are a high arched palate, bifid uvula, cleft lip and/or palate, delayed dental eruption and the presence of diastemas. Enamel hypoplasia is present in the permanent dentition of almost all individuals, and is associated with an increased risk of caries. This anomaly typically affects the vestibular surfaces of several teeth (Harutunian et al., 2011).

#### TSC1-TSC2

Individuals with TSC present variants of the *TSC1* and *TSC2* genes, which intervene in cell cycle regulation. The *TSC1* gene (9q34) encodes hamartin, a protein that interacts with tuberin (TSC2) to form a protein complex that inhibits signal transduction to the downstream effectors of the mammalian target of rapamycin (MTOR) (Inoki et al., 2002). The *TSC1* gene consists of 23 exons, of which the last 21 contain coding sequence and the second is alternatively spliced (Slegtenhorst et al., 1997).

The *TSC* gene on chromosome 16 was named *TSC2*. *TSC2* has 41 small exons spanning 45 kb of genomic DNA and encodes a 5.5-kb mRNA (van Bakel et al., 1997). No variants of *TSC1* or *TSC2* were identified in our cohort.

##### EEC syndrome-3 (EEC3) #604292 Rapp-Hodgkin syndrome #129400—AD—*TP63*

Ectrodactyly, ectodermal dysplasia, and cleft lip/palate syndrome 3 EEC3 (#604292 Maas et al., 1996; Celli et al., 1999; Rinne et al., 2006; Kosaki et al., 2008), ankyloblepharon-ectodermal defects, cleft lip/palate syndrome AEC (#106260), Rapp-Hodgkin (#129400), Acro dermatoungual lacrimal tooth syndrome ADULT (#103285), SHFM4 (#605289), Hay-Wells syndrome (#106260), and limb-mammary syndrome (#603543) are autosomal dominant allelic conditions due to mutations in the same gene, namely, *TP63* (15 exons, 3q28) (Yang et al., 1999) encoding tumor protein 63.

In EEC 3, ectodermal defects manifest as sparse and fine hair, dry skin, soft nails and decrease in sweet capacity (Sutton and van Bokhoven, 2010). (Sripathomsawat et al., 2011) also reported individuals with enamel hypoplasia and hypodontia.

Rapp-Hodgkin syndrome (RHS) is characterized by anhidrotic ectodermal dysplasia and cleft lip/palate. The face of the individuals is characteristic. They have narrow nose and small mouth, wiry, slow growing, and uncombable hair, sparse eyelashes and eyebrows, obstructed lacrimal puncta/epiphora, bilateral stenosis of external auditory canals, microsomia, hypodontia, cone-shaped incisors, enamel hypoplasia, and dystrophic nails (Kantaputra et al., 2003). The first individuals described in the literature were: a mother and her son and daughter (Rapp and Hodgkin, 1968) presenting with anhidrotic ectodermal dysplasia, cleft lip, and cleft palate and an unusually narrow and a small mouth.

#### TP63

TP63 plays an important role allowing cells to undergo apoptosis in response to DNA damage (Flores et al., 2002) and is involved in tumor and metastasis suppression (Su et al., 2010). Given its broad expression pattern, we suggest that *TP63* mutation affect the cell differentiation or fate of ameloblasts in development, though we await experimental evidence. Kantaputra et al., 2003 identified a heterozygous missense variant (S545P) in the *TP63* gene in a Thaï teenager presenting all the characteristics of the syndrome. No variant in this gene is reported in this publication.

##### Craniosynostosis 2 # 604757—AD—*MSX2*

Craniosynostosis is the premature fusion of calvarial sutures.

#### MSX2

The gene is located on chromosome 5q34-q35 (Jabs et al., 1993). (Hassan et al., 2004) showed that Msx2 regulates the expression of osteocalcin and therefore is implicated in the control of bone formation. This gene is reported for craniosynostosis in human. Two previous publications (Aïoub et al., 2007; Molla et al., 2010) reported that, in the targeted deletion mouse model *Msx2* ^−/−^, Msx2 was implicated in both isolated enamel dysplasia (regulating amelogenin, enamelin) and syndromic enamel dysplasia (through alterations in cell-cell junctions). To date one duplication of the entire gene *MSX2* has been reported in a syndromic (craniofacial, eye and limb anomalies) individual associated with hypoplastic AI (Plaisancié et al., 2015). No variant in this gene was identified in our cohort.

##### Raine syndrome # 259775—AR—*FAM20C*

Raine syndrome is an autosomal recessive disease characterized by neonatal osteosclerotic bone dysplasia with a poor prognosis and individuals who generally die within the first few weeks of life. The density of all bones is increased and it is especially evident for the skull. The face is dysmorphic with a narrow prominent forehead, proptosis, depressed nasal bridge, and midface hypoplasia. The periosteal bone formation is typical of the disease and extends along the diaphysis of long bones adjacent to areas of cellular soft tissue (Simpson et al., 2007; 2009).

The syndrome was firstly described *postmortem* by (Raine et al., 1989) on a female fetus presenting with microcephaly, exophthalmos, hypoplastic nose and midface, gum hyperplasia, cleft palate, low-set ears, osteosclerosis and hypoplastic lungs. Simpson et al., 2009 reported the first two unrelated individuals who survived during the childhood and showing typical features of the Raine syndrome. Hypoplastic amelogenesis imperfecta was observed in patients surviving childhood (Acevedo et al., 2015).

#### FAM20C

(Simpson et al., 2007) identified homozygous and compound heterozygous variants in *FAM20C* (7p22) in individuals with Raine syndrome. FAM20C is a Golgi associated secreted protein kinase, partnering with FAM20A, phosphorylating small integrin-binding ligand N-linked glycoproteins SIBLINGS, among other proteins, and playing a substancial role in osteogenesis and amelogenesis. No variant in this gene was identified in our cohort.

##### Focal dermal hypoplasia #305600—XLD—*PORCN*

Focal dermal hypoplasia (FDH) also named Golz or Golz-Gorlin syndrome is an X-linked dominant syndrome. FDH features include atrophy and linear pigmentation of the skin, herniation of fat through the dermal defects, and multiple papillomas of the mucous membranes or skin. In addition, digital anomalies consist of syndactyly, polydactyly, camptodactyly, and oligodactyly, ridged dysplastic nails, alopecia (scalp, eyebrow, and eyelashes). Ocular anomalies (coloboma of iris and choroid, strabismus, microphthalmia) have also been present in some cases. Intellectual disability occurs in some individuals. Striated trabecular bones (osteopathia striata) are a constant feature (Larrègue and Duterque, 1975; Happle and Lenz, 1977; Alsharif et al., 2018; Frisk et al., 2018). There is considerable diversity in the severity of the craniofacial and oral manifestations between individuals. The face is asymmetric, the nose displays hypoplastic alae and the philtrum looks flat in some cases. More severely affected individuals show thin lips with very little vermillion. Dental manifestations are the most commonly observed oral findings, presenting widely spaced teeth, including hypoplastic teeth, showing both hypoplasia and yellow brown hypomineralized areas of enamel. The developmental dental defects vary and include notching of the incisal edges of teeth and marked mamelons, localized hypoplastic vertical enamel groves, and hypodontia (Wright et al., 2016).

#### PORCN

Most of the individuals with FDH are female, with heterozygous or mosaic variants in the *PORCN* gene. Males (mosaic variants) account for 10% of affected individuals; heterozygous non-mosaic variants are lethal in males (Deidrick et al., 2016).

*PORCN* gene is located in chromosome Xp11.23, and contains 15 exons and spans about 12 kb. The first exon is non-coding (Caricasole et al., 2002). *PORCN* encodes and endoplasmic reticulum protein: the protein-serine O-palmitoleoyl transferase porcupine or porcupine O-acyltransferase. Although the exact function of the PORCN protein is uncertain, proteins in the porcupine (PORC) family are involved in WNT (wingless and int homologue) signaling pathway which is extremely important for embryonic development (Durmaz et al., 2018) including odontogenesis. No variant in this gene was identified in our cohort.

##### MLS syndrome with associated hypoplastic enamel X—ARHGAP6

Microphthalmia with linear skin defects (MLS) syndrome is an X-linked disorder that has been linked to different loci. One such condition includes associated AI in a hypoplastic form.

#### ARHGAP6

Because the Amelogenin gene (*AMELX*) is nested within intron 1 of *ARHGAP6,* partial deletions in *ARHGAP6* can completely remove *AMELX* giving a distinctive enamel phenotype resembling “snow-capped” teeth (Hu J. C.-C. et al., 2012). This phenotype was already described in isolated hypomature AI IIC section.

Ras homologue GTPase activation protein 6 (*ARHGAP6*), belongs to the Rho GTPase-activating protein (RhoGAP) family. *ARHGAP6* does not appear essential for normal enamel formation. Failed *ARHGAP6* expression did not appreciably alter the severity of enamel defects when *AMELX* was absent (Hu J. C.-C. et al., 2012).

We report one individual (20.1) carrying a 12 Mb deletion of the Xp22.2 region (Table 2). This region is including not only the *AMELX* gene but also the full *ARHGAP6* gene.

This female individual suffers from microphthalmia with linear skin defects (MLS) syndrome and associated hypoplastic enamel with a banding pattern characteristic of the female lyonisation effect (bands of normal enamel next to defective enamel; AI hypoplastic IE) (Supplementary Figure S1N).

##### Smith-Magenis syndrome # 182290—AD—*RAI1*

Smith-Magenis syndrome is an autosomal dominant disease which occurs mostly *de novo*. Clinical phenotype includes moderate intellectual deficiency with frequent behavioral issues (temper tantrums, nail yanking, insertion of foreign objects into body orifices, self-injurious behaviors), sleep disturbance, dysmorphic facial features. Affected patients can present with variable congenital anomalies (heart defects, structural renal anomalies, scoliosis) including oral anomalies like AI or dental agenesis (Vieira et al., 2012).

#### RAI1

The syndrome is in 90% of the cases due to a 3.7 Mb deletion in chromosome 17p11.2 encompassing the *RAI1* gene (Vieira et al., 2012).

We identified with the GenoDENT panel, in individual 21.1, a deletion in the 17p11.2 region leading to a possible diagnosis of Smith-Magenis syndrome with hypoplastic AI (Table 2). The exact size of the deletion was further characterized by array analysis. Individual 21.1 presented the classical 3.7 Mb deletion associated with the syndrome as well as classical associated phenotype including the hypoplastic enamel described by (Wright et al., 2015).

##### Loeys-Dietz syndrome 2 # 610168—AD—*TGFBR2*

Loeys-Dietz syndrome type 2 is characterized by micrognathia, retrognathia, hypertelorism, bifid uvula, cardiovascular anomalies, pectus deformity, joint laxity, scoliosis, hand and feet anomalies, skin texture and color anomalies and hypoplastic AI (Jani et al., 2020).

#### TGFBR2

Variants in *TGFBR2* have been associated with Loeys Dietz syndrome type 2. TGFBR2 codes for a transmembrane protein with a serine/threonine-kinase domain forming a heterodimeric complex with TGF-beta type I receptor, TGFBR1, binding TGF-beta, TGF-beta1, TGF-beta2 and TGF-beta3, ligands. This receptor/ligand complex phosphorylates proteins, which then enter the nucleus and regulate the transcription of genes related to cell proliferation, cell cycle arrest, wound healing, immunosuppression, and tumorigenesis.

Here we report an individual 22.1 with a heterozygous missense variant in the *TGFBR2* NM_003242.6:c.1561T>C; p.(Trp521Arg) (Table 2; Supplementary Figures S1O, S3.43) and diagnosed with Loeys-Dietz syndrome including hypoplastic amelogenesis imperfecta. This variation was already reported by (Mátyás et al., 2006). The variant is most probably transmitted by the affected mother though we were not able to access the mother’s DNA to confirm.

##### Developmental and epileptic encephalopathy 93 #618012—AD—*ATP6V1A*

Developmental and epileptic encephalopathy 93 is an autosomal dominant disorder with, among other features, delayed psychomotor and impaired intellectual developments, as well as early onset epilepsy. Additional clinical features like microcephaly and hypoplastic amelogenesis imperfecta were observed in epileptic encephalopathy (Guerrini et al., 2022).

#### ATP6V1A

This disorder has been associated with *ATP6V1A* (ATPase, H + Transporting, Lysosomal, 70-KD, V1 Subunit A). It codes for a component of vacuolar ATPase, a multimeric enzyme, an ATP-dependent protein pump function, which mediates acidification of eukaryotic intracellular organelles and is necessary to activate mTORC1 (Zoncu et al., 2011). No variant in this gene was found among our cohort.

##### Hypomagnesemia 3, renal # 248250—AR—*CLDN16*

Familial hypomagnesemia with hypercalciuria and nephrocalcinosis is an autosomal recessive progressive renal disorder with progressive loss of the renal function characterized by excessive urinary Ca^2+^ and Mg^2+^ excretion (Müller et al., 2006). Hypoplastic, hypomature amelogenesis imperfecta is also detected in some individuals (Bardet et al., 2016).

#### CLDN16

(Simon et al., 1999) identified homozygous and compound heterozygous variants in the *CLDN16* gene in ten individuals presenting renal hypomagnesemia. CLDN16 is localized on chromosome 3q28, and consists of 5 exons and encodes a protein of 305 AA with 4 transmembrane domains and intracellular N and C termini (Simon et al., 1999). *CLDN16* is required for cell division (Kittler et al., 2004), is selectively expressed at tight junctions of renal epithelial cells and plays a central role in the reabsorption of divalent cations (Kausalya et al., 2006). No variant in this gene was identified among our cohort.

##### Renal Hypomagnesemia-5 with ocular involvement # 248190—AR—*CLDN19*

Renal hypomagnesemia-5 with ocular involvement (HOMG5) is an autosomal recessive disorder characterized by severe renal magnesium wasting, progressive renal failure, nephrocalcinosis, and severe visual impairment (Konrad et al., 2006). Hypoplastic, hypomature amelogenesis imperfecta is also present in some individuals (Yamaguti et al., 2017).

#### CLD19

Konrad et al., 2006 found two different homozygous missense variants in the *CLDN19* gene in families with renal magnesium wasting, renal failure, and severe ocular involvement. Indeed, the syndrome is caused by homozygous or compound heterozygous variants in the claudin-19 gene on chromosome 3q28. Claudins, such as CLDN19, are transmembrane proteins found in tight junctions. Tight junctions form barriers that control the passage of ions and molecules across an epithelial sheet and the movement of proteins and lipids between apical and basolateral domains of epithelial cells (Lee et al., 2006). No variant in this gene was identified among our cohort.

##### Heimler syndrome #234580 and #616617—AR—*PEX1* and *PEX6*

Heimler syndrome-1 (HMLR1) is an autosomal recessive syndrome reported as the mildest form of the peroxisomal biogenesis disorder spectrum (PBD 1A (Zellweger)). This disease is characterized by sensorineural hearing loss, enamel hypoplasia of the permanent dentition and nail abnormalities with Beau lines (transverse ridges) of the toenails and white patches in the fingernails (leukonychia) (Heimler et al., 1991; Pollak et al., 2003), with or without retinal dystrophy (Ratbi et al., 2015; Mechaussier et al., 2020). In contrast to individuals with PBDs at the severe end of the clinical spectrum (neurologic dysfunction, craniofacial abnormalities, liver dysfunction, and biochemically absence of peroxisomes), Heimler affected individuals showed no identifiable dysmorphic or additional neurologic features.

#### PEX1

Ratbi et al. (2015) identified homozygous and compound heterozygous variants in the peroxisome biogenesis factor 1 gene (*PEX1*) in 4 families, including the family reported by (Heimler et al., 1991). Human PEX1 gene is located on chromosome 7q21, and is composed of 24 exons and encodes a 147-kD protein member of the AAA protein family (ATPases associated with diverse cellular activities). Those proteins participate in a broad range of cellular processes, as indicated by the designation AAA which comes from ATPases associated with diverse cellular activities and are specially required for peroxisomal matrix protein import (Portsteffen et al., 1997; Reuber et al., 1997). No variant in this gene was identified among our cohort.

#### PEX6

Another form of Heimler syndrome (HMLR2; #616617) is caused by a variant in the *PEX6* gene (601,498) located on chromosome 6p21, consisting of 17 exons and 16 introns, spanning about 14 kb (Zhang et al., 1999). No variant in this gene was identified among our cohort.

##### Peroxisome biogenesis disorder PBD 7A (Zellweger) #614872 7B #614873—AR—*PEX26*

It has been shown by (Neuhaus et al., 2017) that *PEX26* is responsible for Zellweger Syndrome. Patient carrying recessive variant present hypoplastic AI (Kim et al., 2021a). No variant in this gene is reported in this publication.

##### Developmental and epileptic encephalopathy 25, with AI #615905—AR—*SLC13A5*

Individuals with developmental and epileptic encephalopathy 25 present epileptic seizures since the first days of life in most of the cases, and a developmental outcome ranging from mild to severe intellectual disability, plus variable combinations and degrees of ataxia, and in addition, teeth with hypoplastic enamel (Hardies et al., 2015). Several individuals have been reported suffering from early onset, regular and difficult to control seizures. In some cases, seizures could be controlled with conventional antiepileptic treatment but showed deterioration of gait which improved after usage of another antiepileptic medication.

Oral manifestations encompass delayed eruption of permanent teeth, small and cylindrical teeth with wide interdental spaces, and yellowish to brownish discolorations. Lower permanent incisors are described as sharp and thin; premolars and molars seem extremely worn. Clinically, the enamel of primary and permanent teeth is hypoplastic with a smooth, hard and pitted surface. Dental panoramic radiographs show a lack of enamel in both dentitions. Histologically, the enamel layer is very thin. No lines of Retzius or enamel prisms are visible and the surface presents small pits. These pits are a common sign for hypoplastic AI. The dentin presents a normal structure (Schossig et al., 2017).

#### SLC13A5

Developmental and epileptic encephalopathy 25 with amelogenesis imperfecta is a rare disease caused by variants in *SLC13A5* genes*. SLC13A5* is located in the chromosome 17p13-p12, and contains at least 12 exons (Inoue et al., 2002). *SLC13A5* encodes a high affinity sodium-dependent citrate transporter, which is mainly expressed in liver and brain. Neurons are considered incapable of *de novo* synthesis of tricarboxylic acid cycle intermediates; therefore, they rely on the uptake of intermediates, such as citrate, to maintain their energy status and neurotransmitter production (Hardies et al., 2015).

This epilepic encephalopathy was described as Kolschütter-Tönz like syndrome (KTS like) as the phenotype is similar to the one observed in individuals with variations in the *ROGDI* gene except for the enamel defects: AI is hypoplastic in patient with *SLC13A5* variations and hypomature in individuals with *ROGDI* mutations. Kolschütter-Tönz syndrome will be described later in the paper as individuals present a hypomature AI.

We report one individual 23.1 carrying a compound heterozygous missense variants affecting exons 2 and 4 NM_177550.5:c.203C>A; p.(Pro68Gln), c.434C>A; p.(Thr145Lys) showing a hypoplastic AI with thin opaque enamel (Table 2; Supplementary Figures S1O, S2T). The *SLC13A5* individual in our panel, is an individual described with *SLC13A5* variant discovered through GenoDENT panel after the identification of the gene by WES in another family of the Schossig et al., 2017 cohort (Schossig et al., 2017) with a phenotype described as Kohlschütter-Tonz like syndrome (KTS).

##### Syndromic hypomature AI

Hypomature AI is also manifest in syndromic conditions. Three genes have thus far been identified as causing syndromes with associated hypomature AI.

##### Immunodeficiency 9 # 612782—AR—*ORAI1*

Primary immunodeficiency-9 (IMD9) is a recessive disease characterized by early onset of recurrent infections due to defective T-cell activation. The individuals present congenital myopathy resulting in muscle weakness, and features of ectodermal dysplasia including a hypomature amelogenesis imperfecta with soft dental enamel (McCarl et al., 2009).

#### ORAI1

By analyzing individuals described by (Feske et al., 1996), (Feske, 2010) showed for the first time that individuals with this type of immune dysfunction were homozygous for a variant in the *ORAI1* gene. Indeed, the disease is caused by homozygous or compound heterozygous variants in *ORAI1* (610,277). This gene located on chromosome 12q24, encodes a subunit of the plasma membrane calcium channel CRAC essential for store-operated calcium entry (Vig et al., 2006) and the channel function. The gene is expressed in cells and organs involved in immunity (CD4^+^ and CD8^+^ T-cells, CD19^+^ B-cells, and in a subset of cells in the thymus, spleen, and tonsils) but also in sarcolemma of muscle fibers, eccrine sweat glands, skin, vascular endothelium, hepatocytes, lung, and kidney (McCarl et al., 2009) and in ameloblast (Nurbaeva et al., 2015). No variant in this gene was identified among our cohort.

##### Immunodeficiency 10 # 612783—AR—*STIM1*

Immunodeficiency-10 is a primary autosomal recessive immunodeficiency, characterized by recurrent infections due to defective T- and NK-cell function. The individuals also have hypotonia, hypohidrosis and hypomature amelogenesis imperfecta. (Picard et al., 2009; Parry et al., 2016a) reported the disease for the first time in 3 siblings from central Europe who had recurrent infection due to defective T-cells, muscle hypotonia and enamel defects. They identified defects in cellular store-operated calcium entry, which is required for lymphocyte activation.

#### STIM1

Immunodeficiency 10 is due to a homozygous truncating variant in the *STIM1* gene (Picard et al., 2009; Parry et al., 2016a). STIM1 gene is located to chromosome 11p15.5 and contains 12 exons (Parker et al., 1996; Sabbioni et al., 1999)*.* It encodes a 746 AA calcium sensor that conveys the calcium load of the endoplasmic reticulum to store-operated channels at the plasma membrane (Yuan et al., 2007) and mediate the gating of CRAC channels (McNally et al., 2012). ORAI1 and STIM1 are interacting as the ORAI1 membrane calcium channel is activated by the calcium sensor STIM1 when calcium reservoirs are depleted (Lacruz and Feske, 2015). AI, related to Immunodeficiency 10, is classified as hypomineralized on OMIM but due to recent publications we transfer it to hypomature AI section (Wang S. et al., 2014; Furukawa et al., 2017). No variant in this gene was identified among our cohort.

##### Kohlschütter-Tonz syndrome #226750—AR—*ROGDI*

This autosomal recessive syndrome was firstly described by (Kohlschütter et al., 1974) in a family of central Switzerland in which 5 brothers were affected but with no mention of the gene involved. Kohlschütter-Tonz syndrome (KTS) is characterized by severe global developmental delay, seizures, and AI affecting both primary and permanent teeth. The teeth present a yellow to brown discoloration. The most severely affected individuals have profound intellectual disability, never acquire speech, and become bedridden early in life (Mory et al., 2012; Schossig et al., 2012).

#### ROGDI

(Schossig et al., 2012) reported for the first time the causative gene by identifying homozygous or compound heterozygous variants in ROGDI gene in 3 members of 3 unrelated families with KTS. ROGDI maps to chromosome 16p13.3., contains 11 exons and spans over 5.98 kb. It encodes a leucine-zipper protein with high expression in the human brain and spinal cord (Mory et al., 2012).

Both variants in *ROGDI* and *SLC13A5* cause epileptic encephalopathy and AI. Whereas *ROGDI* mutation manifests in an hypomature-hypomineralized AI with a rough colored dental surface and with seizure onset may be as late as age 3, individuals mutated for *SLC13A5* present hypoplastic AI, a smooth the dental surface sometimes with only mild discoloration and seizure onset is within the first days after birth.

Here we report three individuals with stop variants in *ROGDI* (24.1, 24.2, 24.3) (Table 2; Supplementary Figures S1P, S2U). Individuals 24.1 and 24.3 present compound heterozygous variants (Supplementary Figures S3.44) already described in the literature, while 27.2 has a homozygous variant found with our panel and previously published in (Huckert et al., 2014).

##### Syndromic hypomineralized AI

Two syndromic conditions associated with hypomineralized AI have been described.

##### Short stature, amelogenesis imperfecta, and skeletal dysplasia with scoliosis #618363—AR—*SLC10A7*

Affected individuals present with short stature, joints dislocation, advanced carpal ossification, abnormal vertebrae, hyperlordosis or kyphoscoliosis, small epiphyses and hypomineralized AI. Enamel has a yellow-brown appearance with a rough surface. Tooth crowns are short and widely spaced. Variable features include facial dysmorphism, moderate hearing impairment, and mildly impaired intellectual development. The phenotype severity is variable. Indeed, an individual with a milder phenotype was reported in (Laugel-Haushalter et al., 2019).

#### SLC10A7

This autosomal recessive syndrome was firstly described by (Dubail et al., 2018) in 6 unrelated individuals. It is due to variants in the *SLC10A7* gene encoding a calcium transporter.

The key phenotype to distinguish individuals with mutation in *SLC10A7*, within the wide spectrum of skeletal dysplasia, was the hypomineralized/hypomature enamel defects observed in all the individuals and the hypoplastic lower jaw.

Here we report 2 individuals with homozygous variants in *SLC10A7*. Individual 25.1 presents a novel homozygous variant in the gene. The variant identified by GenoDENT NGS panel affects exon 3 NM_001300842.3c.269T>G; p.(Leu90Arg) in which another homozygous variant was already described to be pathogenic (Ashikov et al., 2018). Individual 25.2 was already described in (Laugel-Haushalter et al., 2019, WES). She has a homozygous missense variant in the very last exon of the gene c.908C>T; p.(Pro303Leu) (Table 2; Supplementary Figure S3.45) and presents a mild phenotype of the disease but with the characteristic enamel defects (Supplementary Figures S1Q, S2V).

##### Jalili syndrome #217080—AR—*CNNM4*

The autosomal recessive syndrome was first described by (Jalili and Smith, 1988) in 29 individuals and is characterized by cone-rod dystrophy and AI. Nystagmus and photophobia are present from infancy or early childhood and progress with age. Enamel of primary and permanent teeth is hypomineralized (only 50% of mineralization), with a dark brown discoloration, and individuals are more susceptible to dental caries (Parry et al., 2009).

#### CNNM4

The disorder is caused by homozygous or compound heterozygous variants in *CNNM4* gene (607,805) sitting on chromosome 2q11.2. More than 24 different variants have been identified on individuals all around the world but the molecular mechanism of the disease remains unclear (Daneshmandpour et al., 2019). CNNM4 encodes a deduced 775 amino-acids protein. (Guo et al., 2005) hypothesized that the protein may have a role in metal ion transport and homeostasis. Indeed, (Yamazaki et al., 2013) showed in mice the role of Cnnm4 in Mg^2^+ transport. The protein is localized in keratocytes, in the retina, and in developing teeth specifically in ameloblasts (Parry et al., 2009; Polok et al., 2009).

Here we report two individuals with homozygous missense variants in this gene. In individual 26.1 we detected a homozygous missense variant, firstly described by (Parry et al., 2009): NM_020184.4:c.1495G>A; p.(Val499Met) (Table 2; Supplementary Figure S3.46). Other missense variants, were reported as pathogenic and causative of Jalili syndrome. Individual phenotype was consistent with the literature and described clinical synopsis. The “hypoplastic” enamel appearance of both primary and permanent teeth was due to extensive post-eruptive loss of soft enamel detaching easily from dentin. The teeth displayed yellow-brownish coloration with brown spots (Supplementary Figures S1R, S2W). Individual 26.2 has already been reported in (Prasad et al., 2016a) c.1495G>A; p.(Val499Met).

##### Syndromic hypoplastic/hypomature with taurodontism AI

One syndromic condition with hypoplastic/hypomature AI with taurodontism has been identified.

##### Tricho-dento-osseus syndrome #190320—AD—*DLX3*

Tricho-dento-osseous (TDO) syndrome is a rare autosomal dominant condition characterized by various dental and non-dental findings (Jagtap et al., 2019) (Duverger et al., 2017).

TDO emcompasses abnormal development of ectoderm derived structures. Patients presents with head and neck and skeletal phenotypes. Dysplastic nails, curly hair, abnormal density of bone, taurodontism, and hypoplastic amelogenesis imperfecta are common features of this disorder. The enamel appears extremely thin, with enlarged pulp chambers, and root furcations displaced apically. Mandibular prognathism, delayed teeth eruption, teeth discoloration, periapical abscesses, apically positioned furcation, shortened roots, other non-dental abnormalities are variably present. Dental and non-dental features are variable even among affected individuals in the same family (Jain et al., 2017). The management of TDO individuals require a multidisciplinary approach involving both dentists and physicians. Periodic radiographic follow-up is required to prevent further complications such as osteomyelitis (Jagtap et al., 2019).

#### DLX3

TDO is caused by variants in a transcriptional regulator, Distal-less homeobox 3 gene, *DLX3*.

Here we describe two unrelated individuals, one girl (27.1) and one boy (27.2) presenting with tricho-dento-osseous syndrome (Supplementary Figure S1S). They are both carrying the same *DLX3* heterozygous frameshift variant in exon 3 NM_005220.3:c.561_562del; p.(Tyr188Glnfs*13) (Table 2; Supplementary Figures 3.47 and 3.48). This variant firstly described by (Dong et al., 2005) is altering the two amino-acids of the DNA-binding homeodomain and truncating the protein by 88 amino-acids.

## Discussion

AI classification has always been evolving since the one proposed by (Weinmann et al., 1945). These early classifications were based mostly on detailed phenotypic observations (Darling, 1956; Witkop, 1957). Later it was recognized that the mode of inheritance was important to classified AI so, (Schulze, 1970) and (Witkop, 1971) proposed a classification encompassing the phenotype observation and the mode of inheritance. This classification system has been updated and improved since 1988 by (Witkop, 1988) and is currently the mostly frequently used classification demonstrating its extraordinary accuracy grounded on expert clinical skills. A classification based not only on the phenotype and the mode of inheritance but also on the genetic molecular defects was proposed since 1995 by (Aldred and Crawford, 1995), (Hart et al., 2002a) and (Aldred et al., 2003) but this was never fully achieved due to the lack of knowledge and technologies to complete both a clinical and a molecular diagnosis. In 2007 (Crawford et al., 2007) stated that laboratory genetic diagnosis was at that time only a research tool.

It is however now commonly accepted that the mode of inheritance and underlying genomic change are important to improve genetic counselling of affected individuals and their families.

Here we propose a Witkop’s classification evolution including the phenotypical observations (hypoplastic, hypomature, hypomineralized and hypoplastic-hypomature with taurodontism), the mode of inheritance and the genetic diagnosis.

This is now possible as progresses in next-generation sequencing techniques, their availability and now reduced costs have opened the door to personalized oral medicine. GenoDENT NGS panel, exploring 567 genes involved in orodental development and diseases, was set up in research (Prasad et al., 2016a) and transferred in hospital diagnostic laboratories in 2019 (Rey et al., 2019). Its 60% diagnostic rate testify of its reliability and utility in the context of diagnosis, counselling and evolution of treatment options.

It took more than 10 years to gather an informative AI cohort (221 individuals from 111 families) with detailed clinical information (D[4]/phenodent).

Since the discovery of the first gene underlying an amelogenesis imperfecta from mapping of *AMELX* in 1989 (Lau et al., 1989) to its causative role in AI in 1990 (Lagerström et al., 1990; 1991); more than 70 genes have been discovered as important for amelogenesis and its defects. The most recent ones are *CLAUDIN 10* (Sewerin et al., 2022) and *ATP6V1A* (Guerrini et al., 2022)*.* Knowledge is evolving fast on amelogenesis (Simmer et al., 2021) and enamel disturbances in rare diseases. (de La Dure-Molla et al., 2019) listed among 408 rare diseases with orodental manifestations, 105 conditions with enamel defects either isolated (21) or syndromic (84). In our cohort, 73% were diagnosed with non-syndromic amelogenesis imperfecta and 27% with syndromic amelogenesis imperfecta.

The boundaries between isolated and syndromic forms of AI are shrinking as novel information on genes, role of the proteins and associated symptoms and diseases are evolving. This was well illustrated by *FAM20A* and the recognition of enamel-renal syndrome (ERS); enamel-renal-gingival syndrome, hypoplastic amelogenesis imperfecta with nephrocalcinosis, amelogenesis imperfecta and gingival fibromatosis syndrome as allelic conditions and the subsequent transition from an isolated AI to a syndromic disease requiring a different holistic care. *WDR72* was identified in 2009 (El-Sayed et al., 2009). It was recently associated to distal renal tubular acidosis (Khandelwal et al., 2021) and this finding rapidly confirmed in 2019 that isolated *WDR72* associated hypomature AI was in fact a possible syndromic condition (Zhang et al., 2019). AI diagnosis should always bare in mind possible associated symptoms leading to a syndrome diagnosis.

AI clinical subtype recognition can be difficult as phenotype is evolving through time and might transform from a hypomineralized form to a visible “hypoplastic” form thanks to post-eruptive enamel breakdown. The terms hypocalfication and hypomaturation were used by Witkop (1988) before knowing the genes or mechanisms involved in amelogenesis and the pathogenesis of AI. It is now known from the timing of protein expression that these terms are not accurate and both the hypocalcified and hypomaturation phenotypes have as primary feature hypomineralization. *MMP20,* for example, is expressed during the secretory stage and continues to be expressed during the maturation stage yet is classified as hypomature AI and it is hypomineralized.

Clinically “hypocalcified” refers to softer enamel with post eruptive breakdown and “hypomature” to less mineralized but strong enough enamel preserving the teeth morphology therefore corresponding to a more advanced maturation process and the state of degradation of enamel matrix proteins.

The Witkop’s classification terminology could be adjusted to use only names (I HYPOPLASIA, II HYPOMATURATION, III HYPOMINERALIZATION *versus* HYPOCALCIFICATION, IV HYPOMATURATION/HYPOPLASIA with TAURODONTISM) or only adjectives (hypoplastic, hypomature, hypomineralized, hypomature/hypoplastic). May be sections II and III of the classification should be united in a single hypomineralization section.

This paper gathers in the Supplementary Figures many clinical intraoral pictures and panoramic radiographs of genotype related amelogenesis imperfecta. This resource is meant to help clinicians improving their AI diagnosis and search for associated symptoms.

Dental anomalies and enamel defects are very precise key diagnostic clues (Boch-Zupan et al., 2012; de La Dure-Molla et al., 2019) helping, when recognized, to orientate a clinical diagnosis towards a broader genetic rare disease recognition. These AI phenotypes can be precise diagnostic signatures. Among them the clinical features linked to ERS (almost no enamel, impacted teeth, intrapulpal calcifications, root anomalies … ) would immediately suggest a possible ERS and orientate the clinical team towards kidney investigations *via* ultrasound seeking nephrocalcinosis. In Heimler syndrome, the AI is only present in the permanent dentition and its recognition linked to seusorineural hearing loss could suggest the diagnosis.

Following genetic diagnosis, retro-phenotyping can also lead to the search and recognition of additional traits and the diagnosis of broader rare diseases. For example, the diagnosis of Jalili syndrome or amelogenesis imperfecta and dystrophy of the cones and rods of the retina was subsequently made in a 4-year-old boy who attended a rare disease competence center for enamel problems management. The genetic diagnosis pointed to the presence of autosomal bi–allelic recessive variants in the *CNNM4* gene with further confirmation of the presence of a retinal dystrophy. This transformed an isolated enamel restricted diagnosis to a broader rare disease identification.

Genotype recognition can change a clinical diagnosis: two patients with 2 different diagnoses: spondyloepiphyseal dysplasia and mucopolysaccharidosis type 4A (ORPHA: 309,297) were investigated for associated hypoplastic amelogenesis imperfecta (quantitative enamel defects). The results of the GenoDENT test showed variants in the *GALNS* gene responsible for mucopolysaccharidosis type 4A. The test changed the medical diagnosis for one of the patients. A revised diagnosis facilitates access to other treatments and care.

Phenotype/genotype identification can also lead to accurate information and genetic counselling, guide therapeutic management and facilitate the discovery of new genes and diseases.

Patients with negative results on the panel were further explored with exome sequencing and through international collaborations and larger cohort gathering new genes such as *LTBP3* (Huckert et al., 2015), *SLC13A5* (Schossig et al., 2017), *SLC10A7* (Laugel-Haushalter et al., 2019) were identified. Whole genome sequencing (WGS), as the PFMG 2025 initative (https://pfmg2025.aviesan.fr/en/; rare diseases with orodental manifestations https://pfmg2025.aviesan.fr/professionnels/preindications-et-mise-en-place/formes-syndromiques-de-maladies-rares-a-expression-bucco-dentaire/) could facilitate the discovery of the underlying genetic defects causing both non-syndromic and syndromic AI.

In this cohort we identified 151 variants. Among these, 124 were classified as likely pathogenic or pathogenic (class 4 or 5) and 47 were newly reported. It is interesting to notice that the most frequent genes identified in isolated AI were *AMELX* in hypoplastic, *MMP20* in hypomature and *FAM83H* in hypomineralized AI and *LTBP3* and *FAM20A* in syndromic conditions.

Our results allowed to provide for 81% of the index individuals a definitive genetic diagnosis, and for 19%, variants of unknown significance (VUS) were identified. Twenty-one new VUS were detected in patients with isolated AI and 3 new VUS in patients with syndromic AI.

However, for those uncertain variants, like new candidate genes, or for variants in different domains of the protein or with different possible physiopathological mechanisms, it could be difficult to confirm their pathogenicity and these variants are subsequently classified as variant of unknow significance (VUS).

To reclassify those variants and provide a clear genetic diagnostic it is important to develop reliable, easy to perform *in vitro* assays and functionally validate these variants. Furthermore, functional characterization will open new potential strategies for curative treatments.

Enamel defects have also been reported in others syndromes and the question remains to qualify them as amelogenesis imperfecta. For instance, genes associated with - skin, nails and hair defects among other symptoms are *ATR* (Tanaka et al., 2012), *CLDN1* (Feldmeyer et al., 2006), *COG6* (Shaheen et al., 2013), *FGF10, FGFR3, FGFR2* (Hollister et al., 1973), *HRAS* (Goodwin et al., 2014), *KRAS, NRAS, KRT14* (Tabata et al., 1996), *MBTPS2* (Martino et al., 1992); - with eye defects *NAA10*; - with skeletal anomalies *AKT1, B3GAT3, CYP27B1, CTSK, EVC1, EVC2, ERCC4, ERCC8, GJA1, GNAS, IDUA, IRX5, NDN, PTDSS1, SNORD116, RUNX2, TBCE, VDR*; - genito-urinary anomalies *HNF1B, VPS33B, VIPAR*; - intellectual disability *PSPA, GALC*; Usher syndrome *MYO7A, USH2A, PD2D7, ADGRV1, CLRN1*.

Amelogenesis is at the crossroad of many developmental processes and careful examination of the oral cavity of syndromic patients should be mandatory to deliver appropriate preventive care and follow up targeting oral health.

It is crucial that the team of health professionals involved in diagnosing and managing a possibly syndromic patient knows the value of an expert examination of the oral cavity and the importance of an acute diagnosis of these developmental defects assisting syndrome diagnosis (Bloch-Zupan et al., 2021). On the other hand, it is important that the dentist who can recognize abnormal teeth can convey the right information towards the medical team. Expert reference rare diseases reference centres can assist patients and their treating practitioners in diseases diagnosis and management according to evidence-based information.

Undergraduate, postgraduate and continuous education is important to ensure best management options for rare diseases patients.

Gathering data from large cohorts and pooling information from registries should also lead to a better understanding of the prevalence of AI as a whole or the various AI types and rare diseases. The prevalence stated in the literature from 1:700 to 1:14,000, according to the populations studied may not reflect reality. These data are of importance to facilitate financial undertaking and reimbursement by health authorities of comprehensive lifelong treatments.

Further actions are also needed to update International Classification of Diseases (ICD), The Systematized Nomenclature in Medicine (SNOMED), Orphanet and ontologies (HPO, Orphanet … ) and to develop guidelines (https://www.has-sante.fr/jcms/p_3284538/fr/20amelogeneses-imparfaites) to ensure precision, personalized oral medicine and its dedication to treatment of individuals suffering from amelogenesis imperfecta.

Witkop’s classification was and is still remarkable. It serves as a good basis to understand the nature of enamel defects and as a guide towards its revision as knowledge on genetics and pathophysiology is increasing. JT Wright (2023) in a recent paper discussed these issues and concluded upon the opportunity, thanks to advanced genetics, to “elaborating a more accurate and informative nosology for these conditions in order to improve communication between patients, families, clinicians and researchers”.

The revised classification presented here, developed thanks to GenoDENT NGS panel, will hopefully provide a useful tool for accelerating genotype/phenotype causal relations and improved patient outcomes.

## Data Availability

The variants were submitted in ClinVar (https://www.ncbi.nlm.nih.gov/clinvar/), a freely available, public archive of human genetic variants and interpretations of their significance to disease, maintained at the National Institutes of Health (Landrum et al, 2018). Their accession numbers are: SCV003843192, SCV003843249, SCV003843250, SCV003843251, SCV003843254, SCV003843255, SCV003843256, SCV003843257, SCV003843258, SCV003843259, SCV003843870, SCV003843871, SCV003843872, SCV003843873, SCV003843875, SCV003843877, SCV003843878, SCV003843879, SCV003843881, SCV003843887, SCV003843888, SCV003843252, SCV003843247, SCV003843883, SCV003843886, SCV003842949, SCV003843193, SCV003842321, SCV003842323, SCV003842272, SCV003842271, SCV003842273, SCV003842270, SCV003842952, SCV003842312, SCV003842315, SCV003842322, SCV003842325, SCV003842326, SCV003842947, SCV003842948, SCV003842953, SCV003842954, SCV003842955, SCV003842957, SCV003843190, SCV003843191.

## Group members of Consortia

### O-Rare Consortium

O-Rares Consortium: French Reference and Competence centers, healthcare network for rare oral and dental diseases CRMR/CCMR O-Rares, French Filière “TETECOU” and associated paediatric/genetic teams (Centres de Génétique et Centres de Référence Anomalies du Développement et Syndromes Malformatifs, Filière AnDDI Rares). Edouard Euvrard, Juliette Piard (Centre Hospitalier Universitaire de Besançon; Hôpital Jean Minjoz); Marie José Boileau, Elsa Garot, Didier Lacombe (CHU de Bordeaux—Hôpital Pellegrin); Victorin Ahossi, Laurence Faivre (CHU Dijon, Hôpital François Mitterrand); Caroline Delfosse (CHU de Lille); Klauss Dieterich, Service de génétique Hôpital Couple Enfant, Grenoble; Jean-Pierre Duprez, Jean-Jacques Morrier, Béatrice Thivichon-Prince, Béatrice Richard, Nathalie Guffon-Fouilhoux, Linda Pons, Massimiliano Rossi (Hospices Civils de Lyon, Groupement Centre, Pôle d’Activités Médicales d’Odontologie, Centre de soins Dentaires; Service de génétique, Centre de Référence Anomalies du Développement); Corinne Tardieu, Isabelle Blanchet (APHM, Hôpital de la Timone Enfants, Marseille); Dominique Droz, Magali Hernandez, Bruno Leheup (CHRU de Nancy—Hôpitaux de Brabois); Serena Lopez, Bertand Isidor (CHU de Nantes—Hôtel Dieu); Michèle Muller- Bolla, Clara Joseph-Beaudin (CHU de Nice); Muriel de La Dure Molla, Benjamin Fournier, Ariane Berdal (Hôpital Rothschild, APHP, Paris); Alain Verloes (AP-HP, Hôpital Robert Debré, Paris); Sandrine Marlin (AP-HP, Hôpital Necker Enfants Malades, Paris); Tiphaine Davit-Béal (AP-HP, Paris and CHU de Rennes); Marie Paule Gelle (CHU Reims, Hôpital Maison Blanche, Pôle: Odontologie); Bérénice Doray (CHU de la Réunion Site SUD (Terre Sainte)); Jean-Louis Sixou, Alinoe Lavillaurex, Sylvie Odent (CHU Rennes); Marine Lebrun (CHU Saint-Etienne); Agnès Bloch-Zupan, Isaac Maximiliano Bugueno, François Clauss, Jean-Christophe Dahlet, Jean-Luc Davideau, Olivier Etienne, Bruno Grollemund, Mathilde Huckert, Sophie Jung, Marzena Kawczynski, Adeline Loing, Marie-Cécile Manière, Prune Sadones, Marion Strub, Delphine Wagner, Yves Alembik, Salima El Chehadeh, Hélène Dollfus, Elise Schaefer, Sylvie Soskin, Anne De-Saint-Martin (Hôpitaux Universitaires de Strasbourg); Isabelle Bailleul-Forestier, Emmanuelle Noirrit, Frédéric Vaysse, Nicolas Chassaing (CHU de Toulouse—Hôpital Rangueil); Annick Toutain (CHU de Tours).

### European reference network, ERN CRANIO, Consortium

Tatjana Dostalova, Milan Macek Jr, University Hospital Motol and 2nd Faculty of Medicine, Charles University, Prague, Czeck Republic.

### International Consortium

Mustapha El Alloussi, Faculty of Dentistry, International University of Rabat,CReSS Centre de recherche en Sciences de la Santé, Morocco; Leeila Qebibo, Unité de génétique médicale et d’oncogénétique, CHU Hassan II, Fez, Morocco; Supawich Morkmued, Patimaporn Pungchanchaikul, Faculty of Dentistry, Khon Kaen University, Khon Kaen, Thailand; Nicole Revencu, Centre de Génétique humaine UCL Cliniques universitaires Saint-Luc, Bruxelles, Belgium; Blanca Urzúa Orellana, Instituto de Investigación en Ciencias Odontológicas, Facultad de Odontología, Universidad de Chile, Chile; Juliane Leonhardt Amar, Switzerland; Zouari Noura, Service de Pédiatrie, Hôpital universitaire Sahloul, Sousse Tunisie.

## Supplementary material

Supplementary Figure S1 Clinical intraoral photographs and panoramic radiographs detailing the phenotype of amelogenesis imperfecta patient for each associated genotype. Isolated AI: A COL17A1; B COL7A1; C LAMA3, LAMB3, LAMC2; D ENAM; E AMELX, F AMBN, G ACP4, H KLK4, MMP20; I WDR72, C4ORF26/ODAPH and SLC24A4; J FAM83H; K DLX3; Syndromic AI: L LTBP3; M FAM20A; N GALNS 2700 and ARHGAP6; O TGFBR2 and SLC13A5; P ROGDI; Q SLC10A7; R CNNM4; S DLX3. **Isolated AI**. **A *COL17A1*** Hypoplastic pitted amelogenesis imperfecta. Patients present pitted rough enamel with normal hardness. Some patients present a yellowish discoloration with brown extrinsic stains within the pits. Radiographs show normal density but sometimes thinner enamel. Patients 1.1, 1.3, and 1.4 were already described in Prasad et al., 2016 **B *COL7A1*** Hypoplastic amelogenesis imperfecta. Pitted, and thin enamel with yellow-brownish discoloration. Spaced teeth, and incisal and occlusal attrition reflect thinner enamel. **C *LAMA3, LAMB3 and LAMC2*** Hypoplastic amelogenesis imperfecta. Patient 3.1 presents a digenic mutation affecting both COL17A1 and LAMA3 genes. Enamel displays pits arranged in rows and columns. Comparing with COL17A1 mutations related phenotype, the enamel seems more pitted. Both primary and permanent dentition are affected. LAMB3 Patients 4.1 and 4.3 are 8-year-old. Enamel presents pits, and yellowish discoloration more pronounced in incisors. In the radiographs, enamel shows a normal density however unerupted teeth in 4.2, 4.3, 4.4 are clearly dysplastic. Patient 5.1 of 4-year-old, presents a mutation in LAMC2 showing a primary dentition with thin white enamel. Patient 3.1 and 4.1 were already described in Prasad et al. (2016). **D *ENAM*** Hypoplastic autosomal dominant amelogenesis imperfecta. Thin enamel with white spots, and yellowish discoloration in some cases. In some cases, tooth size might be reduced, and teeth appear slim. Enamel pits, and linear depressions are visible. Patient 6.3 presents agenesis of 16, 26, 36, 46, and stainless-steel crowns covering primary lower molars, with an hypoplastic/hypomature AI. Hypoplastic enamel is visible also on non-erupted teeth in panoramic radiographs. **E *AMELX*** Hypoplastic amelogenesis imperfecta displaying in female individuals a banding pattern (Lyonisation effect) (patients 7.1, 7.3, 7.4, 7.8, 7.11), with vertically ridged teeth, alternating bands of normal and hypoplastic enamel mainly on incisors and spaced yellowish teeth smaller than normal. Male patients (7.2, 7.10) phenotype is more severe with almost no enamel. Patient 7.10 presents a delayed dental eruption. 7.9 is a male displaying a banding pattern AI phenotype similar to females. Further exploration established 7.9 with a XXY karyotype. Radiographs show a thinner with normal density enamel in patients 7.3, 7.6, 7.7, 7.7. In patients 7.2, 7.5, 7.10 there is no obvious contrast between enamel and dentin but enamel is very thin. Hypomature amelogenesis imperfecta (7.5, 7.6, 7.7): Patient 7.7 has agenesis of 12, 15, 22, 28 caused by an additional mutation in WNT10A gene. **F *AMBN*** Hypoplastic amelogenesis imperfecta. Affects both primary and permanent dentition. Patients present rough irregular pitted enamel. The enamel surface acquires a yellowish color with age. Patient 8.1 is 10- year-old, and presents an associated open bite. Individual 8.2 has an end-to-end bite. Patient 8.3 was already described in Prasad et al. (2016). He presents restorations on upper and lower incisors, and first molars. On radiographs, the enamel is almost absent. **G *ACP4*** Hypoplastic amelogenesis imperfecta. Thin enamel with irregular surface, and yellowish discoloration. Patient 9.2 has composite crowns on all her teeth, suggesting a generalized AI. Patient 9.3 presents agenesis of 18, 28 due to a mutation in WNT10A gene. X-rays show a thinner but normal density enamel, long teeth and slim roots. **H *KLK4 and MMP20*** Hypomature amelogenesis imperfecta. Patient 10.1 present a mutation in KLK4 gene showing molars with soft brown enamel, with a lack of radio-contrast between enamel and dentin. Opaque, smooth, and pigmented enamel with incisal and occlusal attrition in primary and permanent dentition is visible in patients with MMP20 variants. i.e., in patient 11.6 and 11.11 both dentition are affected. Thinner enamel than normal detaches easily from the dentin. Patient 11.2 was already published in Prasad et al. (2016) and presents taurodontism. Patients 11.5, 11.6, 11.9, 11.11, were published in Gasse et al. (2017). Patient 11.8 presents stainless steel crowns in molars with pronounced mammelons in permanent incisors. In X-rays, lack of contrast between enamel and dentin is clearly visible. **I *WDR72, C4ORF26/ODAPH, and SLC24A4*** Hypomature amelogenesis imperfecta. Yellow-brownish soft enamel with a mottled appearance that detaches easily from the dentin. Both primary and permanent teeth are affected. Radiographs show a lack of contrast between enamel and dentin. 12.1-4 (WDR72). 13.1 ODAPH. Individual 14.1 (SLC24A4) had primary molars with stainless steel crowns, and an agenesis of 45. Patient 14.2 presented an impacted 13, all permanent incisors were covered by composite veneers, and stainless-steel crowns protected the first lower molars, hypomature enamel is clearly present on other teeth. Radiographs show a lack of contrast between enamel and dentin. Patient 12.2, 13.1 and 14.1 were already published in Prasad et al., 2016. **J *FAM83H*** Hypomineralized amelogenesis imperfecta. Primary and permanent dentitions are affected with soft, colored enamel that detaches easily from the dentin (post eruptive breakdown). Spaced teeth with occlusal and incisal wear giving a conic form in canines are related to secondary enamel loss. Patients complain from high sensitivity. Patients 15.4, 15.6 present slight taurodontism as seen on x-ray. Patients 15.3, 15.5, 15.10 and 15.14 have stainless steel pediatric crowns on primary molars. 15.10, 15.12, 15.13, 15.14 present a clear open bite. Radiographs show no difference in radioopacity between enamel and dentin. **K *DLX3*** Hypoplastic—Hypomature amelogenesis imperfecta with taurodontism. 16.1 has a rough enamel surface, with white and brown spots. A stria as an hypoplastic defect is visible on lower incisal edges. Slight taurodontism is present. 16.2 showed very thin enamel and marked molar taurodontism. 37 is missing. Teeth colour is very “dentin” like as enamel is so thin. 16.3 presented with an hypoplastic clearly hypomature AI with taurodontism (16, 26, 36, 46 and even primary molars), white spots, and brown extrinsic stains. Dental radiographs show normal radiolucency of enamel even if thinner. **Syndromic AI L *LTBP3* (Verloes Bourguignon syndrome, Platyspondyly with AI)** Hypoplastic amelogenesis imperfecta. There is almost no enamel. Delayed teeth eruption. Large pulp chambers, Small, and spaced teeth, with a yellowish color. **M *FAM20A* (Enamel Renal Syndrome)** Hypoplastic amelogenesis imperfecta. There is almost no enamel. Eruption delay and microdontia, with spaced teeth, showing a yellow discoloration, and reduced enamel thickness affecting both dentitions. Patient 18.8 has a prosthetic rehabilitation on upper incisors. The radiographs analysis shows a delayed and impaired eruption of permanent dentition, ankylosed permanent teeth, hyperplasia of follicular sac, intrapulpal calcifications, root formation anomalies. Gingival hyperplasia is clearly visible. **N *GALNS* (Mucopolysaccharidosis type IV) and *ARHGAP6* (Linear skin defects with multiple congenital anomalies 1)** Hypoplastic amelogenesis imperfecta. Patients 19.1 and 19.2 affected with GALNS mutations have teeth with a rough thin enamel surface. On radiograph enamel is thin but with normal radioopacity. Female individual 20.1 has an AMELX deletion displaying a similar phenotype than the isolated form with a banding pattern of normal and hypoplastic enamel, she is affected by MLS syndrome. **O *TGFBR2* (Loeys-Dietz syndrome) and *SLC13A5* (Kohlschutter-Tonz like syndrome)** Hypoplastic amelogenesis imperfect. Patient 22.1 has a TGFBR2 mutation, teeth have a rough surface with pits, the enamel color is opaque yellowish. Panoramic radiograph shows normal enamel radiodensity. In patient 23.1 enamel is thin with opaque yellowish colour, and brown extrinsic stains. SLC13A5 mutation was already reported in Schossig et al. (2017). **P *ROGDI* (Kohlschutter-Tonz syndrome)** Hypomature amelogenesis imperfecta. Individuals present a yellow-brownish discoloration of enamel affecting both primary and permanent dentitions. Stainless steel crowns are covering the molars in patients 24.1 and 24.3. Patient 24.2 was published in Huckert et al., 2016. **Q *SLC10A7* (Short stature, amelogenesis imperfecta, and skeletal dysplasia with scoliosis)** Hypomature/Hypomineralized amelogenesis imperfecta affecting both the 2809 primary and permanent dentition with a yellowish enamel. Patient 25.2 presents stainless steel crowns on upper and lower molars and was already published in Laugel-Haushalter et al. (2019). The radiographs show similar density between dentin and enamel. **R *CNNM4* (Jallili Syndrome)** Hypomineralized amelogenesis imperfecta. 26.1 Extensive post eruptive breakdown and loss of enamel, yellow-brownish coloration with brown spots. High calculus accumulation on rough enamel surface clearly visible in lower incisors. 26.2 Primary dentition affected by AI, the enamel appears thin as it easily wears off from dentin. Stainless still crowns restorations in molars. **S *DLX3* (Tricho-Dento-Osseus syndrome)** Hypoplastic/hypomature? amelogenesis imperfecta with taurodontism. Patient 27.1 and 27.2 are affected by TDO syndrome. Dental radiographs show stronger radiolucency of thin enamel and taurodontism. Patient 27.2 is 6-year-old.

Supplementary Figure S2 Variants and corresponding protein domain. A gene is composed of exons represented by vertical lines and the initiation codon (ATG) and stop codon (TGA) are visualized by a black arrow. The variations detected in this study are symbolized by an arrow associated with the patient number in bold if homozygous. Green arrows correspond to variations never described before. Blue arrows correspond to variations already published by our team in Prasad et al. 2016. Orange arrows correspond to variations published by our team in collaborative studies. Gray arrows correspond to variations already known. Triangles symbolize splice variants, circle missenses variants, square nonsense variants, oval small deletion, rectangle small insertion, horizontal line bigger deletion. The shapes are coloured in blue when the individual is a male, in pink when the person is a female and purple for a male with suspected sex chromosome abnormalities. The corresponding protein domains are represented according to the PFAM database. The arrows indicate the localization of the detected variant with predictable protein impact. **A *COL17A1* variations and corresponding protein domain.** The gene transcript NM_000494.4 is made of 56 exons represented by vertical lines. **B *COL7A1* variations and corresponding protein domain.** The gene transcript NM_000094.4 is composed of 119 exons represented by vertical lines. **C *LAMA3* variations and corresponding protein domain. The** gene transcript NM_198129.4 is composed of 75 exons represented by vertical lines. **D *LAMB3* variations and corresponding protein domain.** The gene transcript NM_000228.3 is composed of 23 exons represented by vertical lines.**E *LAMC2* variations and corresponding protein domain.** The gene transcript NM_005562.3 is composed of 23 exons represented by vertical lines.**F *ENAM* variations and corresponding protein domain.** The gene transcript NM_031889.3 is composed of 9 exons represented by vertical lines. **G *AMELX* variations and corresponding protein domain.** The gene transcript NM_182680.1 is compoed of 7 exons represented by vertical lines. **H *AMBN* variations and corresponding protein domain** The gene transcript NM_016519.6 is composed of 13 exons represented by vertical lines.**I *ACP4* variations and corresponding protein domain.** The gene transcript NM_033068.3 is composed of 11 exons represented by vertical lines.**J *KLK4* variations and corresponding protein domain.** The gene transcript NM_004917.4 is composed of 5 exons represented by vertical lines. **K *MMP20* variations and corresponding protein domain.** The gene transcript NM_004771.4 is composed of 10 exons represented by vertical lines. **L *WDR72* variations and corresponding protein domain.** The gene transcript NM_182758.4 is composed of 20 exons represented by vertical lines. **M *ODAPH* variations and corresponding protein domain.** The gene transcript NM_178497.5 is composed of 2 exons represented by vertical lines. **N *SLC24A4* variations and corresponding protein domain.** The gene transcript NM_153646.4 is composed of 17 exons represented by vertical lines.**O *FAM83H* variations and corresponding protein domain.** The gene transcript NM_198488.5 is composed of 5 exons represented by vertical lines.**P *DLX3* variations and corresponding protein domain.** The gene transcript NM_005220.3 is composed of 3 exons represented by vertical lines.**Q *LTBP3* variations and corresponding protein domain.** The gene transcript NM_001130144.3 is composedd of 28 exons represented by vertical lines.**R *FAM20A* variations and corresponding protein domain.** The gene transcript NM_017565.4 is composed of 11 exons represented by vertical lines.**S *GALNS* variations and corresponding protein domain.** The gene transcript NM_000512.5 is composed of 14 exons represented by vertical lines.**T *SLC13A5* variations and corresponding protein domain.** The gene transcript NM_177550.5 is composed of 12 exons represented by vertical lines.**U *ROGDI* variations and corresponding protein domain.** The gene transcript NM_024589.3 is composedd of 11 exons represented by vertical lines.**V *SLC10A7* variations and corresponding protein domain.** The gene transcript NM_001300842.3 is composed of 13 exons represented by vertical lines.**W *CNNM4* variations and corresponding protein domain.** The gene transcript NM_020184.4 is composed of 7 exons represented by vertical lines.

Supplementary Figure S3 Familial segregation of variants by Sanger sequencing (GRCh37). 1. Variant segregation in individual’s 1.2 family. Individual 1.2 (I.1) is heterozygous for the variant NM_000494.4(*COL17A1*): c.1745-2A>C; p.?. Her affected sister and her two affected daughters are also heterozygous c.1745-2A>C. 2. Variant segregation in individual’s 1.6 family. Individual 1.6 (II.1) is heterozygous for the variant NM_000494.4(*COL17A1*): c.3327del; p.(Pro1110Argfs*21). The variant is inherited from his mother. Phenotype of both parents was not available. 3. Variant segregation in individual’s 2.2 family. Individual 2.2 (II.4) and her affected brothers (II.1 and II.3) are heterozygous for the variant NM_000094.4(*COL7A1*): c.3605G>A; p.(Arg1202His). Parental DNA were not available. 4. Variant segregation in individual’s 2.3 family. Individual 2.3 (II.1) is heterozygous for the variant NM_000094.4(*COL7A1*): c.3785T>C; p.(Met1262Thr). The variant is inherited from his affected mother (I.2). Father (I.1) DNA and phenotype are not available. 5.Variant segregation in individual’s 4.4 family. Individual 4.4 (II.1) is heterozygous for the variant NM_000228.3(*LAMB3*): c.2926del; p.(Val976Trpfs*54). The variant is inherited from his heterozygous father (I.1). 6. Variant segregation in individual’s 4.1 family. Individual 4.1 (II.1) is compound heterozygous for the variant NM_000228.3(*LAMB3*): c.124C>T; p.(Arg42*) and c.3490C>T; p.(Arg1164Cys). 7. Variant segregation in individual’s 4.2 family. Individual 4.2 (II.1) is compound heterozygous for the variant NM_000228.3(*LAMB3*): c.1288+1G>T; p.? and c.1903C>T; p.(Arg635*). 8. Variant segregation in individual’s 5.1 family. Individual 5.1 (II.1) is heterozygous for the variant NM_005562.3(*LAMC2*): c.493C>T; p.(Arg165Cys). The variant is inherited from her mother (I.2). Parental phenotype is not available. 9. Variant segregation in individual’s 6.3 family. Individual 6.3 (II.1), her affected sister (II.2) and affected father (I.1) are all heterozygous for the variant NM_031889.3(*ENAM*): c.588+1dup; p.?.10. Variant segregation in individual’s 6.6 family. Individual 6.6 (II.1) is heterozygous for the variant NM_031889.3(*ENAM*): c.588+1del; p.?. Father (I.1) DNA and phenotype are unavailable (NA). 11. Variant segregation in individual’s 6.7 family. Individual 6.7 (II.1), his affected sister (II.3) and mother (I.2) are heterozygous for the variant NM_031889.3(*ENAM*): c.588+1del; p.?. His second non -affected sister (II.2) and his father (I.1) do not carry the variant. 12. Variant segregation in individual’s 6.8 family. Individual 6.8 (II.1), his affected sister (II.2) and mother (I.2) are heterozygous for the variant NM_031889.3(*ENAM*): c.664C>T; p.(Gln222*). His father does not carry the variant and his mother’s phenotype is unavailable. 13. Variant segregation in individual’s 7.4 family. Individual 7.4 (II.1) is heterozygous for the variant NM_182680.1(*AMELX*): c.47C>A; p.(Ala16Asp). The variant is inherited from her affected hemizygous father (I.1). 14. Variant segregation in individual’s 7.8 family. Individual 7.8 (III.1) is heterozygous for the variant NM_182680.1(*AMELX*): c.473del; p.(Pro158Hisfs*31). The variant spread inside affected members of the family. The variant is heterozygous for females II.2 and II.3 and for male III.2 and hemizygous for 7.8’s (III.1) grandfather (I.1). Individual 7.9 (III.2) is a male with a XXY karyotype explaining his heterozygous genotype. 15. Variant segregation in individual’s 7.10 family. Individual 7.10 (II.1) is hemizygous for the variant NM_182680.1(*AMELX*): c.541del; p.(Leu181Cysfs*8). The variant is inherited from his affected heterozygous mother (I.2).16. Variant segregation in individual’s 7.6 family. Individual 7.6 (II.1) and his brother (II.2) are hemizygous for the variant NM_182680.1(*AMELX*): c.208C>A; p.(Pro70Thr). Parental results are not available. 17. Variant segregation in individual’s 7.7 family. Individual 7.7 (II.1) is hemizygous for the variant NM_182680.1(*AMELX*): c.208C>A; p.(Pro70Thr). The variant is inherited from mother (I.2) who are heterozygous as his sister (II.2). Their phenotypes are not available. 18. Variant segregation in individual’s 8.1 family. Individual 8.1 (II.1) is heterozygous for the two variants NM_016519.6(*AMBN*): c.15+1G>A; p? and c.577G>T; p.(Gly193*). Her mother I.2 is heterozygous only for c.15+1G>A and her father (I.1) result are not available. 19. Reads visualization of individual 10.1 (II.1) highlighting two heterozygous compound variants NM_004917.4(*KLK4*) c.443G>T; p.(Cys148Phe) and c.458G>A; p.(Trp153*). Parental DNA was not available. 20. Variant segregation in individual’s 11.1 family. Individual 11.1 (II.1) is heterozygous for the two variants NM_004771.4(*MMP20*): c.103A>C; p.(Arg35=) and c.389C>T; p.(Thr130Ile). The variant c.103A>C is inherited from his heterozygous mother (I.2) and the variant c.389C>T by his heterozygous father (I.1).21. Variant segregation in individual’s 11.3 family. Individual 11.3 (II.1) and his sister (II.2) are compound heterozygous for the two variants NM_004771.4(*MMP20*): c.103A>C; p.(Arg35=) and c.954-2A>T; p.?. His mother (I.2) and brother (II.3) are heterozygous only for c.103A>C. His father (I.1) and second brother (II.1) are heterozygous only for c.954-2A>T.22. Variant segregation in individual’s 11.4 family. Individual 11.4 (II.1) is compound heterozygous for the two variants NM_004771.4(*MMP20*): c.103A>C; p.(Arg35=) and c.1362C>G; p.(Tyr454*). The variant c.103A>C is inherited from his heterozygous mother (I.2) and the variant c.1362C>G from his heterozygous father (I.1).23. Variant segregation in individual’s 11.8 family. Individual 11.8 (II.1) is compound heterozygous for the two variants NM_004771.4(*MMP20*): c.359dup; p.(Asn120Lysfs*9) and c.954-2A>T; p?. The variant c.359dup is inherited from his heterozygous father (I.1) and the variant c.954-2A>T from his heterozygous mother (I.2).24. Variant segregation in individual’s 11.11 family. Individual 11.11 (II.1) is compound heterozygous for the two variants NM_004771.4(*MMP20*): c.530G>A; p.(Gly177Glu) and c.954-2A>T; p?. The variant c.530G>A is inherited from her heterozygous mother (I.2) and the variant c.954-2A>T from her heterozygous father (I.1).25. Variant segregation in individual’s 11.15 family. Individual 11.15 (II.1) and her affected brother (II.2) are homozygous for NM_004771.4(*MMP20*): c.954-2A>T; p?. Their parents (I.1 and I.2) are heterozygous for c.954-2A>T.26. Reads visualization of individual 12.4 (II.1) with IGV using the paired-end sorting option highlighting two heterozygous compound variants NM_182758.4(*WDR72*): c.2146del; p.(Ala716Profs*10) and c.2388del ; p.(Lys796Asnfs*16). Arrow indicates informative read pairs for allelic relative position of the two variants. Parental DNA was not available.27. Variant segregation in individual’s 14.2 family. Individual 14.2 (II.1) and her affected sister (II.2) are homozygous for the variant NM_153646.4(*SLC24A4*): c.1716+5G>A; p.?. Parents’ DNA and phenotype are not available.28. Variant segregation in individual’s 15.1 family. Individual 15.1 (II.1) is heterozygous for the variant NM_198488.5(*FAM83H*): c.930_939dup; p.(Val314Argfs*14). Her non-affected sister (II.2) do not carry the variant. Parents’ DNA and phenotype are not available.29. Variant segregation in individual’s 15.2 family. Individual 15.2 (II.1) is heterozygous de novo for the variant NM_198488.5(*FAM83H*): c.973C>T; p.(Arg325*). Her parents do not carry the variant and SNP filiation controls were verified.30. Variant segregation in individual’s 15.6 family. Individual 15.6 (II.1) is heterozygous for the variant NM_198488.5(*FAM83H*):c.1309_1311delinsTAG; p.(His437*). This variant is inherited from her father (I.1) also heterozygous. 31Variant segregation in individual’s 15.7 family. Individual 15.7 (II.1) is heterozygous de novo for the variant NM_198488.5(*FAM83H*): c.1374C>G; p.(Tyr458*). His parents do not carry the variant and SNP filiation controls were verified.32. Variant segregation in individual’s 15.8 family. Individual 15.8 (II.1) is heterozygous de novo for the variant NM_198488.5(*FAM83H*): c.1374C>G; p.(Tyr458*). His parents do not carry the variant and SNP filiation controls were verified.33. Variant segregation in individual’s 15.9 family. Individual 15.9 (II.1) is heterozygous for the variant NM_198488.5(*FAM83H*): c.1375C>T; p.(Gln459*). The variant is inherited from her father also heterozygous.34. Variant segregation in individual’s 15.10 family. Individual 15.10 (II.1) is heterozygous de novo for the variant NM_198488.5(*FAM83H*): c.1379G>A; p.(Trp460*). Her parents do not carry the variant and SNP filiation controls were verified.35. Variant segregation in individual’s 15.12 family. Individual 15.12 (II.1) and his brother (II.3) are heterozygous for the variant NM_198488.5(*FAM83H*): c.1387C>T; p.(Gln463*). Individual 16.12 (II.1) and his brother (II.3) are homozygous for the variant NM_182758.4(*WDR72*): c.1283T>G; p.(Ile428Ser). This variant is inherited from their mother (I.2) also heterozygous.36. Variant segregation in individual’s 15.13 family. Individual 15.13 (II.1) and her brother (II.3) are heterozygous for the variant NM_198488.5(*FAM83H*): c.1498C>G; p.(Leu500Val). The variant *FAM83H*: c.1498C>G is not inherited from their mother and she is heterozygous for the *WDR72* c.1283T>G. Their father results are not available.37. Variant segregation in individual’s 15.14 family. Individual 15.14 (II.1) is heterozygous for the variant NM_198488.5(*FAM83H*): c.1993C>T; p.(Gln665*). The variant is inherited from her father (I.1).38. Variant segregation in individual’s 17.6 family. Individual 17.6 (II.1) is homozygous for the variant NM_001130144.3(*LTBP3*): c.3629-2A>G; p?. Her parents (I.1 and I.2) are heterozygous for the variant.39. Variant segregation in individual’s 18.3 family. Individual 18.3 (II.1) and his sister (II.2) are compound heterozygous for the variants NM_017565.4(*FAM20A*): c.217C>T; p.(Arg73*) and c.727C>T; p.(Arg243*). The mother (I.2) is heterozygous for the variant c.727C>T and the father (I.1) is heterozygous for the variant c.217C>T.40. Variant segregation in individual’s 18.5 family. Individual 18.5 (II.1) is compound heterozygous for the variants NM_017565.4(*FAM20A*): c.915_918del; p.(Phe305Leufs*76) and c.928+2T>C; p?. Her mother (I.2) is heterozygous for c.928+2T>C and her father is heterozygous for c.915_918del.41. Variant segregation in individual’s 18.6 family. Individual 18.6 (II.1) and his brother (II.2) are compound heterozygous for the variants NM_017565.4(*FAM20A*): c.915_918del; p.(Phe305Leufs*76) and c.1301+5G>A; p?. Their mother (I.2) is heterozygous for c.915_918del and their father is heterozygous for c.1301+5G>A.42. Variant segregation in individual’s 18.9 family. Individual 18.9 (II.1) and his brother (II.2) are homozygous for the variant NM_017565.4(FAM20A): c.1369A>T; p.(Lys457*). Their mother (I.2) is heterozygous for c.1369A>T and their father’s (I.1) result is not available.43. Variant segregation in individual’s 22.1 family. Individual 22.1 (II.1) is heterozygous for the variant NM_003242.6(*TGFBR2*): c.1561T>C; p.(Trp521Arg). His father (I.1) do not carry the variant and his mother’s (I.2) DNA is not available.44. Variant segregation in individual’s 24.3 family. Individual 24.3 (II.1) is compound heterozygous for the variants NM_2024589.2(*ROGDI*): c.366dup; p.(Ala123Serfs*19) and c.366dup; p.(Tyr134*). The variant c.366dup is inherited from her mother (I.2) and the variant c.366dup from her father (I.1).45. Variant segregation in individual’s 25.1 family. Individual 25.1 (II.1) is homozygous for the variant NM_001300842.3(*SLC10A7*): c.269T>G; p.(Leu90Arg). His mother (I.2) and father (I.1) are heterozygous for the variant.46. Variant segregation in individual’s 26.1 family. Individual 26.1 (II.1) and her sister (II.2) are homozygous for the variant NM_2020184.4(*CNNM4*): c.586T>C; p.(Ser196Pro). Her mother (I.2) is heterozygous and her father’s (I.1) DNA is not available.47. Variant segregation in individual’s 27.1 family. Individual 27.1 (II.1), her brother (II.2) and mother (I.2) are heterozygous for the variant NM_005220.3(*DLX3*): c.561_562del; p.(Tyr188Glnfs*13). Her father’s (I.1) DNA is not available.48. Variant segregation in individual’s 27.2 family. Individual 27.2 (II.1) is heterozygous for the variant NM_005220.3(*DLX3*): c.561_562del; p.(Tyr188Glnfs*13). This variant is inherited from his affected heterozygous father (I.1).

Supplementary Table S1 List of gene sequenced in version 6.0 of the GenoDENT panel. Exons and 25 bp in flanking introns of these genes were sequenced using Next Generation Sequencing technics.

Supplementary Table S2 List of primer’s sequences used for Sanger sequencing.

Supplementary Table S3 Variations found in individuals presenting with isolated amelogenesis imperfecta. Variations found in 17 different genes in 85 individuals presenting with isolated amelogenesis imperfecta. One hundred eleven variants were found, 19 variants are of uncertain significance. Variants known and previously published are reported in grey, variants previously reported by the team are represented in salmon, variants published thanks to GenoDENT panel are represented in blue or green, new variants reported for the first time are highlighted in green. Familial segregation is also reported when available and reported as: Family member code (Phenotype code, Genotype code): Fa: father; Mo: mother; S: sibling; D: daughter; So: son; Co: cousin; A: affected; U: unaffected; NA: not available; C: carrier; R: reference genotype.

Supplementary Table S4 Key diagnostic clinical signs associated to AI in syndromes.
