# Supplementary material for: Amelogenesis imperfecta: Next-generation sequencing sheds light on Witkop’s classification
Source: Front Physiol. 2023 May 9;14:1130175. doi: 10.3389/fphys.2023.1130175 (PMC10205041; doi:10.3389/fphys.2023.1130175)
Supplement: Supplementary file 1 [file Table4.pdf]

Supplementary Table S4: Key diagnostic clinical signs associated to AI in syndromes

Clinical signs	Associated to AI	Syndrome - mode of inheritance	Phenotype OMIM number	Gene	AI type
**HEAD & NECK**					
Head	Craniosynostosis	Craniosynostosis 2 - AD	604757	*MSX2*	Hypoplastic
	Micrognathia, Retrognathia	Loeys-Dietz syndrome 2 - AD	610168	*TGFBR2*	Hypoplastic
	Dysmorphic traits	Smith-Magenis syndrome - AD<br>Short stature, and skeletal dysplasia with scoliosis - AR	182290<br>618363	*RAI1*<br>*SLC10A7*	Hypoplastic<br>Hypomineralized
Eye	Hypertelorism, Exotropia	Loeys-Dietz syndrome 2 - AD	610168	*TGFBR2*	Hypoplastic
	Severe visual impairment	Hypomagnesemia 5, renal, with ocular involvement - AR	248190	*CLDN19*	Hypoplastic
	Corneal opacities	Mucopolysaccharidosis IVA - AR	253000	*GALNS*	Hypoplastic
	Myopia	Craniosynostosis 2 - AD	604757	*MSX2*	Hypoplastic
	Cone-rod dystrophy, Nystagmus, Photophobia	Jalili syndrome - AR	217080	*CNNM4*	Hypomineralized
Ears	Sensorineural hearing loss	Heimler syndrome 1 – AR<br>Heimler syndrome 2 - AR<br>Heimler syndrome - AR	234580<br>616617<br>/	*PEX1*<br>*PEX6*<br>*PEX26*	Hypoplastic<br>Hypoplastic<br>Hypoplastic
	Moderate hearing impairment	Short stature, and skeletal dysplasia with scoliosis - AR	618363	*SLC10A7*	Hypomineralized
Orodental	Lip papillomas	Focal dermal hypoplasia - XLD	305600	*PORCN*	Hypoplastic
	Cleft lip	EEC syndrome-3 – AD<br>Raine syndrome - AR	604292<br>259775	*TP63*<br>*FAM20C*	Hypoplastic<br>Hypoplastic
	Cleft palate	EEC syndrome-3 – AD<br>Raine syndrome – AR<br>Short stature, and skeletal dysplasia with scoliosis - AR	604292<br>259775<br>618363	*TP63*<br>*FAM20C*<br>*SLC10A7*	Hypoplastic<br>Hypoplastic<br>Hypomineralized
	Bifid uvula	Loeys-Dietz syndrome 2 - AD	610168	*TGFBR2*	Hypoplastic
	Gingival hyperplasia	Enamel-renal syndrome – AR<br>Raine syndrome - AR	204690<br>259775	*FAM20A*<br>*FAM20C*	Hypoplastic<br>Hypoplastic
	Gingival fibroma	Tuberous sclerosis-1 – AD<br>Tuberous sclerosis-2 - AD	191100<br>613254	*TSC1*<br>*TSC2*	Hypoplastic<br>Hypoplastic
	Tooth agenesis	Dental anomalies and short stature – AR	601216	*LTBP3*	Hypoplastic

		EEC syndrome-3 – AD<br>Developmental and epileptic encephalopathy 25 -AR	604292<br>615905	*TP63*<br>*SLC13A5*	Hypoplastic<br>Hypoplastic
	Teeth eruption delay	Developmental and epileptic encephalopathy 25 -AR	615905	*SLC13A5*	Hypomature
	Microdontia	EEC syndrome-3 - AD	604292	*TP63*	Hypoplastic
	Taurodontism	Dental anomalies and short stature – AR<br>Trichodontoosseous syndrome - AD	601216<br>190320	*LTBP3*<br>*DLX3*	Hypoplastic<br>Hypoplastic/ hypomature
	Widely spaced teeth	Mucopolysaccharidosis IVA - AR	253000	*GALNS*	Hypoplastic
	Caries	Mucopolysaccharidosis IVA – AR<br>EEC syndrome-3 – AD<br>Jalili syndrome - AR	253000<br>604292<br>217080	*GALNS*<br>*TP63*<br>*CNNM4*	Hypoplastic<br>Hypoplastic<br>Hypomineralized
**SKIN, NAILS, & HAIR**					
**Skin**	Melanotic macules, Facial angiofibromas	Tuberous sclerosis-1 – AD<br>Tuberous sclerosis-2 - AD	191100<br>613254	*TSC1*<br>*TSC2*	Hypoplastic<br>Hypoplastic
	Ectodermal dysplasia	EEC syndrome-3 – AD<br>Focal dermal hypoplasia – XLD<br>Immunodeficiency 9 – AR<br>Immunodeficiency 10 - AR	604292<br>305600<br>612782<br>612783	*TP63*<br>*PORCN*<br>*ORAI1*<br>*STIM1*	Hypoplastic<br>Hypoplastic<br>Hypomature<br>Hypomature
	Linear or reticular hyperpigmentation, Localized cutaneous deposits of superficial fat, Arborescent papillomas	Focal dermal hypoplasia - XLD	305600	*PORCN*	Hypoplastic
	Translucent skin	Loeys-Dietz syndrome 2 - AD	610168	*TGFBR2*	Hypoplastic
**Nails**	Nails abnormalities	Heimler syndrome 1 – AR<br>Heimler syndrome 2 - AR<br>Heimler syndrome – AR<br>Trichodontoosseous syndrome - AD	234580<br>616617<br>/<br>190320	*PEX1*<br>*PEX6*<br>*PEX26*<br>*DLX3*	Hypoplastic<br>Hypoplastic<br>Hypoplastic<br>Hypoplastic/ hypomature
**Hair**	Kinky hair	Trichodontoosseous syndrome - AD	190320	*DLX3*	Hypoplastic/ hypomature
**SKELETAL**					
	Short stature	Dental anomalies and short stature – AR<br>Mucopolysaccharidosis IVA - AR	601216<br>253000	*LTBP3*<br>*GALNS*	Hypoplastic<br>Hypoplastic

		Raine syndrome - AR<br>Focal dermal hypoplasia - XLD<br>Short stature, and skeletal dysplasia with scoliosis - AR	259775<br>305600<br>618363	*FAM20C*<br>*PORCN*<br>*SLC10A7*	Hypoplastic<br>Hypoplastic<br>Hypomineralized
	Osteosclerosis	Raine syndrome - AR	259775	*FAM20C*	Hypoplastic
	Brachyolmia	Dental anomalies and short stature – AR	601216	*LTBP3*	Hypoplastic
	Vertebral defects	Dental anomalies and short stature – AR	601216	*LTBP3*	Hypoplastic
	Skeletal dysplasia	Mucopolysaccharidosis IVA – AR<br>Short stature, and skeletal dysplasia with scoliosis – AR<br>Trichodontoosseous syndrome - AD	253000<br>618363<br>190320	*GALNS*<br>*SLC10A7*<br>*DLX3*	Hypoplastic<br>Hypomineralized<br>Hypoplastic
	Split-hand/foot malformation	EEC syndrome-3 – AD<br>Focal dermal hypoplasia - XLD	604292<br>305600	*TP63*<br>*PORCN*	Hypoplastic<br>Hypoplastic
	Joint laxity	Loeys-Dietz syndrome 2 - AD	610168	*TGFBR2*	Hypoplastic
	Scoliosis	Short stature, and skeletal dysplasia with scoliosis - AR	618363	*SLC10A7*	Hypomineralized
**MUSCLE**					
	Muscular hypotonia	Developmental and epileptic encephalopathy 25 -AR<br>Immunodeficiency 9 – AR<br>Immunodeficiency 10 - AR	615905<br>612782<br>612783	*SLC13A5*<br>*ORAI1*<br>*STIM1*	Hypoplastic<br>Hypomature<br>Hypomature
**NEUROLOGIC**					
	Hamartomatous lesions of the brain, Learning difficulties, Autism	Tuberous sclerosis-1 – AD<br>Tuberous sclerosis-2 - AD	191100<br>613254	*TSC1*<br>*TSC2*	Hypoplastic<br>Hypoplastic
	Ataxia, Lack of speech acquisition	Developmental and epileptic encephalopathy 25 -AR<br>Kohlschutter-Tonz syndrome - AR	615905<br>226750	*SLC13A5*<br>*ROGDI*	Hypoplastic<br>Hypomature
	Abnormal involuntary movements	Developmental and epileptic encephalopathy 25 -AR	615905	*SLC13A5*	Hypoplastic
	Spasticity	Developmental and epileptic encephalopathy 25 -AR<br>Kohlschutter-Tonz syndrome - AR	615905<br>226750	*SLC13A5*<br>*ROGDI*	Hypoplastic<br>Hypomature
	Seizures	Tuberous sclerosis-1 – AD<br>Tuberous sclerosis-2 – AD<br>Developmental and epileptic encephalopathy 93 – AD<br>Developmental and epileptic encephalopathy 25 –AR<br>Kohlschutter-Tonz syndrome - AR	191100<br>613254<br>618012<br>615905<br>226750	*TSC1*<br>*TSC2*<br>*ATP6V1A*<br>*SLC13A5*<br>*ROGDI*	Hypoplastic<br>Hypoplastic<br>Hypoplastic<br>Hypoplastic<br>Hypomature
	Intellectual disability	Smith-Magenis syndrome – AD<br>Kohlschutter-Tonz syndrome - AR	182290<br>226750	*RAI1*<br>*ROGDI*	Hypoplastic<br>Hypomature
	Behaviour anomalies, Sleep	Smith-Magenis syndrome - AD	182290	*RAI1*	Hypoplastic

	disturbance				
	Delayed psychomotor development	Developmental and epileptic encephalopathy 93 – AD<br>Developmental and epileptic encephalopathy 25 –AR<br>Kohlschutter-Tonz syndrome - AR	618012<br>615905<br>226750	*ATP6V1*<br>*SLC13A5*<br>*ROGDI*	Hypoplastic<br>Hypoplastic<br>Hypomature
	Impaired intellectual development	Developmental and epileptic encephalopathy 93 – AD<br>Short stature, and skeletal dysplasia with scoliosis - AR	618012<br>618363	*ATP6V1A*<br>*SLC10A7*	Hypoplastic<br>Hypomineralized
**CARDIOVASCULAR**					
	Vascular defect	Dental anomalies and short stature – AR	601216	*LTBP3*	Hypoplastic
	Artery aneurysm, Arterial tortuosity	Loeys-Dietz syndrome 2 - AD	610168	*TGFBR2*	Hypoplastic
**GENITOURINARY**					
	Nephrocalcinosis	Enamel-renal syndrome – AR<br>Raine syndrome – AR<br>Hypomagnesemia 3, renal – AR<br>Hypomagnesemia 5, renal, with ocular involvement – AR<br>Amelogenesis imperfecta, type IIA3 – AR	204690<br>259775<br>248250<br>248190<br>613211	*FAM20A*<br>*FAM20C*<br>*CLDN16*<br>*CLDN19*<br>*WDR72*	Hypoplastic<br>Hypoplastic<br>Hypoplastic<br>Hypoplastic<br>Hypomature
	Renal lesions	Tuberous sclerosis-1 – AD<br>Tuberous sclerosis-2 – AD<br>Hypomagnesemia 3, renal – AR<br>Hypomagnesemia 5, renal, with ocular involvement – AR<br>Smith Magenis	191100<br>613254<br>248250<br>248190<br>182290	*TSC1*<br>*TSC2*<br>*CLDN16*<br>*CLDN19*<br>*RAI1*	Hypoplastic<br>Hypoplastic<br>Hypoplastic<br>Hypoplastic<br>Hypoplastic
**ENDOCRINE FEATURES**					
	Adrenal insufficiency, Hypoparathyroidism	Autoimmune polyendocrinopathy syndrome - AD/AR	240300	*AIRE*	Hypoplastic
**IMMUNOLOGY**					
	Chronic mucocutaneous candidiasis	Autoimmune polyendocrinopathy syndrome - AD/AR	240300	*AIRE*	Hypoplastic
	Recurrent infections	Immunodeficiency 9 – AR<br>Immunodeficiency 10 - AR	612782<br>612783	*ORAI1*<br>*STIM1*	Hypomature<br>Hypomature
**LABORATORY ABNORMALITIES**					
	N-acetylgalactosamine-6-sulfatase deficiency	Mucopolysaccharidosis IVA - AR	253000	*GALNS*	Hypoplastic
	Hypomagnesemia, Hypercalciuria	Hypomagnesemia 3, renal - AR	248250	*CLDN16*	Hypoplastic
