# Supplementary material for: Amelogenesis imperfecta: Next-generation sequencing sheds light on Witkop’s classification
Source: Front Physiol. 2023 May 9;14:1130175. doi: 10.3389/fphys.2023.1130175 (PMC10205041; doi:10.3389/fphys.2023.1130175)
Supplement: Supplementary file 3 [file Presentation1.zip › Supplementary Figure 1.PDF]

Isolated

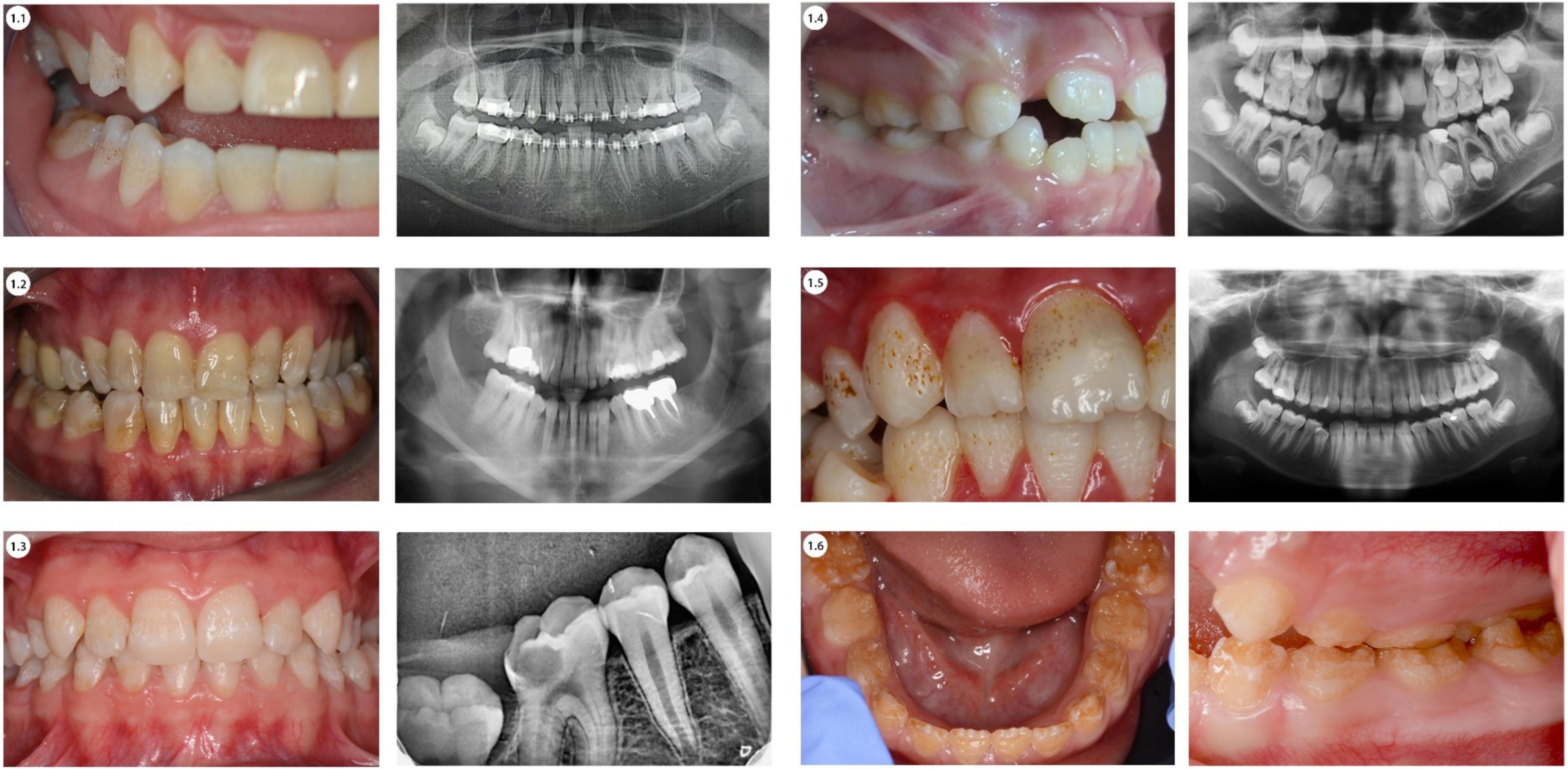

Supplementary Figure 1A *COL17A1*

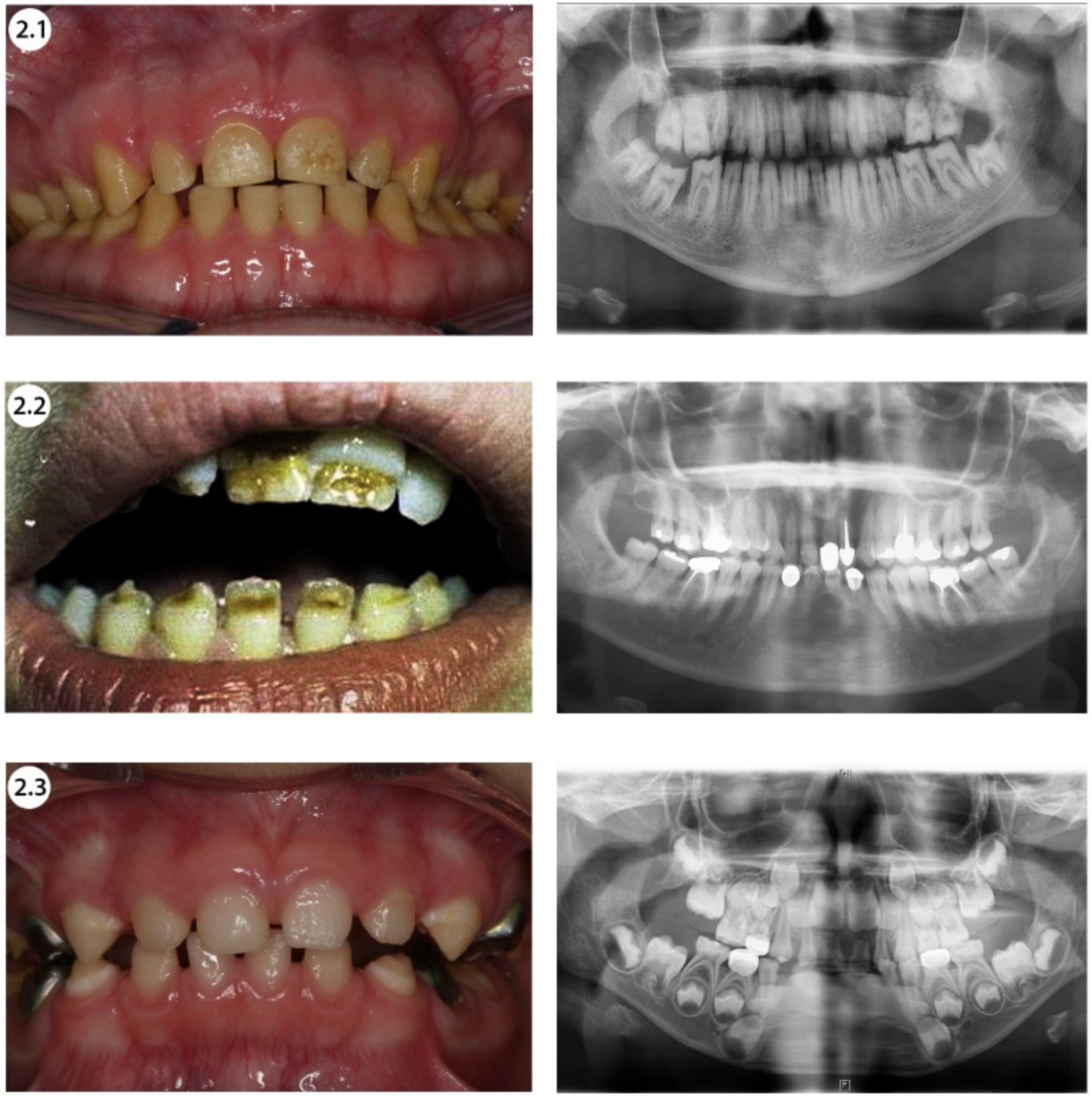

Supplementary Figure 1B *COL7A1*

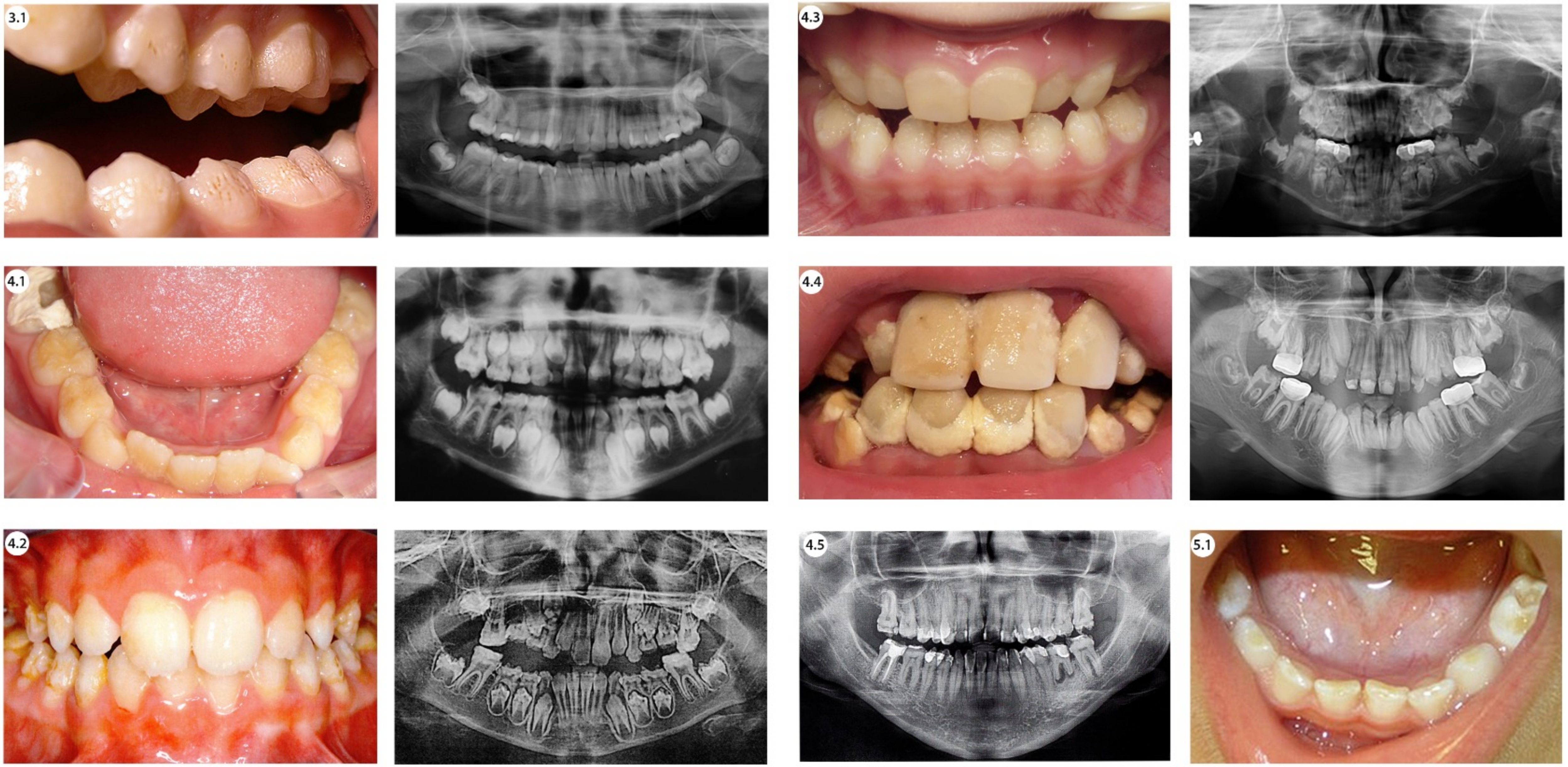

Supplementary Figure 1C ***LAMA3, LAMB3 and LAMC2***

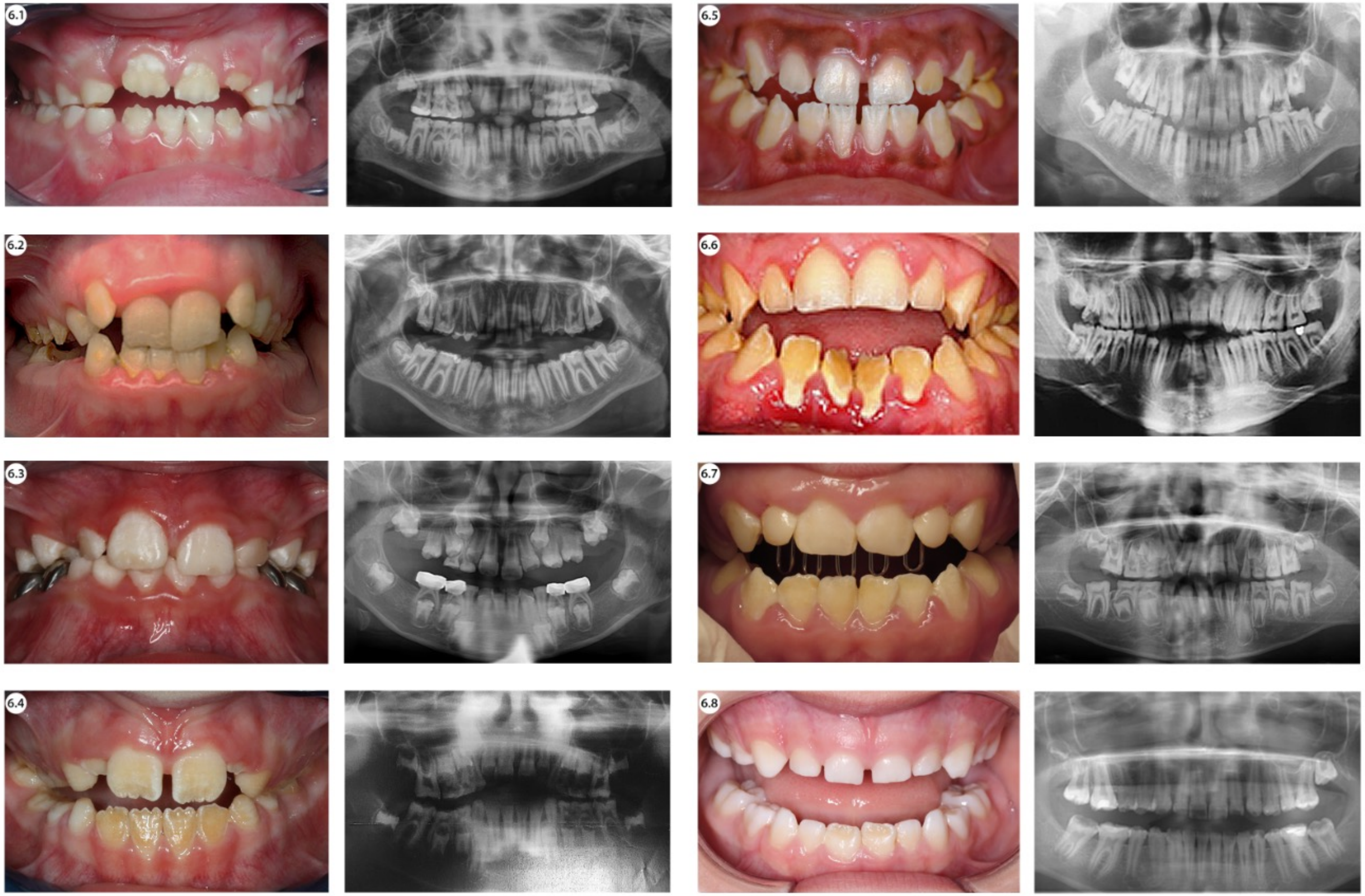

Supplementary Figure 1D ***ENAM***

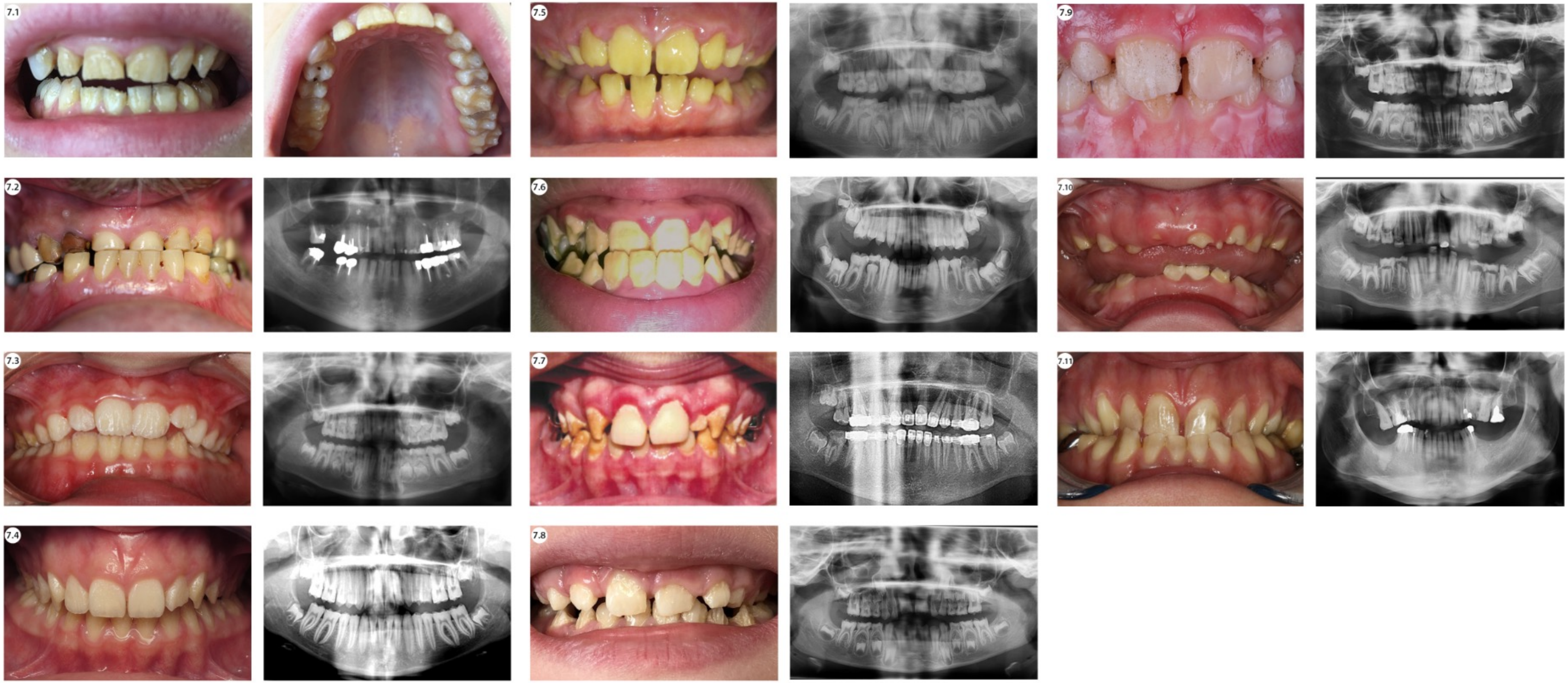

Supplementary Figure 1E ***AMELX***

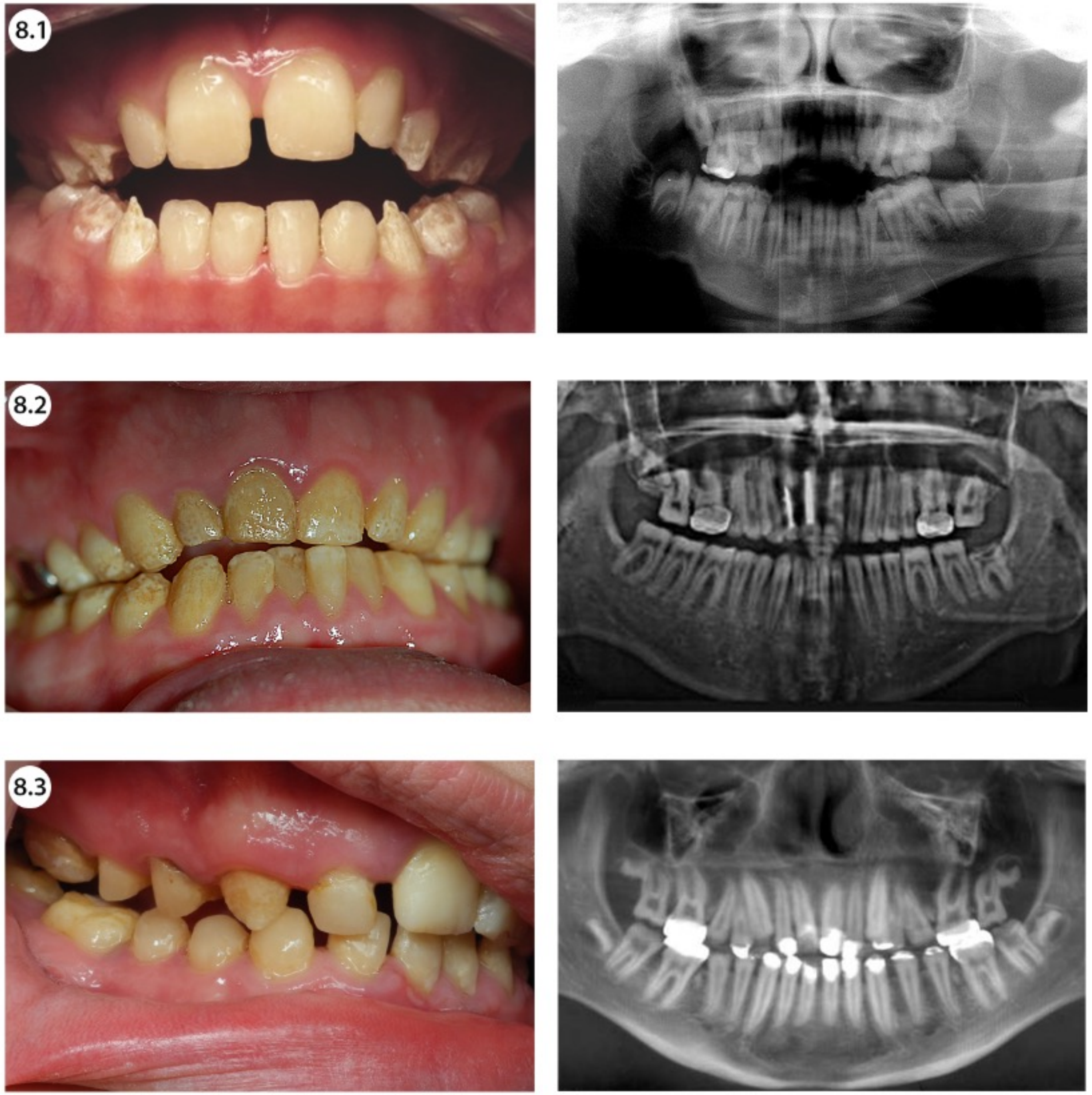

Supplementary Figure 1F *AMBN*

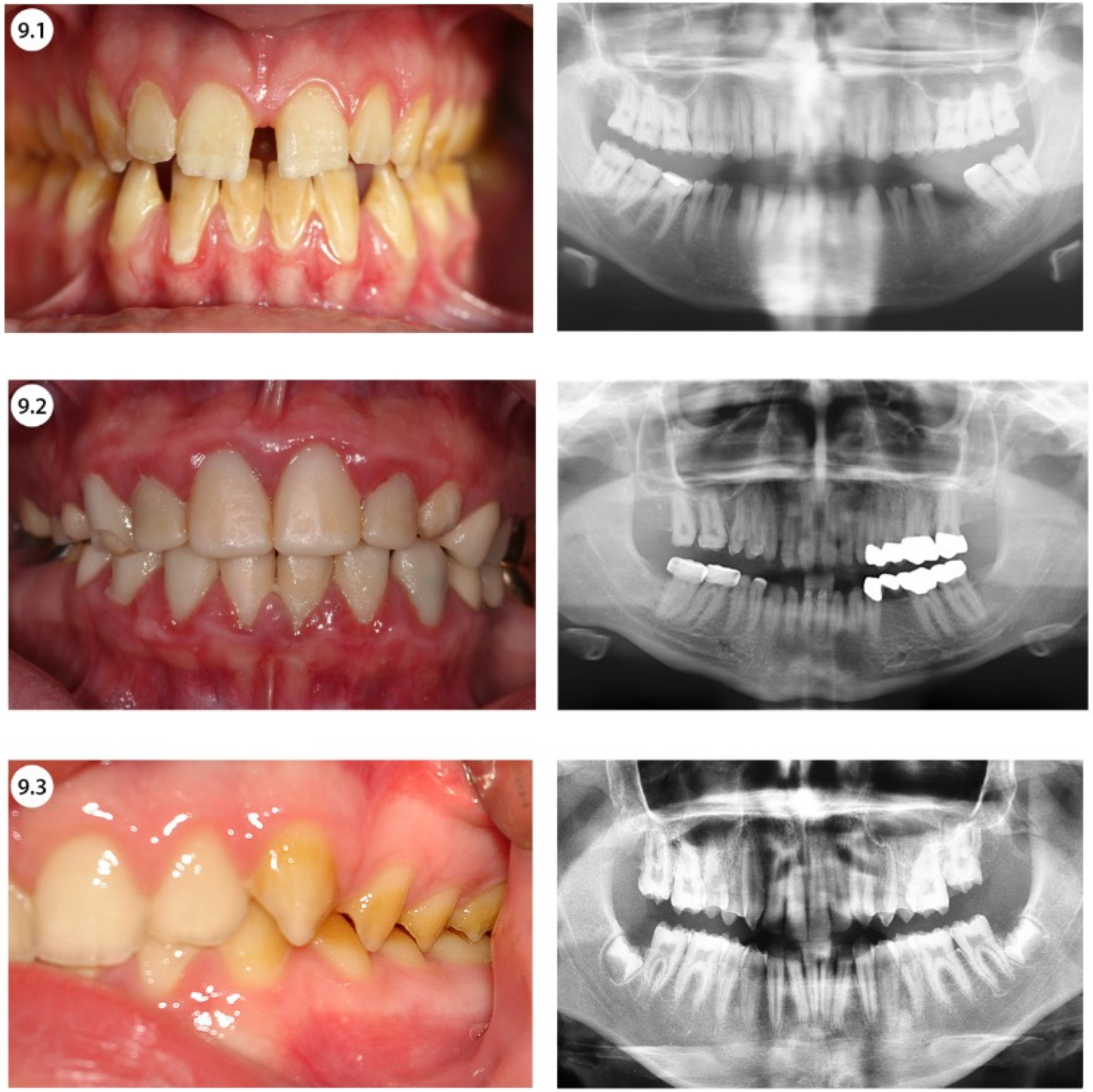

Supplementary Figure 1G *ACP4*

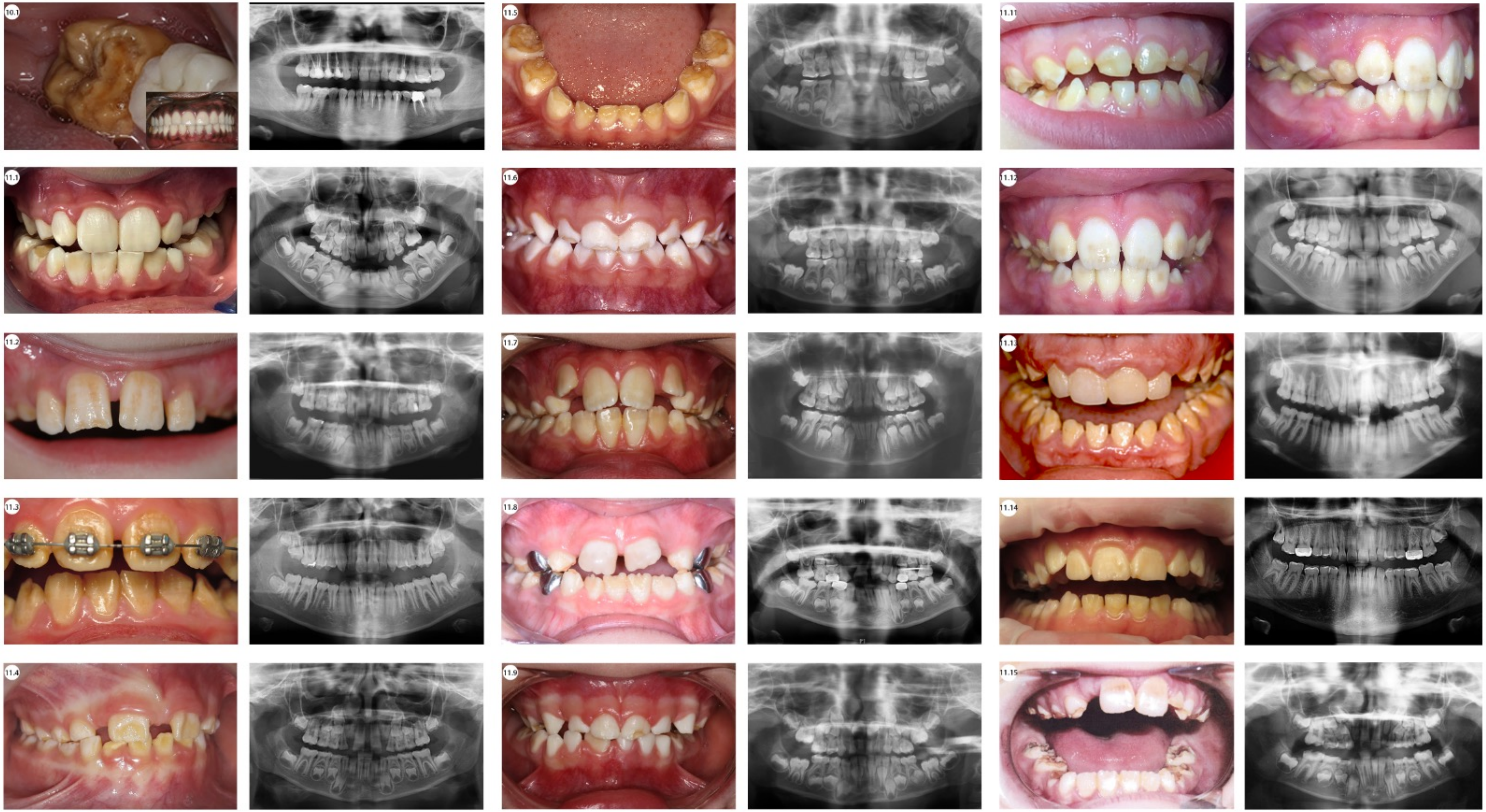

Supplementary Figure 1H *KLK4 and MMP20*

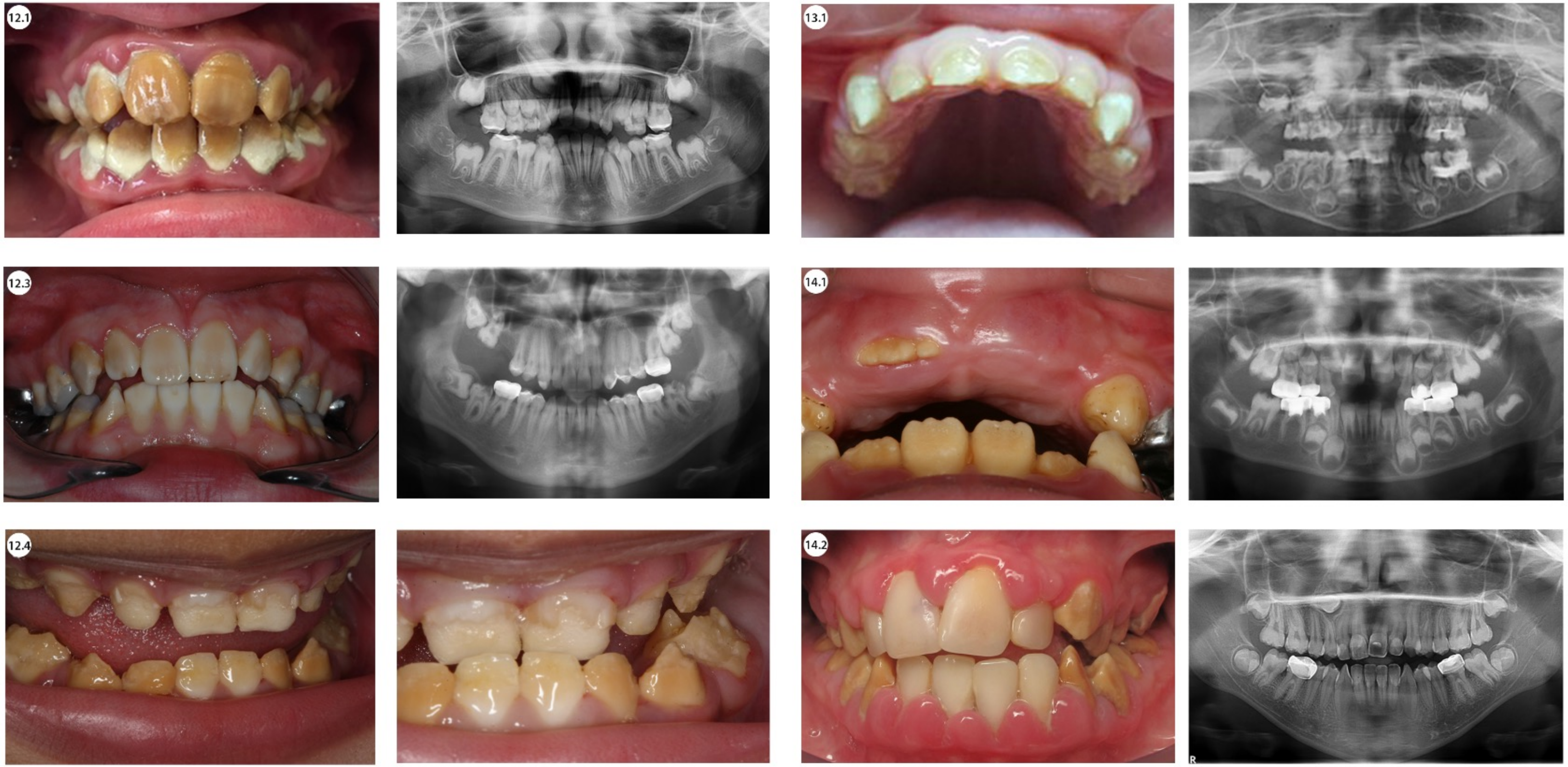

Supplementary Figure 1I ***WDR72, C4ORF26/ODAPH and SLC24A4***

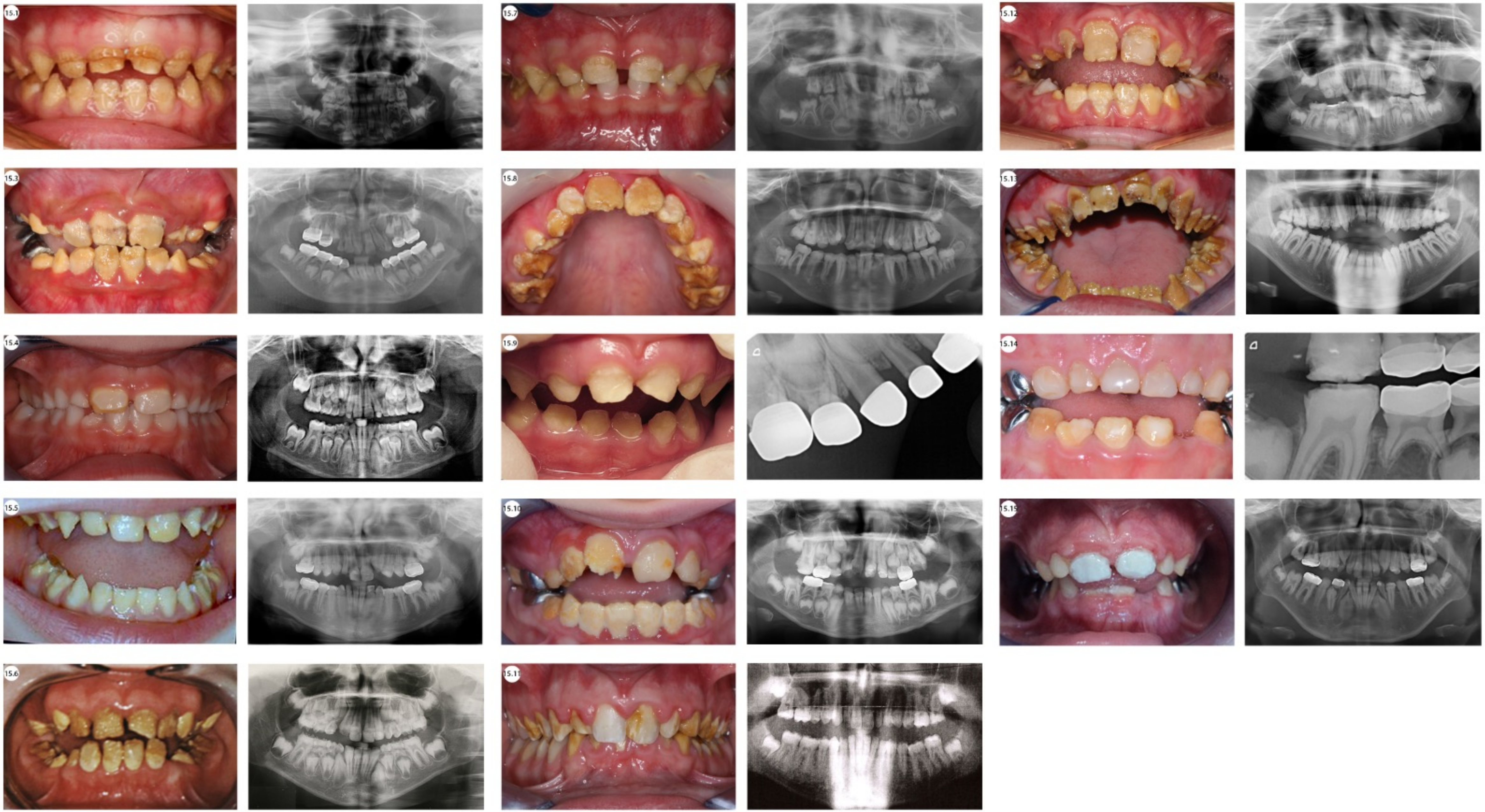

Supplementary Figure 1J *FAM83H*

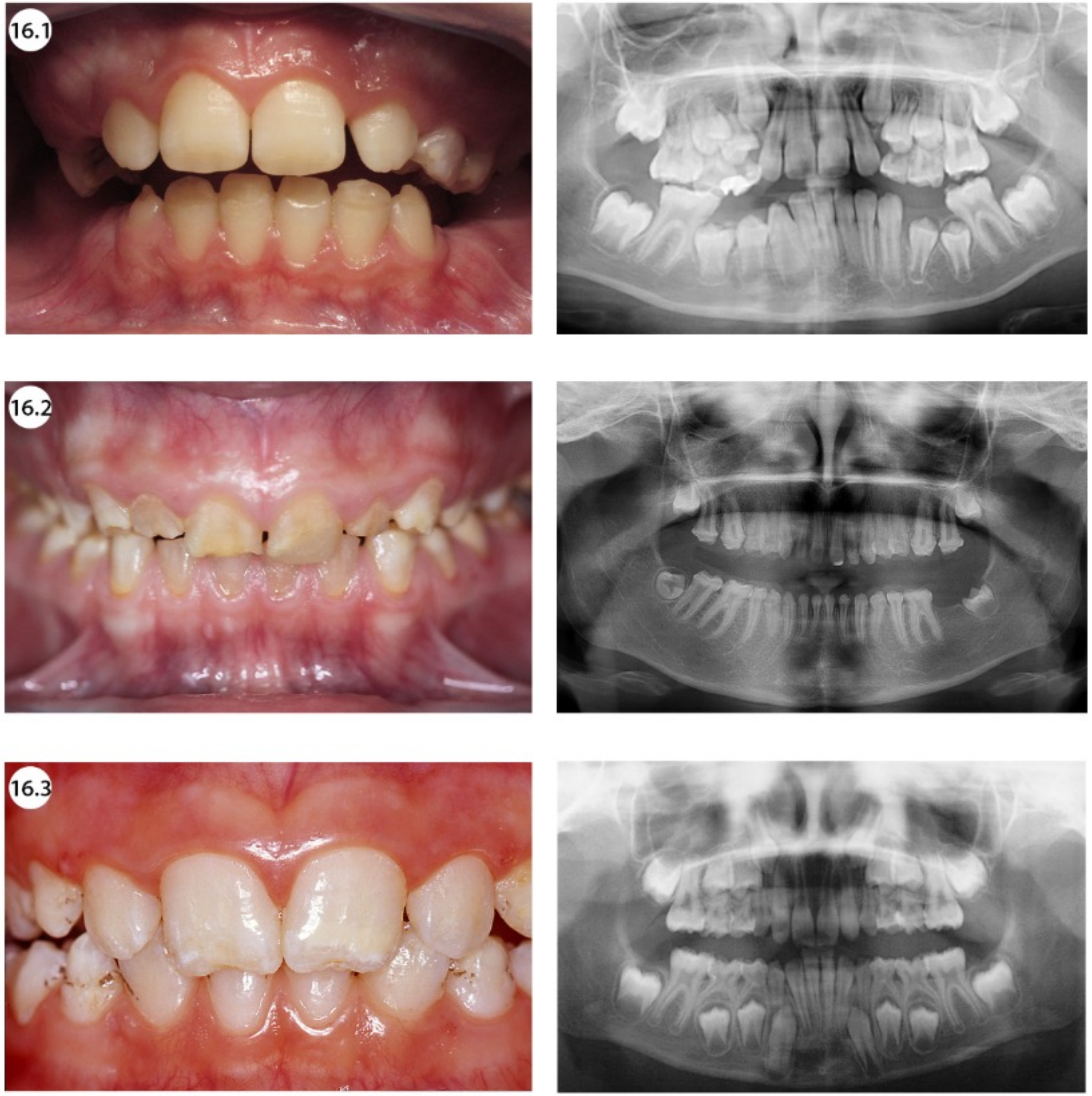

Supplementary Figure 1K *DLX3*

Syndromic

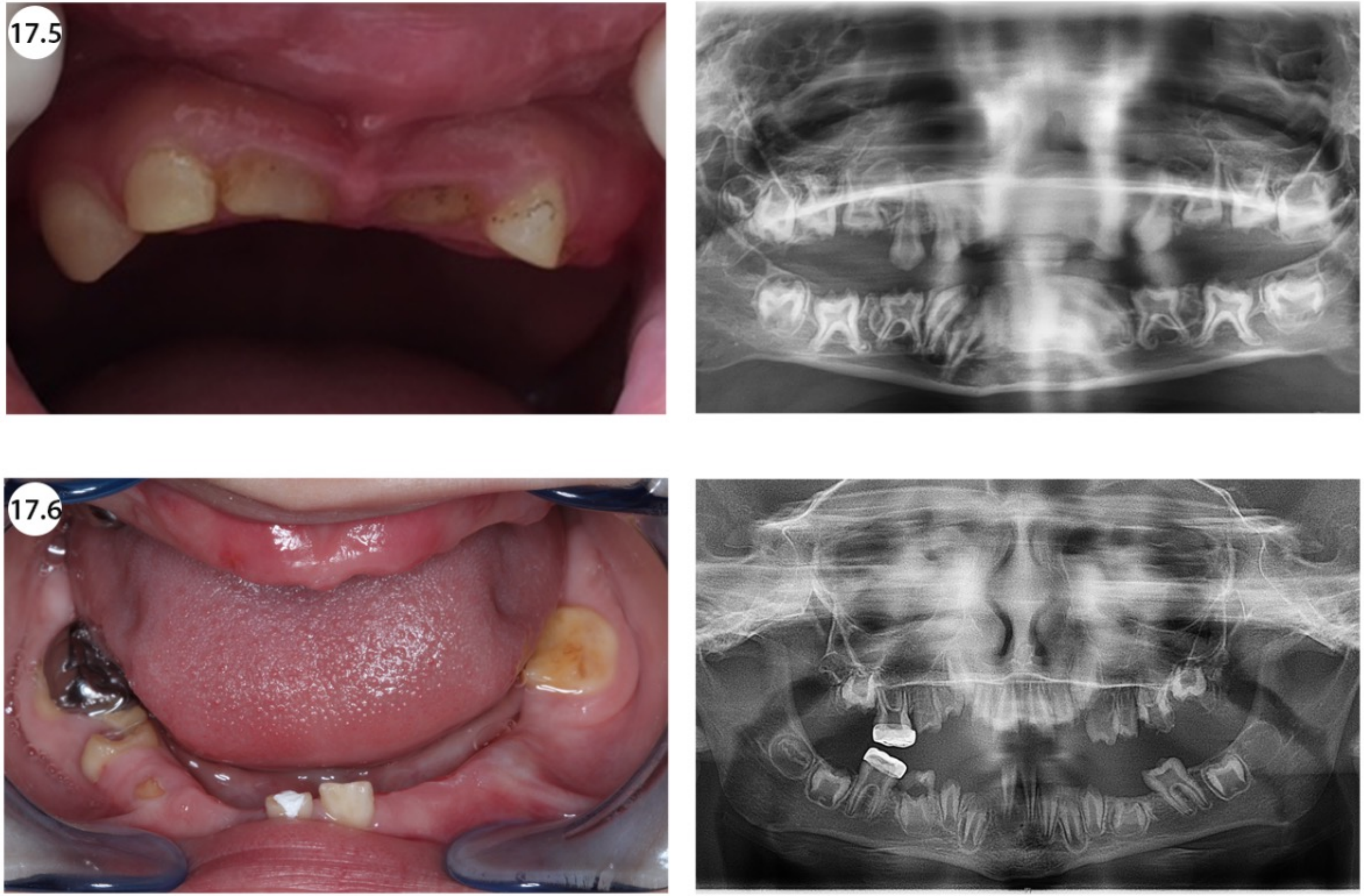

Supplementary Figure 1L *LTBP3*

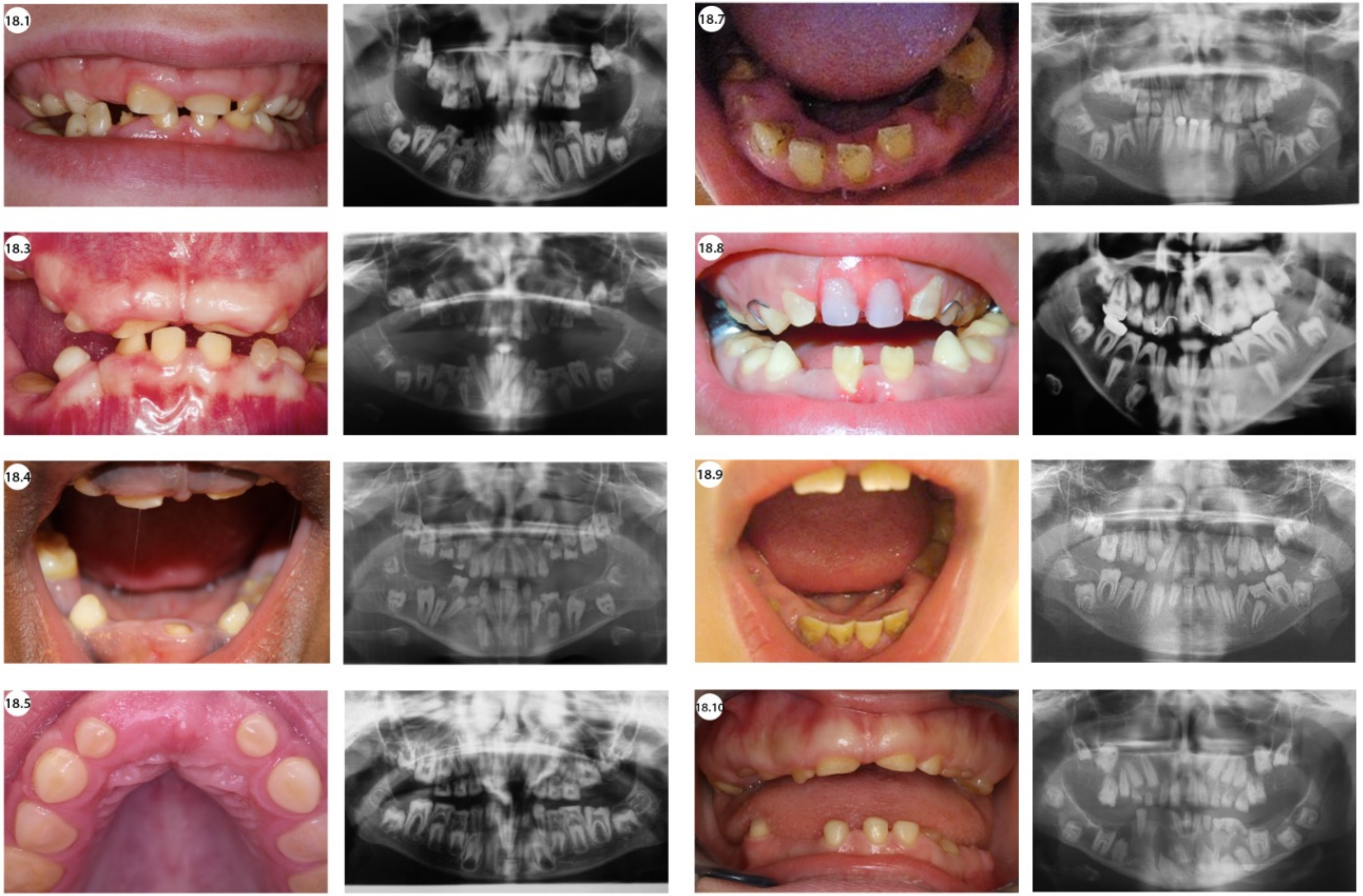

Supplementary Figure 1M *FAM20A*

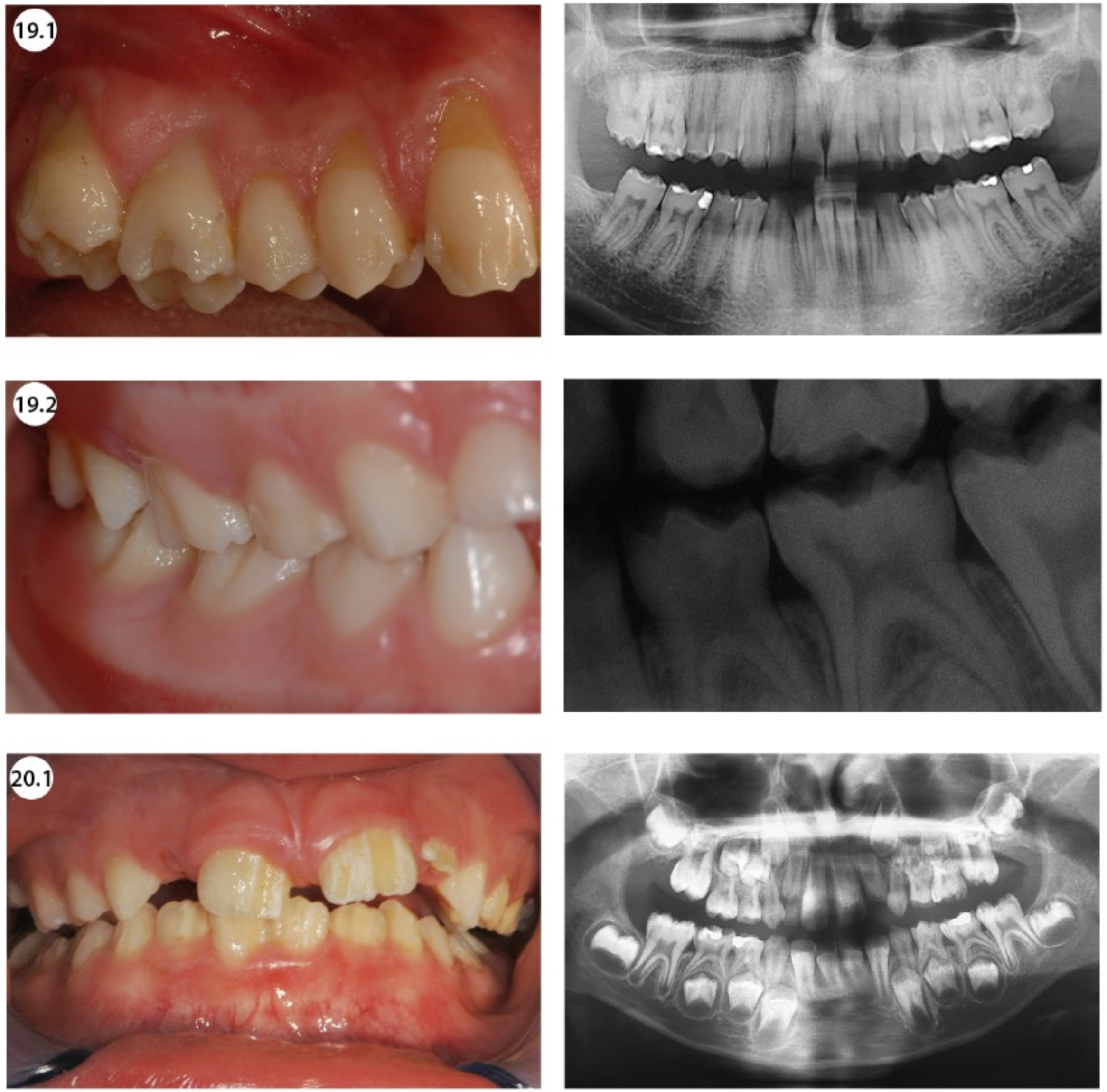

Supplementary Figure 1N ***GALNS and ARHGAP6***

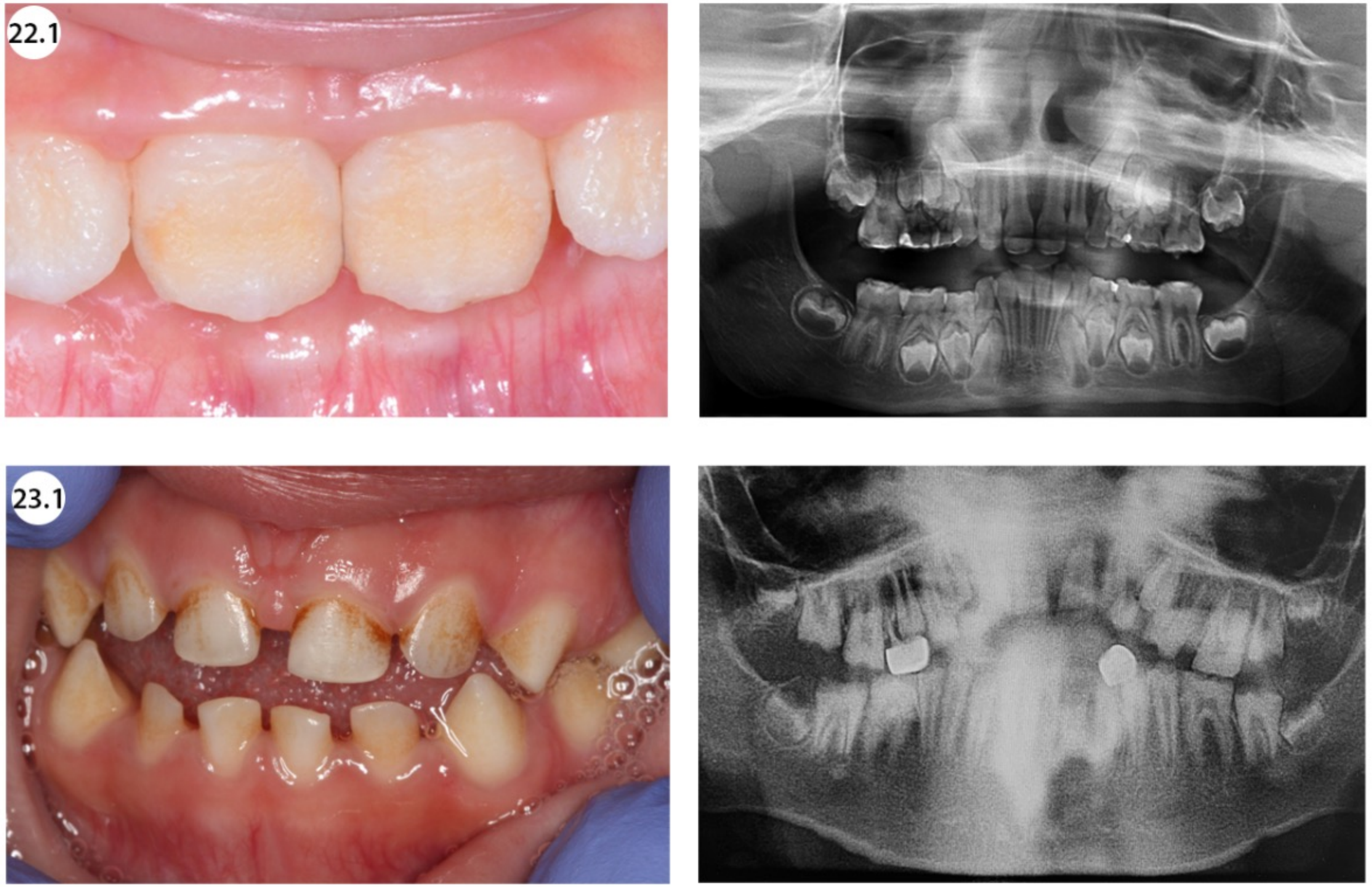

Supplementary Figure 1O *TGFBR2* and *SLC13A5*

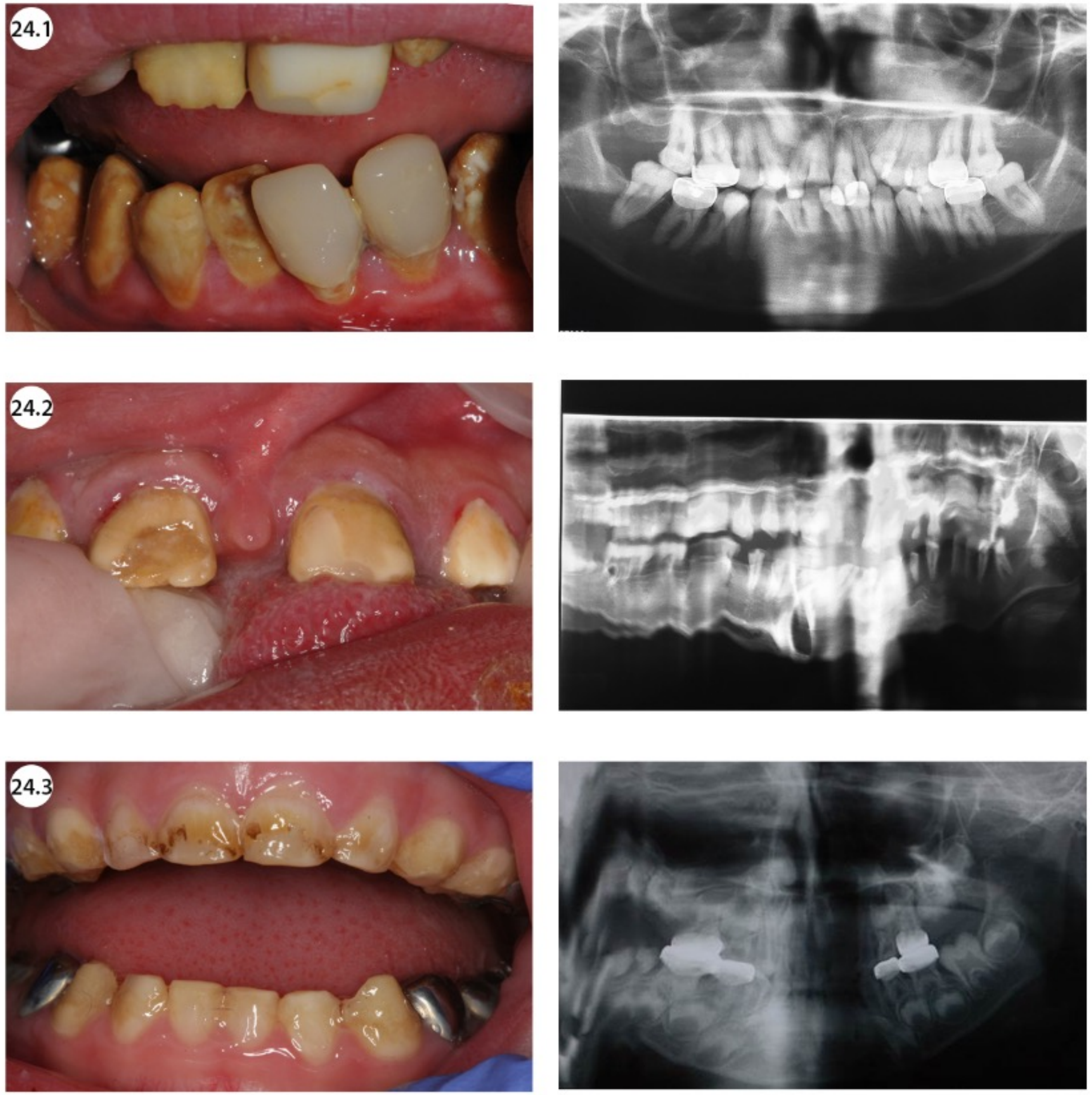

Supplementary Figure 1P *ROGDI*

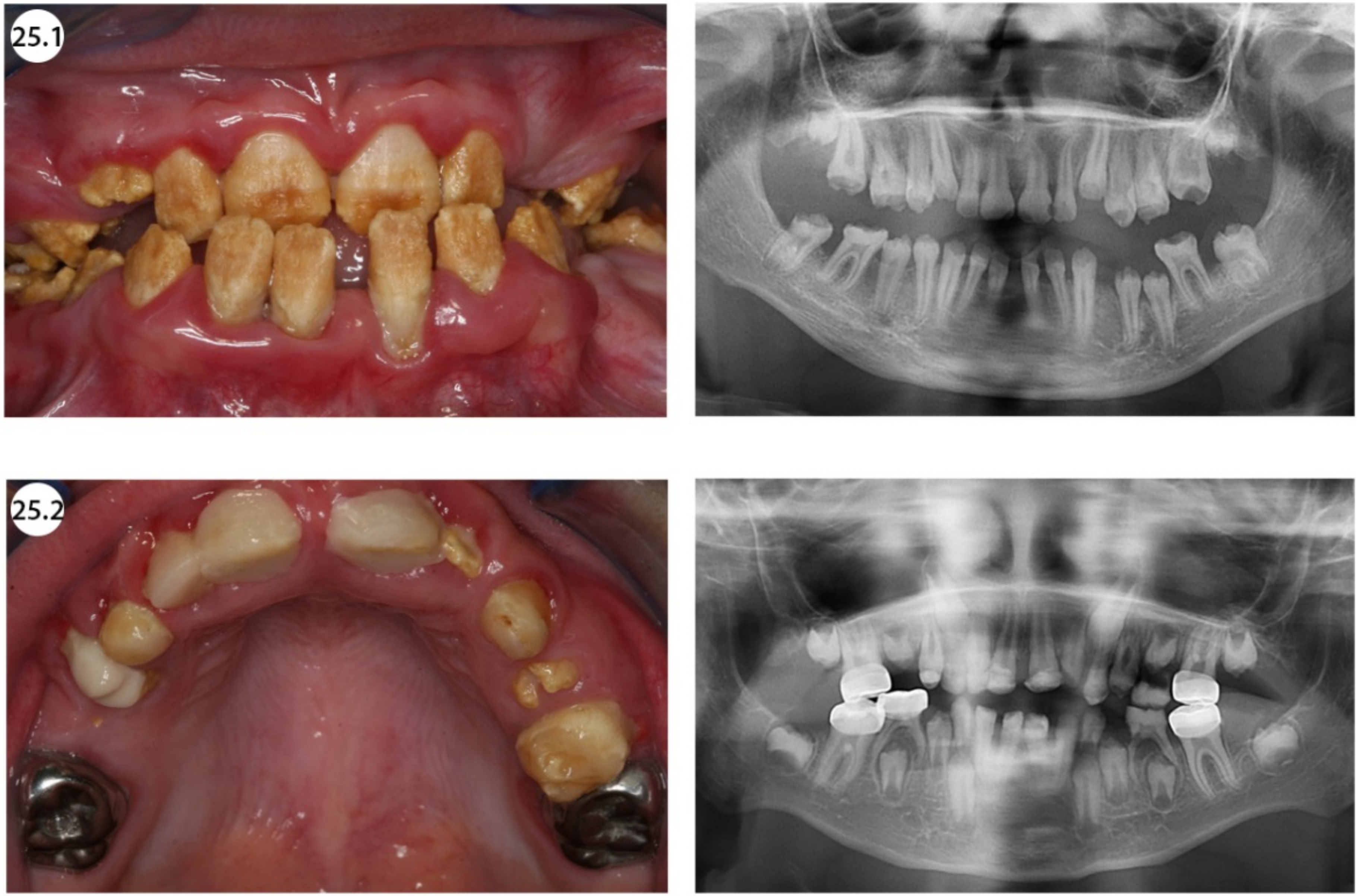

Supplementary Figure 1Q *SLC10A7*

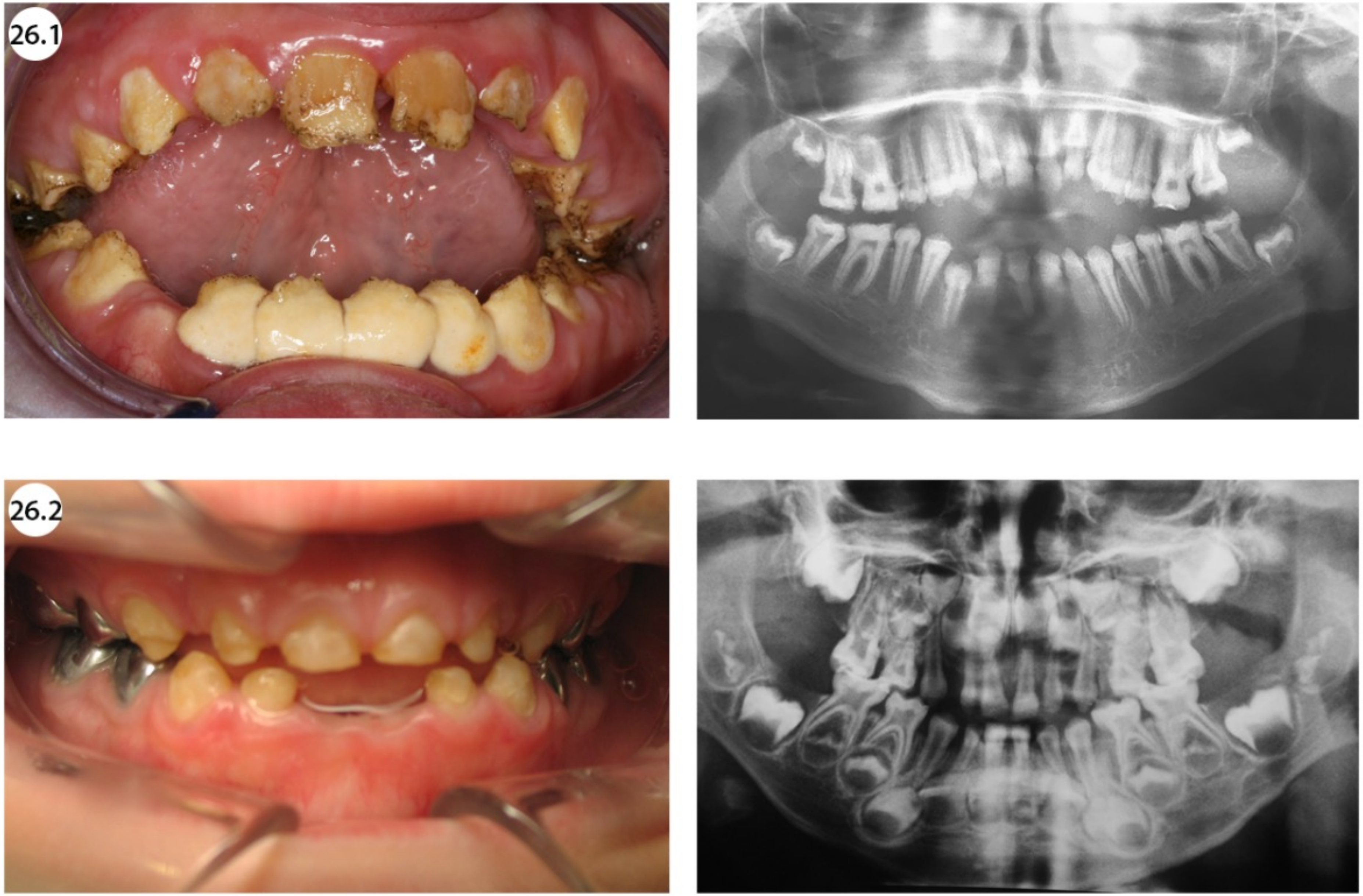

Supplementary Figure 1R *CNNM4*

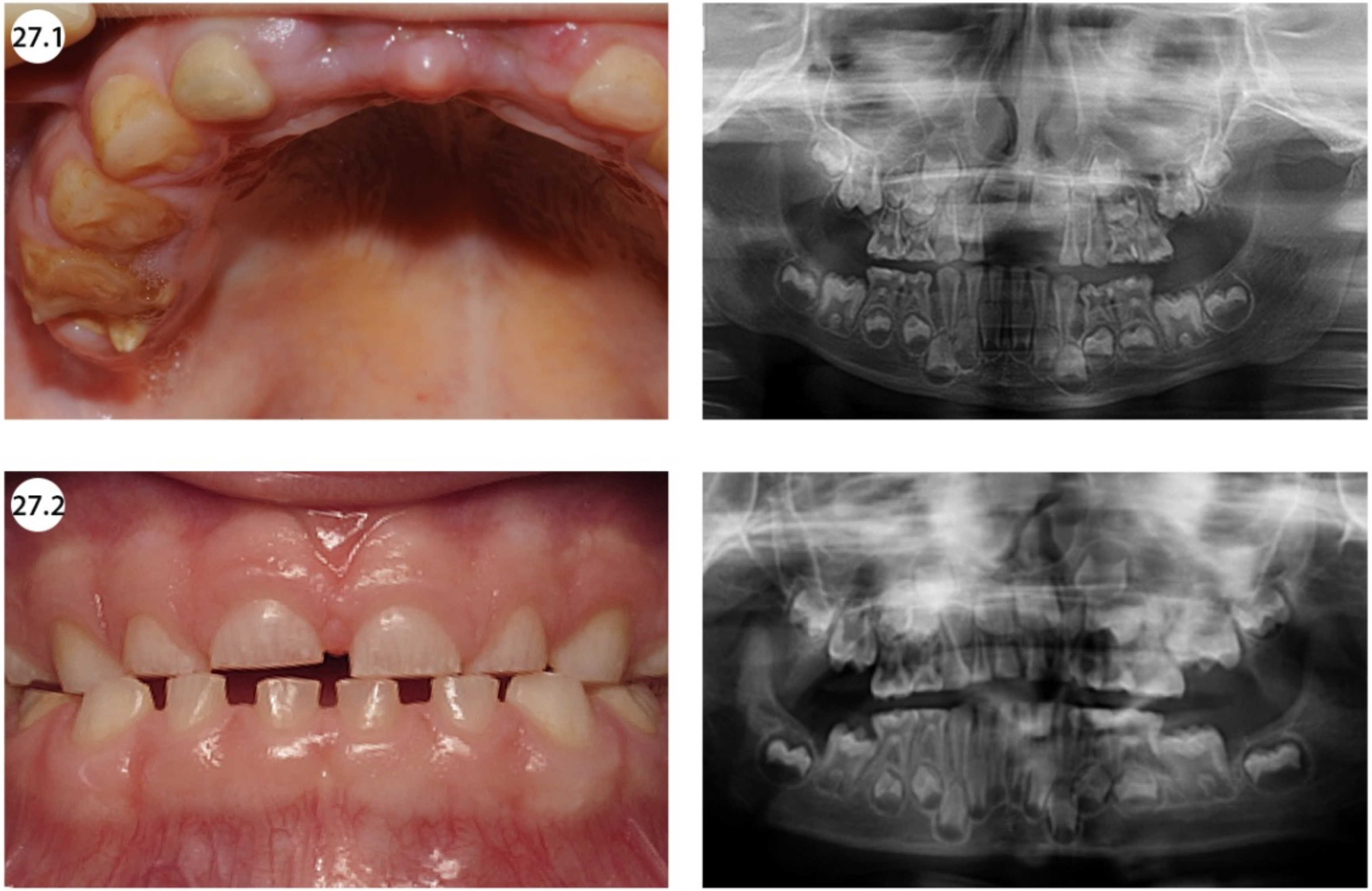

Supplementary Figure 1S *DLX3*
