# Supplementary material for: Amelogenesis imperfecta: Next-generation sequencing sheds light on Witkop’s classification
Source: Front Physiol. 2023 May 9;14:1130175. doi: 10.3389/fphys.2023.1130175 (PMC10205041; doi:10.3389/fphys.2023.1130175)
Supplement: Supplementary file 3 [file Presentation1.zip › Supplementary Figure 2.PDF]

# Isolated AI

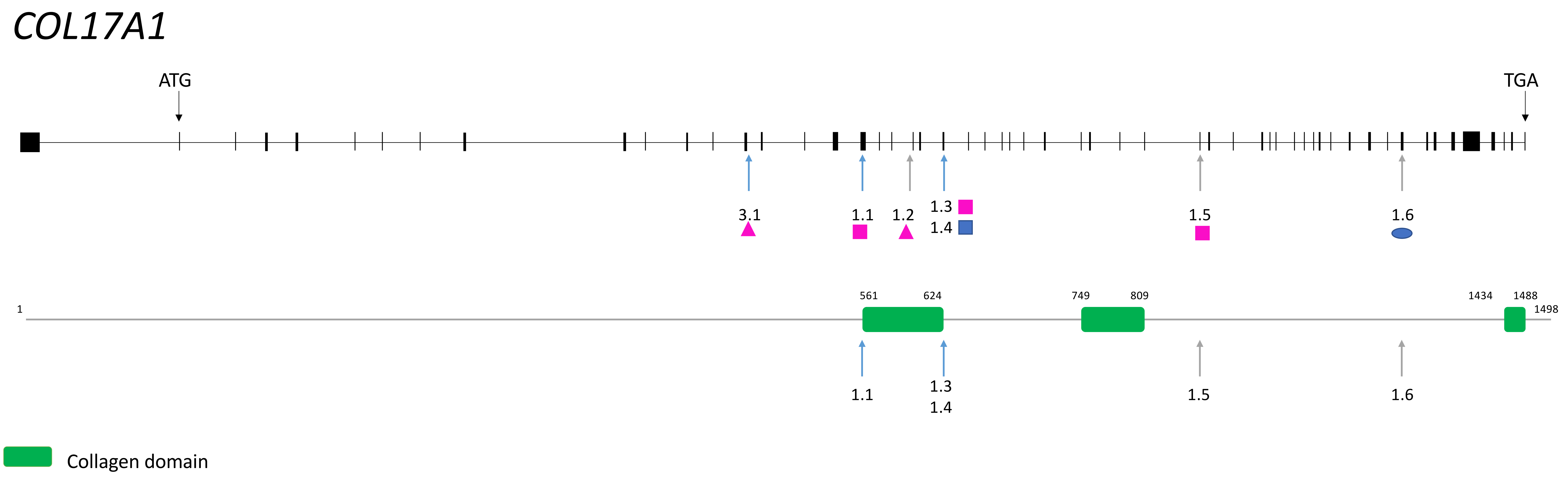


Supplementary Figure 2A

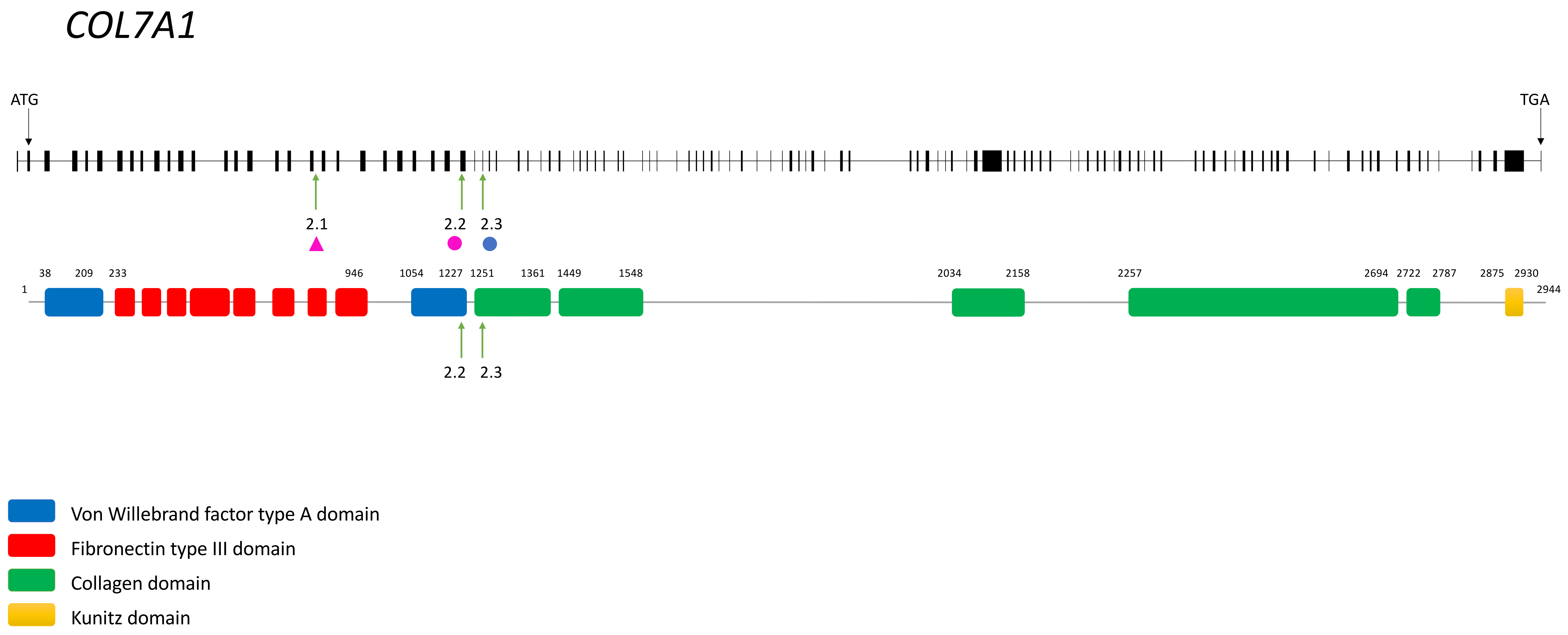


Supplementary Figure 2B

*LAMA3*

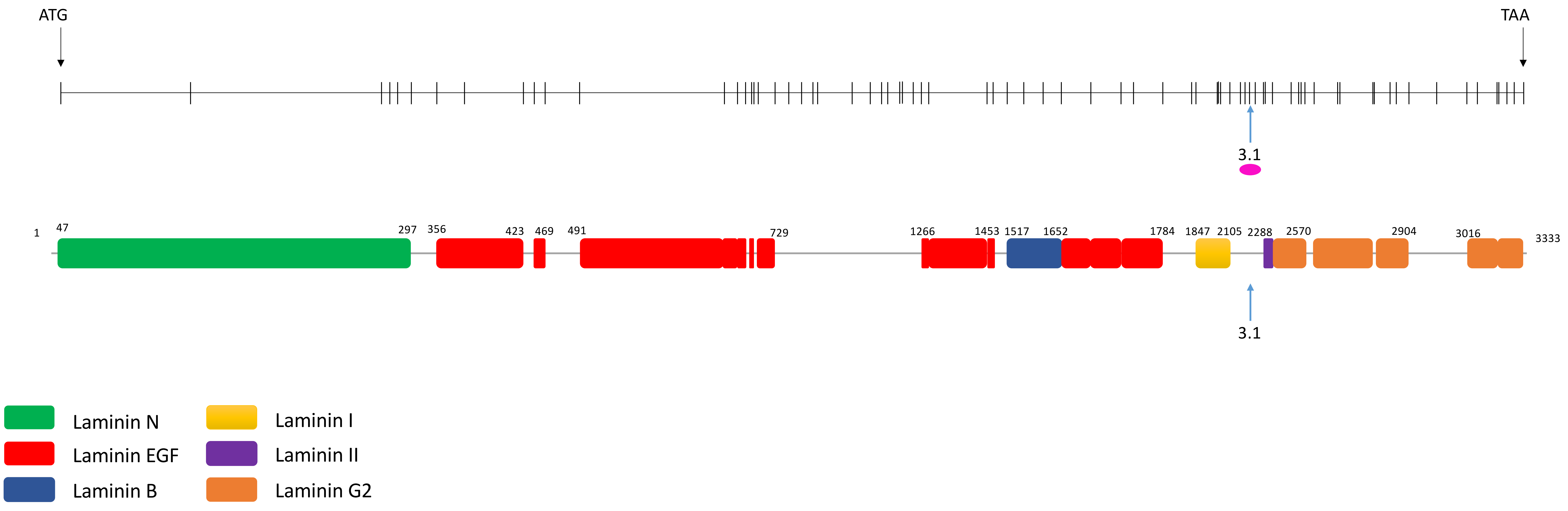


Supplementary Figure 2C

*LAMB3*

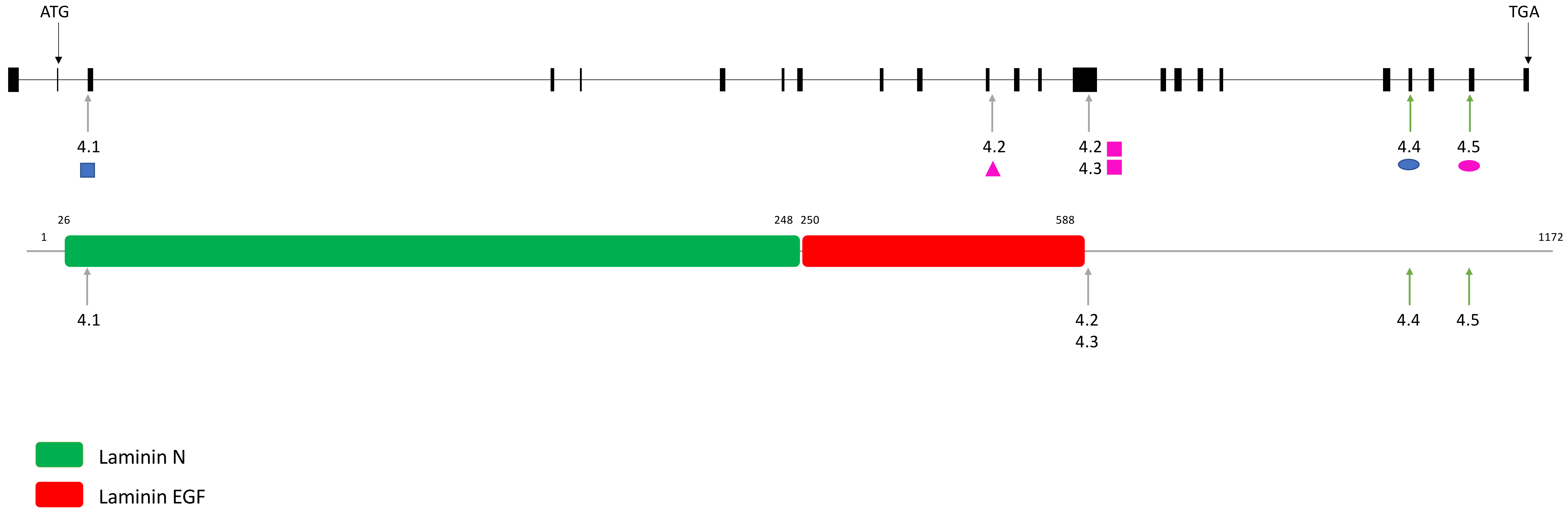


Supplementary Figure 2D

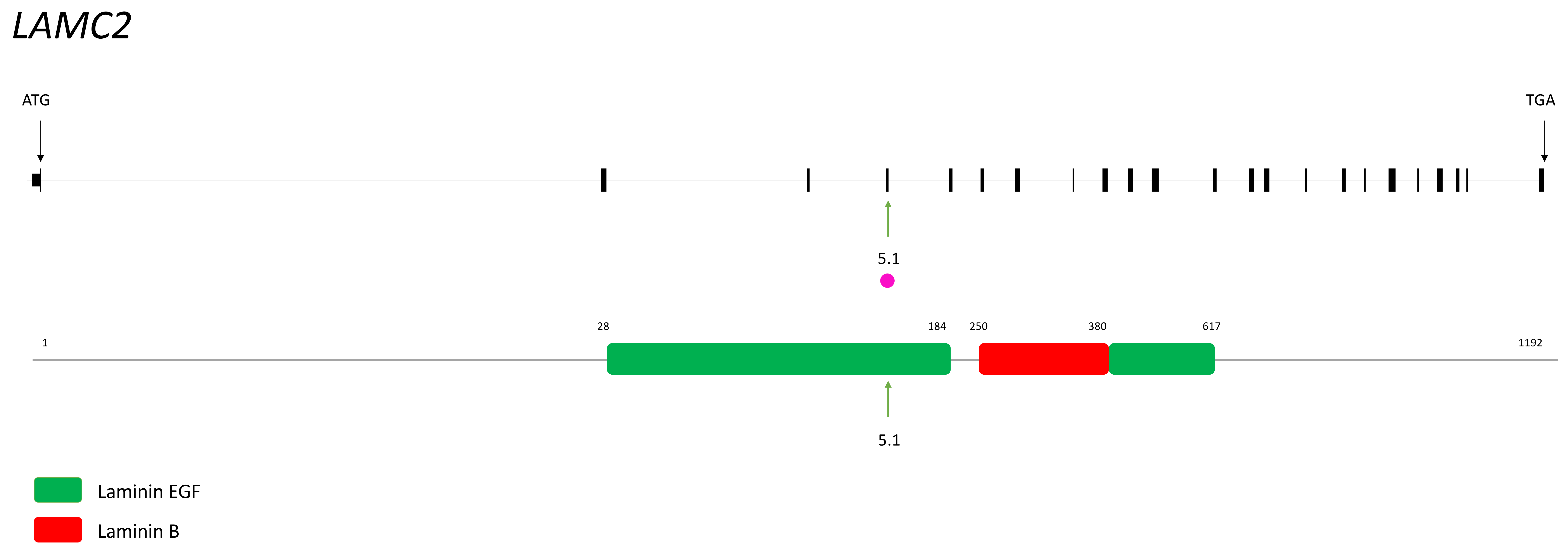


Supplementary Figure 2E

# *ENAM*

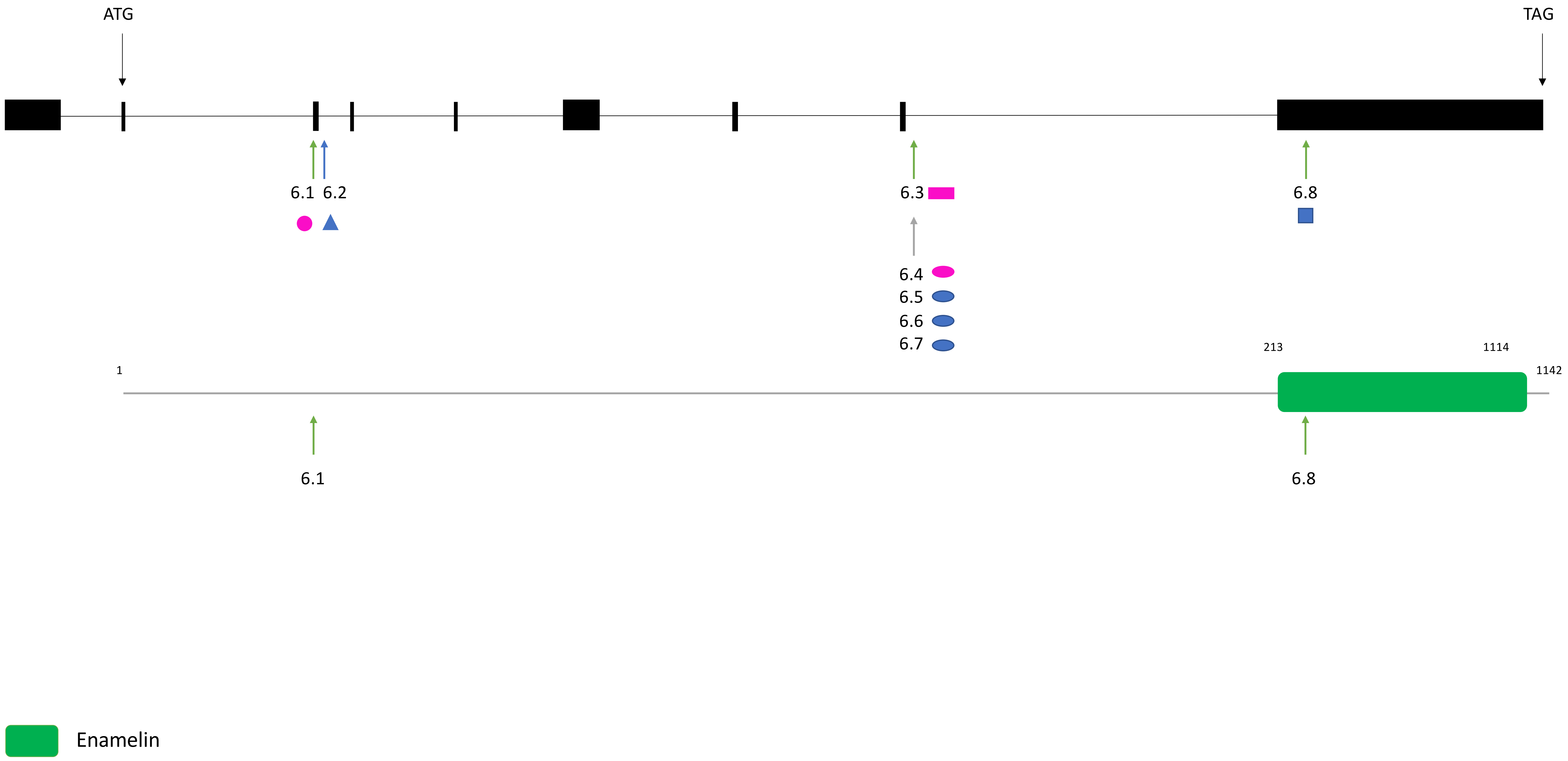


Supplementary Figure 2F

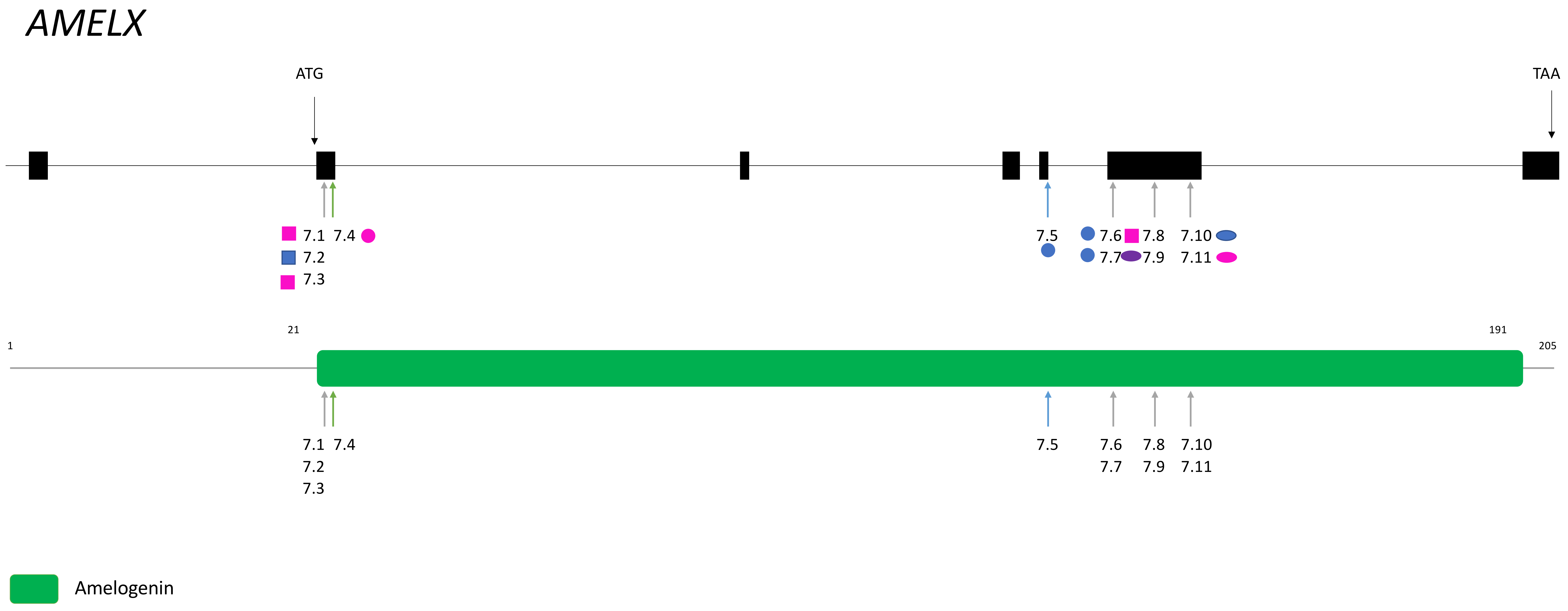


Supplementary Figure 2G

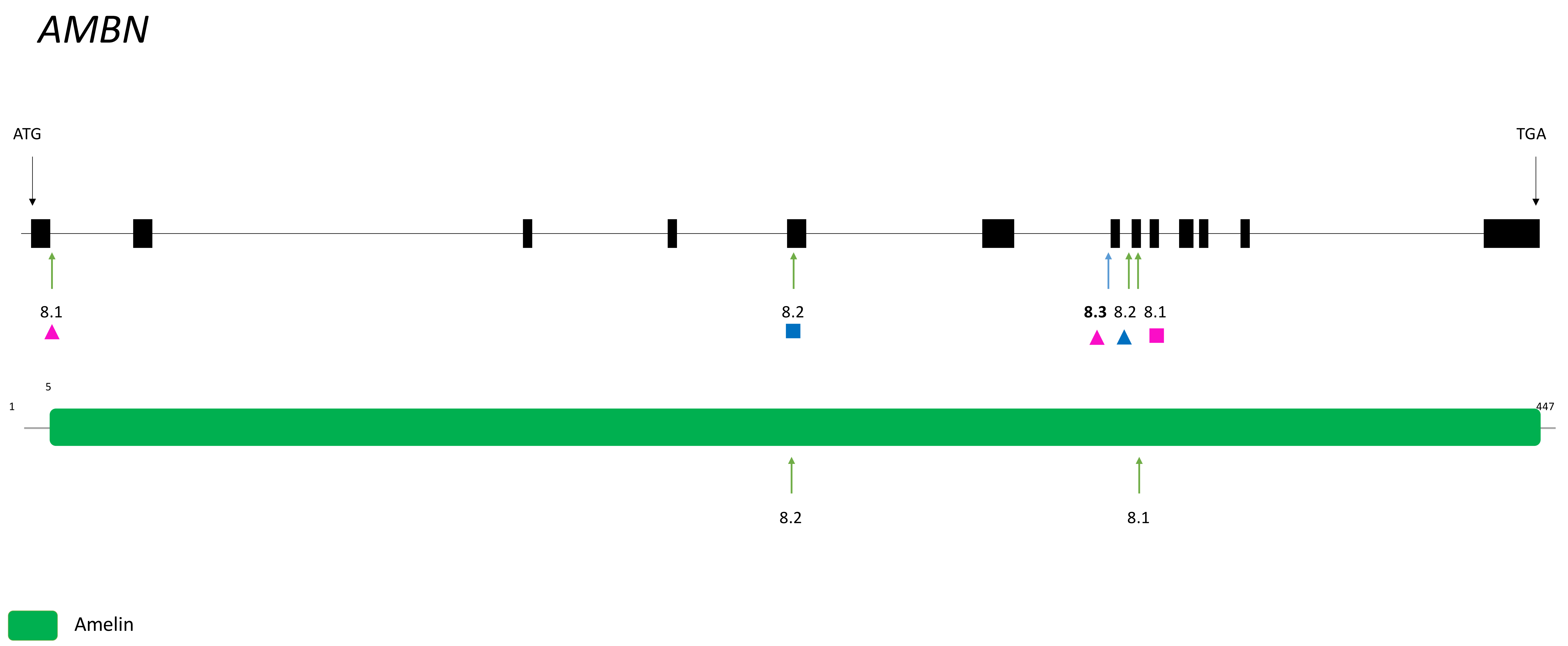


Supplementary Figure 2H

# *ACP4*

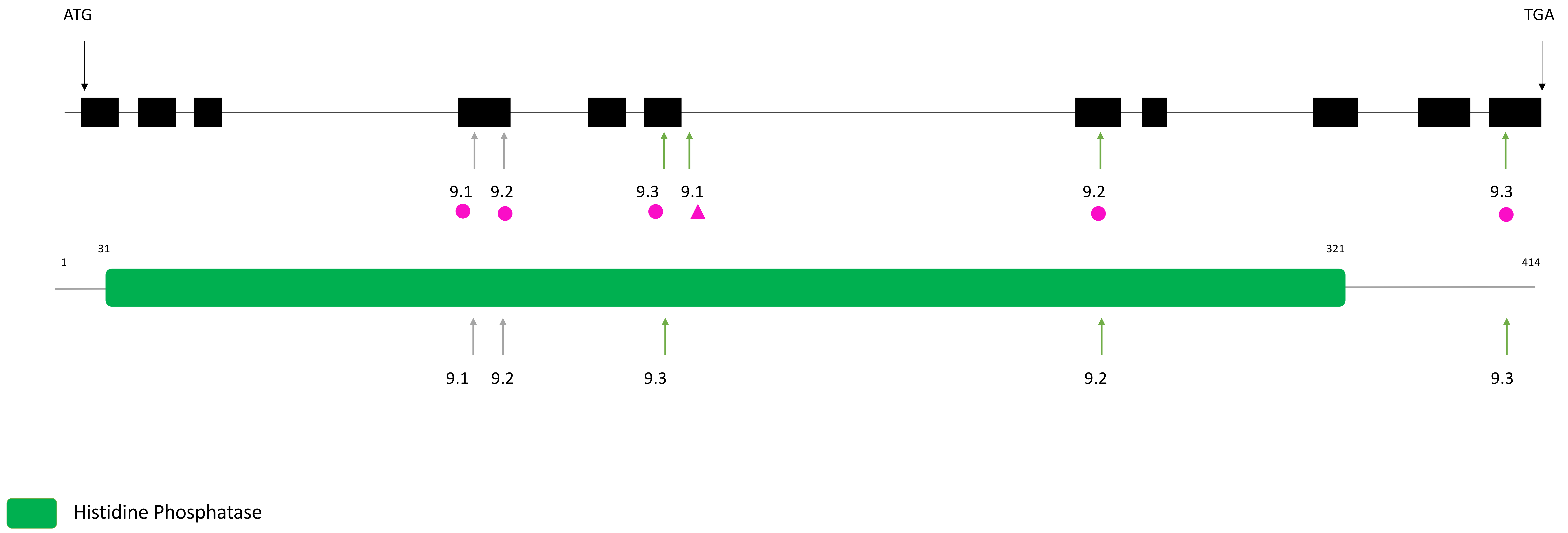


Supplementary Figure 2I

## *KLK4*

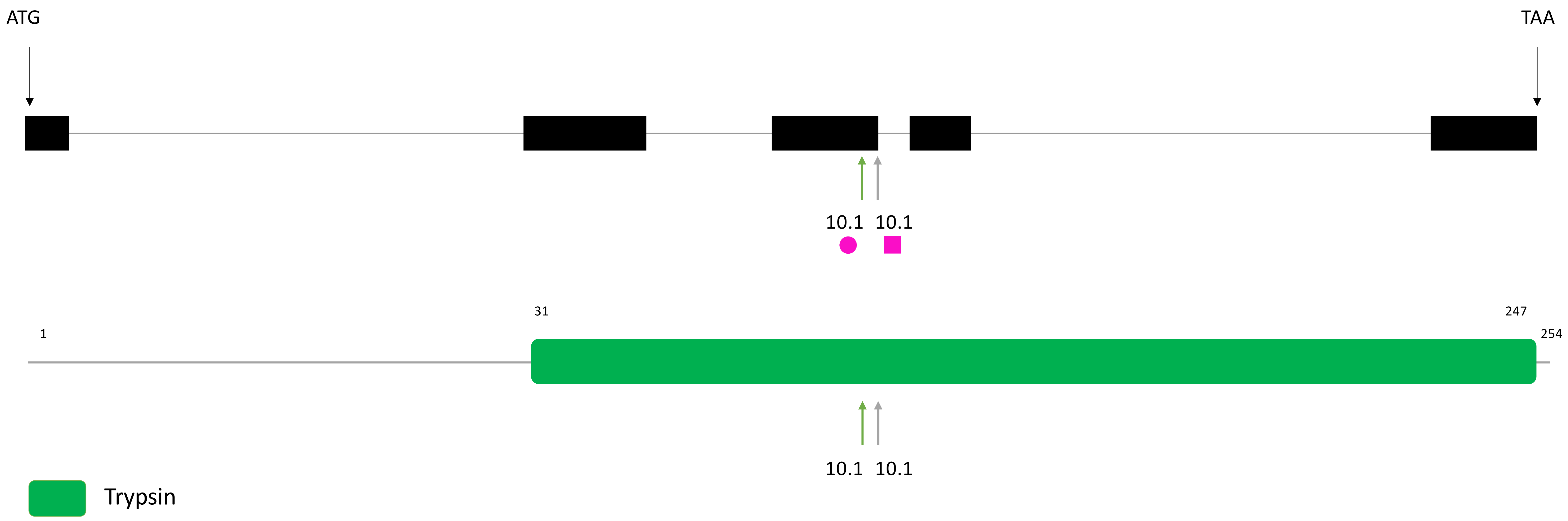


Supplementary Figure 2J

## *MMP20*

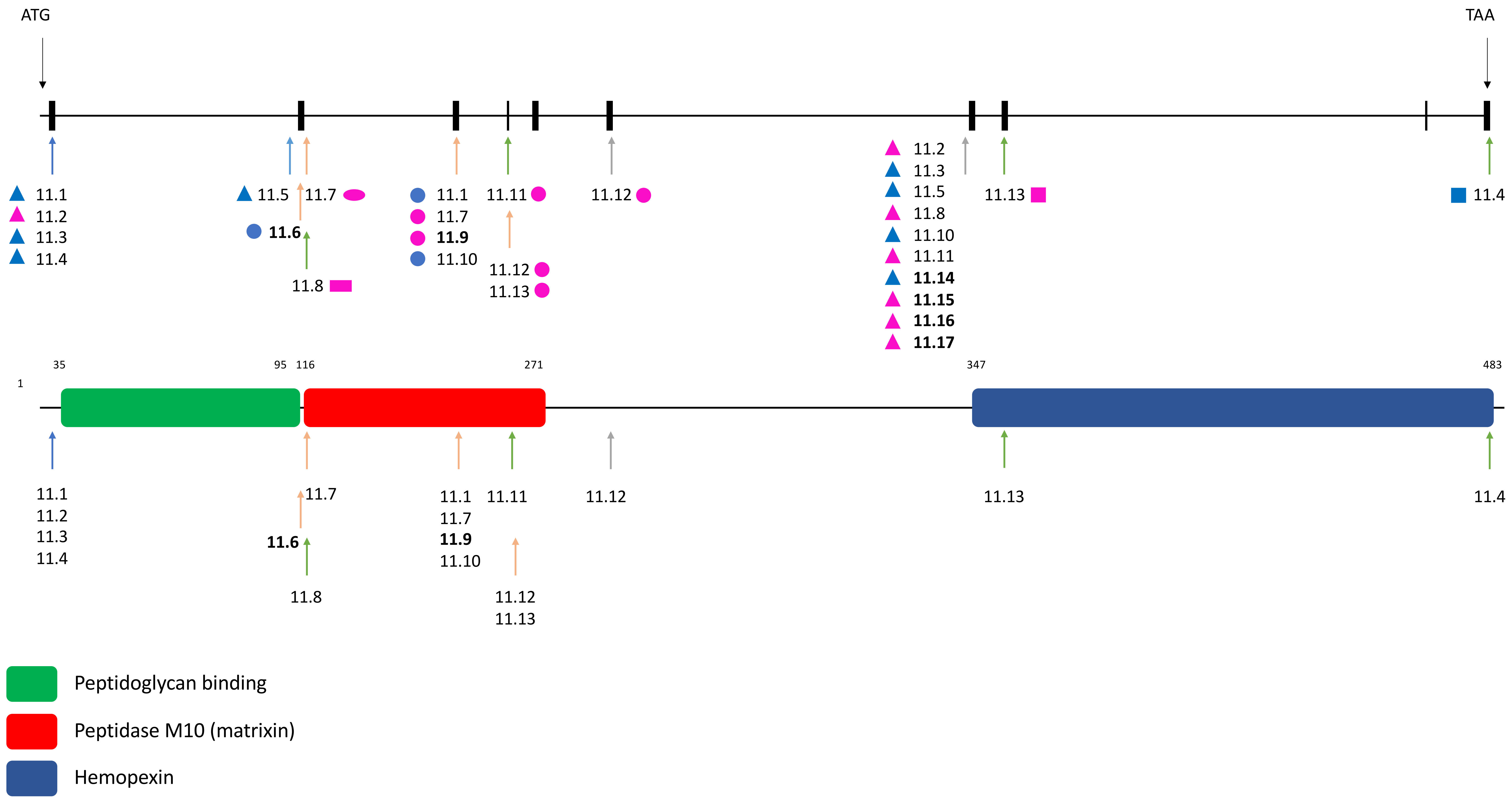


Supplementary Figure 2K

*WDR72*

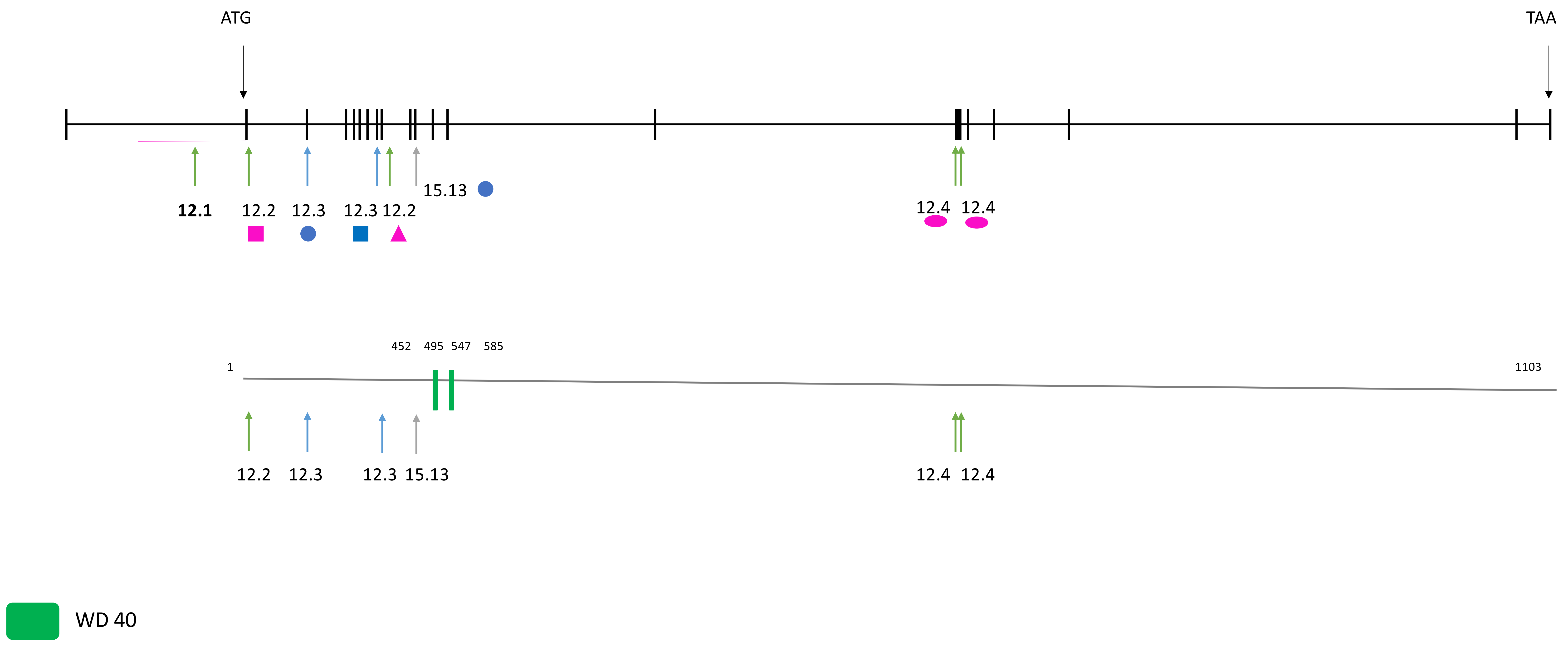


Supplementary Figure 2L

*ODAPH*

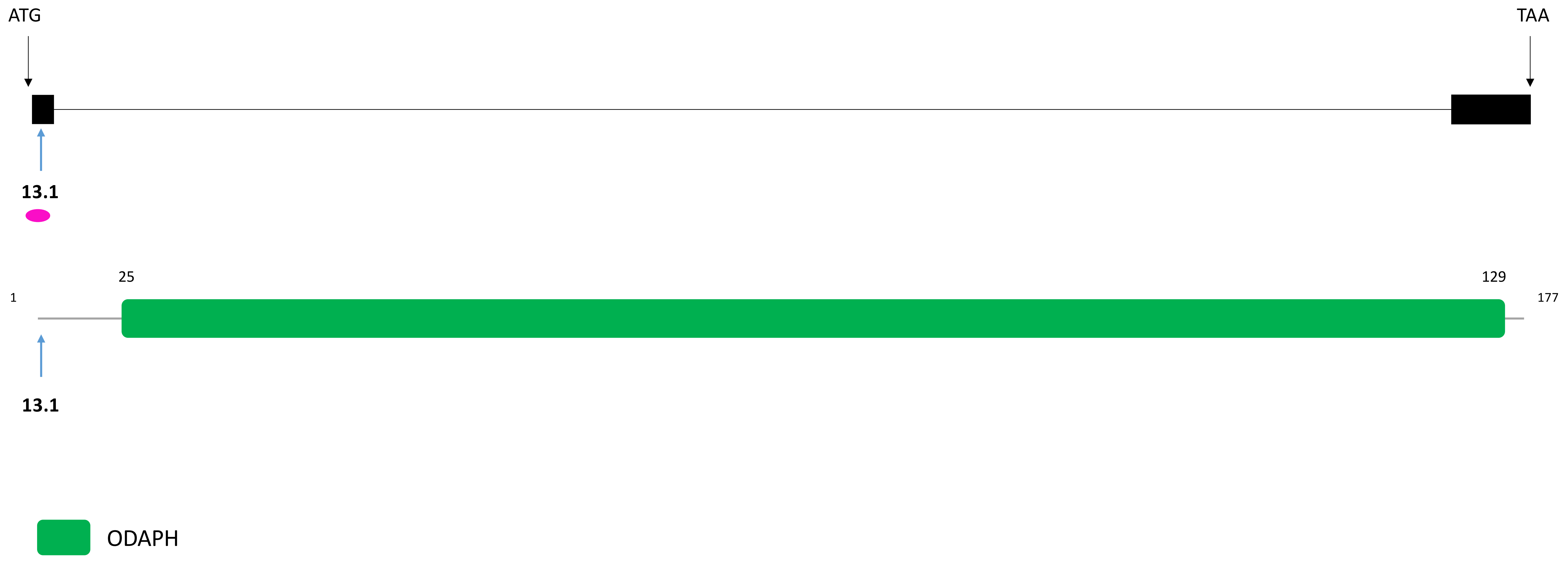


Supplementary Figure 2M

*SLC24A4*

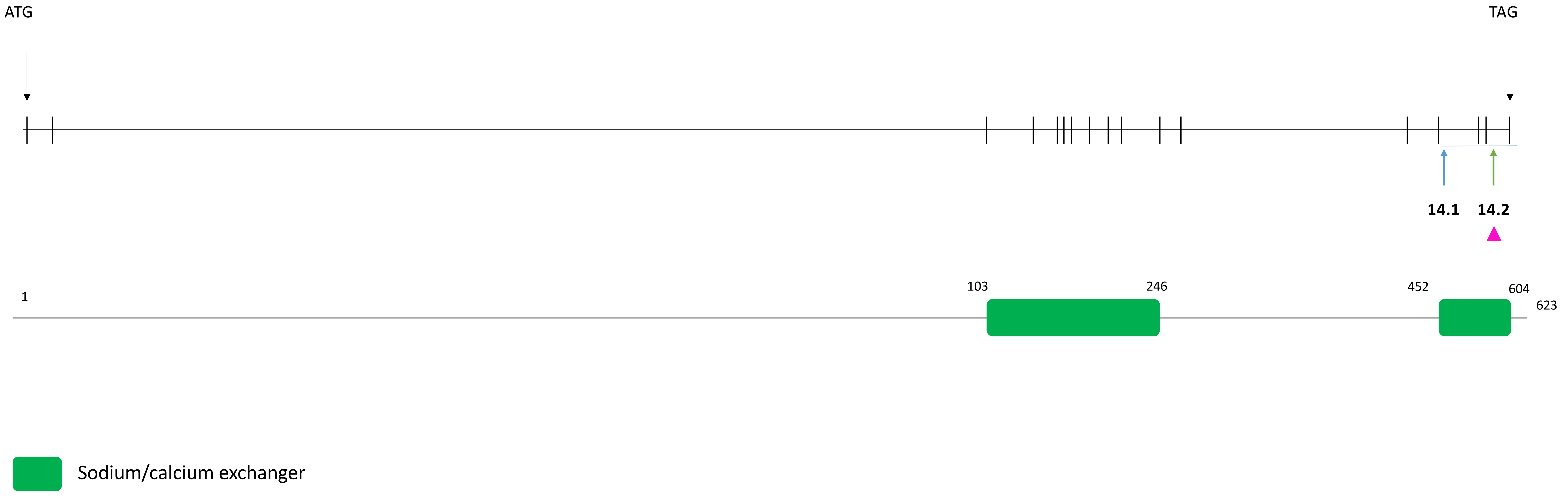


Supplementary Figure 2N

*FAM83H*

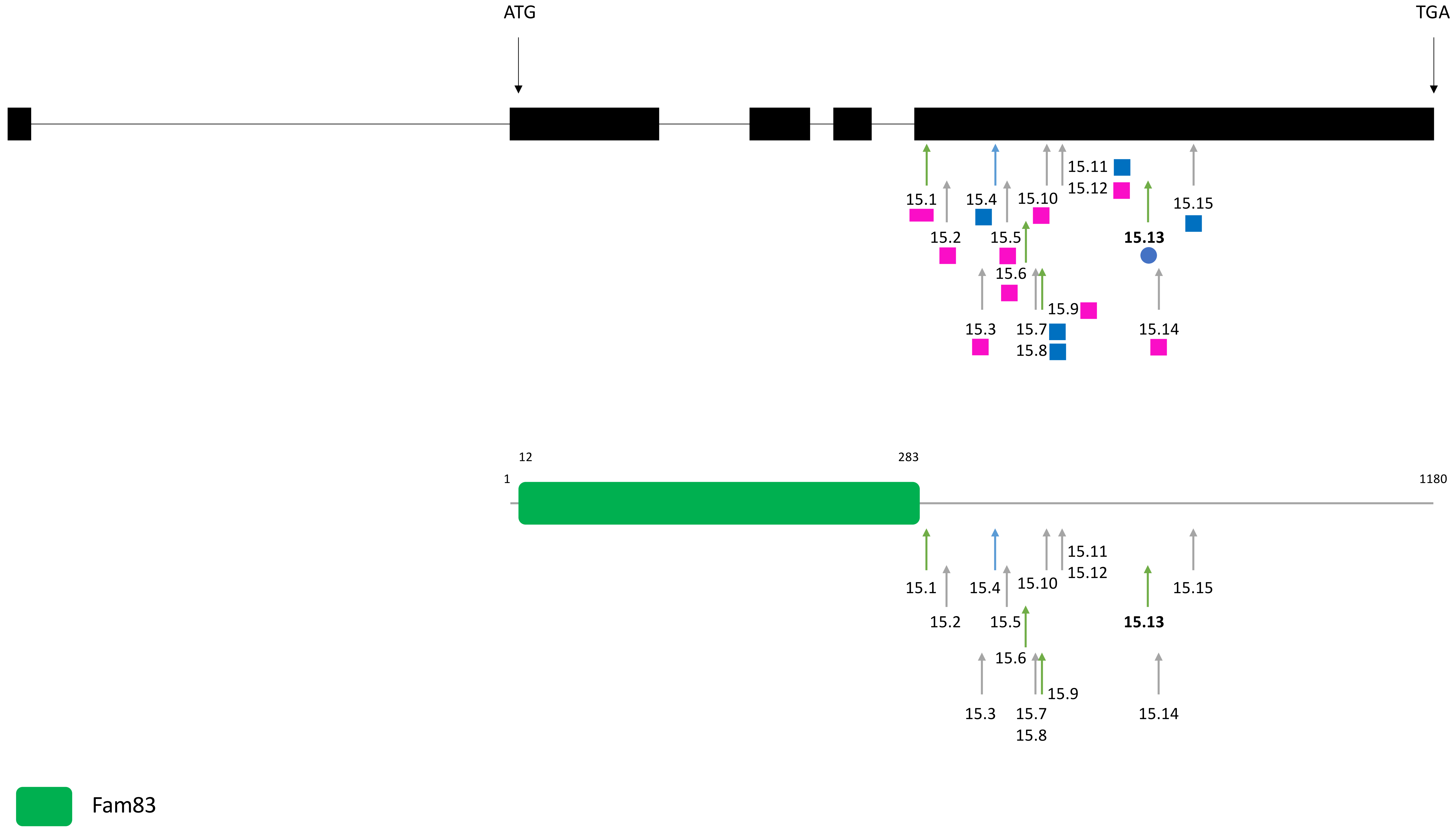


Supplementary Figure 2O

# *DLX3*

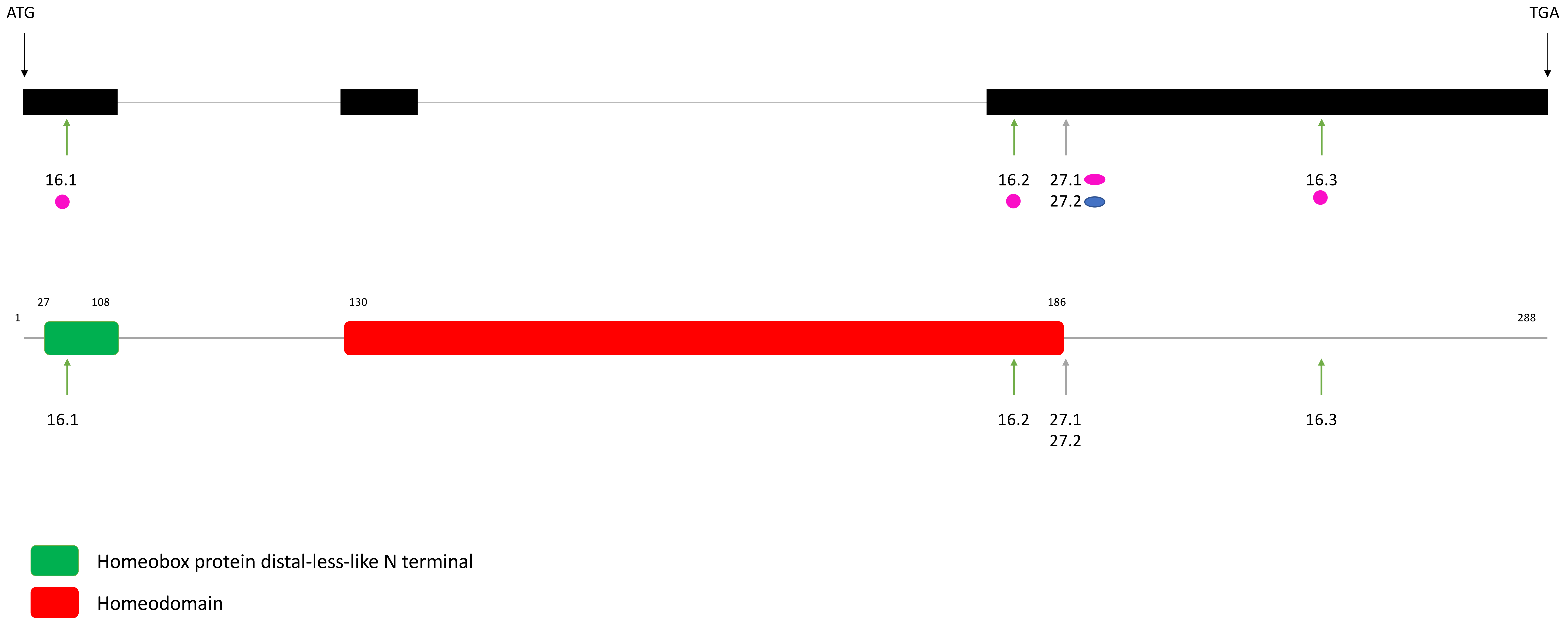


Supplementary Figure 2P

# Syndromic AI

*LTBP3*

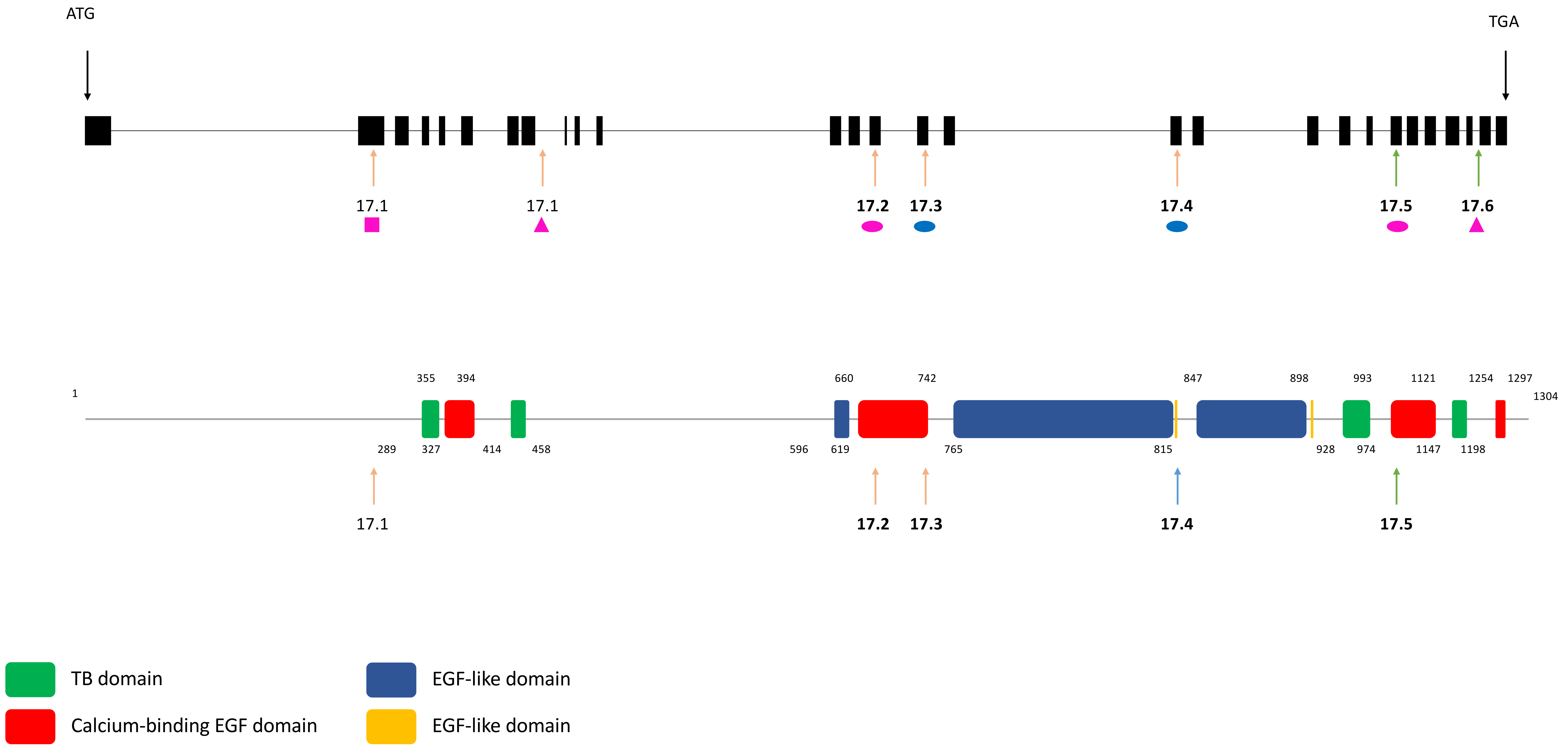


Supplementary Figure 2Q

*FAM20A*

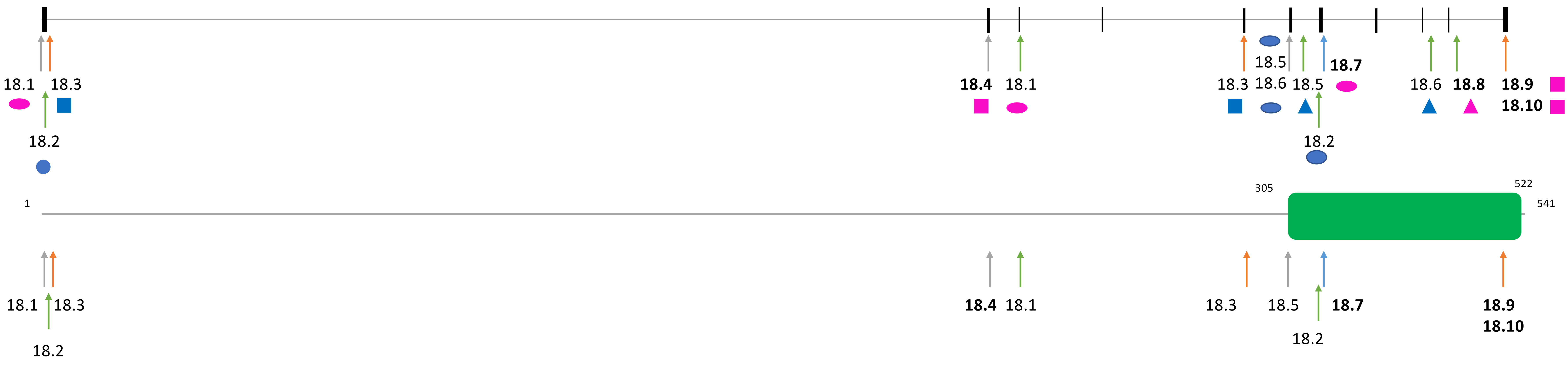


Supplementary Figure 2R

# *GALNS*

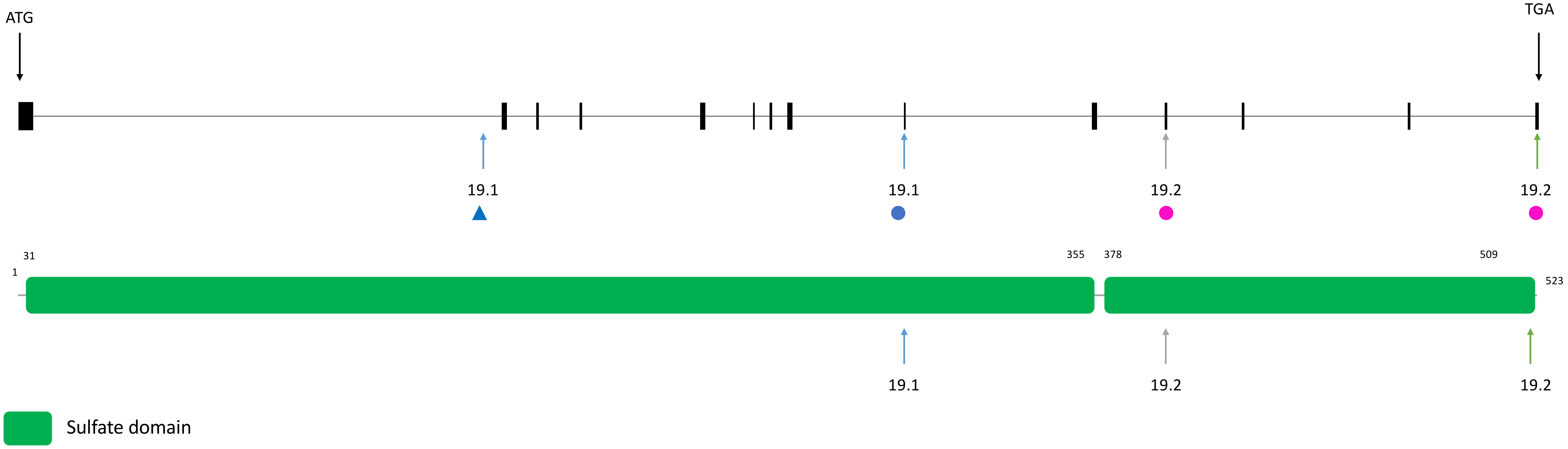


Supplementary Figure 2S

# *SLC13A5*

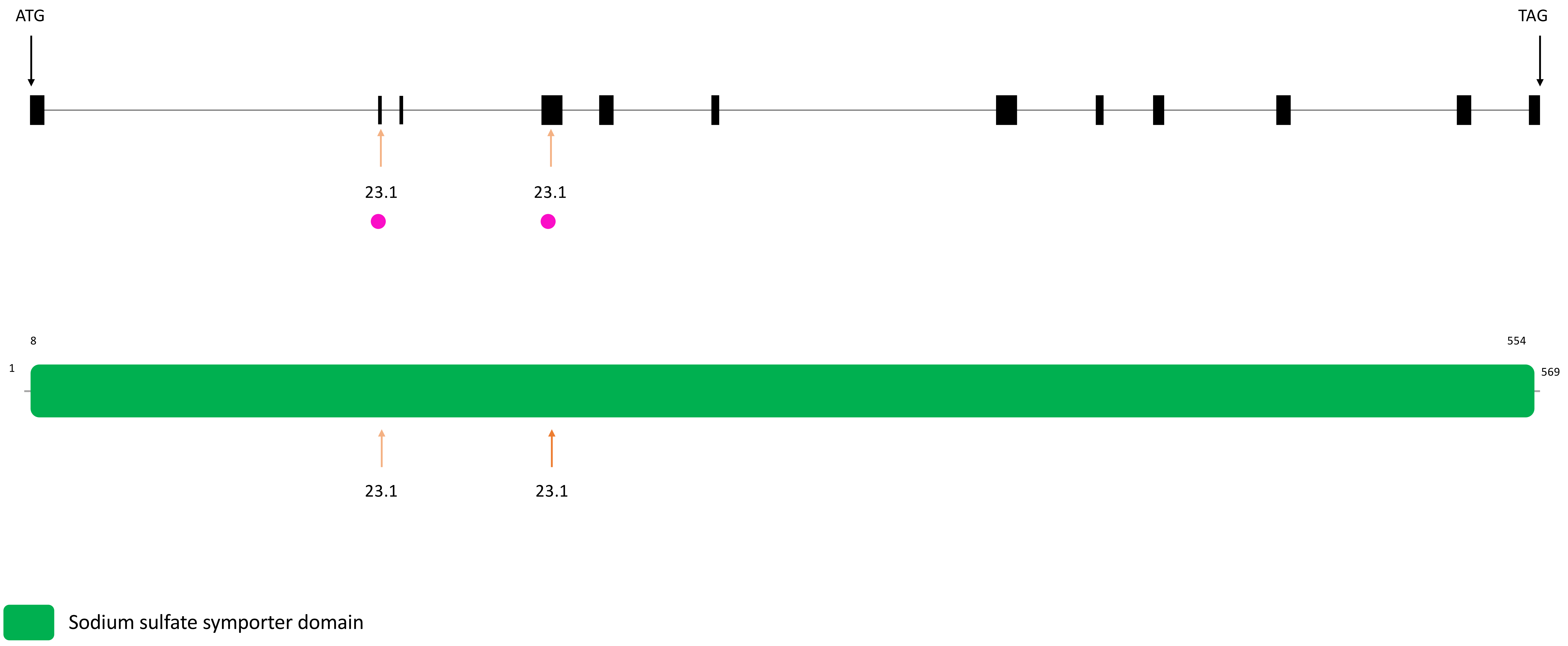


Supplementary Figure 2T

*ROGDI*

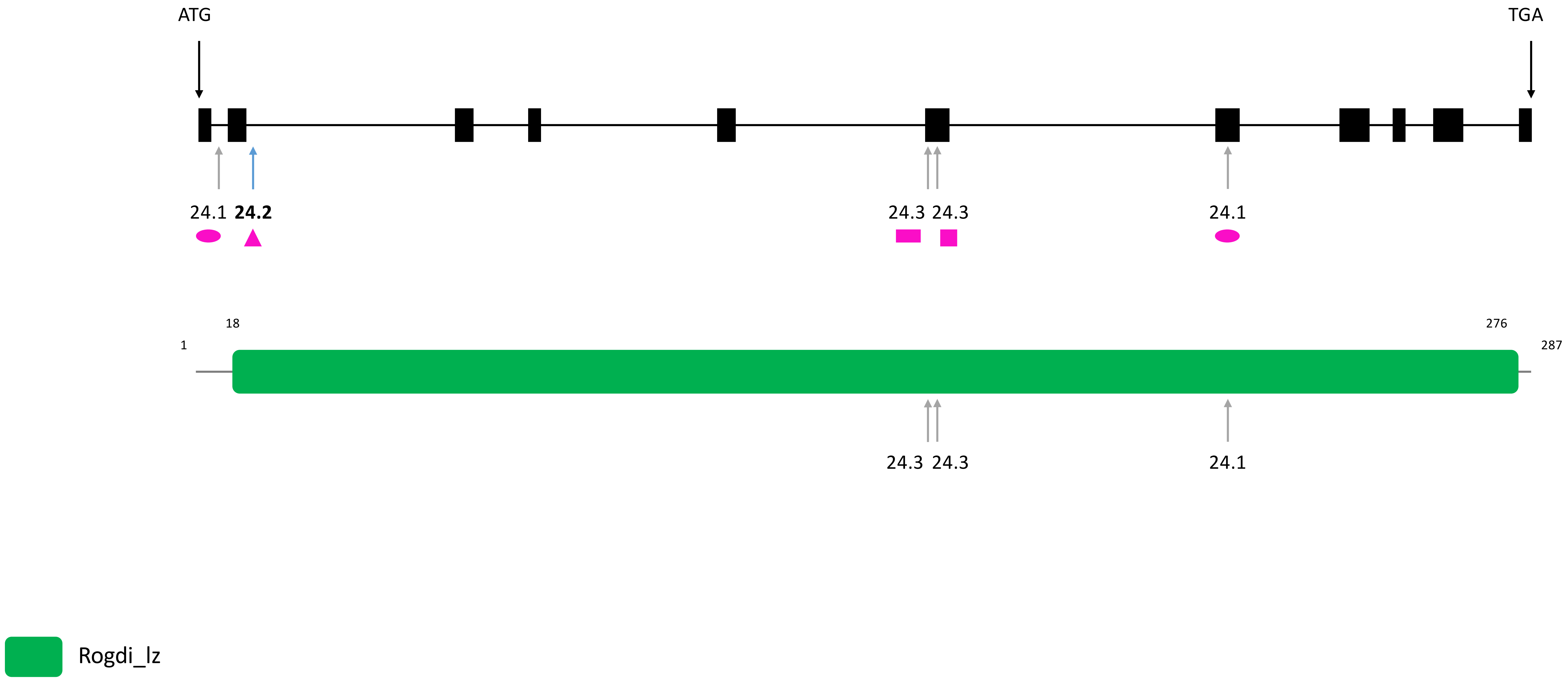


Supplementary Figure 2U

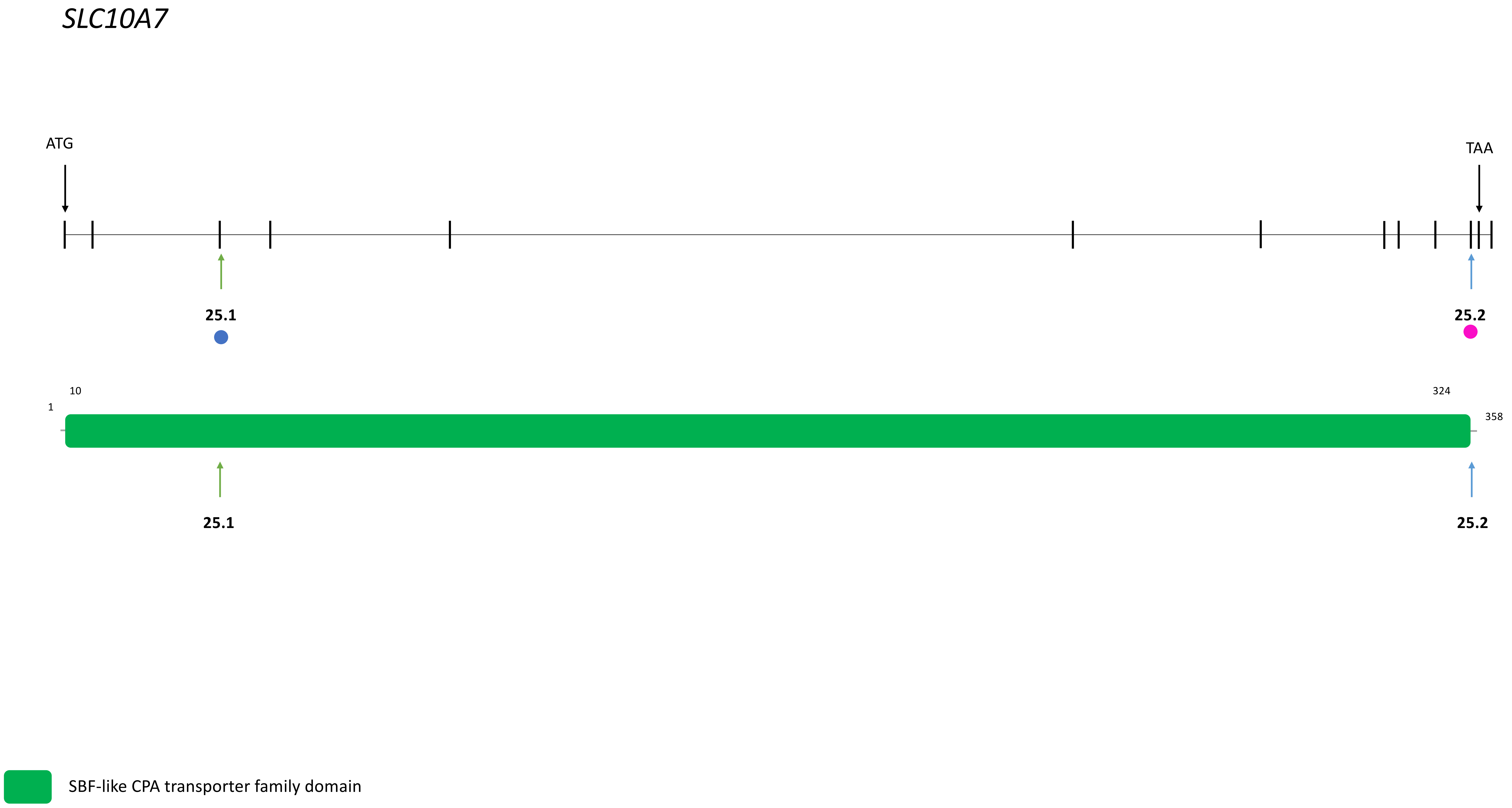


Supplementary Figure 2V

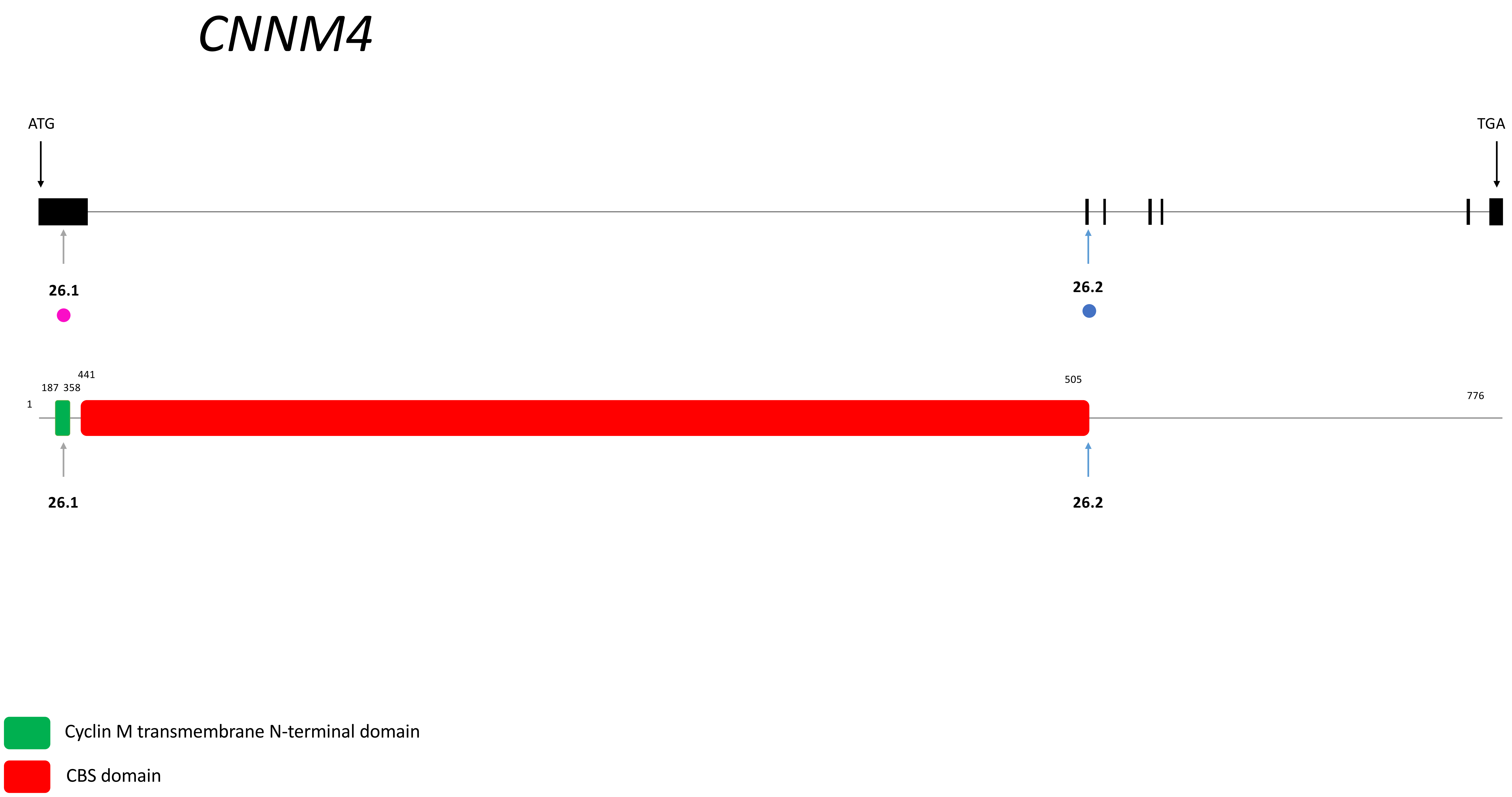


Supplementary Figure 2W
