# Supplementary material for: Amelogenesis imperfecta: Next-generation sequencing sheds light on Witkop’s classification
Source: Front Physiol. 2023 May 9;14:1130175. doi: 10.3389/fphys.2023.1130175 (PMC10205041; doi:10.3389/fphys.2023.1130175)
Supplement: Supplementary file 3 [file Presentation1.zip › Supplementary Figure 3.PPTX]

## Slide 1
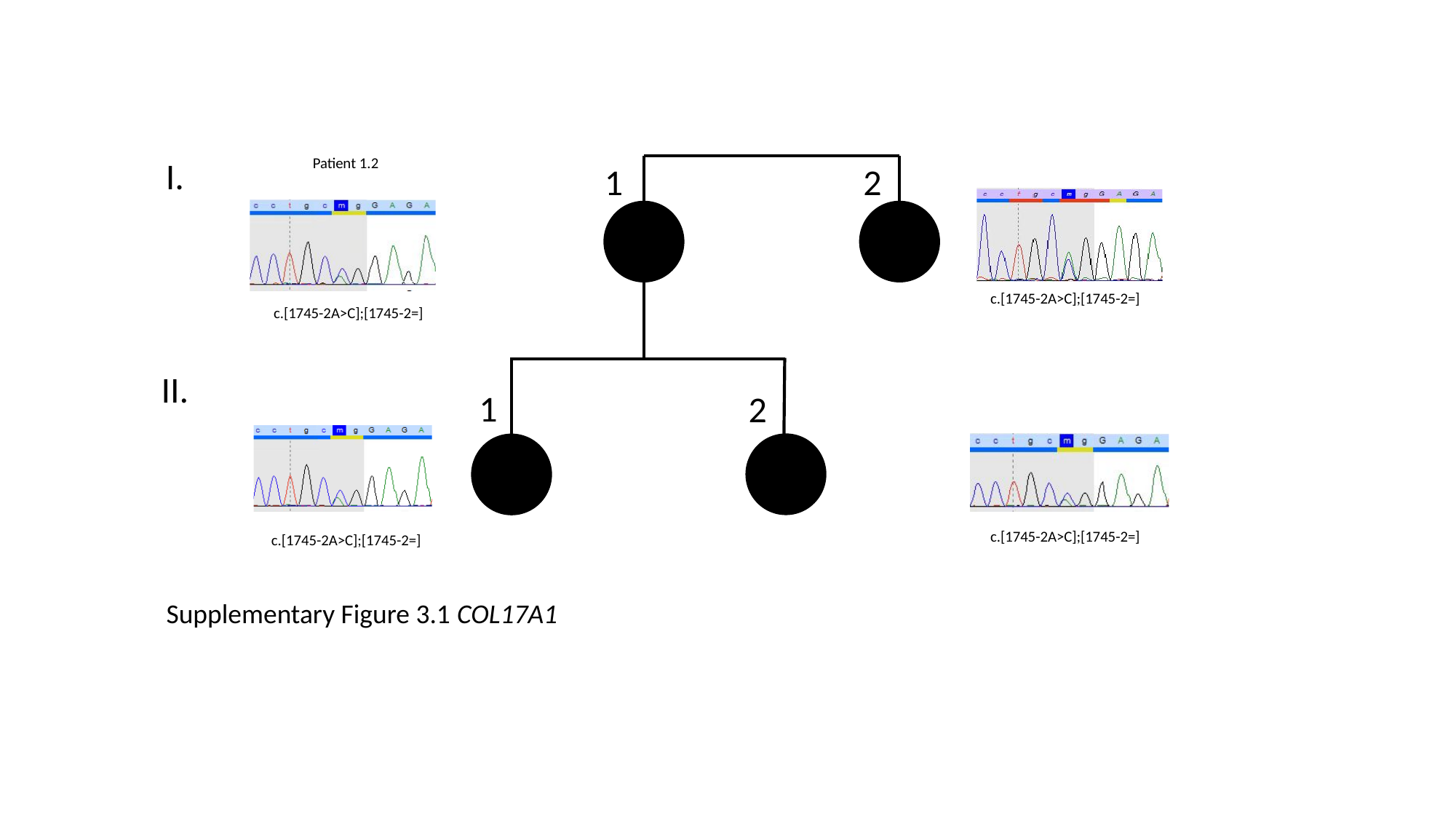

I.
Patient 1.2
1
2
c.[1745-2A>C];[1745-2=]
c.[1745-2A>C];[1745-2=]
II.
1
2
c.[1745-2A>C];[1745-2=]
c.[1745-2A>C];[1745-2=]
Supplementary Figure 3.1 COL17A1

## Slide 2
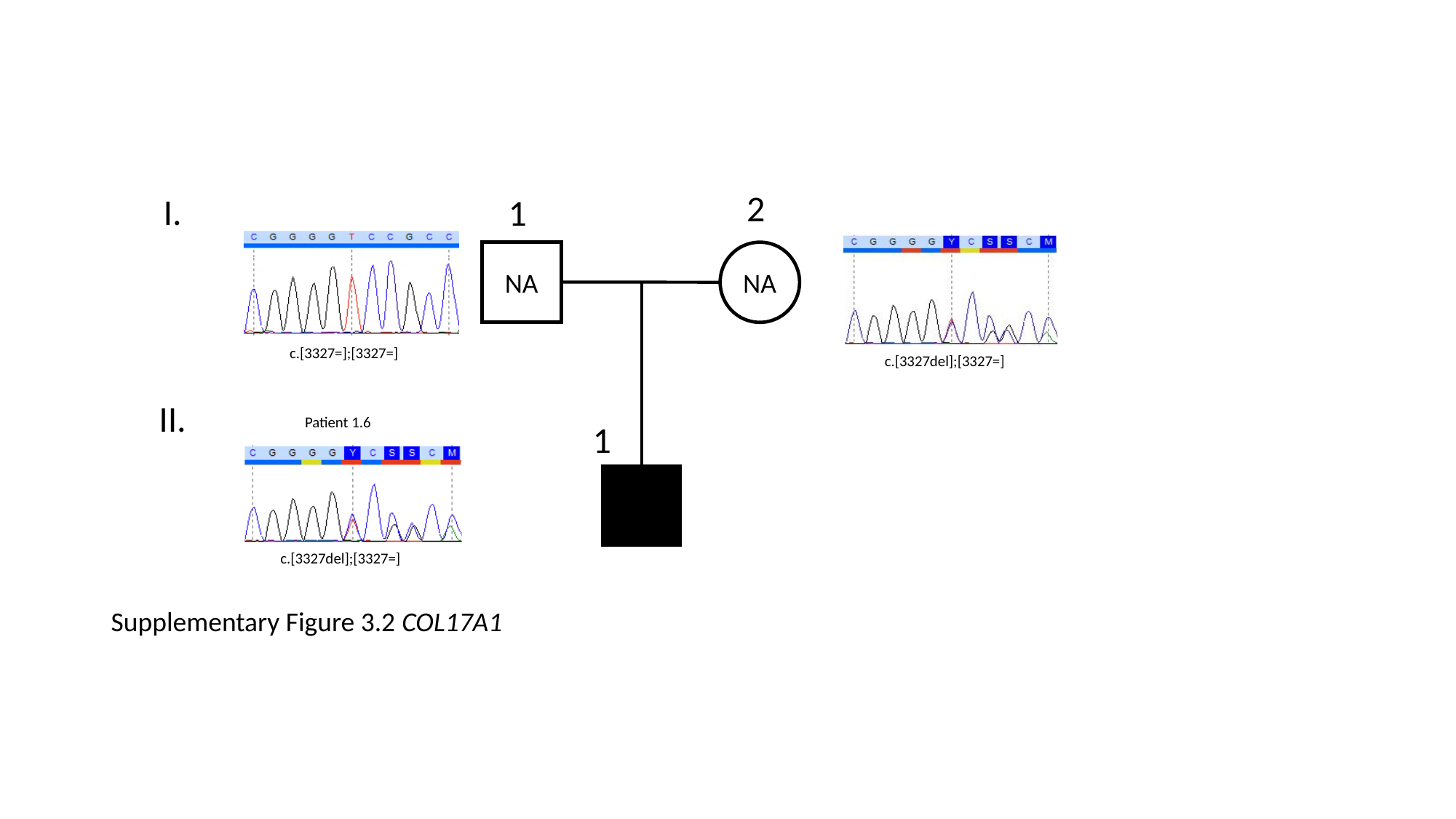

2
I.
1
NA
NA
c.[3327=];[3327=]
c.[3327del];[3327=]
II.
Patient 1.6
1
c.[3327del];[3327=]
Supplementary Figure 3.2 COL17A1

## Slide 3
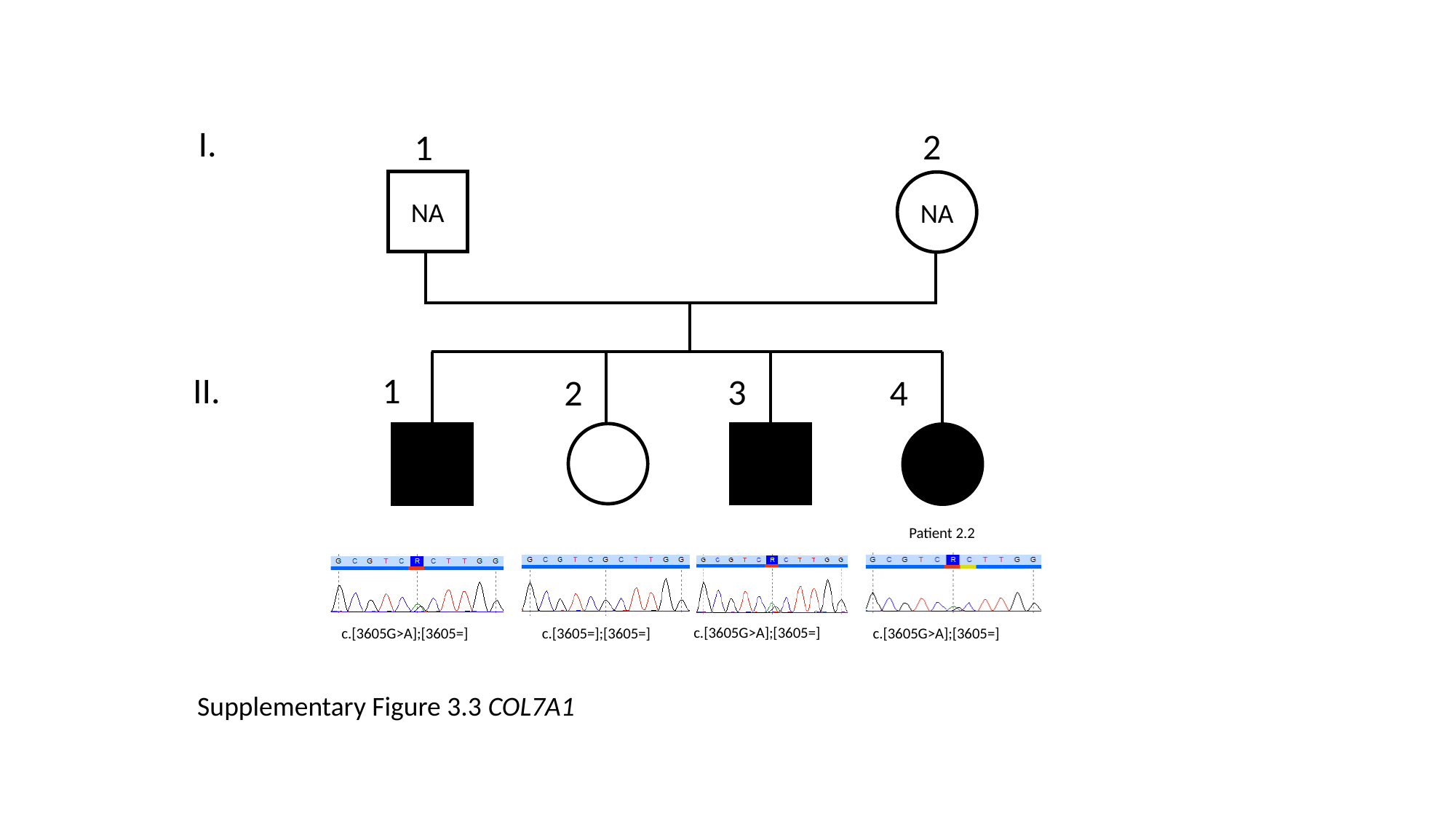

I.
2
1
NA
NA
II.
1
3
2
4
Patient 2.2
c.[3605G>A];[3605=]
c.[3605=];[3605=]
c.[3605G>A];[3605=]
c.[3605G>A];[3605=]
Supplementary Figure 3.3 COL7A1

## Slide 4
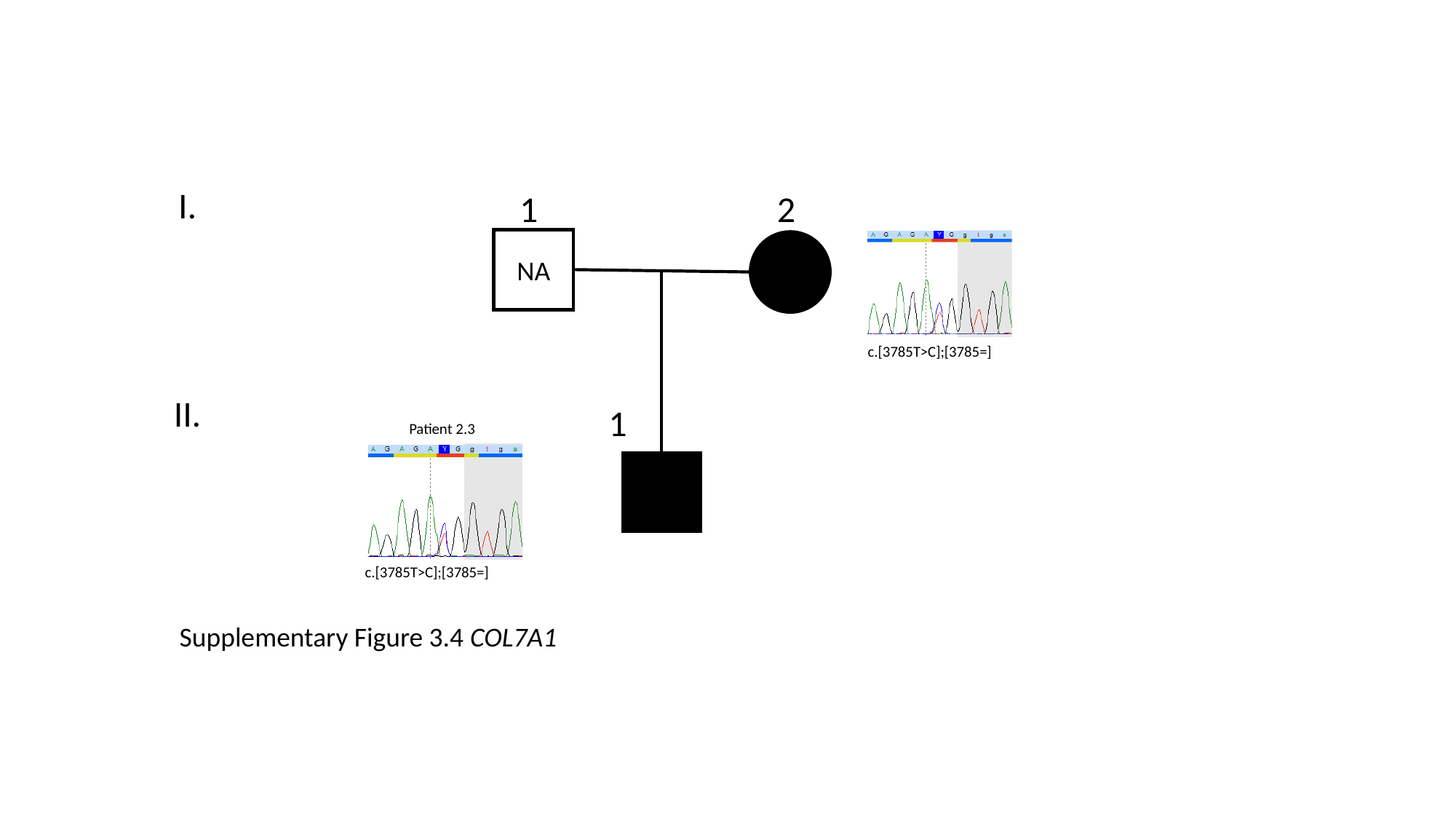

I.
1
2
NA
c.[3785T>C];[3785=]
II.
1
Patient 2.3
c.[3785T>C];[3785=]
Supplementary Figure 3.4 COL7A1

## Slide 5
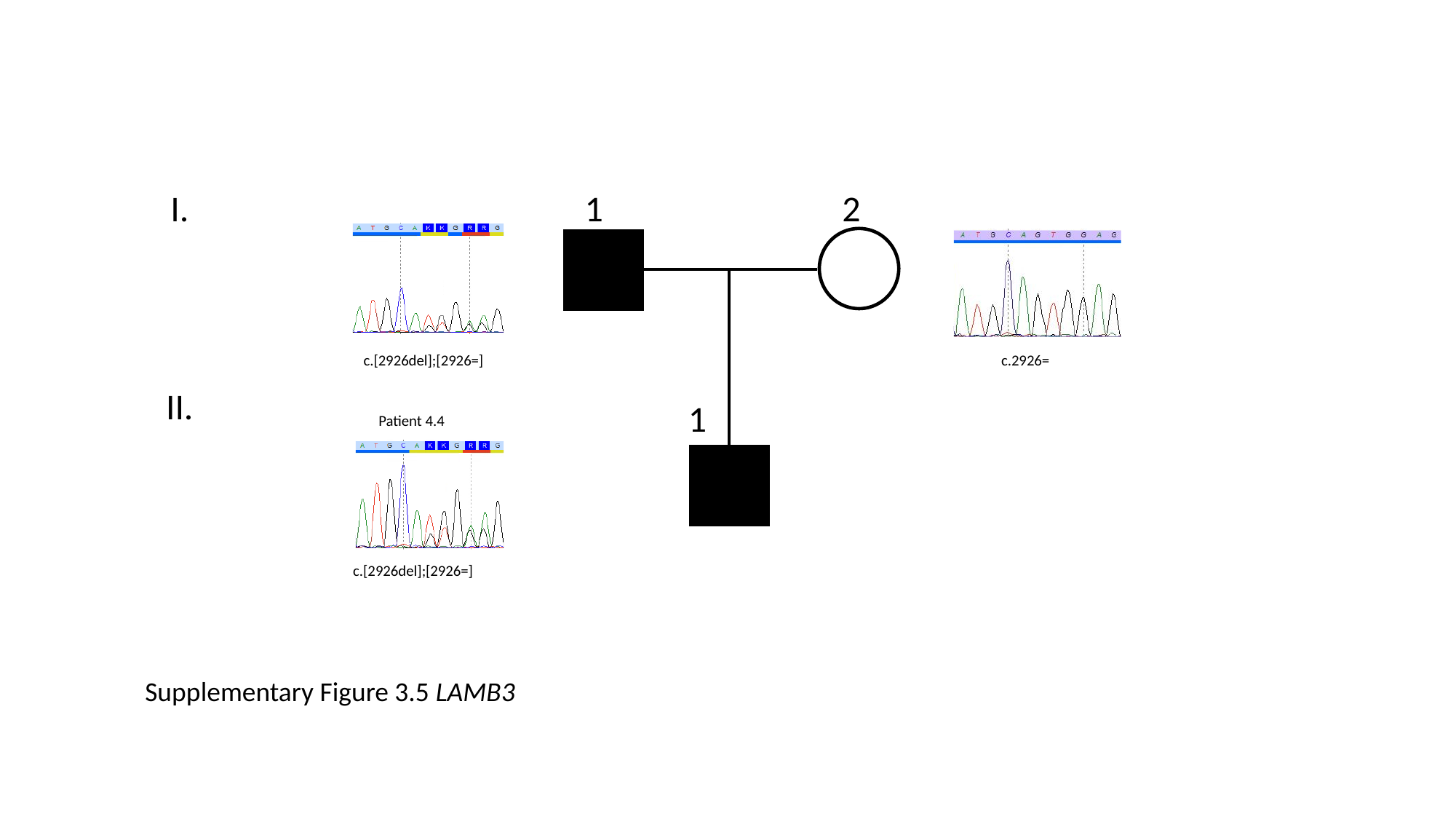

I.
1
2
c.[2926del];[2926=]
c.2926=
II.
1
Patient 4.4
c.[2926del];[2926=]
Supplementary Figure 3.5 LAMB3

## Slide 6
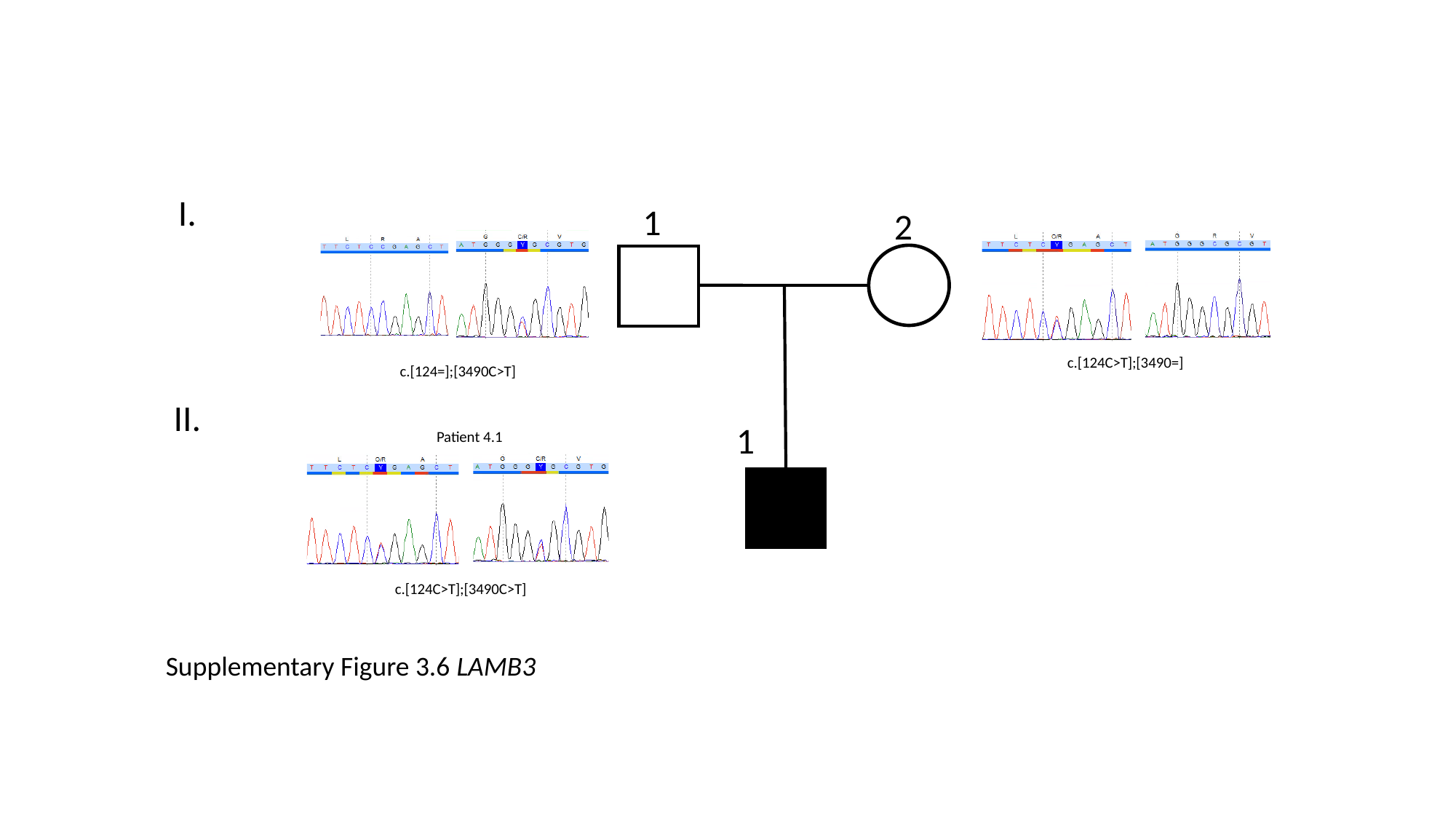

I.
1
2
c.[124C>T];[3490=]
c.[124=];[3490C>T]
II.
1
Patient 4.1
c.[124C>T];[3490C>T]
Supplementary Figure 3.6 LAMB3

## Slide 7
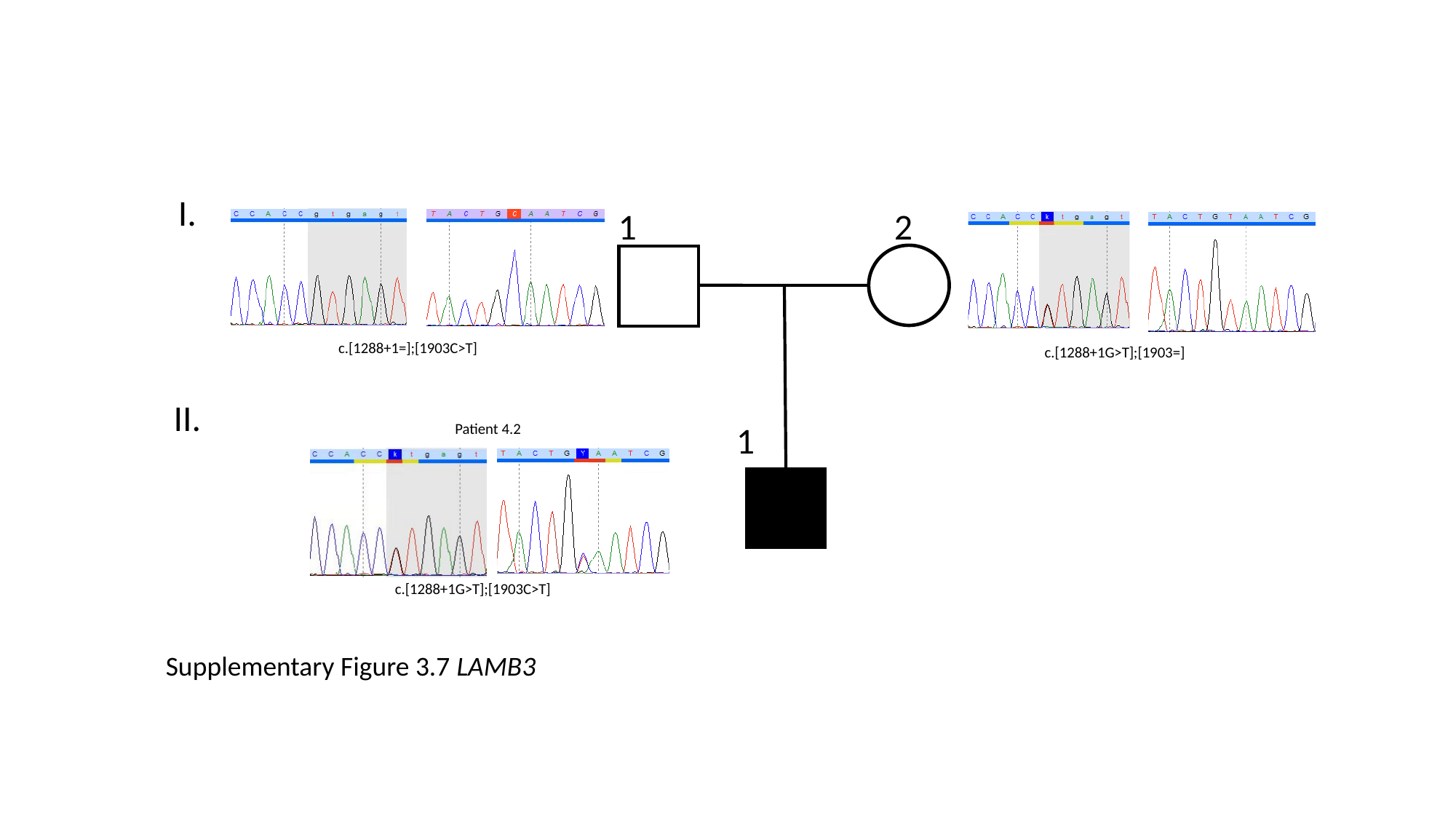

I.
1
2
c.[1288+1=];[1903C>T]
c.[1288+1G>T];[1903=]
II.
1
Patient 4.2
c.[1288+1G>T];[1903C>T]
Supplementary Figure 3.7 LAMB3

## Slide 8
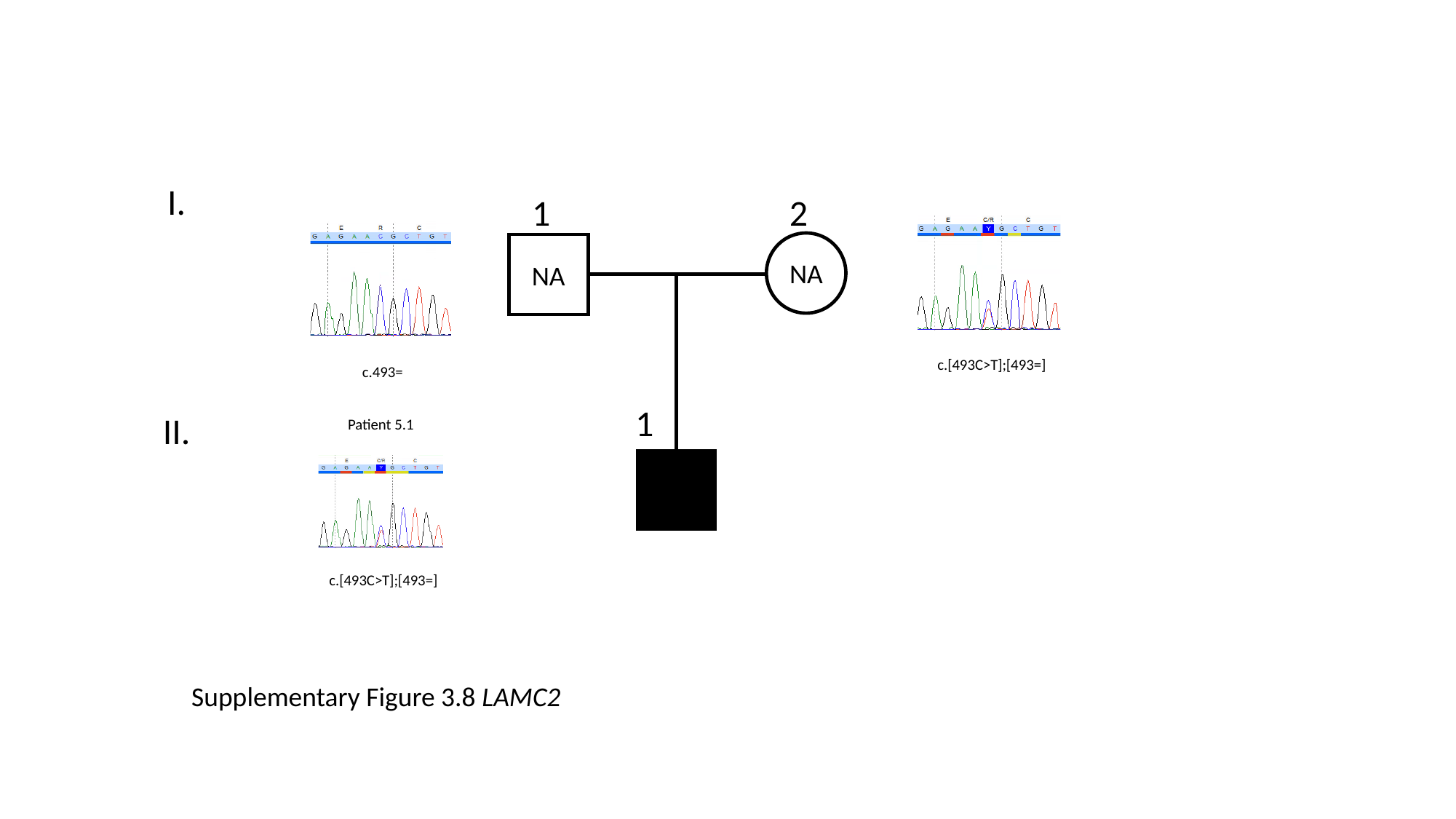

I.
1
2
NA
NA
c.[493C>T];[493=]
c.493=
1
II.
Patient 5.1
c.[493C>T];[493=]
Supplementary Figure 3.8 LAMC2

## Slide 9
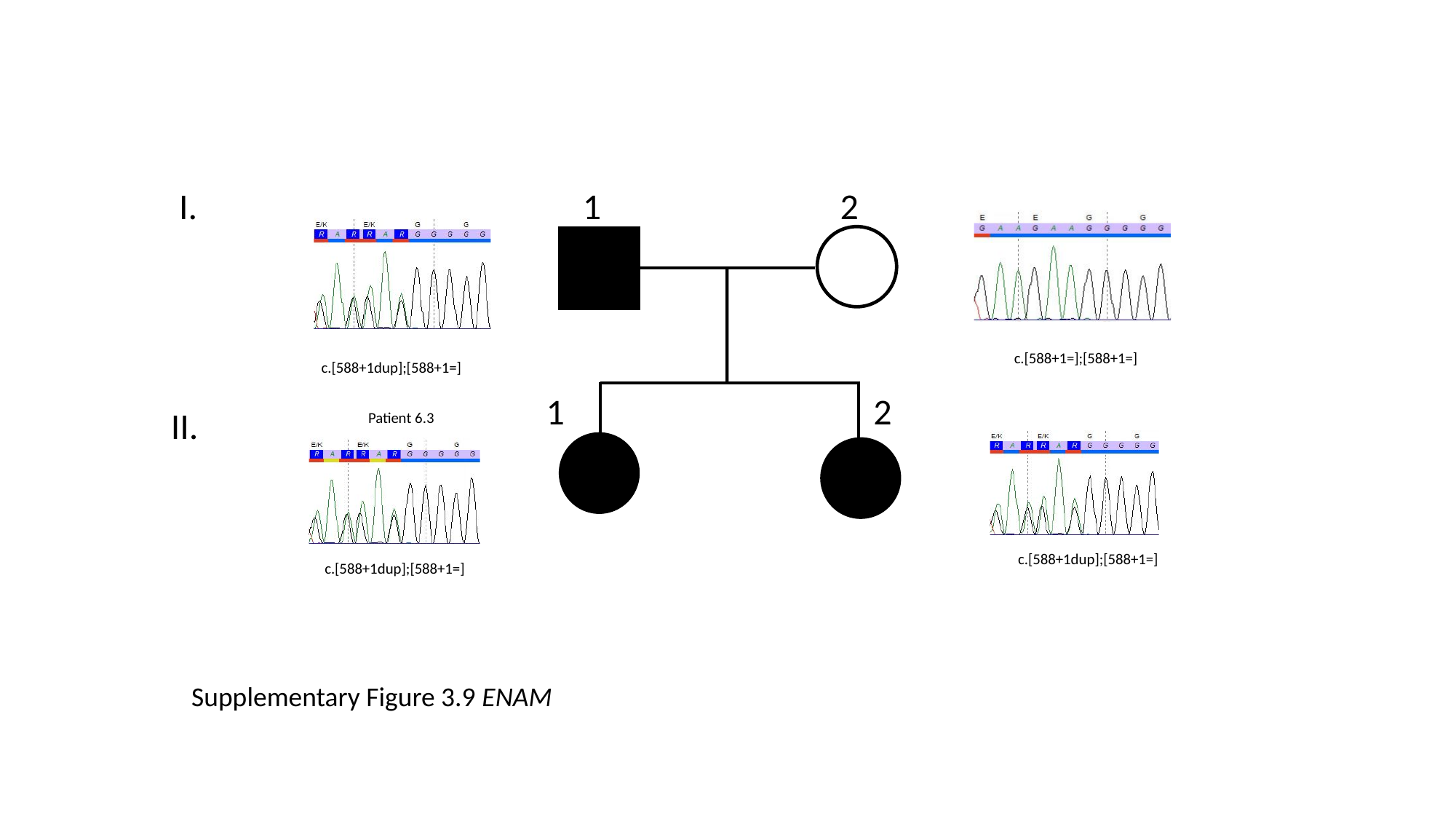

I.
1
2
c.[588+1=];[588+1=]
c.[588+1dup];[588+1=]
1
2
II.
Patient 6.3
c.[588+1dup];[588+1=]
c.[588+1dup];[588+1=]
Supplementary Figure 3.9 ENAM

## Slide 10
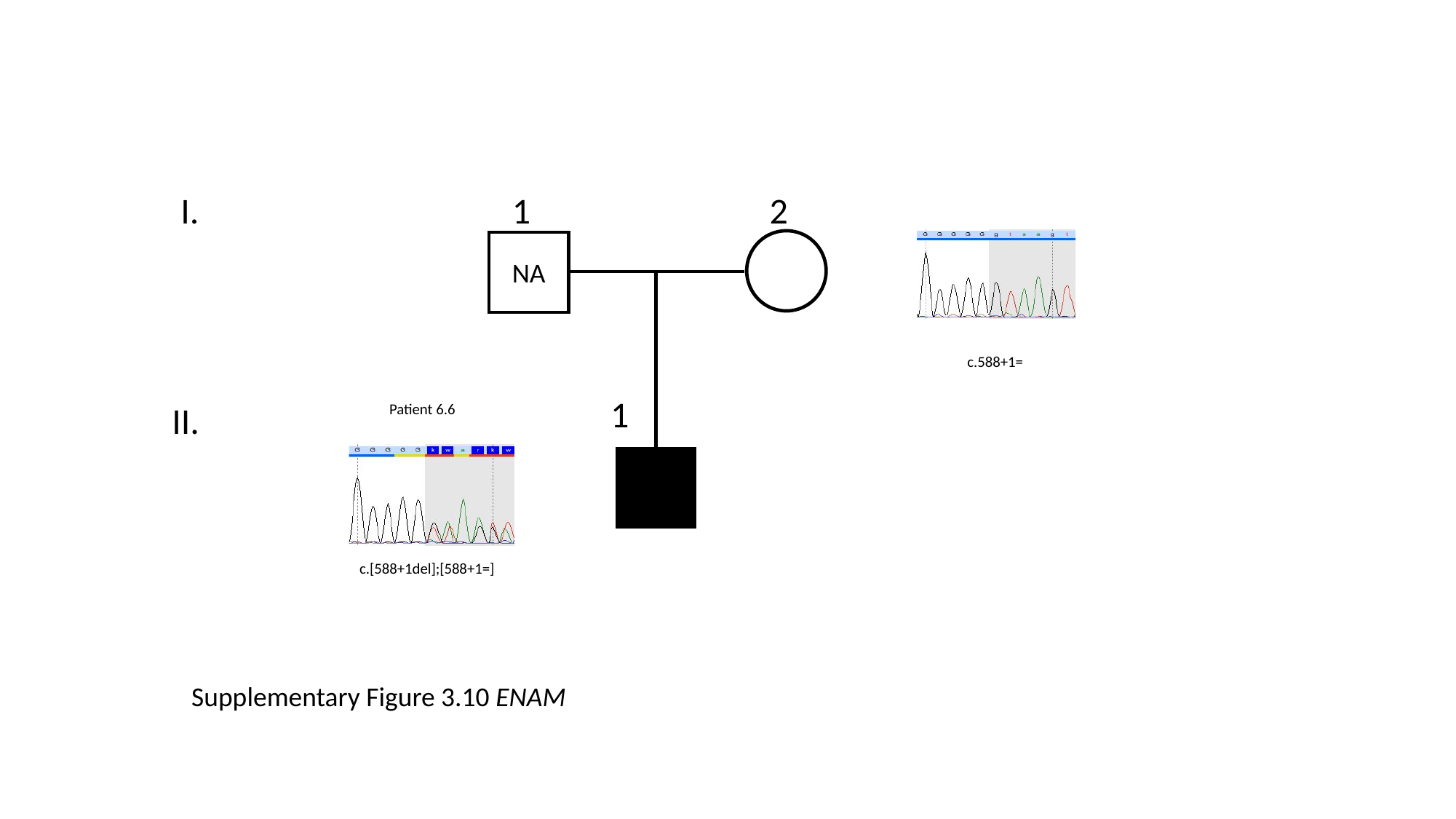

I.
1
2
NA
c.588+1=
1
II.
Patient 6.6
c.[588+1del];[588+1=]
Supplementary Figure 3.10 ENAM

## Slide 11
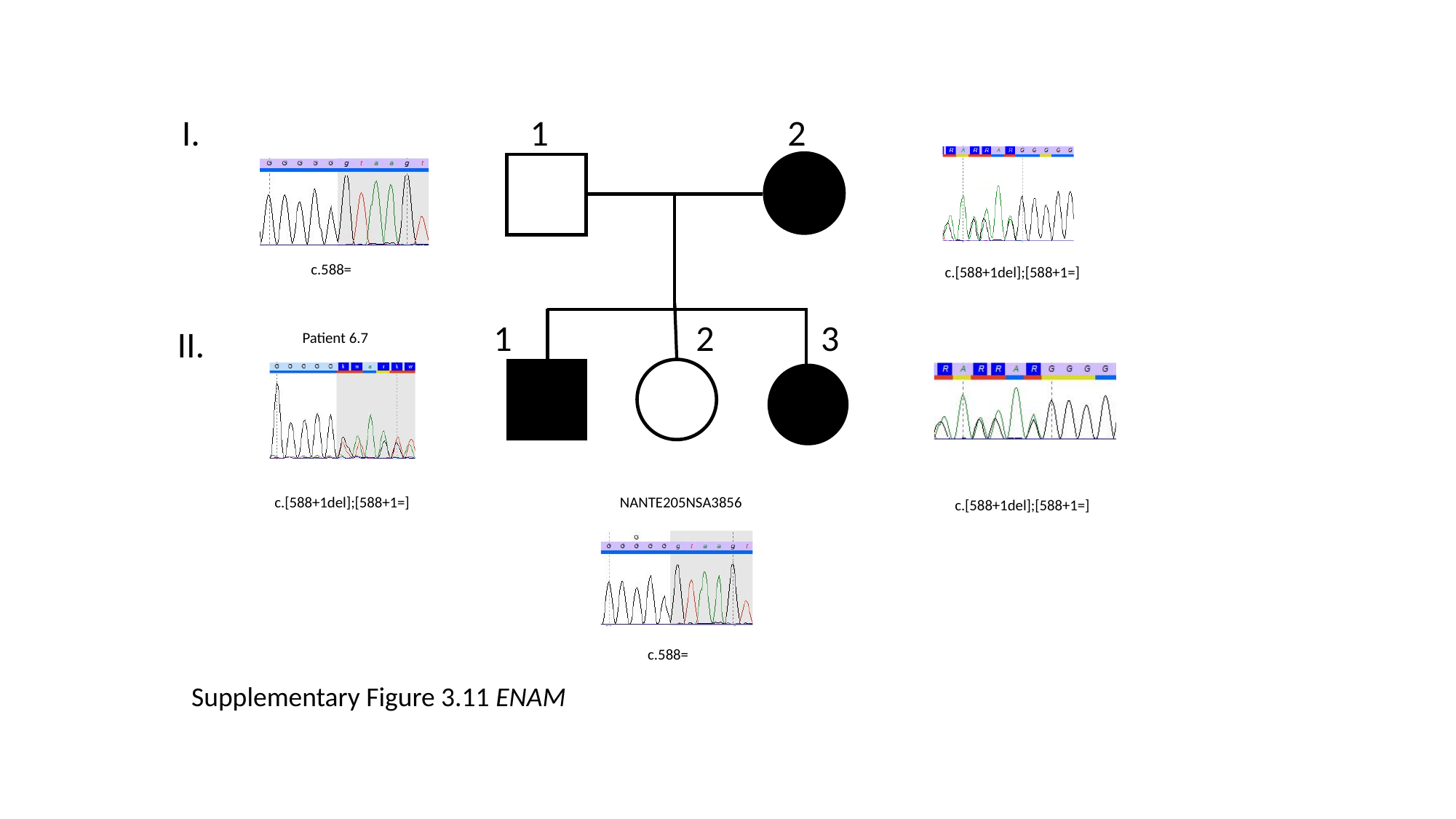

I.
1
2
c.588=
c.[588+1del];[588+1=]
1
2
3
II.
Patient 6.7
c.[588+1del];[588+1=]
NANTE205NSA3856
c.[588+1del];[588+1=]
c.588=
Supplementary Figure 3.11 ENAM

## Slide 12
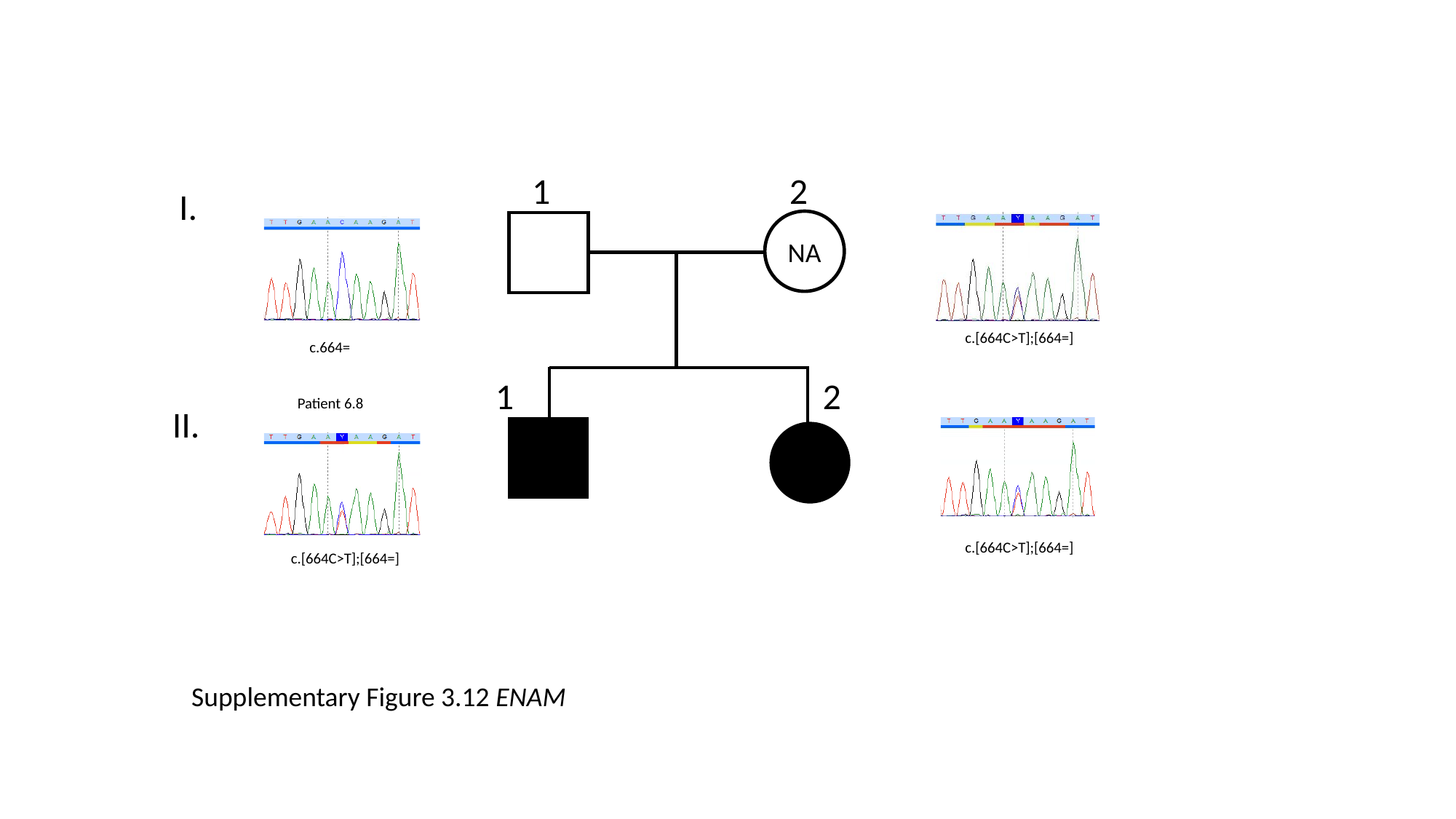

1
2
I.
NA
c.[664C>T];[664=]
c.664=
1
2
Patient 6.8
II.
c.[664C>T];[664=]
c.[664C>T];[664=]
Supplementary Figure 3.12 ENAM

## Slide 13
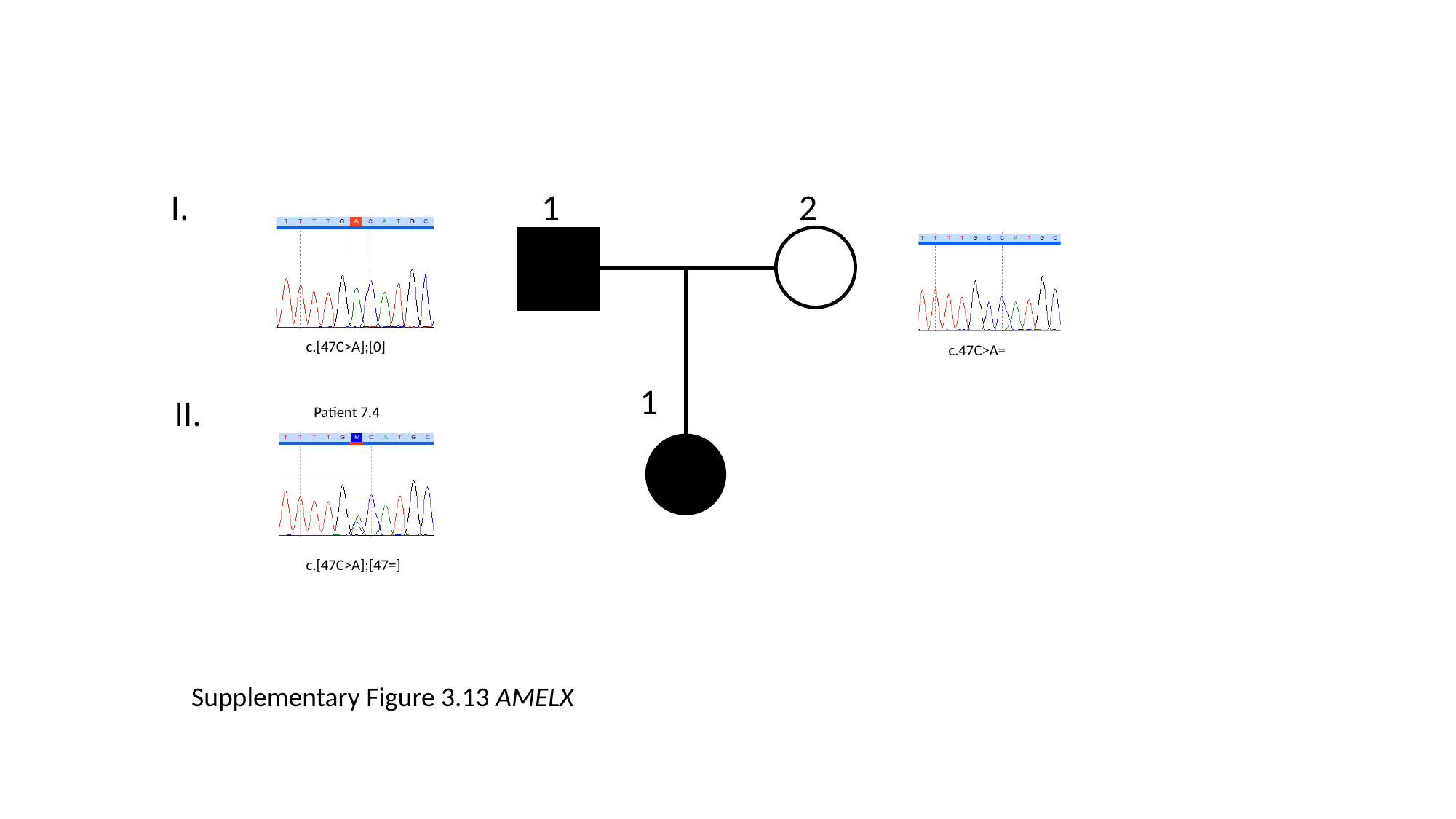

I.
1
2
c.[47C>A];[0]
c.47C>A=
1
II.
Patient 7.4
c.[47C>A];[47=]
Supplementary Figure 3.13 AMELX

## Slide 14
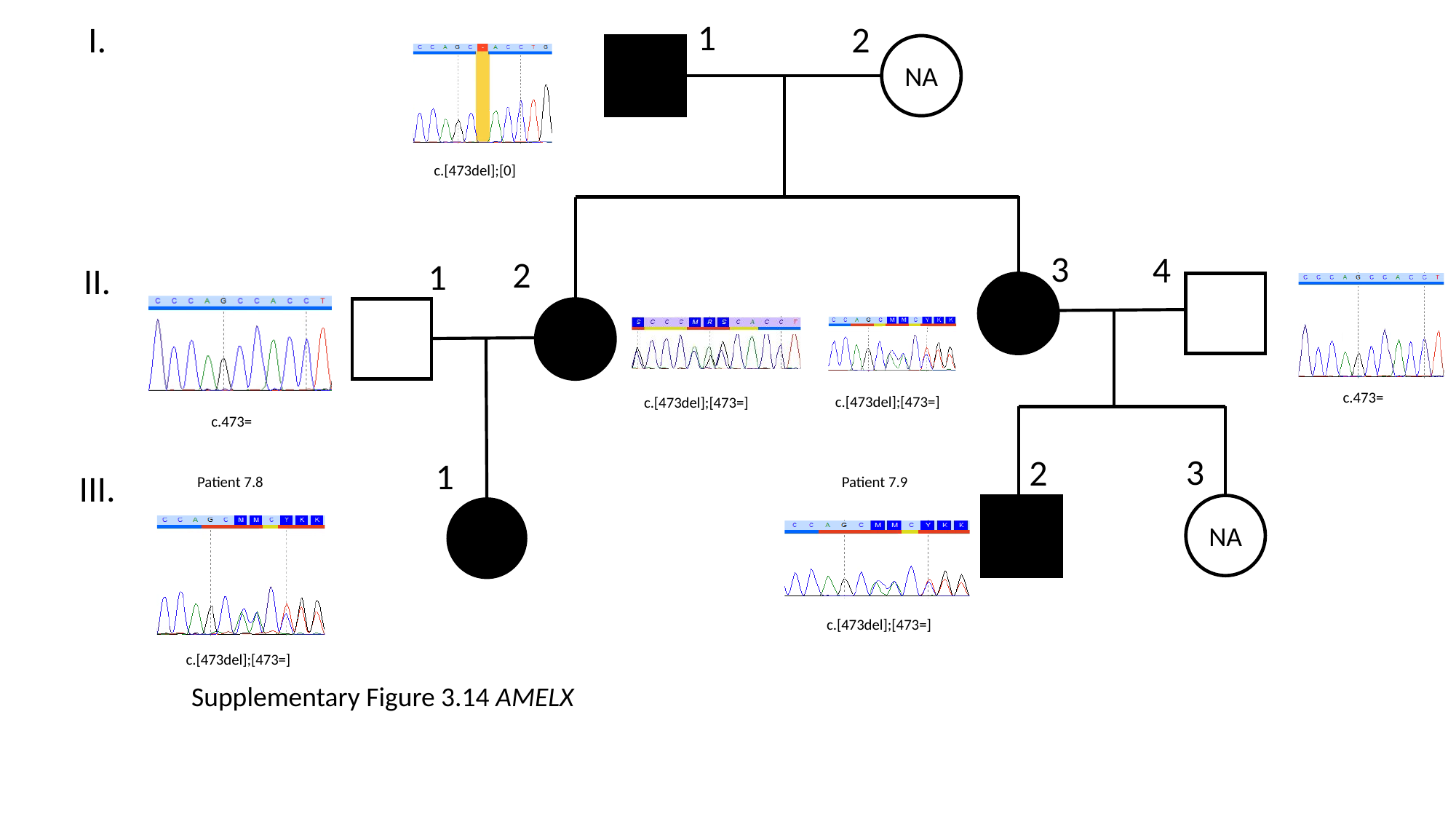

1
I.
2
NA
c.[473del];[0]
3
4
2
1
II.
c.473=
c.[473del];[473=]
c.[473del];[473=]
c.473=
3
2
1
III.
Patient 7.8
Patient 7.9
NA
c.[473del];[473=]
c.[473del];[473=]
Supplementary Figure 3.14 AMELX

## Slide 15
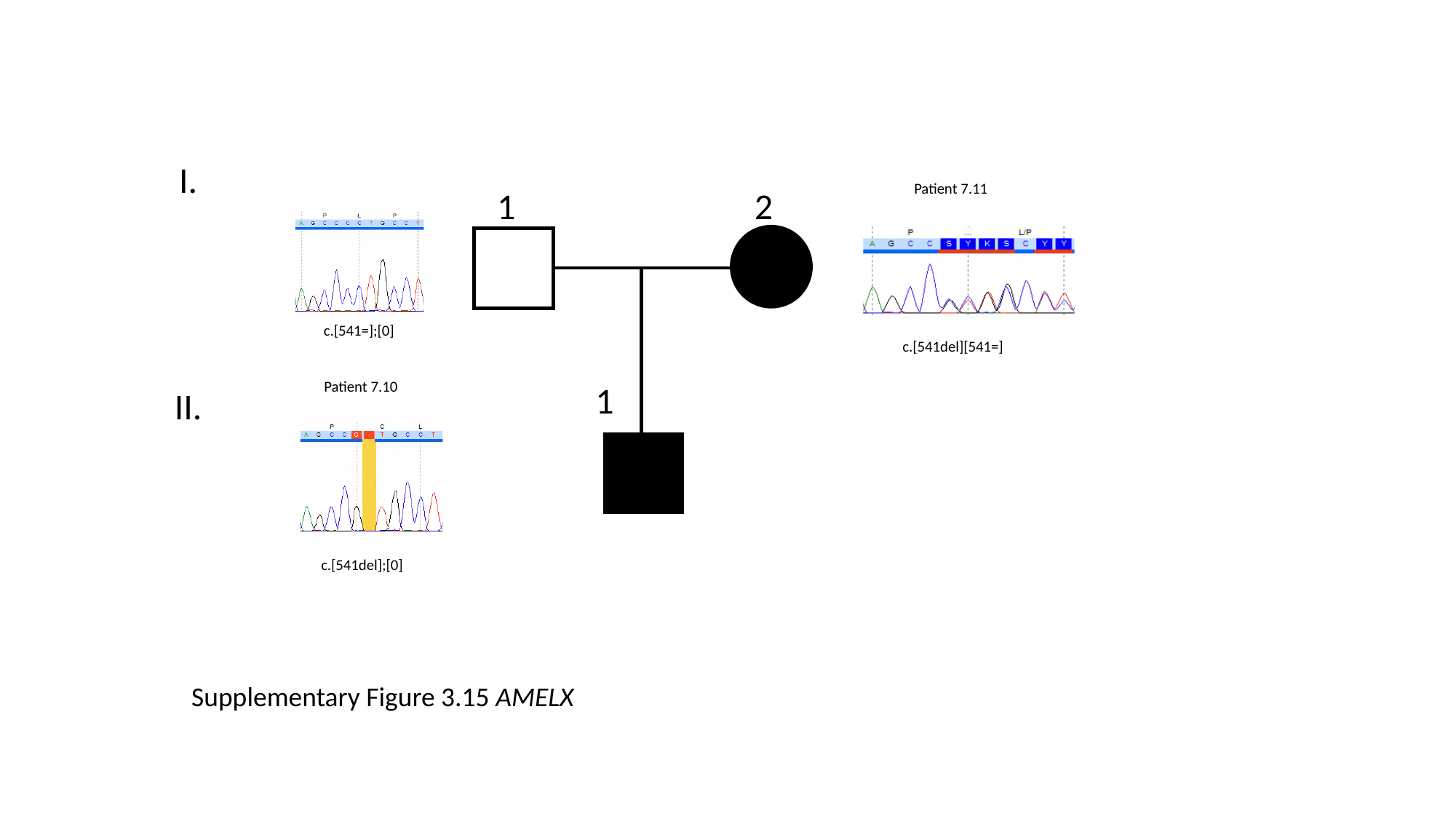

I.
Patient 7.11
1
2
c.[541=];[0]
c.[541del][541=]
Patient 7.10
1
II.
c.[541del];[0]
Supplementary Figure 3.15 AMELX

## Slide 16
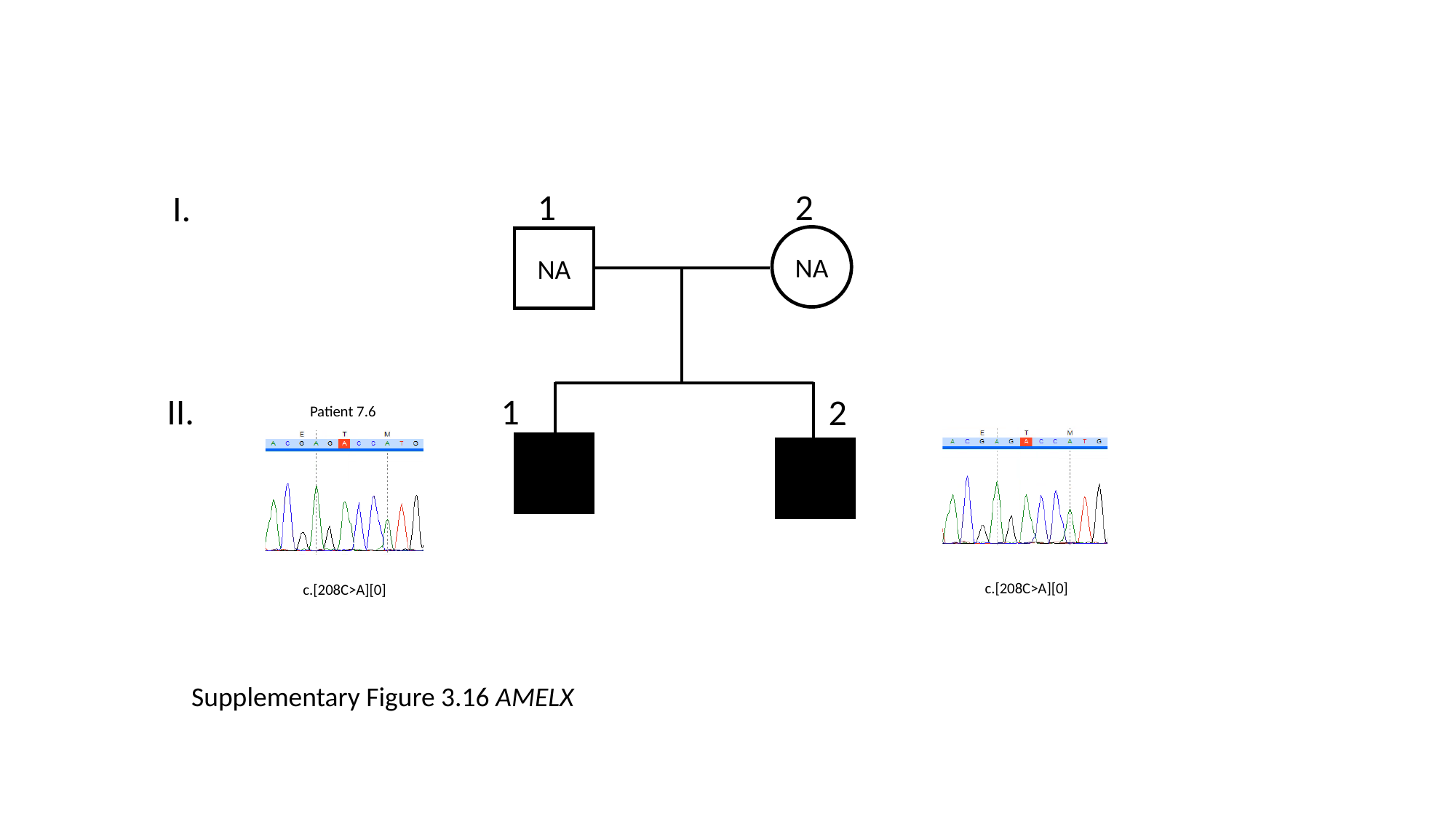

1
2
I.
NA
NA
II.
1
2
Patient 7.6
c.[208C>A][0]
c.[208C>A][0]
Supplementary Figure 3.16 AMELX

## Slide 17
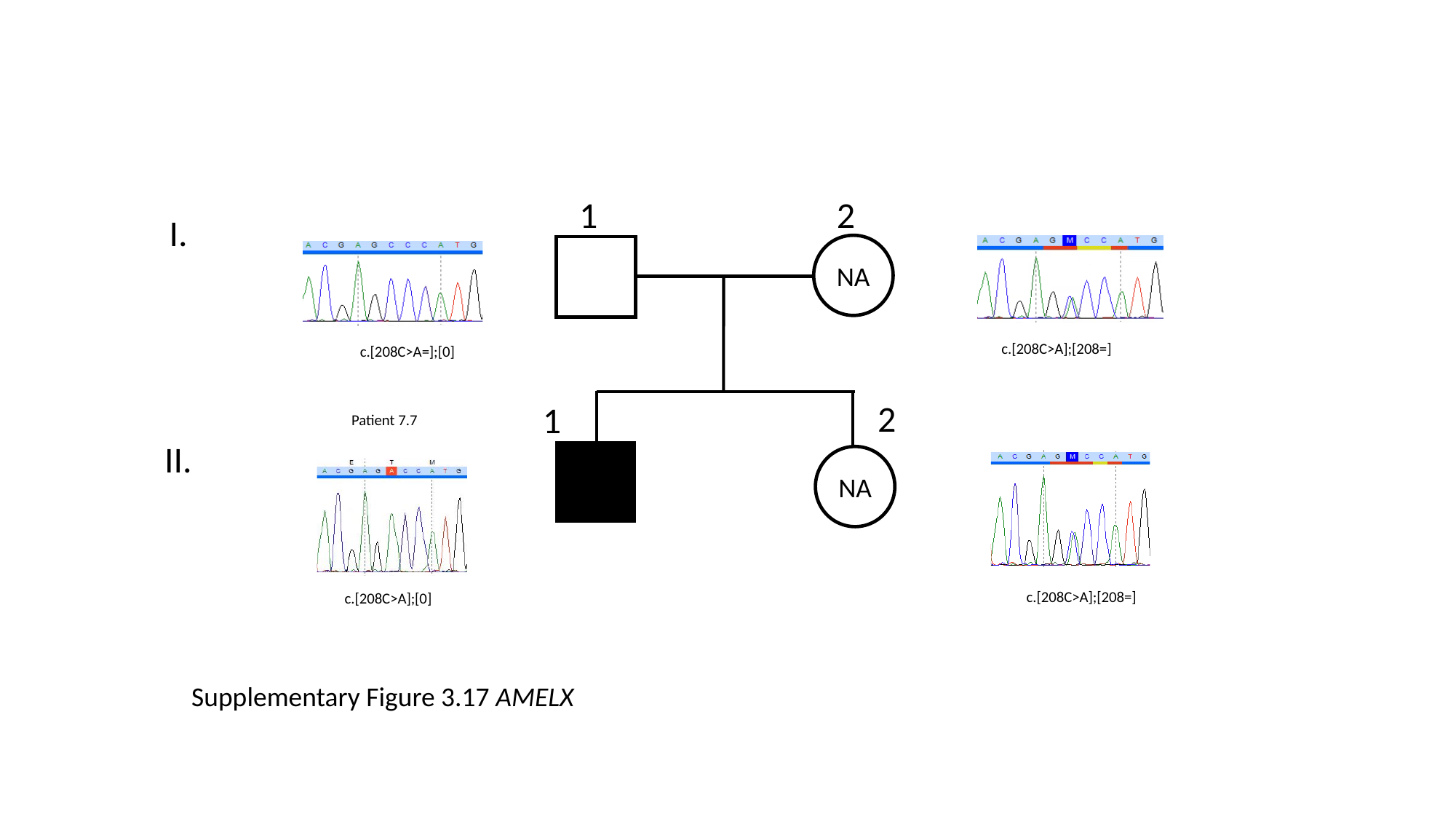

1
2
I.
NA
c.[208C>A];[208=]
c.[208C>A=];[0]
2
1
Patient 7.7
II.
NA
c.[208C>A];[208=]
c.[208C>A];[0]
Supplementary Figure 3.17 AMELX

## Slide 18
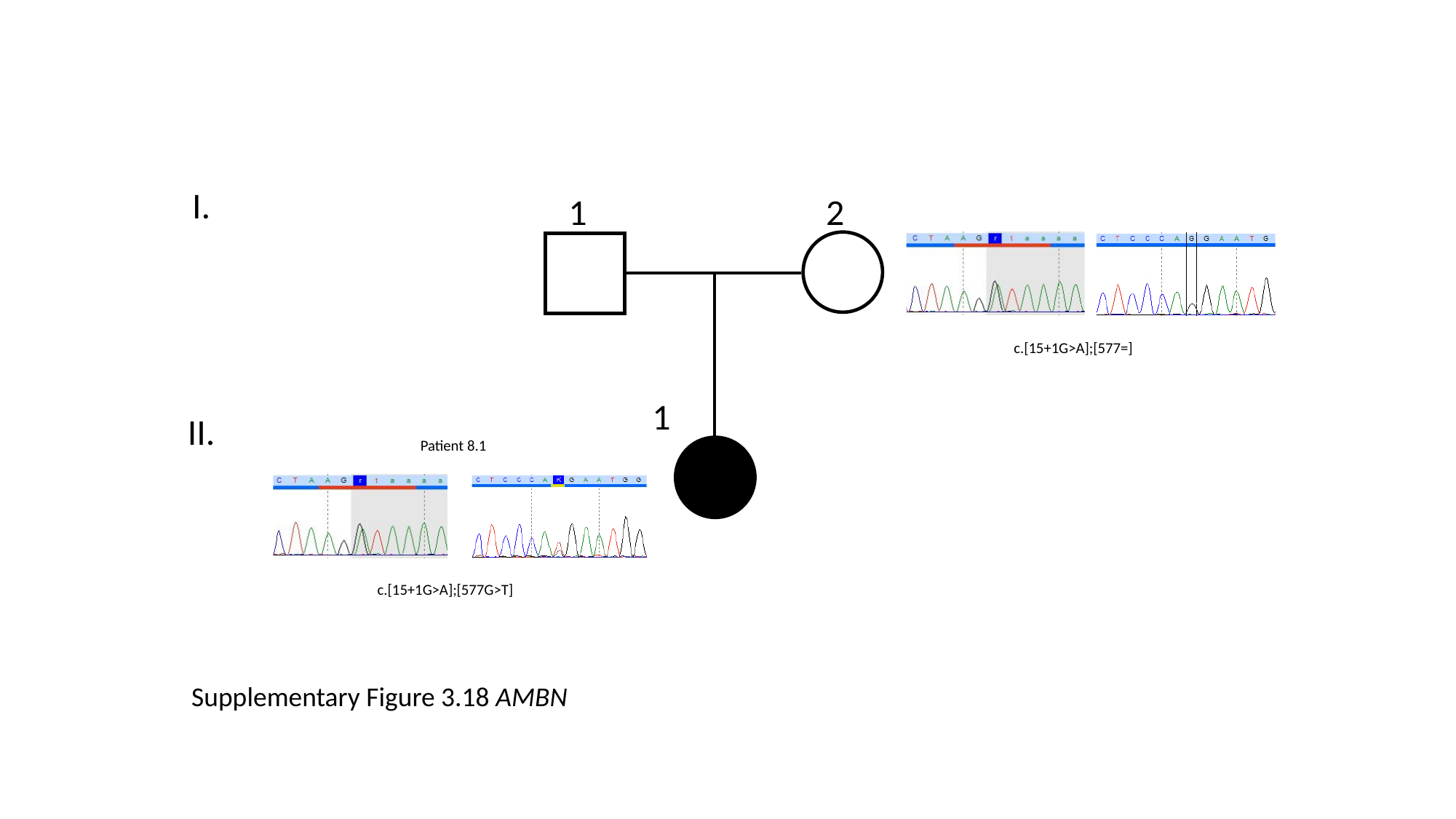

I.
1
2
c.[15+1G>A];[577=]
1
II.
Patient 8.1
c.[15+1G>A];[577G>T]
Supplementary Figure 3.18 AMBN

## Slide 19
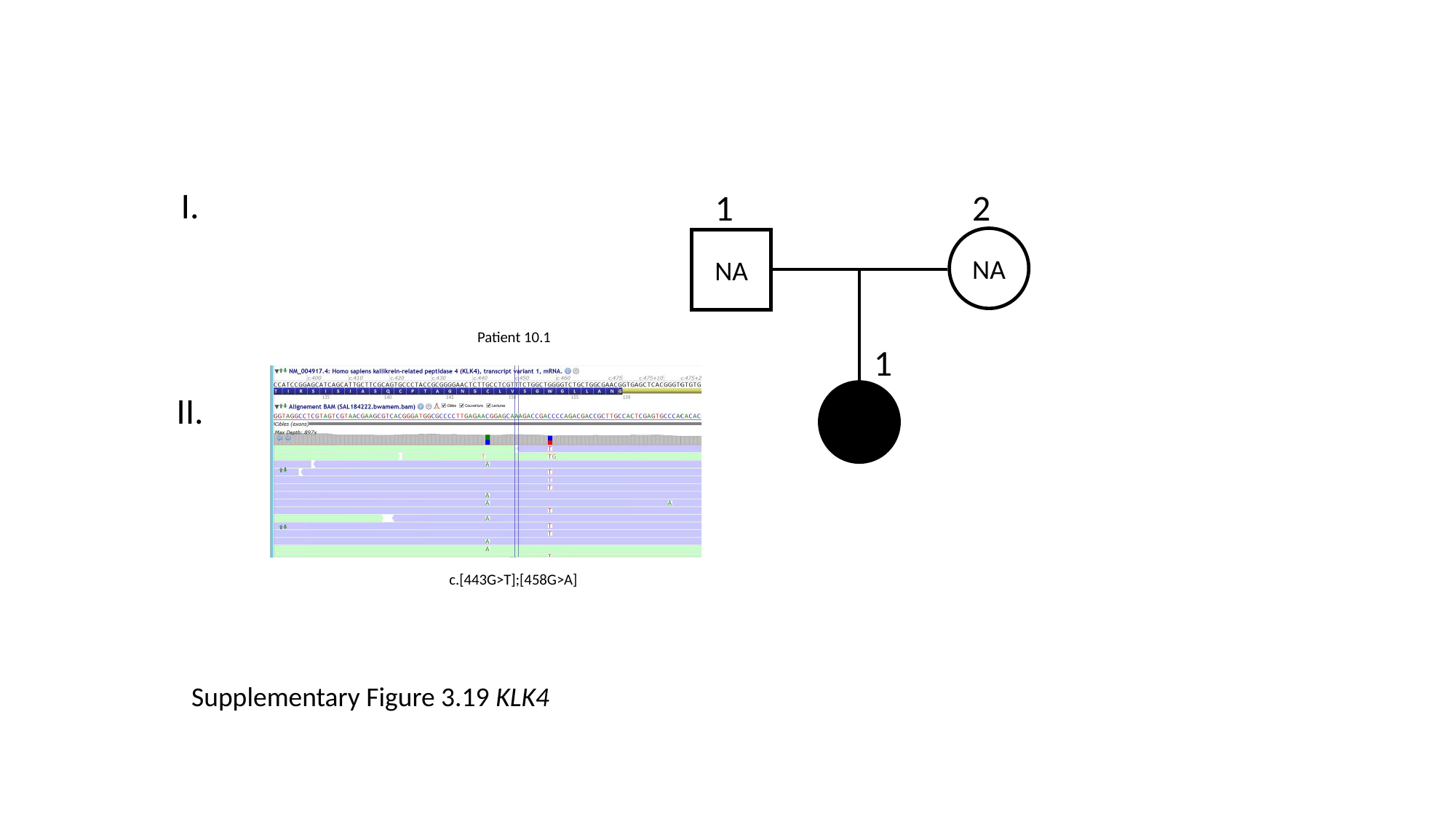

I.
1
2
NA
NA
Patient 10.1
1
II.
c.[443G>T];[458G>A]
Supplementary Figure 3.19 KLK4

## Slide 20
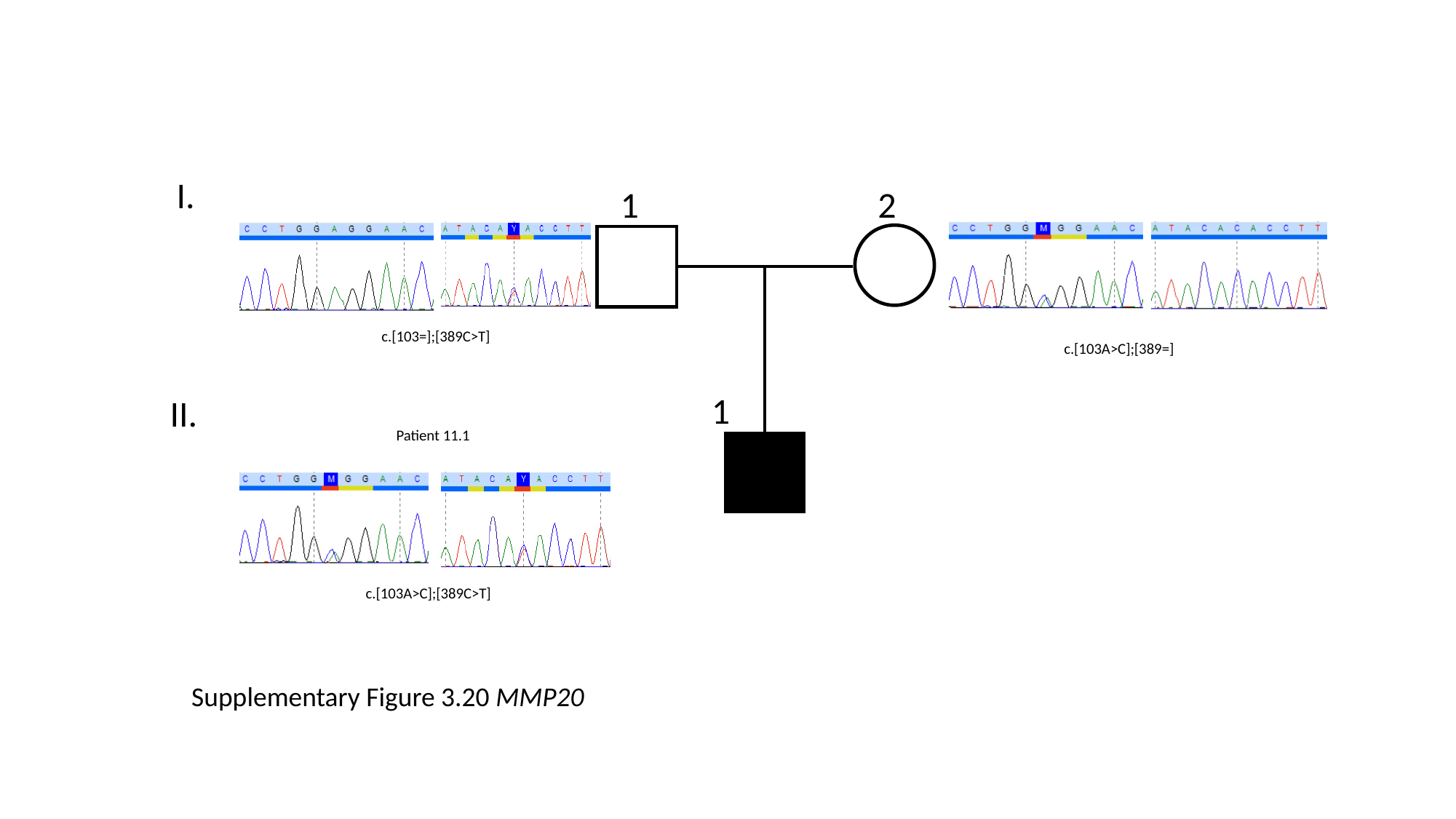

I.
1
2
c.[103=];[389C>T]
c.[103A>C];[389=]
1
II.
Patient 11.1
c.[103A>C];[389C>T]
Supplementary Figure 3.20 MMP20

## Slide 21
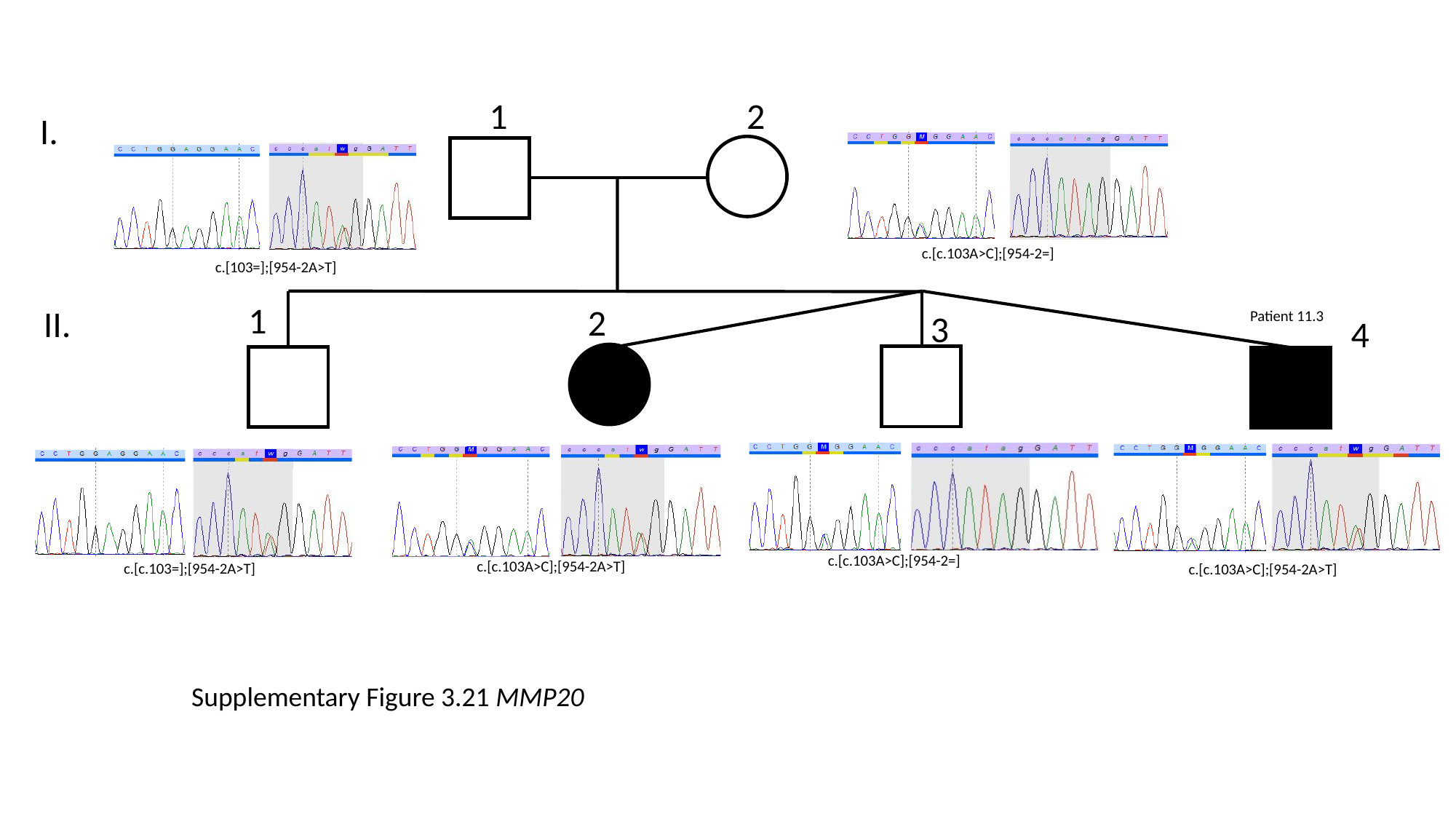

1
2
I.
c.[c.103A>C];[954-2=]
c.[103=];[954-2A>T]
1
2
II.
3
Patient 11.3
4
c.[c.103A>C];[954-2=]
c.[c.103A>C];[954-2A>T]
c.[c.103=];[954-2A>T]
c.[c.103A>C];[954-2A>T]
Supplementary Figure 3.21 MMP20

## Slide 22
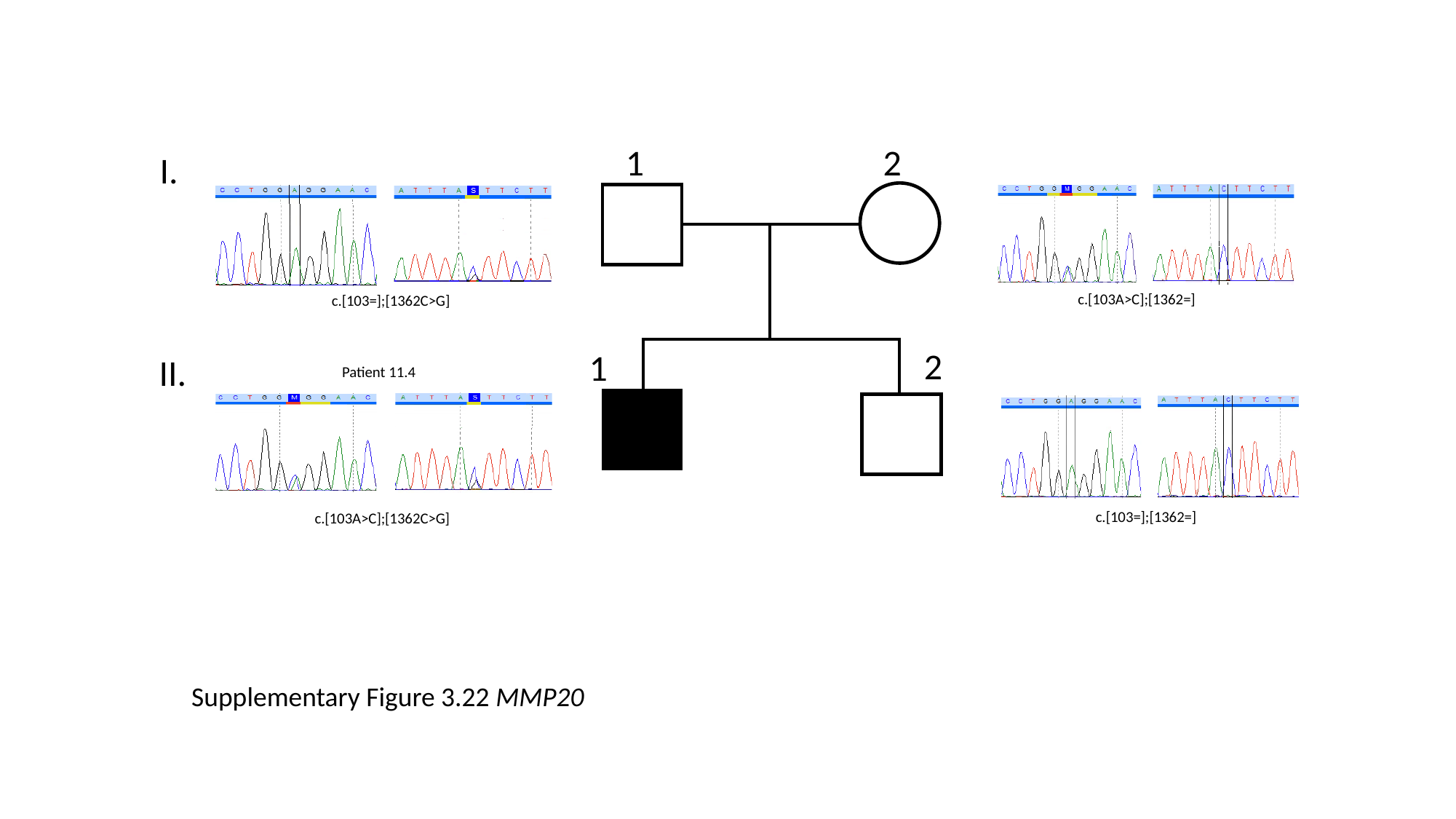

1
2
I.
c.[103A>C];[1362=]
c.[103=];[1362C>G]
2
1
II.
Patient 11.4
c.[103=];[1362=]
c.[103A>C];[1362C>G]
Supplementary Figure 3.22 MMP20

## Slide 23
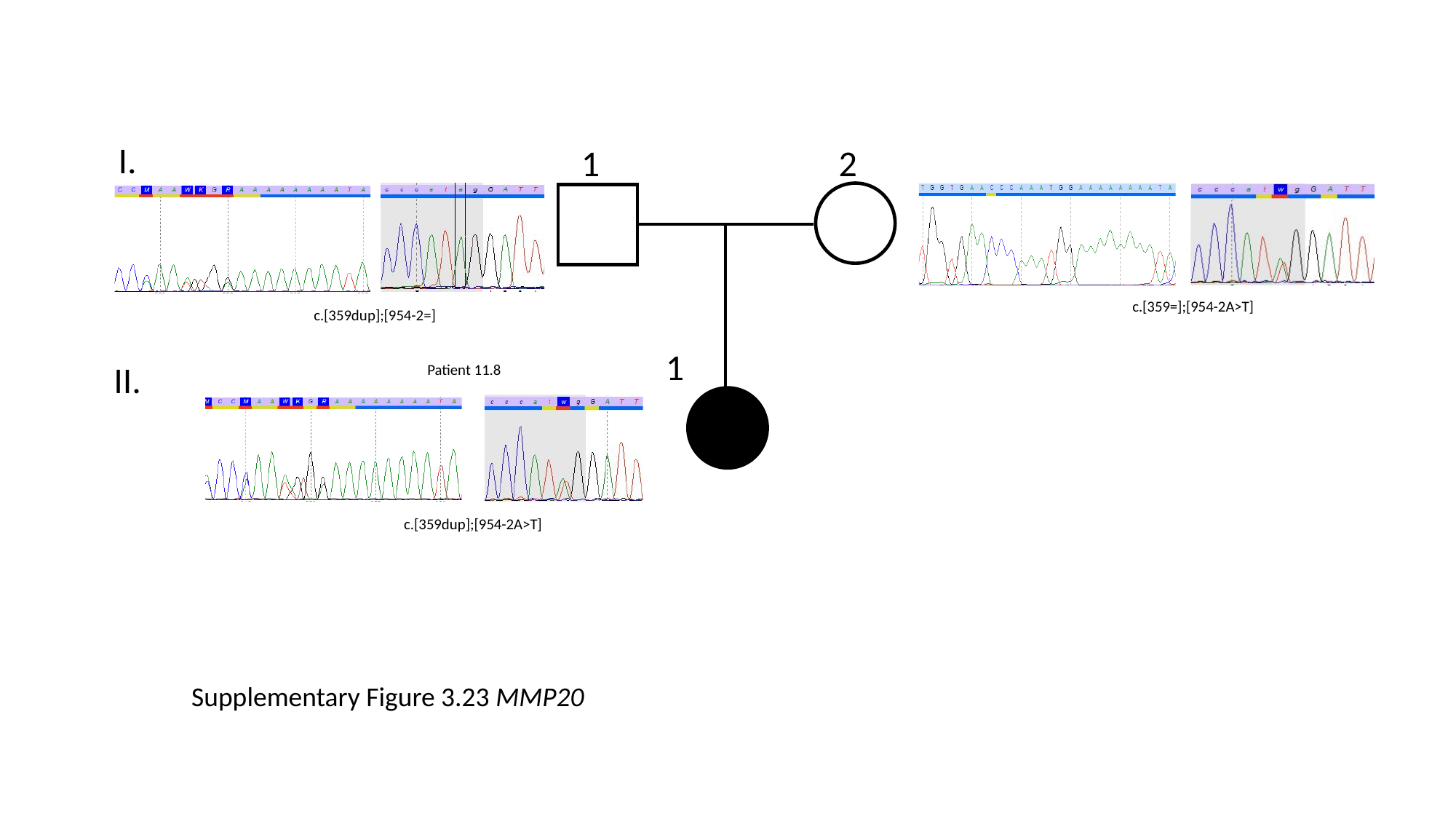

I.
1
2
c.[359=];[954-2A>T]
c.[359dup];[954-2=]
1
II.
Patient 11.8
c.[359dup];[954-2A>T]
Supplementary Figure 3.23 MMP20

## Slide 24
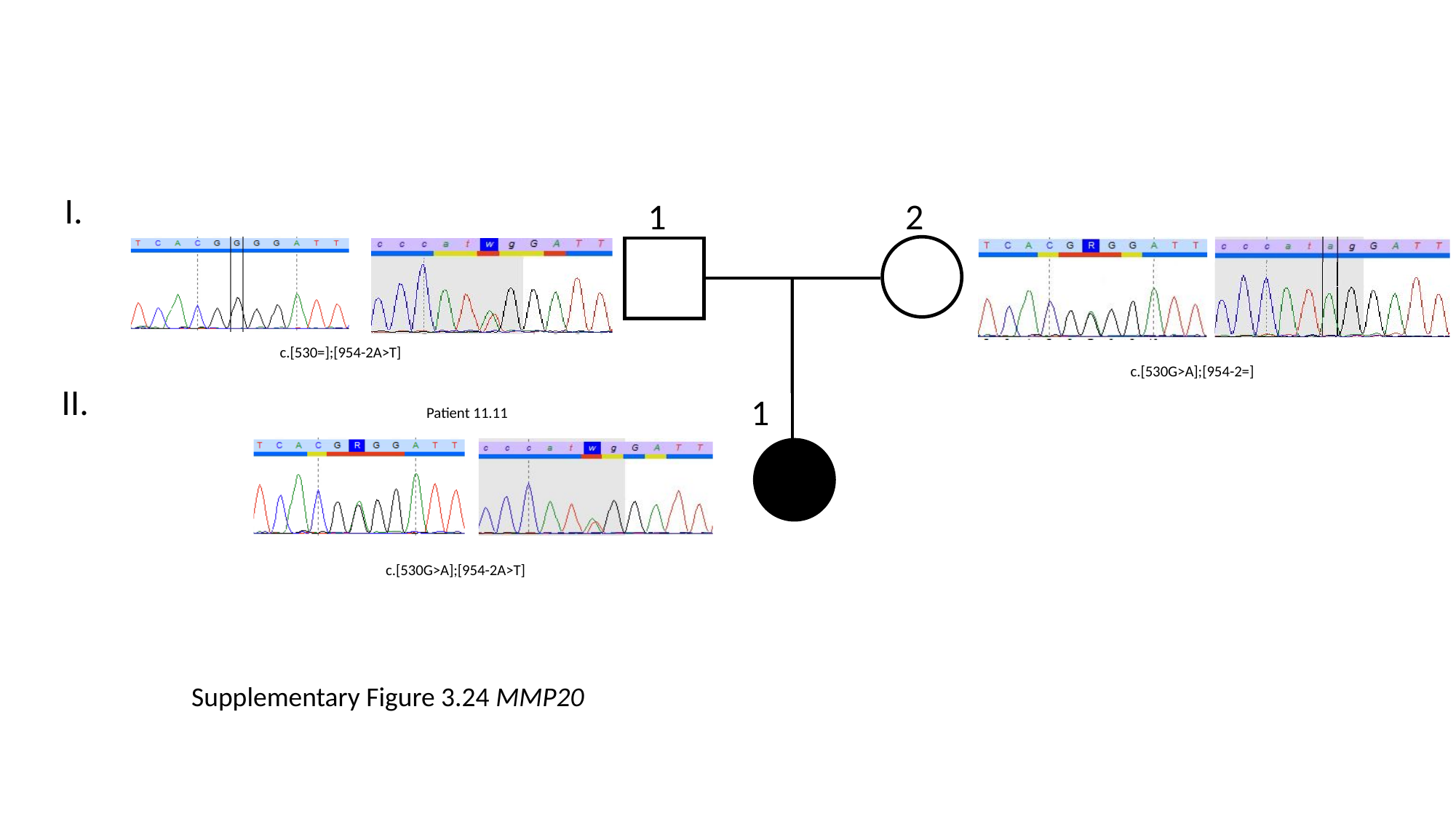

I.
1
2
c.[530=];[954-2A>T]
c.[530G>A];[954-2=]
II.
1
Patient 11.11
c.[530G>A];[954-2A>T]
Supplementary Figure 3.24 MMP20

## Slide 25
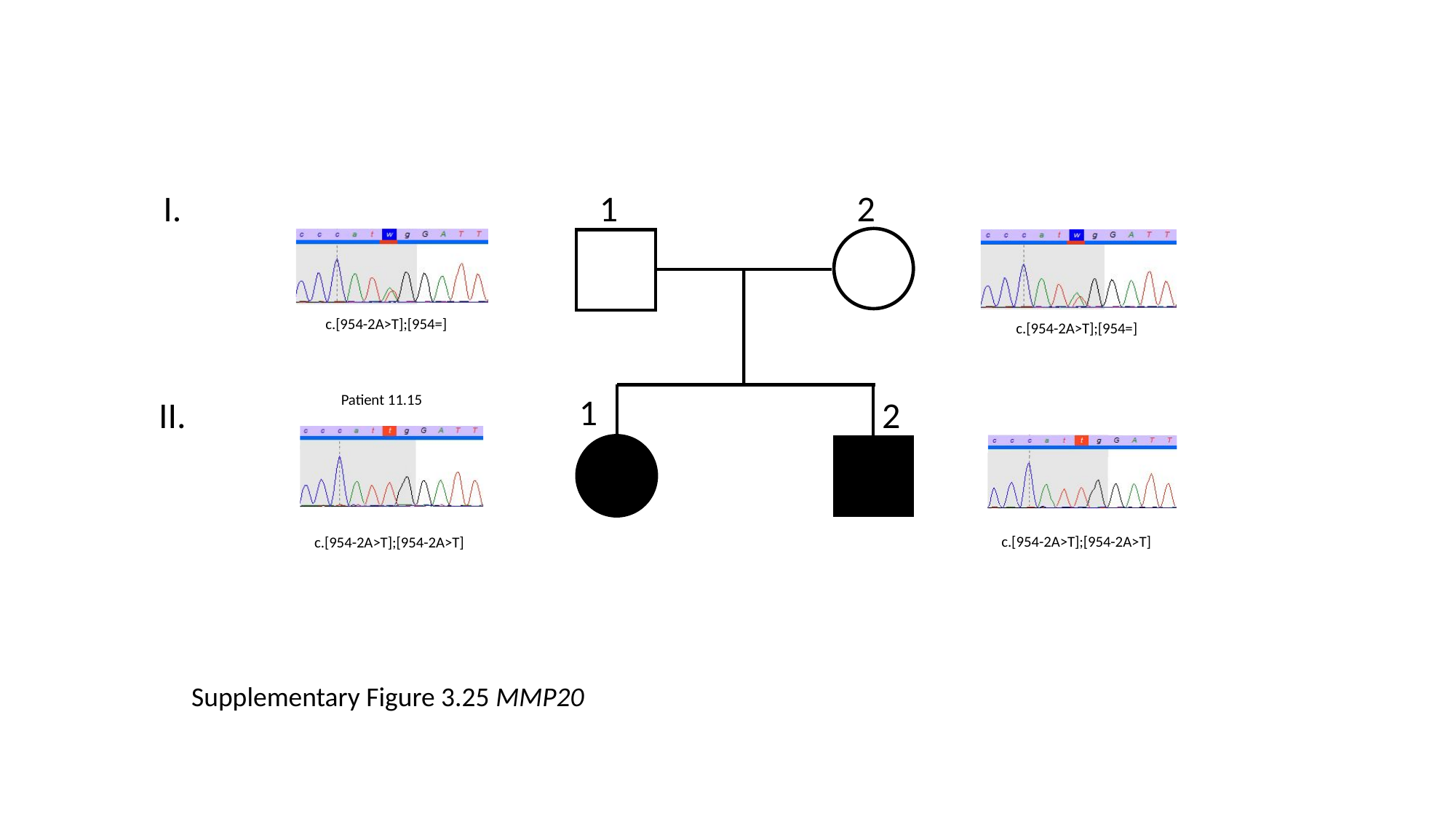

I.
1
2
c.[954-2A>T];[954=]
c.[954-2A>T];[954=]
1
Patient 11.15
II.
2
c.[954-2A>T];[954-2A>T]
c.[954-2A>T];[954-2A>T]
Supplementary Figure 3.25 MMP20

## Slide 26
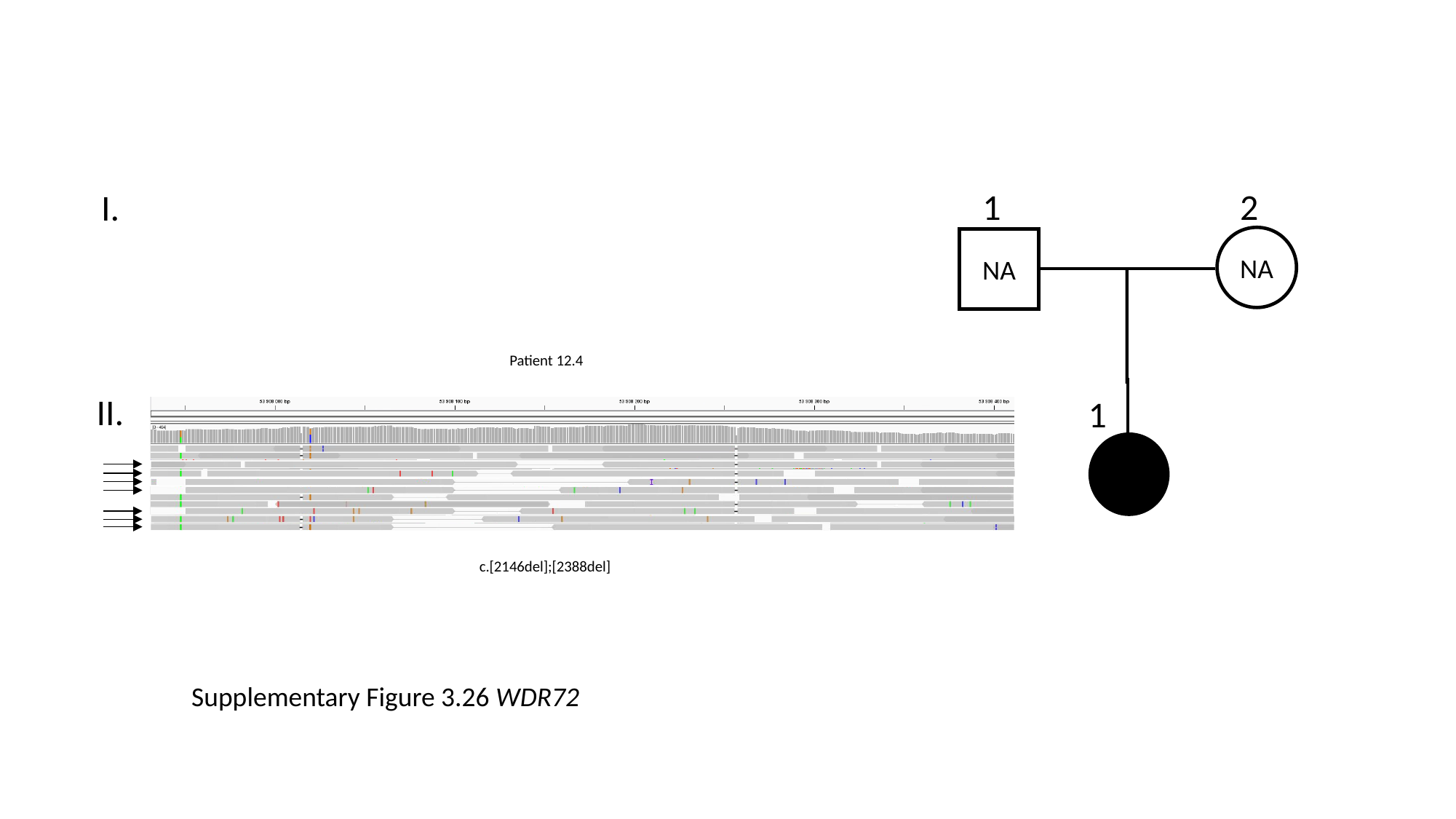

1
2
I.
NA
NA
Patient 12.4
II.
1
c.[2146del];[2388del]
Supplementary Figure 3.26 WDR72

## Slide 27
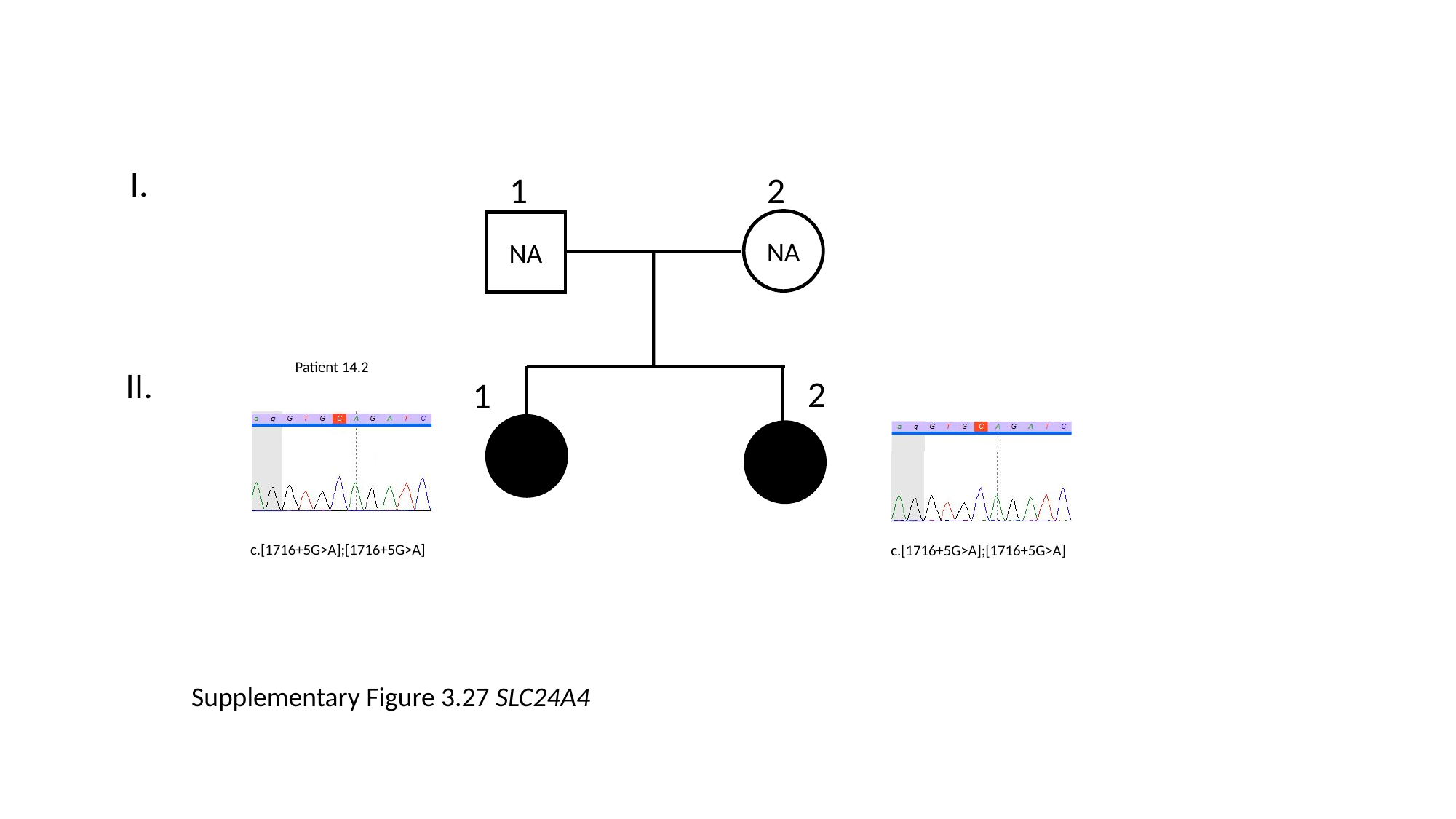

I.
1
2
NA
NA
Patient 14.2
II.
2
1
c.[1716+5G>A];[1716+5G>A]
c.[1716+5G>A];[1716+5G>A]
Supplementary Figure 3.27 SLC24A4

## Slide 28
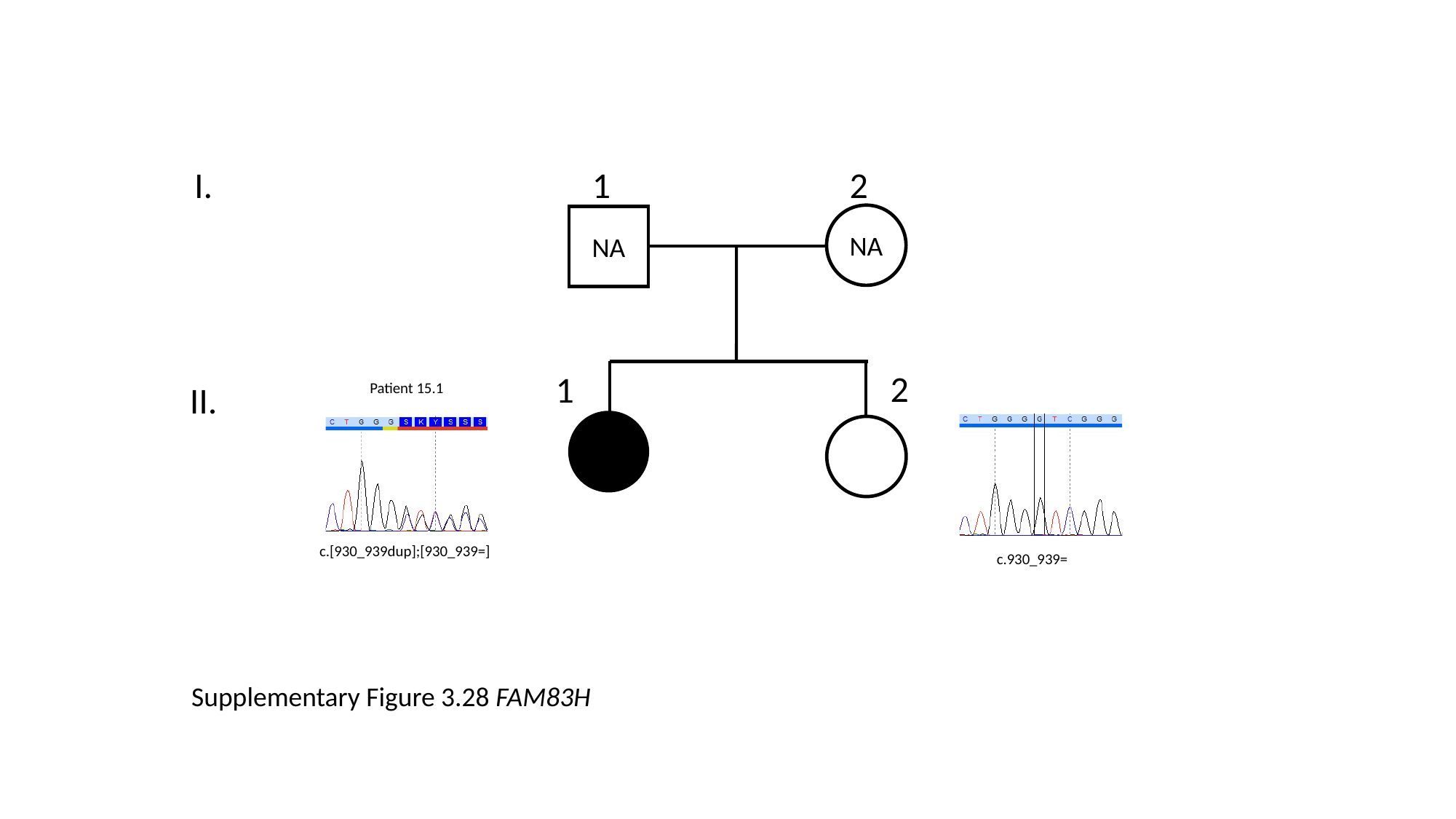

I.
1
2
NA
NA
2
1
II.
Patient 15.1
c.[930_939dup];[930_939=]
c.930_939=
Supplementary Figure 3.28 FAM83H

## Slide 29
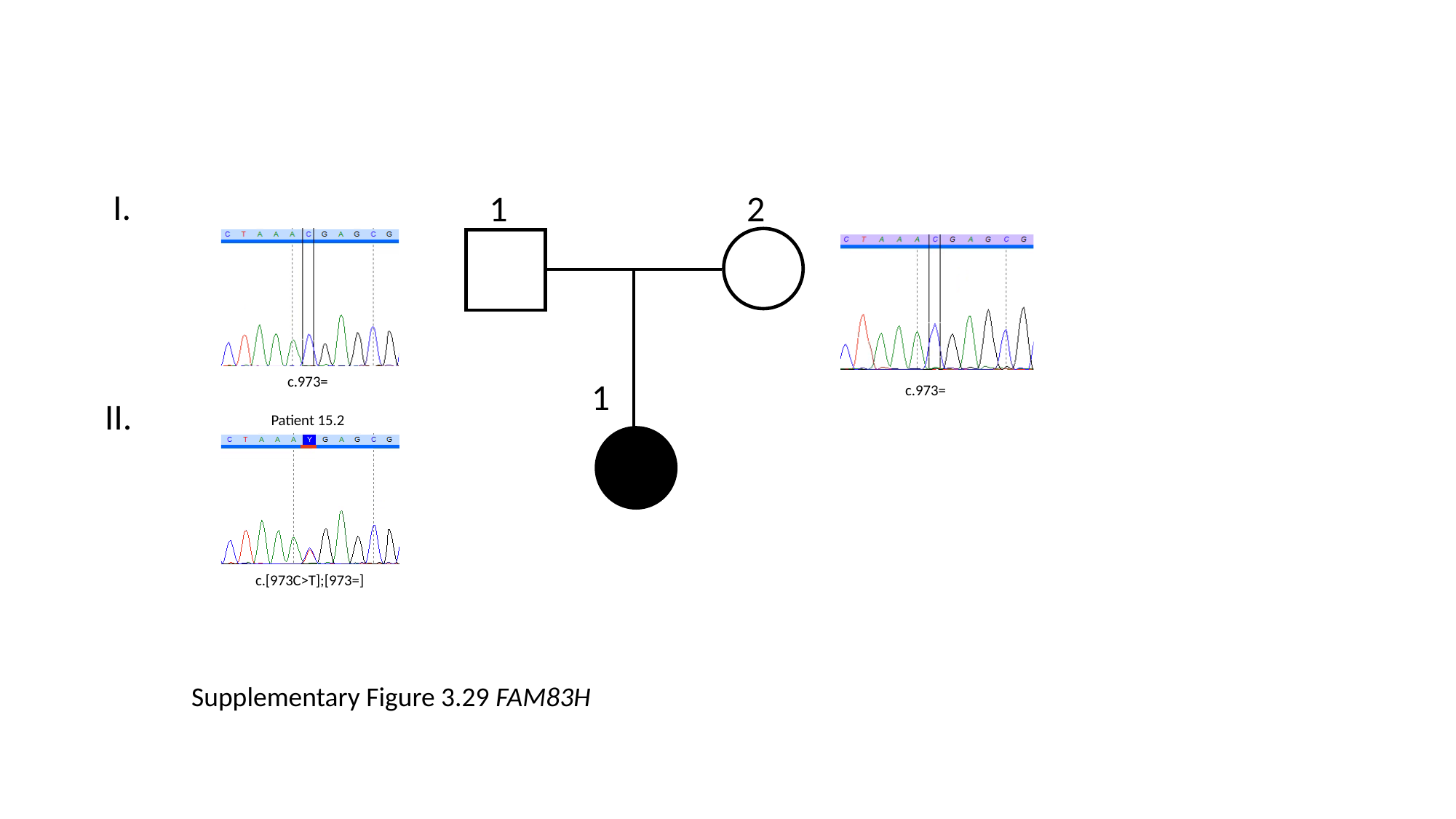

I.
1
2
c.973=
1
c.973=
II.
Patient 15.2
c.[973C>T];[973=]
Supplementary Figure 3.29 FAM83H

## Slide 30
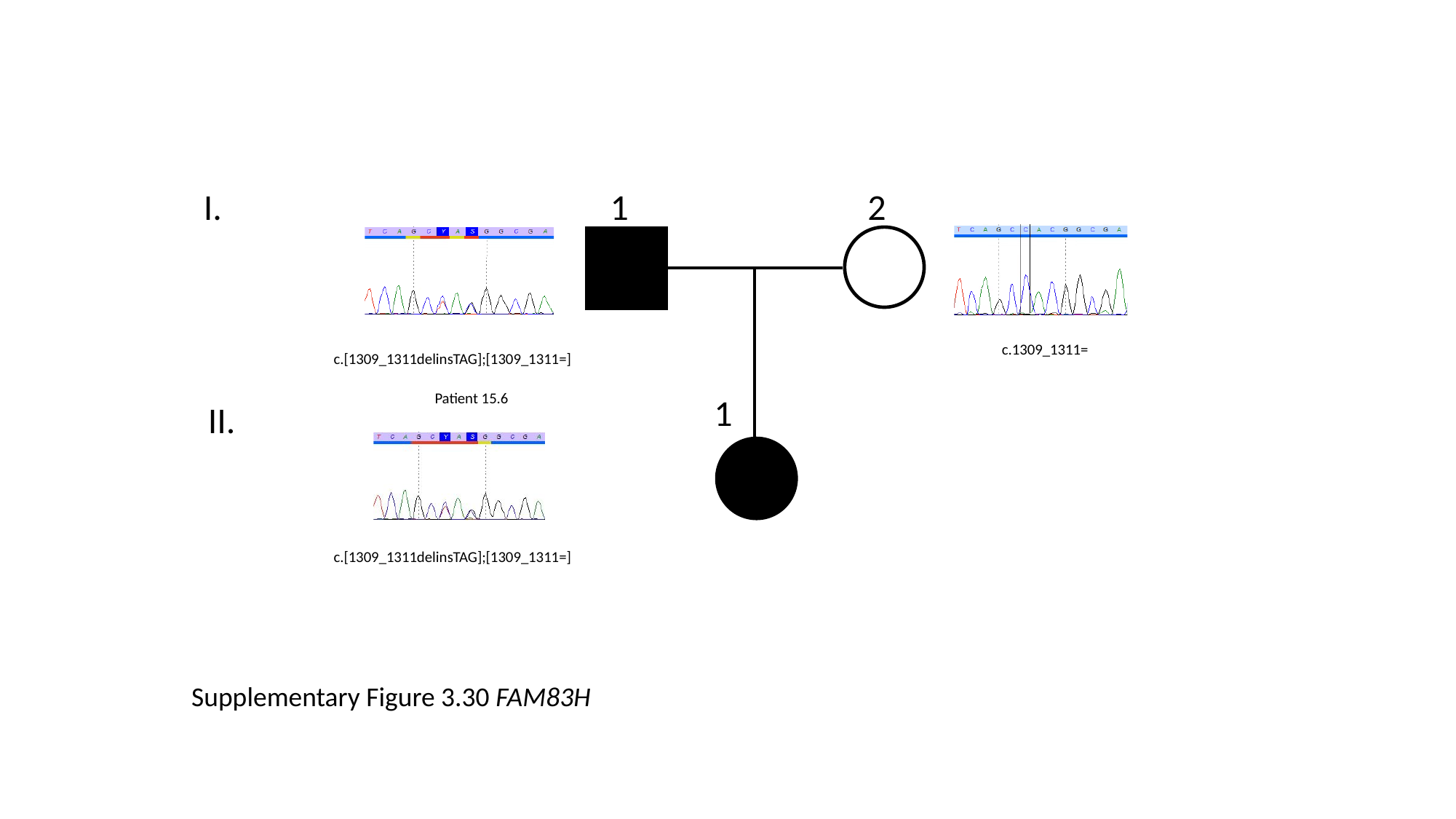

I.
1
2
c.1309_1311=
c.[1309_1311delinsTAG];[1309_1311=]
Patient 15.6
1
II.
c.[1309_1311delinsTAG];[1309_1311=]
Supplementary Figure 3.30 FAM83H

## Slide 31
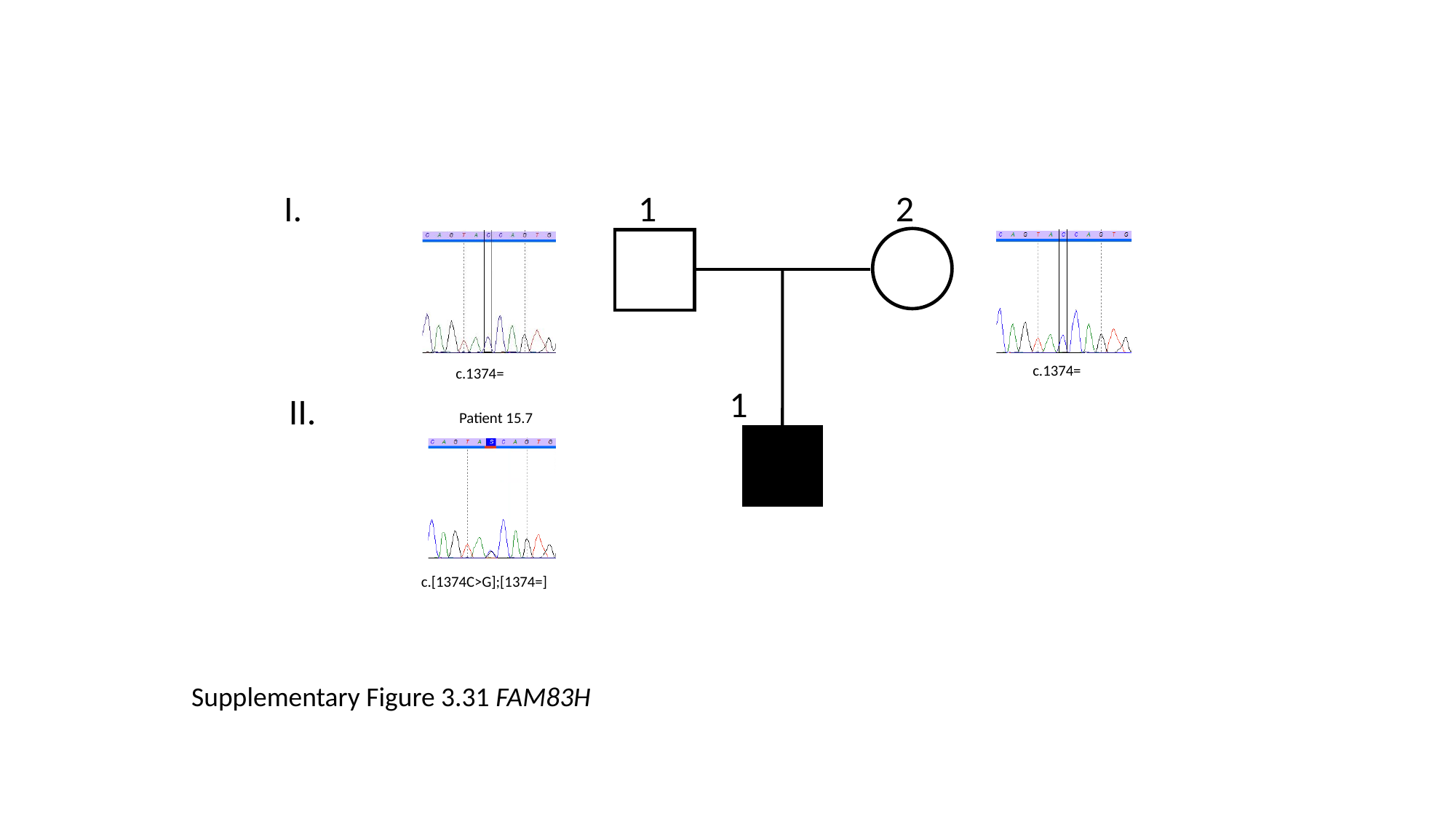

I.
1
2
c.1374=
c.1374=
1
II.
Patient 15.7
c.[1374C>G];[1374=]
Supplementary Figure 3.31 FAM83H

## Slide 32
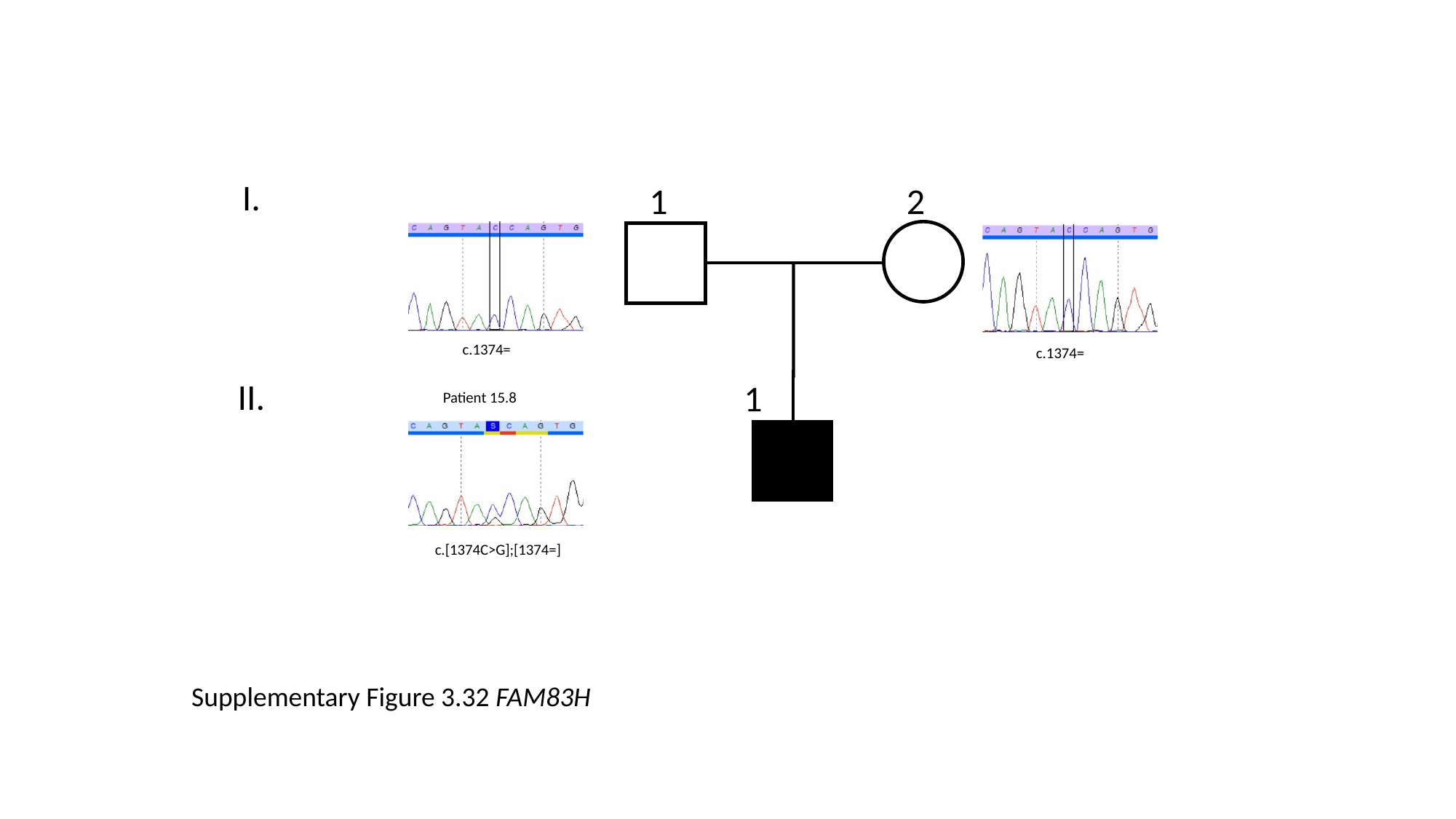

I.
1
2
c.1374=
c.1374=
II.
1
Patient 15.8
c.[1374C>G];[1374=]
Supplementary Figure 3.32 FAM83H

## Slide 33
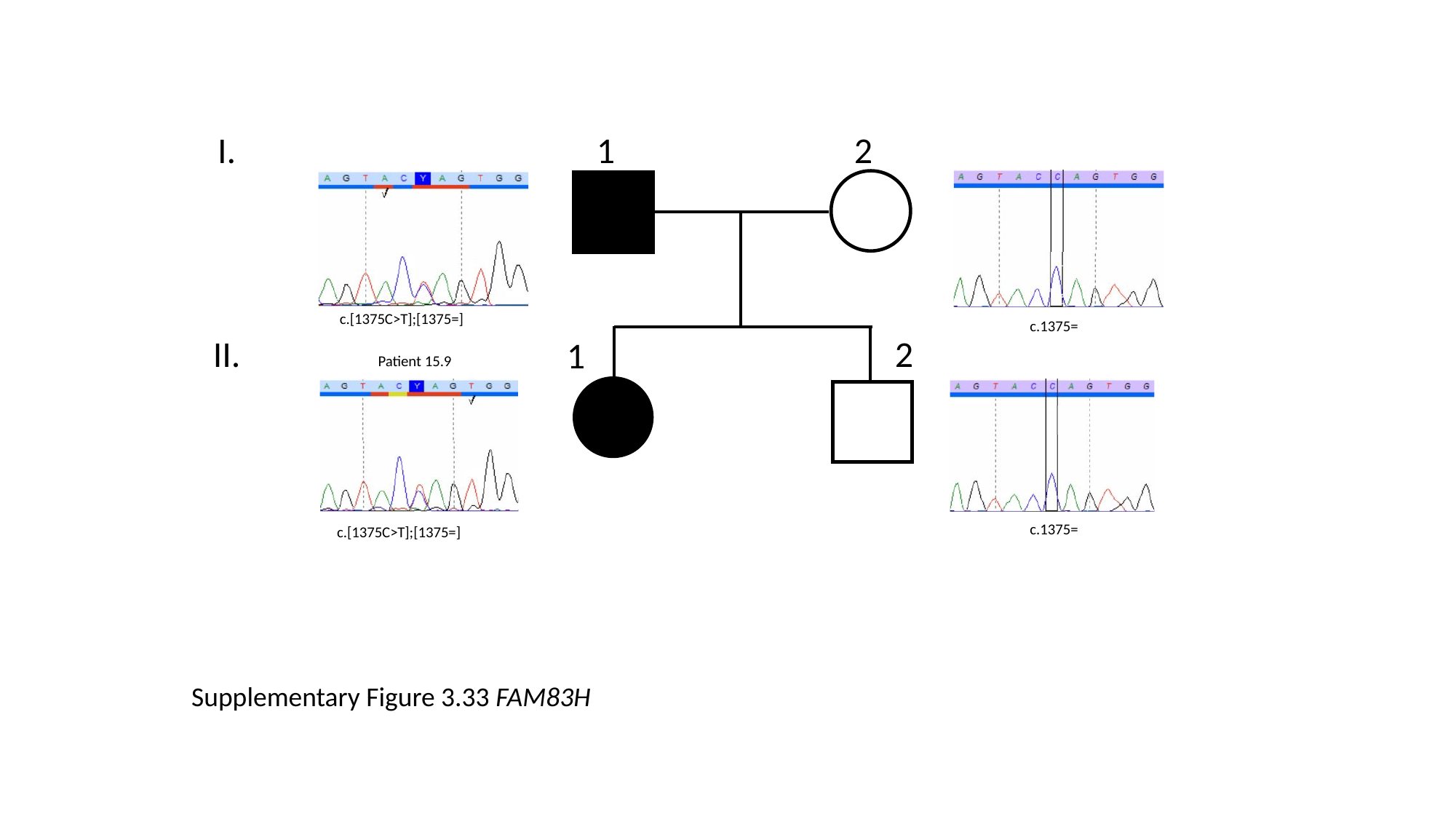

I.
1
2
c.[1375C>T];[1375=]
c.1375=
II.
2
1
Patient 15.9
c.1375=
c.[1375C>T];[1375=]
Supplementary Figure 3.33 FAM83H

## Slide 34
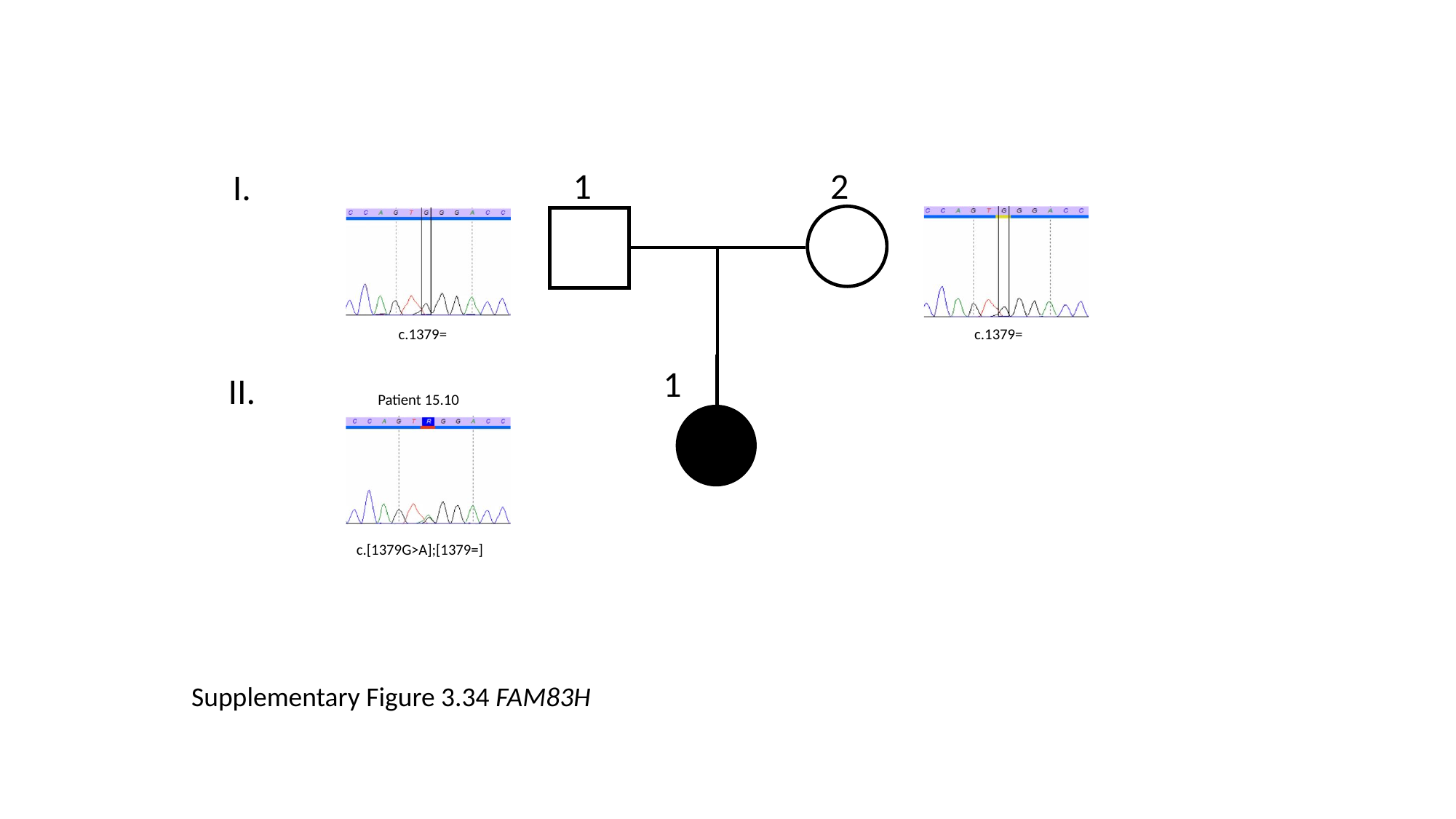

1
2
I.
c.1379=
c.1379=
1
II.
Patient 15.10
c.[1379G>A];[1379=]
Supplementary Figure 3.34 FAM83H

## Slide 35
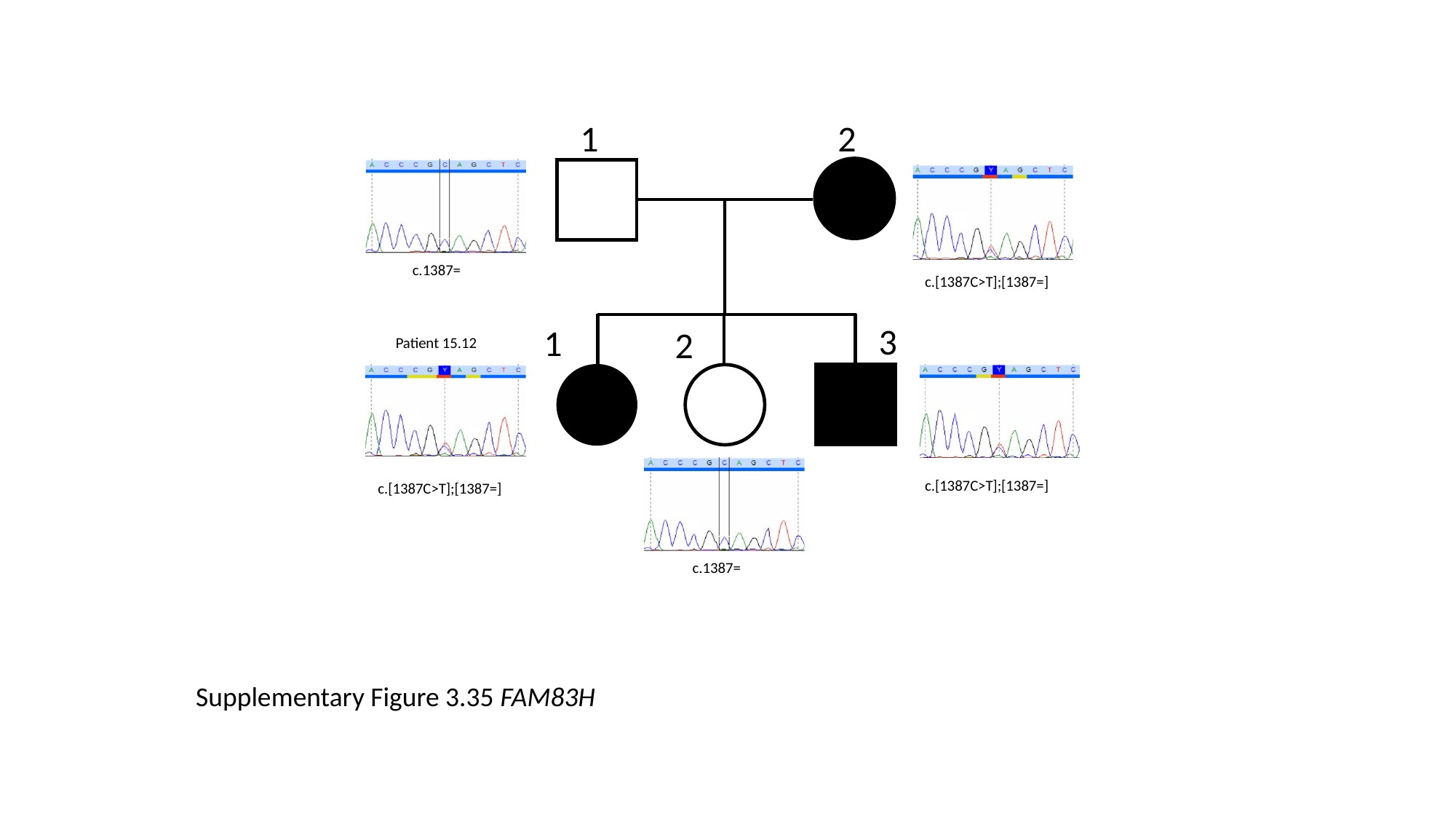

1
2
c.1387=
c.[1387C>T];[1387=]
3
1
2
Patient 15.12
c.[1387C>T];[1387=]
c.[1387C>T];[1387=]
c.1387=
Supplementary Figure 3.35 FAM83H

## Slide 36
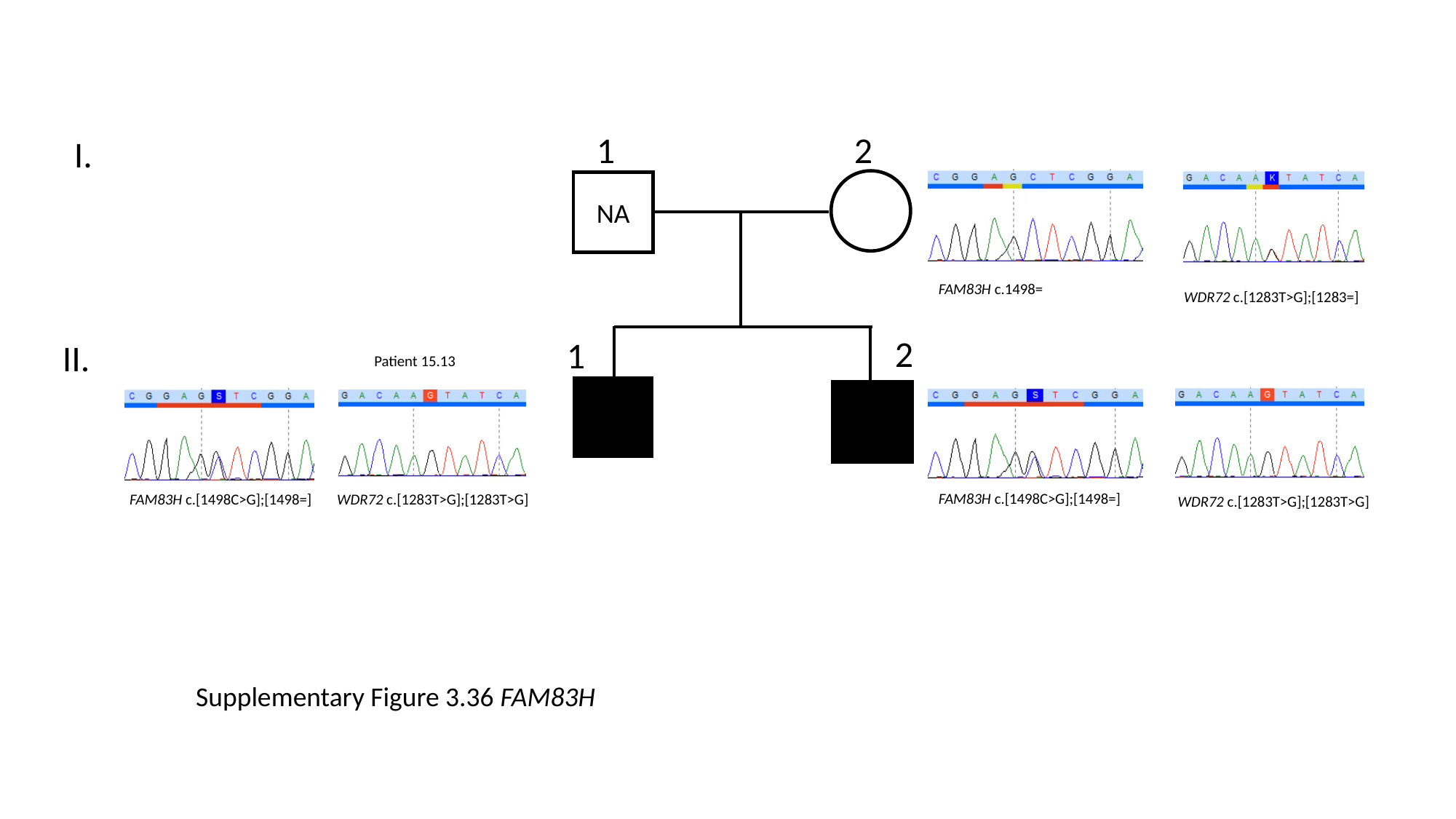

1
2
I.
NA
FAM83H c.1498=
WDR72 c.[1283T>G];[1283=]
2
1
II.
Patient 15.13
FAM83H c.[1498C>G];[1498=]
FAM83H c.[1498C>G];[1498=]
WDR72 c.[1283T>G];[1283T>G]
WDR72 c.[1283T>G];[1283T>G]
Supplementary Figure 3.36 FAM83H

## Slide 37
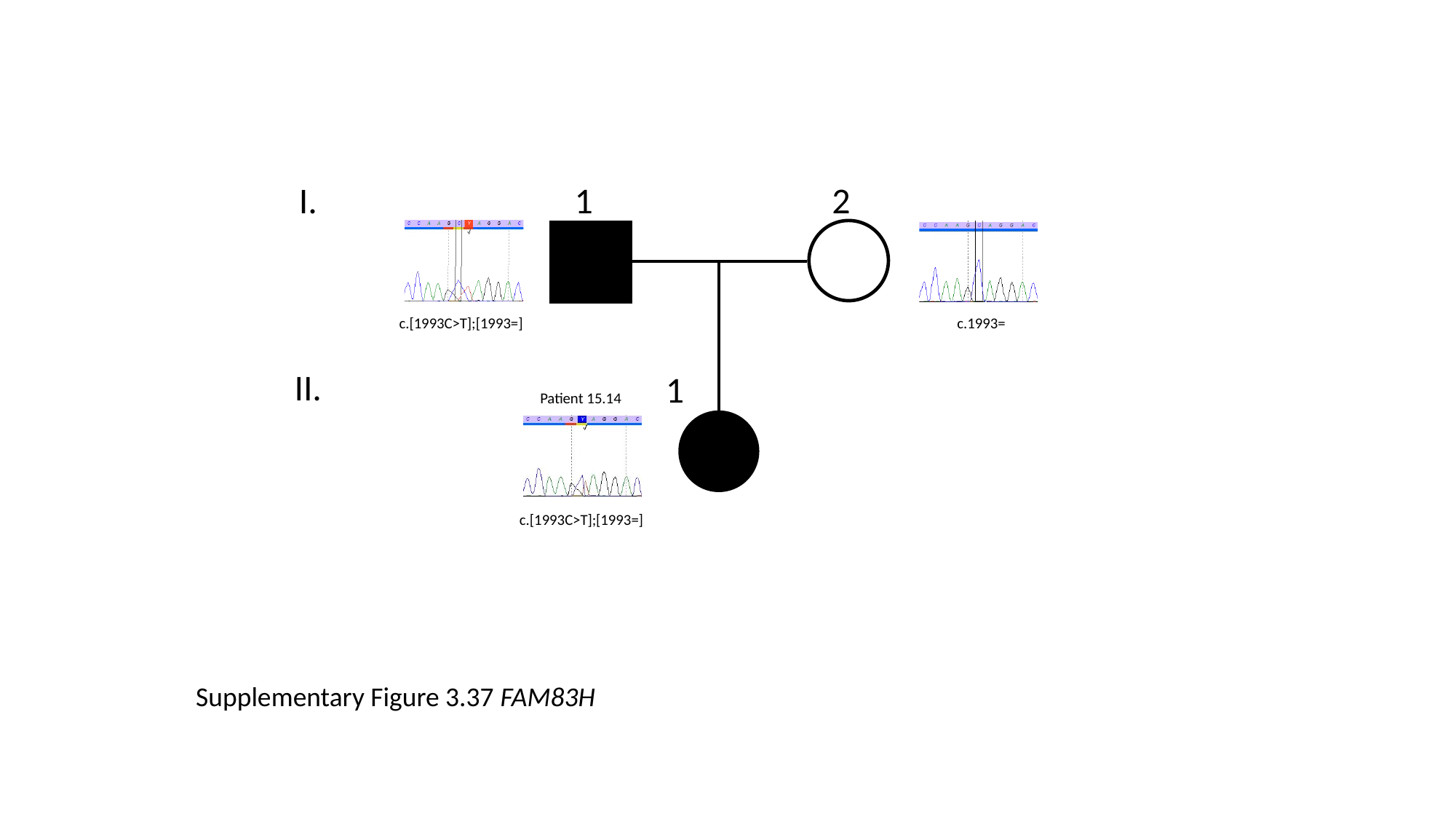

1
2
I.
c.1993=
c.[1993C>T];[1993=]
II.
1
Patient 15.14
c.[1993C>T];[1993=]
Supplementary Figure 3.37 FAM83H

## Slide 38
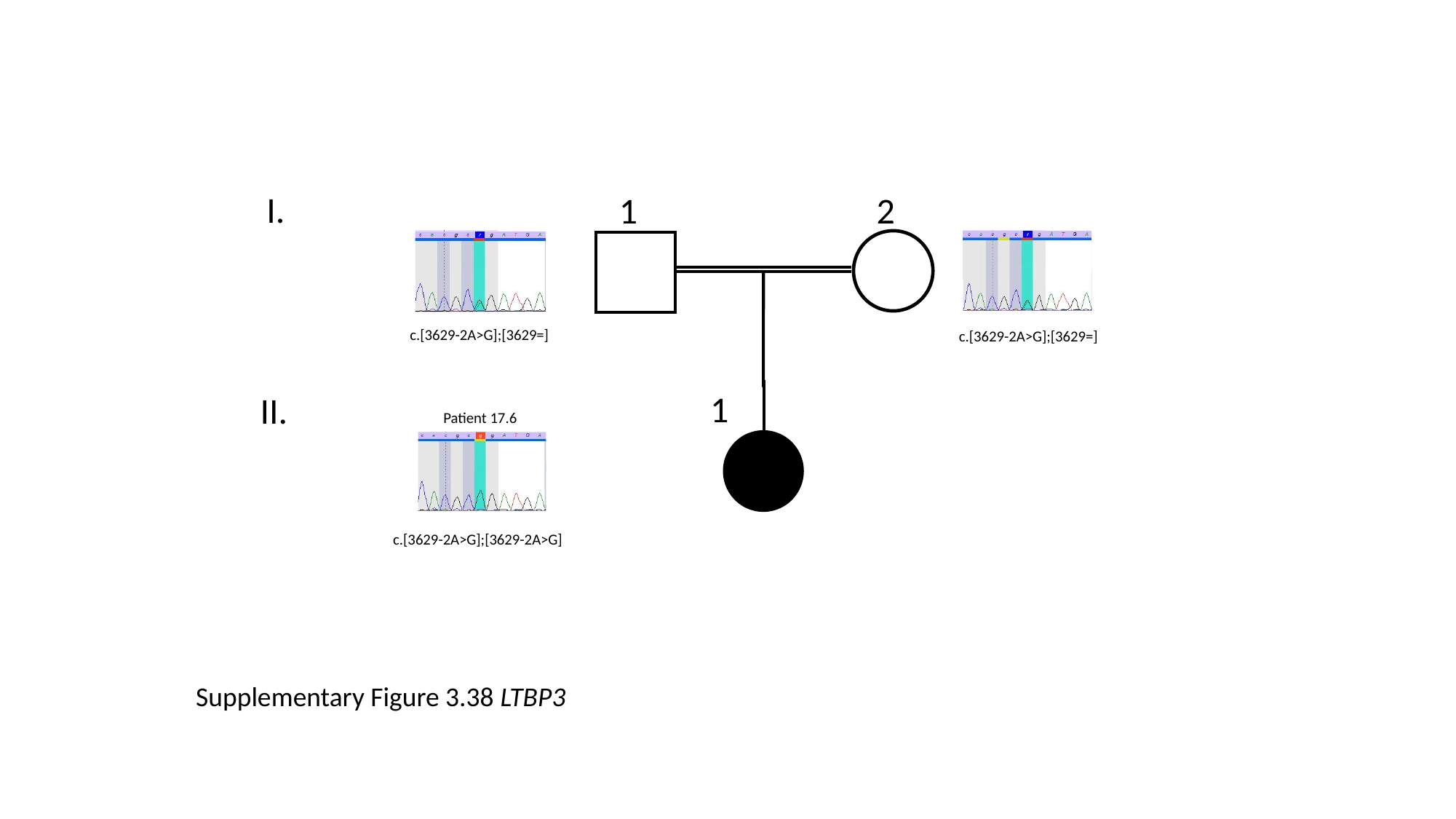

I.
1
2
c.[3629-2A>G];[3629=]
c.[3629-2A>G];[3629=]
1
II.
Patient 17.6
c.[3629-2A>G];[3629-2A>G]
Supplementary Figure 3.38 LTBP3

## Slide 39
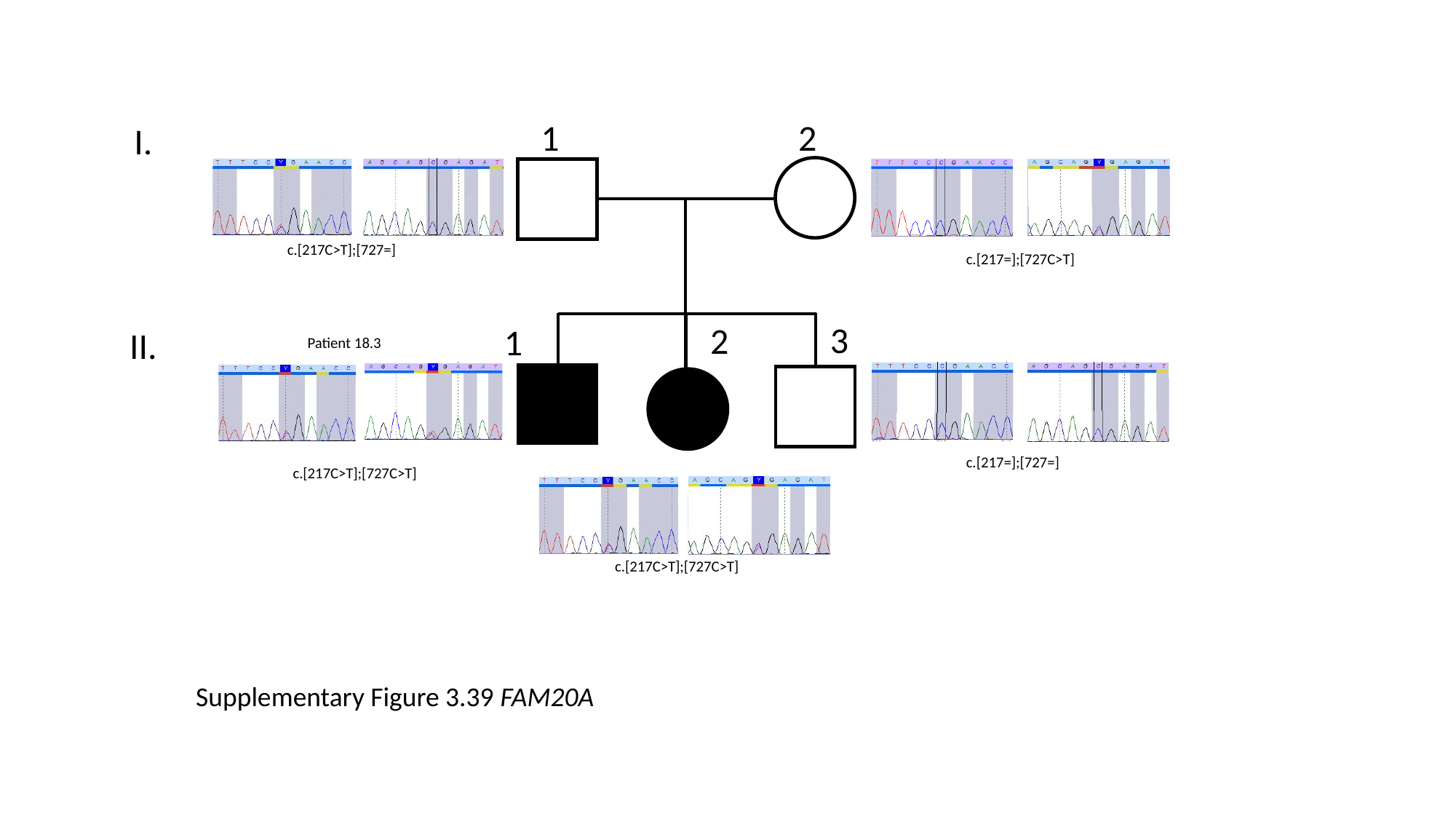

1
2
I.
c.[217C>T];[727=]
c.[217=];[727C>T]
3
2
1
II.
Patient 18.3
c.[217=];[727=]
c.[217C>T];[727C>T]
c.[217C>T];[727C>T]
Supplementary Figure 3.39 FAM20A

## Slide 40
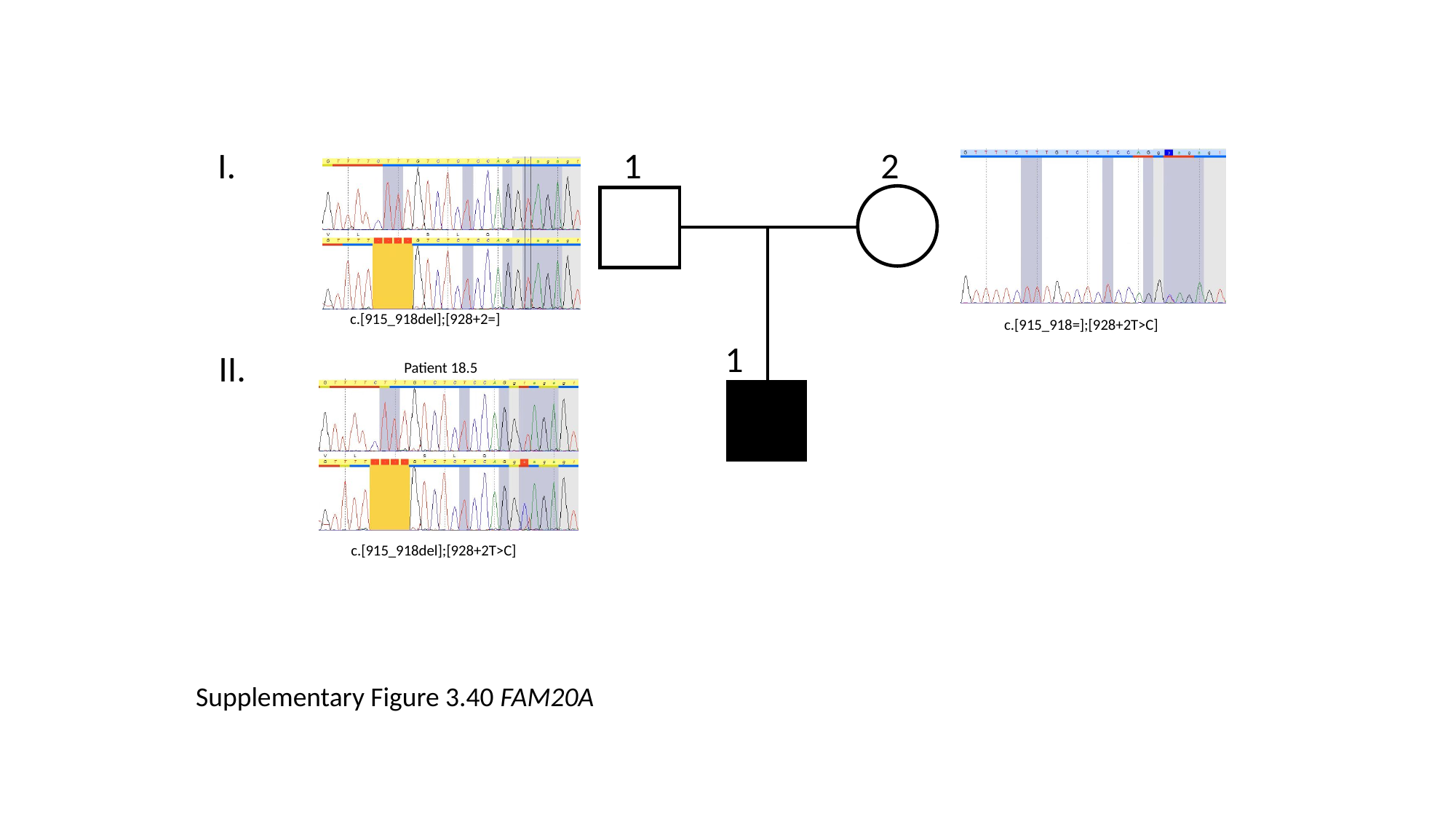

I.
1
2
c.[915_918del];[928+2=]
c.[915_918=];[928+2T>C]
1
II.
Patient 18.5
c.[915_918del];[928+2T>C]
Supplementary Figure 3.40 FAM20A

## Slide 41
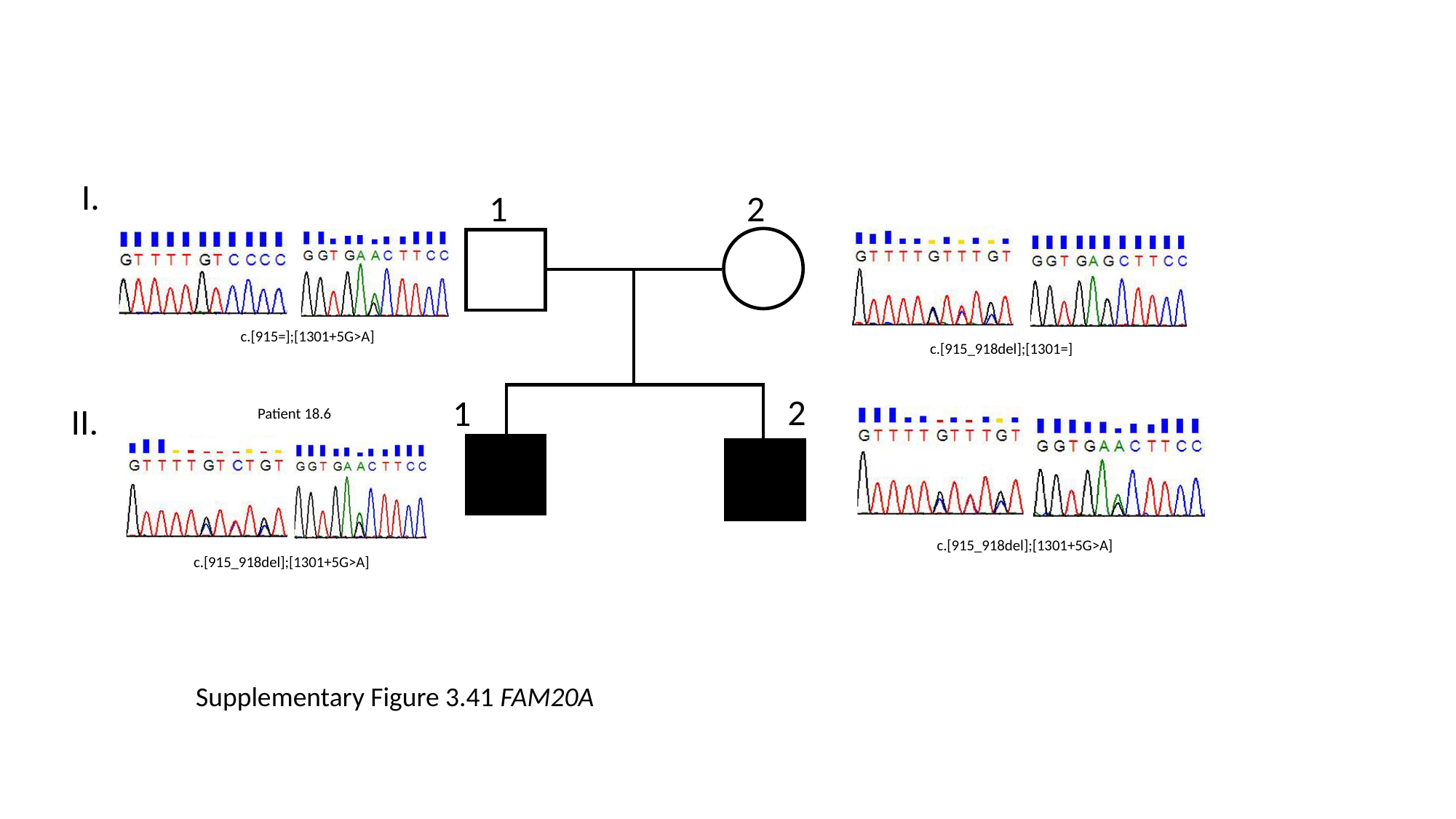

I.
1
2
c.[915=];[1301+5G>A]
c.[915_918del];[1301=]
2
1
II.
Patient 18.6
c.[915_918del];[1301+5G>A]
c.[915_918del];[1301+5G>A]
Supplementary Figure 3.41 FAM20A

## Slide 42
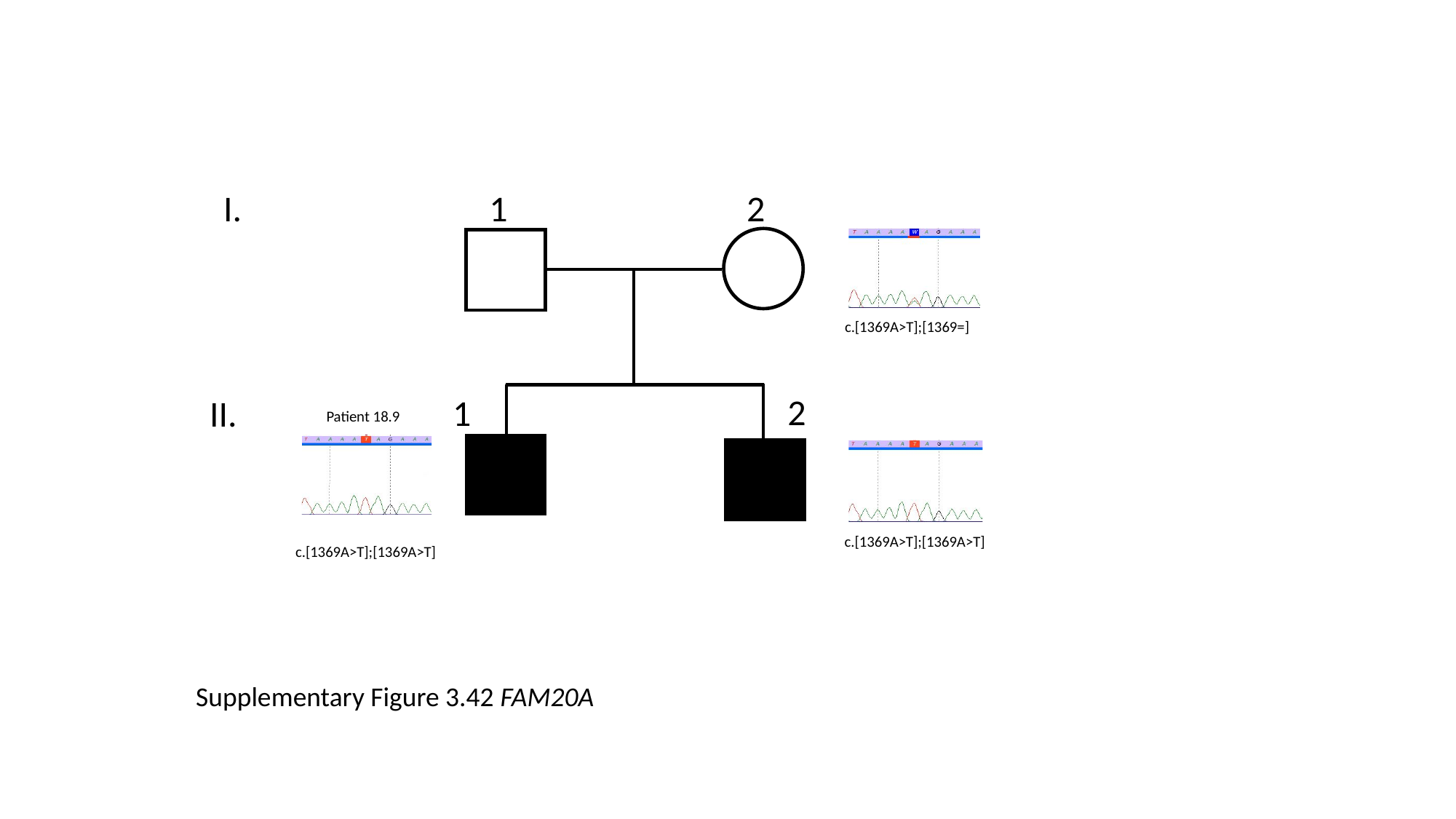

I.
1
2
c.[1369A>T];[1369=]
2
1
II.
Patient 18.9
c.[1369A>T];[1369A>T]
c.[1369A>T];[1369A>T]
Supplementary Figure 3.42 FAM20A

## Slide 43
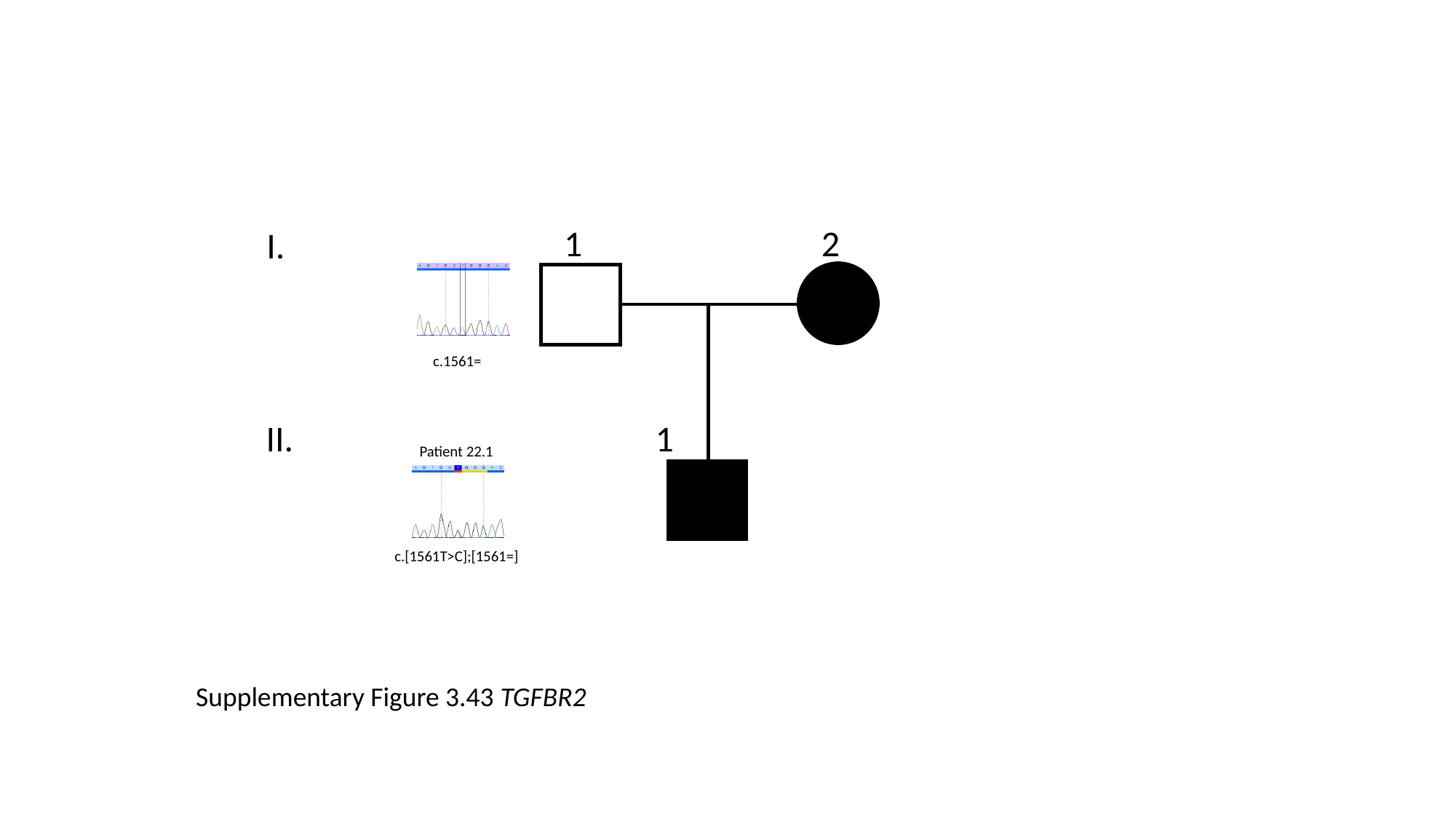

1
2
I.
c.1561=
II.
1
Patient 22.1
c.[1561T>C];[1561=]
Supplementary Figure 3.43 TGFBR2

## Slide 44
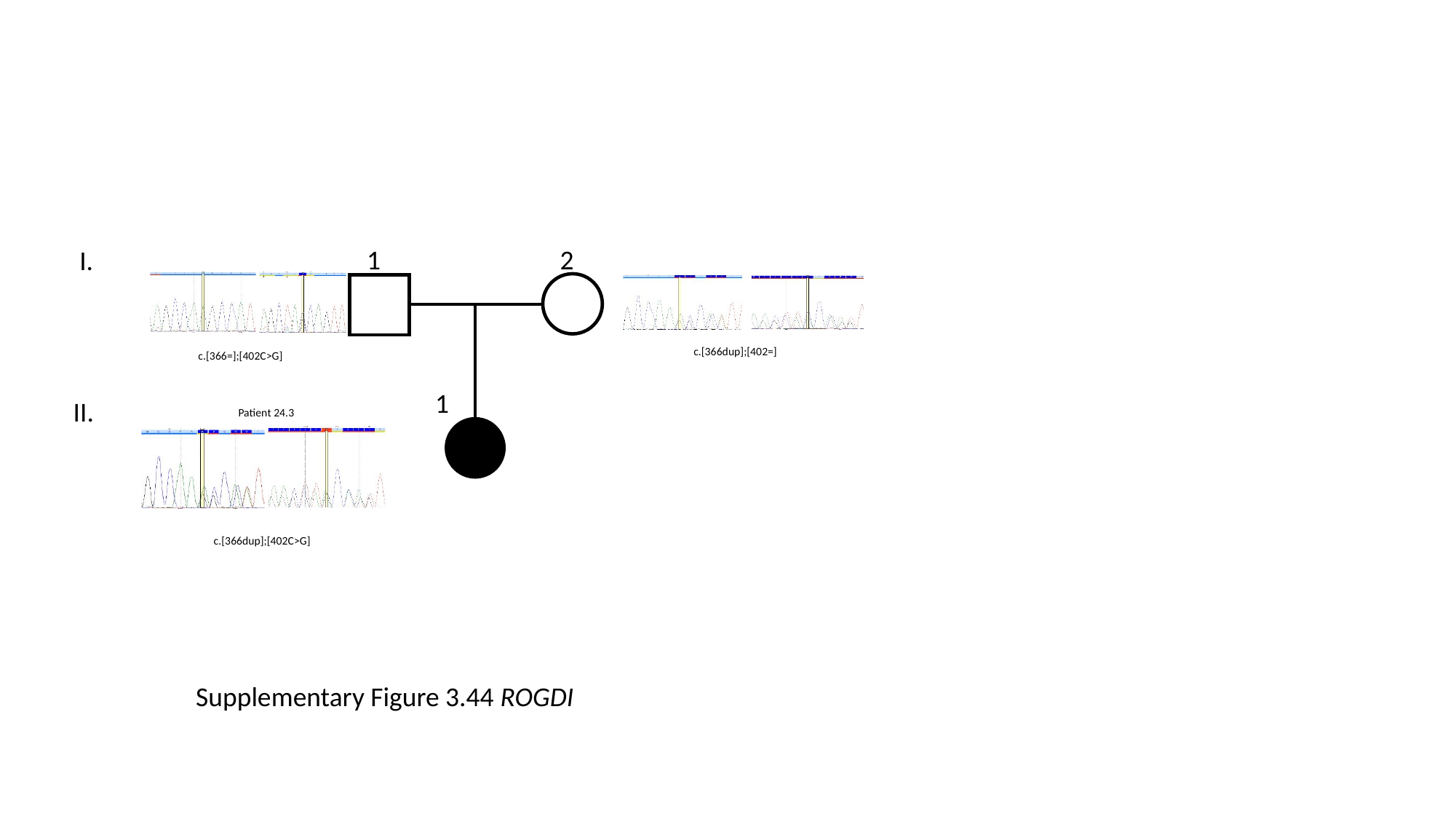

1
2
I.
c.[366dup];[402=]
c.[366=];[402C>G]
1
II.
Patient 24.3
c.[366dup];[402C>G]
Supplementary Figure 3.44 ROGDI

## Slide 45
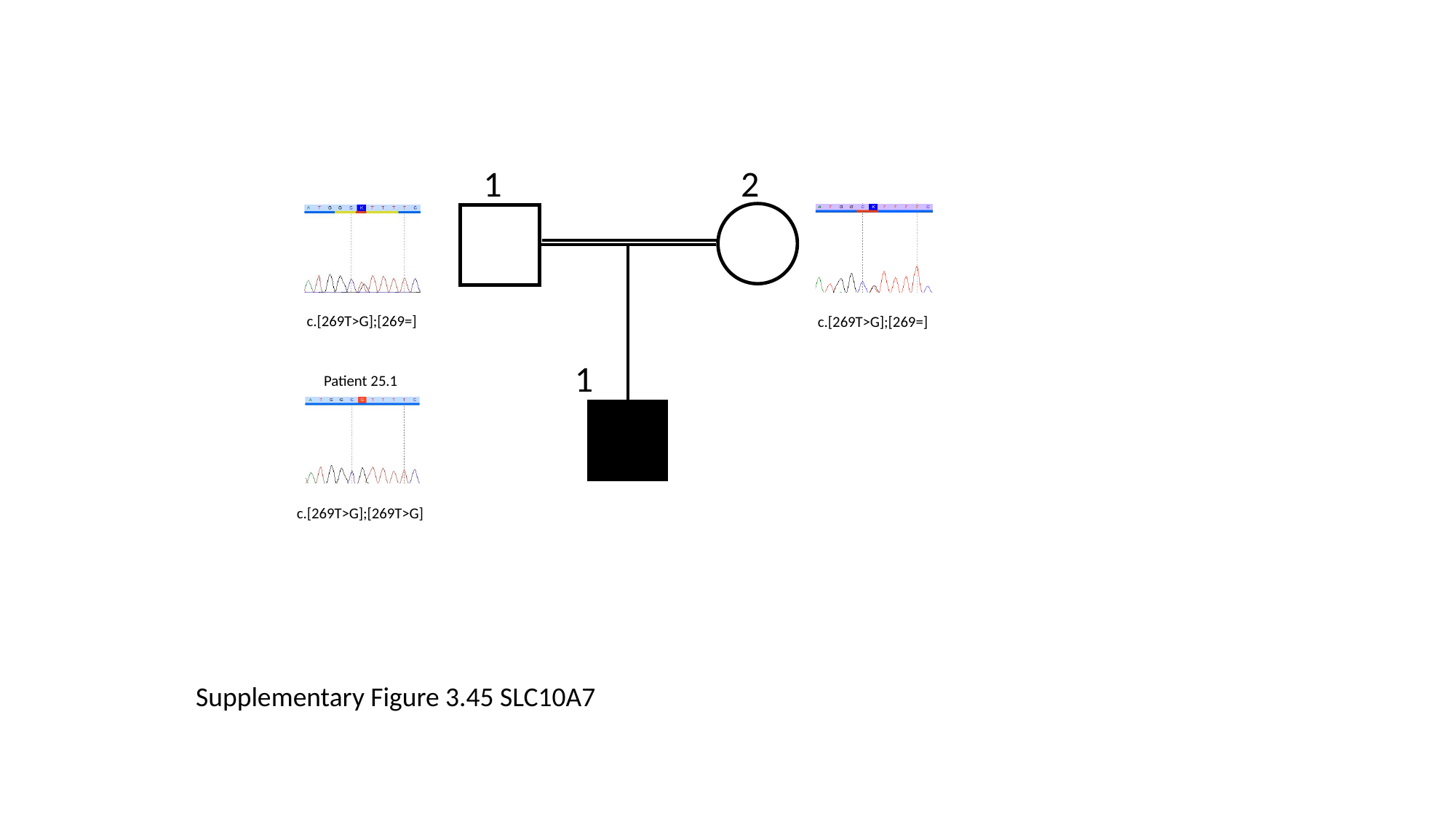

1
2
c.[269T>G];[269=]
c.[269T>G];[269=]
1
Patient 25.1
c.[269T>G];[269T>G]
Supplementary Figure 3.45 SLC10A7

## Slide 46
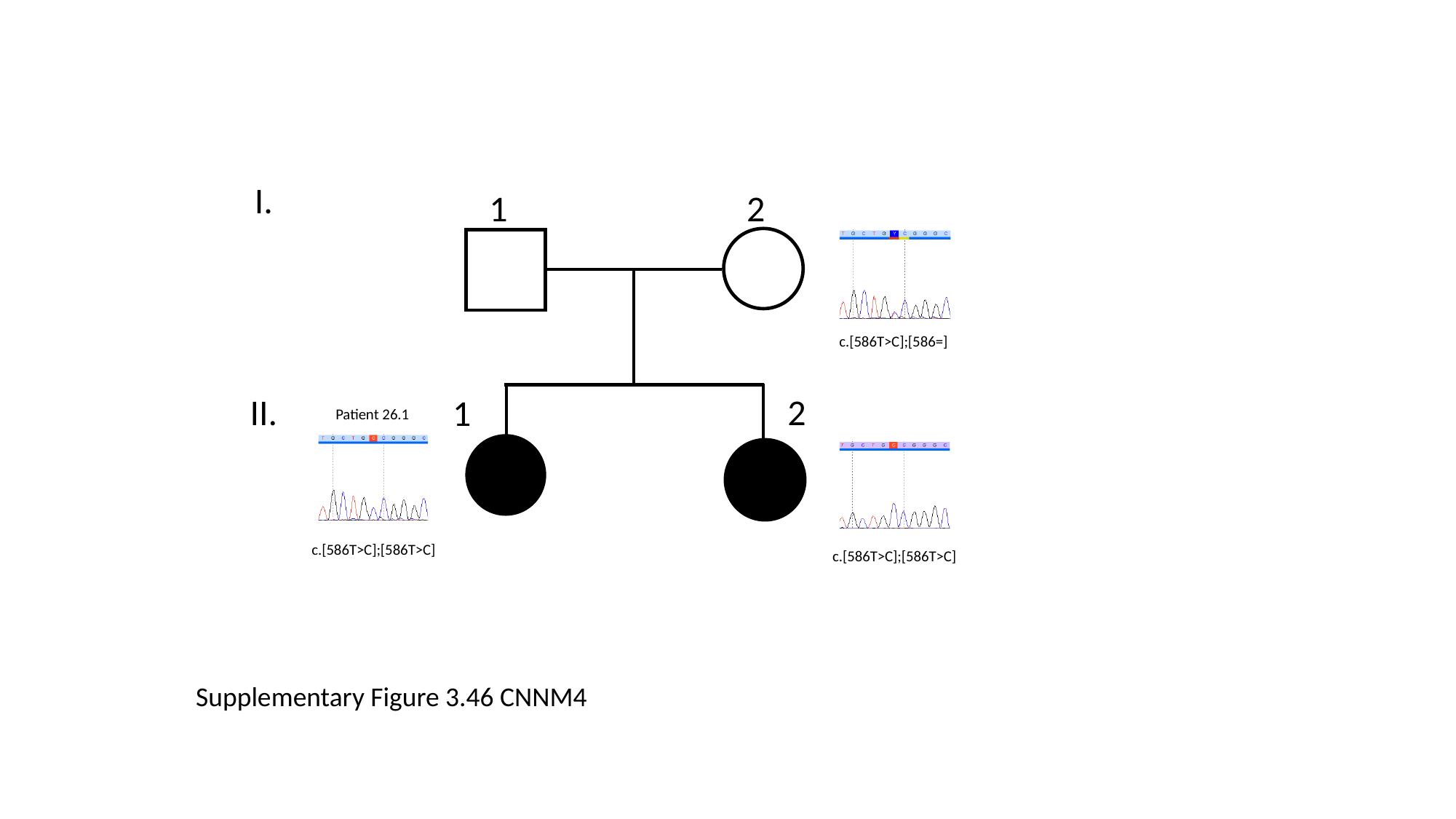

I.
1
2
c.[586T>C];[586=]
II.
2
1
Patient 26.1
c.[586T>C];[586T>C]
c.[586T>C];[586T>C]
Supplementary Figure 3.46 CNNM4

## Slide 47
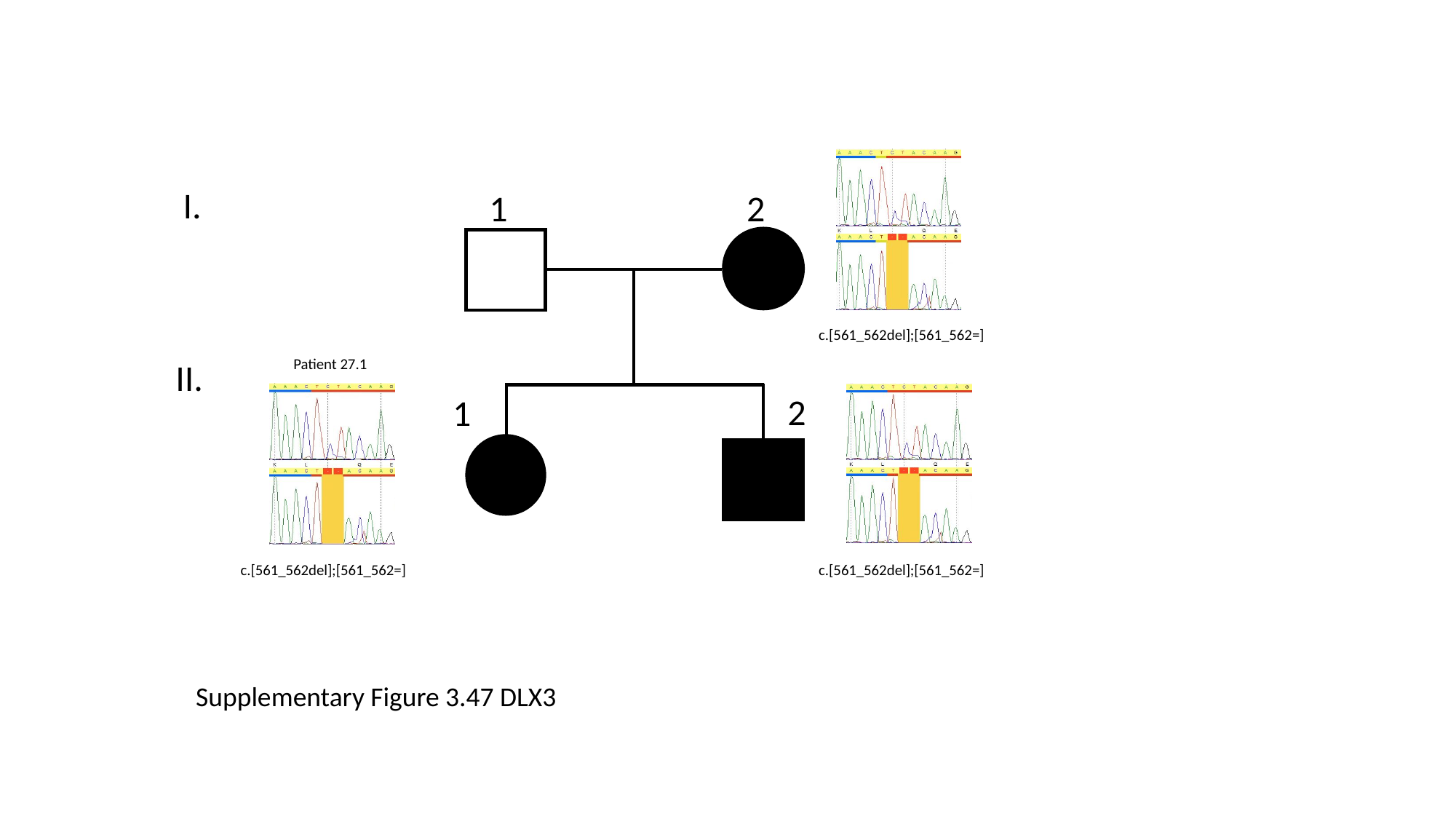

I.
1
2
c.[561_562del];[561_562=]
II.
Patient 27.1
2
1
c.[561_562del];[561_562=]
c.[561_562del];[561_562=]
Supplementary Figure 3.47 DLX3

## Slide 48
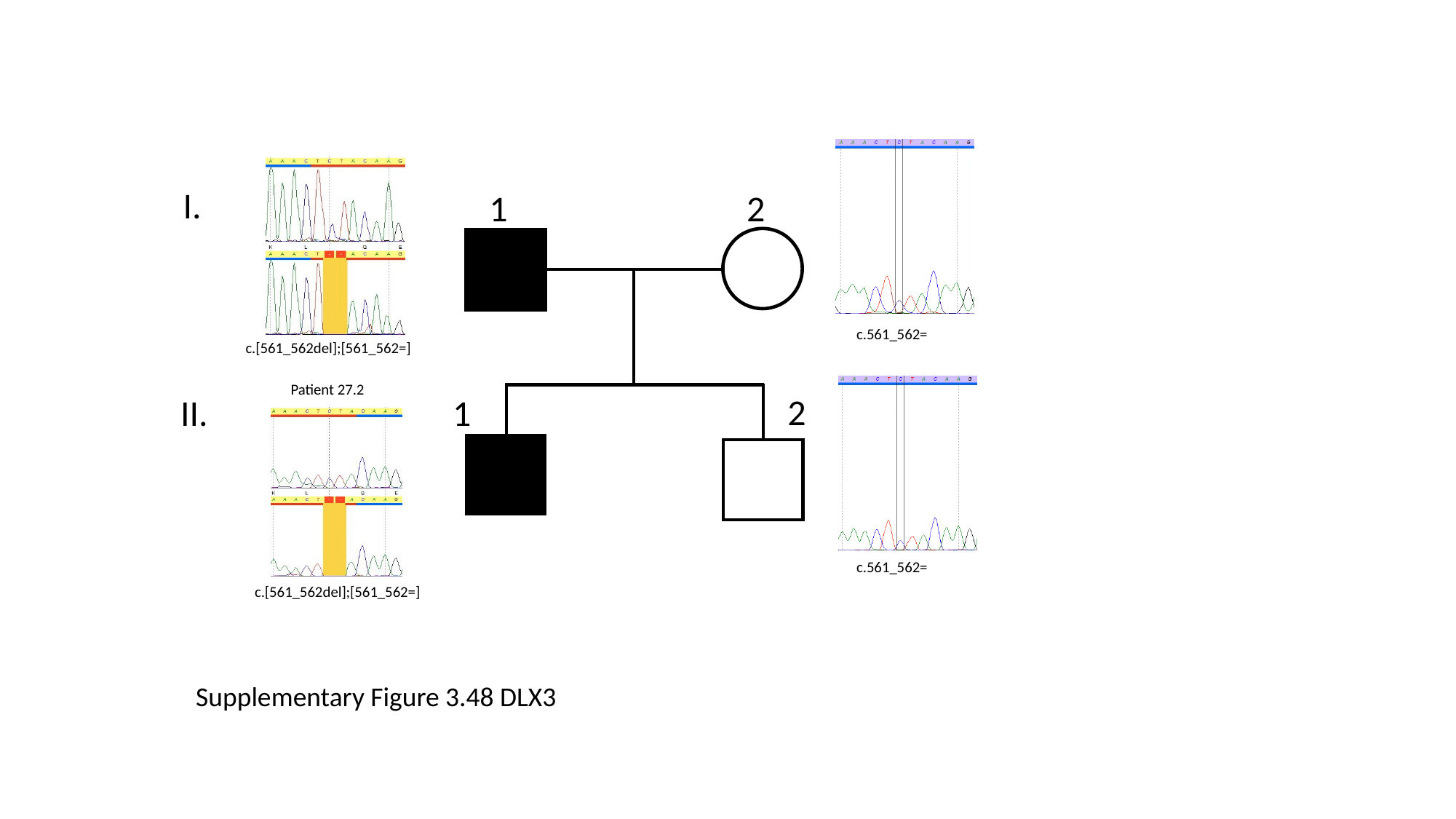

I.
1
2
c.561_562=
c.[561_562del];[561_562=]
Patient 27.2
2
II.
1
c.561_562=
c.[561_562del];[561_562=]
Supplementary Figure 3.48 DLX3
