# Supplementary material for: Amelogenesis imperfecta: Next-generation sequencing sheds light on Witkop’s classification
Source: Front Physiol. 2023 May 9;14:1130175. doi: 10.3389/fphys.2023.1130175 (PMC10205041; doi:10.3389/fphys.2023.1130175)
Supplement: Supplementary file 4 [file Table1.doc]

**GenodentV6.0 567 genes**

| **AAAS** | **ABCA5** | **ACP4** | **ACPT** | **ACVR1** | **ADAM10** | **ADAM15** | **ADAMTS1** | **ADAMTS10** | **ADAMTS2** | **ADGRV1** | **ADNP** | **AGL** | **AHCY** | **AIP** |
| --- | --- | --- | --- | --- | --- | --- | --- | --- | --- | --- | --- | --- | --- | --- |
| **AIRE** | **AKAP13** | **AKT1** | **ALDH3A2** | **ALPL** | **ALX3** | **AMBN** | **AMELX** | **AMELY** | **AMER1** | **AMTN** | **ANKH** | **ANKRD11** | **ANTXR1** | **ANTXR2** |
| **AP1G2** | **AP3B1** | **APAF1** | **APC** | **AR** | **ARHGAP6** | **ARID1B** | **ATP6V0A2** | **ATP6V1B2** | **ATP6V1C2** | **ATR** | **ATRIP** | **ATRX** | **AXIN2** | **B3GAT3** |
| **B4GALT7** | **BANF1** | **BAZ1B** | **BCOR** | **BLM** | **BMP1** | **BMP2** | **BMP4** | **BRAF** | **C1R** | **C1S** | **C4ORF26** | **CA2** | **CACNA1S** | **CACNB2** |
| **CAMK4** | **CARD9** | **CASP14** | **CCBE1** | **CCDC134** | **CCDC8** | **CD96** | **CDC6** | **CDH1** | **CDH23** | **CDH3** | **CDKN1C** | **CDON** | **CELSR1** | **CELSR3** |
| **CENPJ** | **CEP152** | **CFDP1** | **CHD7** | **CHPF** | **CHST3** | **CHSY1** | **CHUK** | **CIAPIN1** | **CIB2** | **CKAP2L** | **CLCN7** | **CLDN1** | **CLDN10** | **CLDN15** |
| **CLDN16** | **CLDN19** | **CLDN3** | **CLEC7A** | **CLRN1** | **CNNM4** | **COG6** | **COL10A1** | **COL11A1** | **COL12A1** | **COL15A1** | **COL17A1** | **COL1A1** | **COL1A2** | **COL24A1** |
| **COL2A1** | **COL3A1** | **COL5A1** | **COL5A2** | **COL6A1** | **COL7A1** | **COL9A1** | **COL9A2** | **COX7B** | **CREB3L1** | **CREBBP** | **CROT** | **CRTAP** | **CRTC2** | **CSNK1A1** |
| **CSPP1** | **CTNNB1** | **CTNND1** | **CTSC** | **CTSK** | **CUL7** | **CYBA** | **CYBB** | **CYP27B1** | **DCAF17** | **DEPTOR** | **DFNB31** | **DGKG** | **DHCR24** | **DHCR7** |
| **DHODH** | **DKC1** | **DLL1** | **DLX3** | **DLX5** | **DMP1** | **DMTF1** | **DNM1** | **DOCK8** | **DOK2** | **DSG4** | **DSP** | **DSPP** | **DVL1** | **DVL3** |
| **EDA** | **EDAR** | **EDARADD** | **EFNB1** | **EHMT1** | **EIF2AK1** | **ELANE** | **ELN** | **EMR2** | **ENAM** | **ENPP1** | **EP300** | **ERCC3** | **ERCC4** | **ERCC8** |
| **EVC** | **EVC2** | **EXT1** | **EXT2** | **EYA1** | **EZR** | **FADD** | **FAM111B** | **FAM20A** | **FAM20B** | **FAM20C** | **FAM83H** | **FBN1** | **FERMT1** | **FERMT3** |
| **FGD1** | **FGF10** | **FGF13** | **FGF23** | **FGF3** | **FGF8** | **FGF9** | **FGFR1** | **FGFR2** | **FGFR3** | **FKBP10** | **FLNA** | **FLNB** | **FN1** | **FOXC1** |
| **FRAS1** | **FREM2** | **G6PC** | **GALC** | **GALNS** | **GALNT3** | **GAS1** | **GBP5** | **GDF5** | **GJA1** | **GJB3** | **GJB4** | **GJB6** | **GLA** | **GLB1** |
| **GLI2** | **GLI3** | **GNAS** | **GOLIM4** | **GORAB** | **GPC3** | **GPR68** | **GREM1** | **GREM2** | **GRHL2** | **GRHL3** | **GRIP1** | **GTF2I** | **GTF2IRD1** | **H19** |
| **H1FNT** | **HAX1** | **HCCS** | **HEMK1** | **HENMT1** | **HMBS** | **HMCN1** | **HNF1B** | **HOXB13** | **HOXD13** | **HRAS** | **HSPG2** | **IBSP** | **IDS** | **IDUA** |
| **IFIH1** | **IFITM5** | **IFT122** | **IFT140** | **IFT20** | **IFT43** | **IGF1R** | **IGSF3** | **IKBKG** | **IL11RA** | **IL17F** | **IL17RA** | **INSR** | **IPO4** | **IRF6** |
| **IRF8** | **IRX5** | **ITGA11** | **ITGA6** | **ITGB2** | **ITGB4** | **ITGB6** | **ITPR3** | **JAG1** | **KAL1** | **KANSL1** | **KAT6B** | **KATNB1** | **KAZN** | **KCNH1** |
| **KCNJ1** | **KCNJ2** | **KCTD1** | **KDF1** | **KDM6A** | **KIF7** | **KISS1** | **KISS1R** | **KL** | **KLK4** | **KMT2C** | **KMT2D** | **KRAS** | **KREMEN1** | **KRT14** |
| **KRT16** | **KRT17** | **KRT5** | **KRT6A** | **KRT6B** | **KRT6C** | **KRT83** | **LAMA3** | **LAMB2** | **LAMB3** | **LAMC2** | **LEF1** | **LEMD3** | **LEPRE1** | **LIFR** |
| **LIMK1** | **LMNA** | **LONP1** | **LRFN2** | **LRP4** | **LRP5** | **LRP6** | **LSS** | **LTBP2** | **LTBP3** | **LTF** | **LYSMD4** | **LYST** | **MAP2K1** | **MAP2K2** |
| **MASP1** | **MBTPS2** | **MED12** | **MED25** | **MEGF8** | **MEPE** | **MID1** | **MMP1** | **MMP14** | **MMP2** | **MMP20** | **MMP9** | **MPPE1** | **MSX1** | **MSX2** |
| **MTERF2** | **mTOR** | **MUTYH** | **MYCBP2** | **MYO1H** | **MYO7A** | **NAA10** | **NACC1** | **NCF1** | **NCF2** | **NCF4** | **NDN** | **NFKBIA** | **NHS** | **NIPBL** |
| **NKG7** | **NLRP1** | **NOD2** | **NOP10** | **NOTCH2** | **NOTCH3** | **NOTUM** | **NRAS** | **NSD1** | **NSUN2** | **NTRK1** | **OBSL1** | **OCRL** | **ODAM** | **OFD1** |
| **ORAI1** | **ORC1** | **OSTM1** | **PAX3** | **PAX9** | **PCDH15** | **PCNT** | **PDGFRB** | **PDZD7** | **PEX1** | **PEX26** | **PEX6** | **PHEX** | **PHKA2** | **PIGA** |
| **PIGL** | **PIK3CA** | **PIK3R1** | **PITX2** | **PKP1** | **PLEC** | **PLEKHM1** | **PLG** | **PLK4** | **PLOD1** | **PLXNB1** | **PLXNB2** | **PLXNB3** | **PLXND1** | **POC1A** |
| **POLD1** | **POLR1C** | **POLR1D** | **POLR3A** | **POLR3B** | **PORCN** | **PPIB** | **PRKAR1A** | **PROK2** | **PROKR2** | **PSAP** | **PTCH1** | **PTCH2** | **PTDSS1** | **PTH1R** |
| **PTHLH** | **PTPN11** | **PVRL1** | **PVRL4** | **RAB23** | **RAI1** | **RAPSN** | **RASGRP2** | **RBBP8** | **RBM28** | **RECQL4** | **RELT** | **REST** | **RFC2** | **RIMS4** |
| **RIN2** | **RMRP** | **RNF10** | **ROGDI** | **ROR2** | **RPS6KA3** | **RUNDC1** | **RUNX2** | **SALL4** | **SAMD12** | **SAT1** | **SATB2** | **SCARF2** | **SEC23A** | **SEC24D** |
| **SERPINF1** | **SERPING1** | **SERPINH1** | **SH3BP2** | **SH3PXD2B** | **SHH** | **SIX3** | **SLC10A7** | **SLC13A5** | **SLC20A1** | **SLC20A2** | **SLC24A4** | **SLC26A2** | **SLC29A3** | **SLC34A1** |
| **SLC34A2** | **SLC34A3** | **SLC35C1** | **SLC37A4** | **SLC39A13** | **SLC9A3R1** | **SMAD3** | **SMARCAL1** | **SMARCD2** | **SMG9** | **SMO** | **SMOC2** | **SNRPN** | **SNX33** | **SOS1** |
| **SOST** | **SOX10** | **SOX11** | **SOX18** | **SOX2** | **SOX21** | **SOX9** | **SP6** | **SP7** | **SPARC** | **SPARCL1** | **SPECC1L** | **SPP1** | **SPRED1** | **SPRY4** |
| **SQSTM1** | **SSUH2** | **STAT1** | **STAT3** | **STIM1** | **SUFU** | **SUMO1** | **SUOX** | **TACR3** | **TBCE** | **TBX1** | **TBX2** | **TBX22** | **TBX3** | **TCEAL7** |
| **TCIRG1** | **TCOF1** | **TCTEX1D2** | **TERC** | **TERT** | **TFAP2A** | **TFAP2B** | **TGFA** | **TGFB1** | **TGFB2** | **TGFB3** | **TGFBR1** | **TGFBR2** | **TGIF1** | **THRA** |
| **TINF2** | **TMCO1** | **TMEM165** | **TMEM38B** | **TNFRSF11A** | **TNFRSF11B** | **TNFSF11** | **TP63** | **TRAF6** | **TRIM37** | **TRIP10** | **TRIP11** | **TRPS1** | **TRPV3** | **TSC1** |
| **TSC2** | **TSPEAR** | **TUFT1** | **TWIST1** | **TWIST2** | **UBB** | **UBE3B** | **UBR1** | **UHRF1** | **USH1C** | **USH1G** | **USH2A** | **USP19** | **VAV1** | **VDR** |
| **VIPAS39** | **VPS13A** | **VPS13B** | **VPS33B** | **VPS4B** | **WDR19** | **WDR35** | **WDR6** | **WDR72** | **WDR83** | **WHSC1** | **WISP3** | **WNT1** | **WNT10A** | **WNT10B** |
| **WNT3** | **WNT5A** | **WRN** | **XPR1** | **ZEB1** | **ZEB2** | **ZFHX4** | **ZFPM1** | **ZIC2** | **ZMPSTE24** | **ZNF469** | **ZNF878** |  |  |  |
