# Supplementary material for: Amelogenesis imperfecta: Next-generation sequencing sheds light on Witkop’s classification
Source: Front Physiol. 2023 May 9;14:1130175. doi: 10.3389/fphys.2023.1130175 (PMC10205041; doi:10.3389/fphys.2023.1130175)
Supplement: Supplementary file 5 [file Table2.docx]

| Target gene | Target exon/intron | Transcript | Forward sequence | Reverse sequence |
| --- | --- | --- | --- | --- |
| COL17A1 | Intron 20 | NM_000494.3 | GATACTGCCAGAGTACCTGAA | CTCTCCAGAGCCTAAAACAAG |
| COL17A1 | Exon 48 | NM_000494.3 | TGCTCTGATCTGCTTGCTGAGT | ACAGGTTTGAACGGCTCTGA |
| COL7A1 | Exon 28 | NM_000094.3 | GAGAGGTGACATATTCAGCCCCAT | GTGGGGATAAGCCAGTCAG |
| COL7A1 | Exon 30 | NM_000094.3 | GAGAGGTGACATATTCAGCCCCAT | GTGGGGATAAGCCAGTCAG |
| LAMB3 | Exon 3 | NM_000228.3 | CCTCTTGCCCAACCAACAG | TGGAAGCTTGAGTGAGGGAAAGG |
| LAMB3 | Exon 23 | NM_000228.3 | CCTCTACACATGCTACCTCCAGTTA | CATGAAAGTCTCCTGGAGATGGAA |
| LAMB3 | Intron 11 | NM_000228.3 | TCCTACTGCGGTGGCTGCTGTT | TGACTCAGTCCCACCTTCCAA |
| LAMB3 | Exon 14 | NM_000228.3 | TCTTGGCCCTAGCCTGTGACT | ACCAGCATGCCCGGTACTGGAA |
| LAMB3 | Exon 20 | NM_000228.3 | TACAGAGGGCTGGGAGACAGGA | CACAAGACCAGAAAATCCAAG |
| LAMC2 | Exon 4 | NM_002336.2 | AGTGAGTCCTTAAGTGTGTGAGA | GAAGGTCATCAACGTTTGTCAAGTA |
| ENAM | Intron 8 | MN_031889.2 | GGATTGCCAACAGATGCAGCCATA | CGGAAGCTTGAGCTACAGGTTCAT |
| ENAM | Intron 8 | MN_031889.2 | GGATTGCCAACAGATGCAGCCATA | CGGAAGCTTGAGCTACAGGTTCAT |
| ENAM | Intron 8 | MN_031889.2 | GGATTGCCAACAGATGCAGCCATA | CGGAAGCTTGAGCTACAGGTTCAT |
| ENAM | Exon 9 | NM_031889.2 | AAAGCTGAGCAGTGGTCAGAA | GCCCCATGACAGTACCAGTGAAAT |
| AMELX | Exon 2 | NM_182680.1 | CCCAACCTTTAGAGCCAACT | GGTCCCTTCCAAAAGCTTCCT |
| AMELX | Exon 6 | NM_182680.1 | GTCTACTCCACATGCAGACA | CTCTAGATCCTTGGTTGTCG |
| AMELX | Exon 6 | NM_182680.1 | GTCTACTCCACATGCAGACA | CTCTAGATCCTTGGTTGTCG |
| AMELX | Exon 6 | NM_182680.1 | GTCTACTCCACATGCAGACA | CTCTAGATCCTTGGTTGTCG |
| AMELX | Exon 6 | NM_182680.1 | GTCTACTCCACATGCAGACA | CTCTAGATCCTTGGTTGTCG |
| AMELX | Exon 6 | NM_182680.1 | GTCTACTCCACATGCAGACA | CTCTAGATCCTTGGTTGTCG |
| AMELX | Exon 6 | NM_182680.1 | GTCTACTCCACATGCAGACA | CTCTAGATCCTTGGTTGTCG |
| AMBN | Intron 1 | NM_016519.5 | TGTGGACTAATTGCAGGAGCAGAGA | GGATTACAACAAGCAATAATTCAGC |
| AMBN | Exon 8 | NM_016519.5 | GCAAACTCTTGGGTCATACCTC | GGAACCCATTATACTTCATATT |
| MMP20 | Exon 1 | NM_004771.3 | ATCCTTGCTCGGAGGGTCCAGA | TTCACAAGCCACCTGGAATTCT |
| MMP20 | Exon 3 | NM_004771.3 | GTACCGGATTATCCCAACTGTCTC | AGGTCATGTGGCTAACGGGAAA |
| MMP20 | Exon 1 | NM_004771.3 | ATCCTTGCTCGGAGGGTCCAGA | TTCACAAGCCACCTGGAATTCT |
| MMP20 | Intron 6 | NM_004771.3 | GGCAAGAGCAAAGGGCATTTAGTG | CAACCTGAGGACAAAGAGCAACTGA |
| MMP20 | Exon 1 | NM_004771.3 | ATCCTTGCTCGGAGGGTCCAGA | TTCACAAGCCACCTGGAATTCT |
| MMP20 | Exon 10 | NM_004771.3 | AACTGCCACCTGACAAATTCACAA | TTACAATATATGTCATGGAATCCAC |
| MMP20 | Exon 2 | NM_004771.3 | CTTCAGTGACAAATAAGTGATCC | GTTCTTATTCTTATGGTTGTGAGG |
| MMP20 | Intron 6 | NM_004771.3 | GGCAAGAGCAAAGGGCATTTAGTG | CAACCTGAGGACAAAGAGCAACTGA |
| MMP20 | Exon 4 | NM_004771.3 | GTGGGGTAGGTTTTGCTATCGAAT | AGGGGATGACTGGTTCTTCAGATG |
| MMP20 | Intron 6 | NM_004771.3 | GGCAAGAGCAAAGGGCATTTAGTG | CAACCTGAGGACAAAGAGCAACTGA |
| MMP20 | Intron 6 | NM_004771.3 | GGCAAGAGCAAAGGGCATTTAGTG | CAACCTGAGGACAAAGAGCAACTGA |
| SLC24A4 | Intron 16 | NM_153647.3 | TGCTGGGATTTCTGGATGGATTGG | TGCTCTTGGCAAAGACTGGACT |
| FAM83H | Exon 5 | NM_198488.3 | AGGAGTTCCGCATCCTCTTCG | AAGTCATCCGGGTCCGCGAAA |
| FAM83H | Exon 5 | NM_198488.3 | AGGAGTTCCGCATCCTCTTCG | AAGTCATCCGGGTCCGCGAAA |
| FAM83H | Exon 5 | NM_198488.3 | AGGAGTTCCGCATCCTCTTCG | AAGTCATCCGGGTCCGCGAAA |
| FAM83H | Exon 5 | NM_198488.3 | AGGAGTTCCGCATCCTCTTCG | AAGTCATCCGGGTCCGCGAAA |
| FAM83H | Exon 5 | NM_198488.3 | AGGAGTTCCGCATCCTCTTCG | AAGTCATCCGGGTCCGCGAAA |
| FAM83H | Exon 5 | NM_198488.3 | AGGAGTTCCGCATCCTCTTCG | AAGTCATCCGGGTCCGCGAAA |
| FAM83H | Exon 5 | NM_198488.3 | AGGAGTTCCGCATCCTCTTCG | AAGTCATCCGGGTCCGCGAAA |
| FAM83H | Exon 5 | NM_198488.3 | AGGAGTTCCGCATCCTCTTCG | AAGTCATCCGGGTCCGCGAAA |
| FAM83H | Exon 5 | NM_198488.3 | CTCTACCAGCAGCAGTACCA | GCCTGTGACGTGCTGAAGATG |
| WDR72 | Exon 11 | NM_182758.3 | ATGCTGGCAATATGAATCTGG | AATCTTGTTTAGTGCAGTCT |
| FAM83H | Exon 5 | NM_198488.3 | CTCTACCAGCAGCAGTACCA | GCCTGTGACGTGCTGAAGATG |
| LTBP3 | Intron 26 | NM_001130144.3 | AGCAATTCCTTCTGGGACACAAGC | ATATCTGAAGGTGAGGGCGACA |
| FAM20A | Exon 1 | NM_017565.3 | TTCCACCTCTGGCCCCAAGTA | GACTGGACTGTGAGGTCTGCAA |
| FAM20A | Exon 5 | NM_017565.3 | TTCCTTTCTCGCTGGTCATGCTCA | GGCGGGAAGCGTGTTCTTTGTA |
| FAM20A | Exon 6 | NM_017565.3 | CCTAGGTTTAAAGGCCCAGTGTTGA | TGCCCACACAGACTAGGTA |
| FAM20A | Intron 6 | NM_017565.3 | CCTAGGTTTAAAGGCCCAGTGTTGA | TGCCCACACAGACTAGGTA |
| FAM20A | Exon 6 | NM_017565.3 | CCTAGGTTTAAAGGCCCAGTGTTGA | TGCCCACACAGACTAGGTA |
| FAM20A | Intron 9 | NM_017565.3 | GAAAGCCTTAGACTTGAAGCCA | TTGTGAATCAGAGATTGGG |
| FAM20A | Exon 11 | MN_017565.3 | GCAAAGGACTGCAGGATACTGGAT | AGATTTCCCAGTGCACTCAGGAGA |
| DLX3 | Exon 3 | NM_005220.2 | TGACAGAGCAGAATTTGGAC | CTGGAAGATGATGAGCCATTTA |
| DLX3 | Exon 3 | NM_005220.2 | TGACAGAGCAGAATTTGGAC | CTGGAAGATGATGAGCCATTTA |
| TGFBR2 | Exon 7 | NM_003242.5 | ACTCAGTCAGCACATGTTAAAT | TGACAATGTCAAAGGCATAGAAT |
| CNNM4 | Exon 1 | NM_020184.3 | TCGAGCTCACCAAGGACCTG | TGTTGGCACCCACAGCCAG |
| SLC10A7 | Exon 3 | NM_001300842.3 | TGACCAGTGCTTTGGTGCATCT | CACAGGATGAAGCATGAGGGTGTA |
| ROGDI | Exon 6 | NM_024589.2 | AACTCGCACAGACTCTTGTCTTGG | TGGTGAACAGTCACTCCAGCTT |
| ROGDI | Exon 6 | NM_024589.2 | AACTCGCACAGACTCTTGTCTTGG | TGGTGAACAGTCACTCCAGCTT |

**Supplementary Table 2: List of primer's sequences used for Sanger sequencing.**
